# Inhibition of CDKL3 downregulates STAT1 thus suppressing prostate cancer development

**DOI:** 10.1038/s41419-023-05694-3

**Published:** 2023-03-10

**Authors:** Qi Jiang, Juan Li, Jingyue Wang, Weibing Zhang

**Affiliations:** 1 Department of Urology, Shanghai General Hospital, Shanghai Jiao Tong University School of Medicine, 100 Haining Road, Shanghai, 200080 China; 2 Department of Medical Social Services, Zhongnan Hospital of Wuhan University, 169 Donghu Road, Wuhan, 430071 China; 3 Department of Urology, The Ninth Hospital of Wuhan, 20 Jilin street, Wuhan, 430081 China; 4 Department of Urology, Zhongnan Hospital of Wuhan University, 169 Donghu Road, Wuhan, 430071 China

**Keywords:** Prostate cancer, Cell biology

## Abstract

Prostate cancer poses a great threat to men’s health worldwide, yet its treatment is still limited by the unclear understanding of its molecular mechanisms. CDKL3 is a molecule with a recently discovered regulatory role in human tumors, and its relationship with prostate cancer is unknown. The outcomes of this work showed that CDKL3 was significantly upregulated in prostate cancer tissues compared with adjacent normal tissues, and was significantly positively correlated with tumor malignancy. Knockdown of CDKL3 levels in prostate cancer cells significantly inhibited cell growth and migration and enhanced apoptosis and G2 arrest of the cell cycle. Cells with lower CDKL3 expression also had relatively weaker in vivo tumorigenic capacity as well as growth capacity. Exploration of downstream mechanisms of CDKL3 may regulate STAT1, which has co-expression characteristics with CDKL3, by inhibiting CBL-mediated ubiquitination of STAT1. Functionally, STAT1 is aberrantly overexpressed in prostate cancer and has a tumor-promoting effect similar to that of CDKL3. More importantly, the phenotypic changes of prostate cancer cells induced by CDKL3 were dependent on ERK pathway and STAT1. In summary, this work identifies CDKL3 as a new prostate cancer-promoting factor, which also has the potential to be a therapeutic target for prostate cancer.

## Introduction

Prostate cancer is one of the most common malignancies worldwide. Among all male cancer diseases in western countries, prostate cancer ranks second in incidence and sixth in mortality, accounting for ~14% of all cancer cases in men [1, 2]. In recent years, with the aggravation of the aging population in China, the incidence of prostate cancer also showed an increasing trend year by year and has become one of the malignant tumors that seriously affect the health of middle-aged and elderly men [3]. At present, serum PSA score, Gleason score, and tumor stage are the most important prognostic factors for prostate cancer and play an important role in guiding clinical diagnosis and treatment decisions [4, 5]. Nowadays, androgen deprivation therapy (ADT) is the preferred treatment strategy for prostate cancer, and its efficacy is particularly significant in the early stages of the tumor [6, 7]. However, a large number of clinical data showed that, in the early stage of ADT, the androgen receptor is inhibited due to loss-of-ligand, manifested as slow tumor growth. With the extension of treatment time, almost all prostate cancer patients initially sensitive to ADT will eventually develop hormone resistance, as well as the upregulation of prostate-specific antigen (PSA), a gene downstream of androgen receptor, which means the disease progression into castration-resistant prostate cancer [8]. However, there is still no mature subsequent treatment regimen [9]. Therefore, it is important to deeply explore the molecular mechanism of the occurrence and development of prostate cancer and find more and novel tumor regulators to help promote individualized and precise treatment of prostate cancer and improve the prognosis of patients.

CDKL3 (cyclin-dependent kinase-like 3) belongs to the family of cyclin-dependent kinases (CDKs). Recent studies showed that cell cycle-related proteins are abnormally expressed in many tumors (such as overexpression, localization change, etc.), which exhibit the characteristics of oncogenes and are closely related to the occurrence, development, diagnosis, treatment, and prognosis of human cancer. CDKL3 maps to human chromosome 5q31.1, which encodes a protein containing 455 amino acids and is also named NKIAMRE because it contains the “NKIAMRE” sequence, a universal cyclin binding domain [10, 11]. CDKL3 protein is localized in the cytoplasm, and it has been shown to be a component of a kinase complex, which functions to phosphorylate RNA polymerase II. At present, there are few studies on CDKL3, and most of them focus on the fields related to central nervous system development [12, 13]. Since CDKL3 gene sequence contains two highly conserved kinase sequences, it has mitogen-activated protein kinase (MAPK) and cyclin-dependent kinase (CDKs) activities, both of which are closely related to cell signal transduction and cell phenotype regulation [14]. It suggests that CDKL3 is likely to be associated with the regulation of malignant tumors [15]. Although a small number of studies have demonstrated the aberrant expression and progression regulation ability of CDKL3 in malignant tumors [16, 17], its function and mechanism in prostate cancer remain unclear.

In this study, we investigate the expression pattern and biological functions of CDKL3 in prostate cancer on clinical, cellular, and animal levels. A tissue microarray consisting of prostate cancer tissues and normal tissues was used for exhibiting the expression pattern. Loss-of-function experiments were conducted in vitro based on lentivirus-transfected cell models and in vivo based on xenografts formed by cell models. Moreover, a potential downstream mediator, STAT1, was identified by a preliminary mechanistic study, whose essential role in CDKL3-induced prostate cancer regulation was subsequently proved.

## Materials and methods

### Prostate cancer tissue microarray and immunohistochemistry (IHC) assay

Histological microarray of prostate cancer and normal prostate tissues (Shanghai Outdo Biotech Company) was applied for IHC analysis. After antigen retrieval, tissue slides were blocked with 3% H_2_O_2_ and following incubated with CDKL3 antibody at 37 °C for 1 h. After washing, the corresponding secondary antibody was added to identify the first antibody, and antibodies were available in Table S1. Then, slides were stained and images were captured. Staining percentage scores were classified as: 1 (1%–24%), 2 (25%–49%), 3 (50%–74%), and 4 (75%–100%) and staining intensity was scored 0 (signless color) to 3 (light yellow, brown and dark brown). The IHC assay outcomes were determined by the sum staining percentage scores and staining intensity scores. This study was approved by Ethics committee of Shanghai Jiao Tong University. Consent was collected from all patients included in this study.

### Cell lines, lentivirus, and transfection

Human normal prostate stromal immortalized cell line WPMY-1 and prostate cancer cell lines (DU 145, LNCaP, and PC-3, purchased from Cell Bank of the Chinese Academy of Sciences (Shanghai, China)) were used in our research. The cells were cultured in 1640 culture medium containing 10% FBS and maintained in an incubator at 37 °C with 5% CO_2_. shCtrl, shCDKL3, shSTAT1 lentiviruses (BR-V108 construct with GFP tag) and CDKL3- or CBL- overexpression vectors (LV-013 construct with GFP tag) were purchased by Shanghai YBR Bioscires Co., Ltd and the sequences were listed in Table S2. For cell transfection, lentiviruses (1 × 10^8^ TU/mL) were transfected into HEK293T cells upon the application of Lipofectamine 2000. After 48 h, the supernatant containing the recombinant lentiviruses was collected, filtered, and used to treat target cells. Following infection, cells were grown in media as usual. Puromycin (2 μg/ml) was added 48 h after infection. Infection efficiency was evaluated by observing green fluorescent protein (labeled on lentivirus vector) expression using a fluorescence microscope.

### Real-time qPCR

Total RNA was extracted using Trizol reagent, and Hiscript QRT supermix was used for reverse transcription. PCR was performed by using AceQ qPCR SYBR Green Master Mix and 2^−∆∆CT^ method was applied for analyzing with GAPDH served as an endogenous control. Primers used in PCR were listed in Table S3.

### Western blotting, co-immunoprecipitation, and ubiquitination assays

Cells were collected and lysed with ice-cold lysis buffer and quantified with a BCA protein detection kit (#T9300A, TaKaRa). Protein was separated by 10% SDS-PAGE and transferred to a PVDF membrane and incubated with TBST-blocking liquid. The PVDF membrane was immunoblotted with primary antibodies (listed in Table S1) and corresponding secondary antibodies. The membrane was color-developed using enhanced ECL chemiluminescence Kit (#NEL105001EA, Perkin Elmer), and the signal band was quantified using ImageJ software. For the co-immunoprecipitation assay, cell lysates were incubated with anti-STAT1 antibody and protein A/G agarose beads at 4 °C. The immunocomplexes were then washed with 200 μl PBS twice. Both lysates and immunoprecipitants were examined by WB using the indicated primary antibodies.

For analyzing the ubiquitination level of STAT1 in DU 145 and PC-3 cells with or without CDKL3 knockdown or CBL overexpression, the whole-cell lysates were collected after the treatment of cells with 20 μM MG132 for 4 h. Then the lysates were subjected to immunoprecipitation with anti-STAT1 antibody for subsequent WB using an anti-ubiquitin antibody (Table S1).

### Cell proliferation assay

Cells were seeded in 96-well plates in triplicate after lentivirus transfection and plates were scanned by Celigo Image Cytometer (Nexcelom) for consecutive five days at the same time point. The curve of cell number fold changes with time in the experimental group and control group was then drawn. The effect of CDKL3 gene knockdown on cell proliferation was verified by detecting cell viability using MTT assay. Cells were stained by MTT, and OD490 value was scanned using a microplate reader (#M2009PR, Tecan infinite) for consecutive 5 days.

### Flow cytometry assay

Lentivirus transfection cells were suspended with binding buffer, then cells were stained by Annexin V-APC for 15 min without light and FACS Calibur (BD Biosciences) was used to assess the apoptotic rate. For cell cycle detection, the cell was fixed with 4 °C pre-cooled 70% ethanol for 1 h. After washing with 4 °C PBS, cells were stained by PI staining solution (40 × PI, 2 mg/mL: 100 × RNase, 10 mg/mL: 1 × PBS = 25:10:1000), and the cell cycle was detected by FACS Calibur (BD Biosciences).

### Colony formation assay

Transfected cells were seeded in a six-well plate in triplicate and cultured for about eight days. After culturing, the colonies were fixed in 4% paraformaldehyde for 60 min, then, colonies were stained with 500 μL GIEMSA solution for 30 min. Photographs were collected, and the colonies number was counted.

### Wound-healing assay

Transfected cells were seeded into 96-well plates and cultured with serum-free medium for 72 h. Wounds across the monolayer were made using a 96-wounding replicator with debris was washed carefully. Photographs were taken by fluorescence micrograph at 0 h, 24 h, and 48 h for migration rate calculation.

### Transwell assay

Lentiviral vector infected cells (5 × 10^4^ cell/well) were seeded into a 12-well cell culture insert in triplicate which contains 8 μm polycarbonate membrane serum-free medium. 30% FBS culture medium was added into the lower chamber. After 16 h culturing, cells migrate and adhere to the bottom of polycarbonate membrane and were stained and pictures were obtained with a microscope (Olympus).

### PrimeView human gene expression array

Gene expression profiles were detected by PrimeView Human Gene Expression Array. In brief, total RNA was extracted from shCtrl and shCDKL3 DU 145 cells using Trizol Reagent following the manufacturer’s instructions and further purified by RNeasy mini kit (#74106, QIAGEN) and RNase-Free DNase Set (#79254, QIAGEN). Affymetrix human GeneChip 3’ IVT PLUS Reagent Kit (#902416) was used to obtain biotin-labeled cRNA and the outcomes were scanned by Affymetrix Scanner 3000 (Affymetrix, Santa Clara, CA, USA). Raw data statistical significance assessment was accomplished using a Welch t-test with Benjamini-Hochberg FDR (|Fold Change|≥ 1.3 and FDR< 0.05 as significant).

### Human apoptosis antibody array

The experiment was performed to explore the expression of the related proteins in the human apoptosis pathway in DU 145 cells with or without CDKL3 knockdown. Total proteins were extracted from lentivirus-infected DU 145 cells. Each array antibody membrane was blocked, then incubated with the proteins overnight at 4 °C. After washing, the proteins were proceeded to incubate with HRP-linked streptavidin for another 1 h. Enhanced chemiluminescence (ECL) (Amersham, Chicago, IL, USA) was used for visualizing and the signal densities were analyzed with ImageJ software.

### Xenograft model

This study was approved by the Ethics Committee of Shanghai Jiao Tong University. Four-week-old female BALB/c nude mice were obtained from Beijing Vital River Laboratory Animal Technology Co., Ltd (Beijing, China). Mice were randomly assigned to two groups (shCtrl group and shCDKL3 group) and then injected subcutaneously with 4 × 10^6^ shCtrl and shCDKL3 cells (DU 145 and PC-3 cells) suspension in a volume of 0.2 mL, respectively. The size and weight of the tumor were monitored once every 4 days, starting at day 6 post injection. The IVIS Spectrum Imaging System (Perkin Elmer, Waltham, MA, USA) was used to collect the in vivo fluorescence images. All mice were sacrificed by cervical dislocation and tumors were harvested. Tumor volumes were measured using a Vernier caliper. Mice tumor tissues were stained using Ki67 antibody. Stained slides were examined with a microscopic at ×100 and ×200 objective lens.

### Statistical analysis

The data were expressed as mean ± standard deviation (SD). Statistical analyses (SPSS 20.0 and GraphPad Prism software 7.0) were accomplished using Student’s *t* test, Sign test, Mann–Whitney *U* test, and Spearman rank correlation analysis. *P* < 0.05 were valued as significant.

## Results

### CDKL3 is upregulated in prostate cancer

**Fig. 1:**
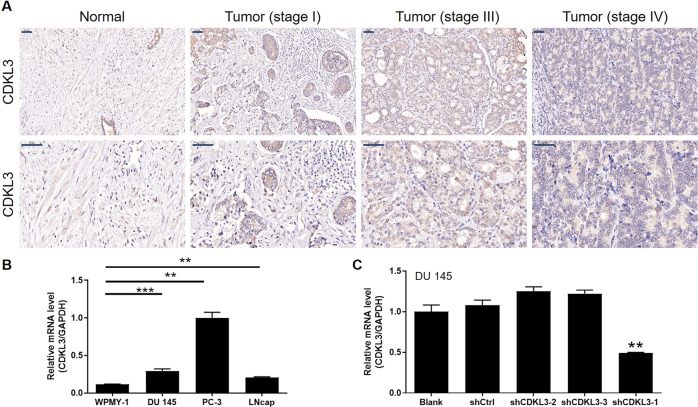
CDKL3 is upregulated in prostate cancer. **A** The immunohistochemical analysis using CDKL3 antibody was performed to show the protein level of CDKL3 in prostate cancer tissues with different stages and para-carcinoma normal tissues. Scale bar = 50 μm. **B** The expression of CDKL3 was detected in immortalized human normal prostate stromal cell line WPMY-1 and prostate cancer cell lines including DU 145, PC-3, and LNcap. **C** The most efficient shRNA for silencing CDKL3 was screened by qPCR assay. Data were presented as mean with standard deviation. **P < 0.01, ***P < 0.001.

As the beginning of this study, we preliminarily investigated the role of CDKL3 in prostate cancer progression by analyzing its expression in 156 prostate cancer tumor tissues and 32 adjacent normal tissues. We divided the tissues into a high CDKL3 expression group and a low CDKL3 expression group based on the median IHC score, and the resulting statistical analysis showed that CDKL3 expression was significantly higher in prostate cancer tumor tissues than in adjacent normal tissues (Table 1), while Fig. 1A showed some typical images. By further including tumor pathological indicators in statistical analysis, the results showed that upregulated CDKL3 expression was positively correlated with the malignancy of the tumor (tumor stage as well as Gleason score) (Tables 2 and S4, Fig. 1A). These results implied that CDKL3 may have a key role in the progression of prostate cancer.

**Table 1:** Expression patterns of CDKL3 in prostate cancer tissues and normal tissues revealed in immunohistochemistry analysis.

CDKL3 expression	Tumor tissue		Normal tissue	
	Cases	Percentage	Cases	Percentage
Low	83	53.2%	27	84.4%
High	73	46.8%	5	15.6%

*P* < 0.001

**Table 2:** Relationship between CDKL3 expression and tumor characteristics in patients with prostate cancer.

Features	No. of patients	CDKL3 expression		*P* value
		low	high	
All patients	156	83	73	
Age (years)				0.542
≤69	81	45	36	
>69	75	38	37	
Gleason Score				0.033
<8	60	37	23	
≥8	89	39	50	
Grade				0.077
1	12	12	0	
2	38	17	21	
3	99	47	52	
Tumor infiltration				0.123
T1	2	0	2	
T2	68	46	22	
T3	37	19	18	
T4	6	2	4	
lymphatic metastasis (N)				0.581
N0	105	63	42	
N1	8	4	4	
Stage				0.027
I	14	12	2	
II	56	34	22	
III	31	15	16	
IV	12	6	6	

### In vitro study proves that knockdown of CDKL3 inhibits prostate cancer development

**Fig. 2:**
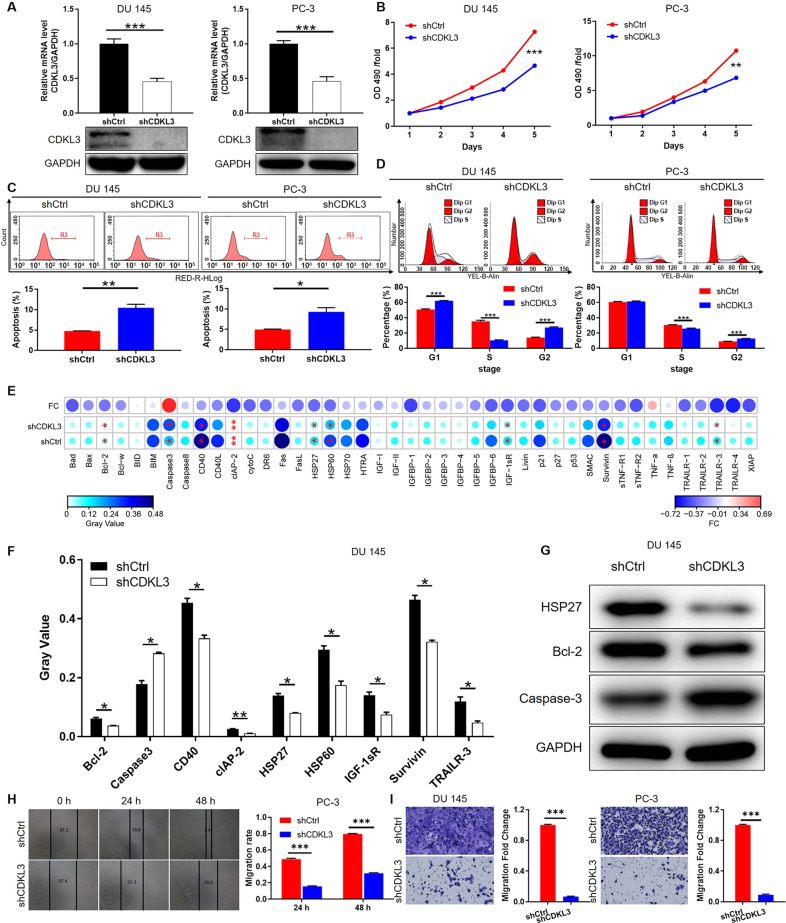
Silencing CDKL3 regulates cell phenotype in vitro. **A** The verification of the successful construction of CDKL3 knockdown prostate cancer models was carried out on mRNA and protein levels by qPCR and western blot, respectively. **B** The comparison of cell proliferation rate in shCtrl and shCDKL3 groups of prostate cancer cells was done by MTT assay. **C** The differential cell apoptosis of shCtrl and shCDKL3 groups of prostate cancer cells was analyzed by flow cytometry. **D** The difference in cell cycle distribution of shCtrl and shCDKL3 groups of prostate cancer cells was shown by flow cytometry. **E**, **F** A human apoptosis antibody array was used to detect the effects of CDKL3 knockdown on the expression levels of apoptosis-related proteins. The expression of apoptosis-related proteins included in the antibody array was separately shown (**E**). The statistical significance of some differentially expressed proteins was analyzed and displayed (**F**). **G** The expression of Bcl-2, HSP27, and Caspase-3 was detected in shCtrl and shCDKL3 groups of DU 145 cells. **H**, **I** The change of cell motility of shCtrl and shCDKL3 groups of prostate cancer cells was evaluated by wound-healing assay (**H**) and transwell assay (**I**). Data were presented as mean with standard deviation. *P < 0.05, **P < 0.01, ***P < 0.001.

Prior to in vitro studies, endogenous expression of CDKL3 in normal (WPMY-1) as well as tumor cells including DU 145, PC-3, and LNcap was detected by qPCR, which showed that CDKL3 expression was significantly lower in normal cells than in tumor cells (Fig. 1B), which was also consistent with clinical tissue-related results. Subsequently, we selected one of the three shRNAs targeting CDKL3 with the highest silencing efficiency (shCDKL3-1) for subsequent experiments (Fig. 1C). Validation experiments showed that both CDKL3 knockdown lentivirus (shCDKL3) as well as control lentivirus (shCtrl) could efficiently infect prostate cancer cells (Figure S1) and knockdown the mRNA as well as protein levels of CDKL3 (Fig. 2A). The cell proliferation level detected by MTT assay showed that the proliferation rate of cells with CDKL3 knockdown was significantly slowed down compared with the shCtrl group (Fig. 2B), while this regulatory effect may be related to the enhanced apoptosis (Fig. 2C) induced by CDKL3 knockdown as well as the G2 phase arrest of the cell cycle (Fig. 2D). Further examination of apoptosis-related antibody array showed that the regulation of apoptosis by CDKL3 may be related to the downregulation of Bcl-2, cIAP-2, CD40, HSP27, HSP60, IGF-1sR, Survivin, TRAILR-3 and the upregulation of Caspase-3 (Fig. S2A–C and 2E, F). Among them, the regulatory effects of CDKL3 on Bcl-2, HSP27, and Caspase-3 were further verified in both DU 145 and PC-3 cells (Fig. 2G and S3). Notably, the regulation of cell apoptosis, as well as Bcl-2, HSP27, and Caspase-3 by CDKL3 knockdown, was further verified in a third cell line LNcap (Figure S4). In addition to these, downregulation of CDKL3 expression not only significantly inhibited the level of cell proliferation but also showed strong interference with cell migration ability. The results of both Fig. 2H (scratch assay) and Fig. 2I (transwell assay) showed that prostate cancer cells in the shCDKL3 group had diminished mobility. In summary, in vitro loss-of-function experiments clearly point to the fact that CDKL3 may have an important role in the disease progression of prostate cancer.

### In vivo study verifies that knockdown of CDKL3 inhibits prostate cancer development

**Fig. 3:**
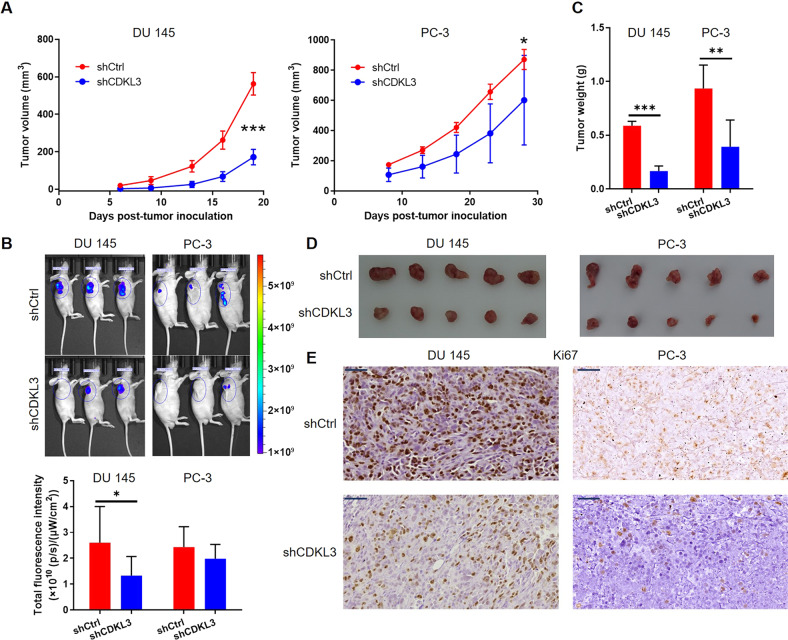
Silencing CDKL3 inhibits tumor growth in vivo. **A** The tumor growth curve was drawn based on the measurement and calculation of tumor volume. **B** The tumor growth was also represented by the in vivo detection of the fluorescent signals resulting from the GFP tag on the lentivirus vector. **C**, **D** The weights (**C**) of xenografts and photos (**D**) of xenografts were obtained after sacrificing the mice and removing the xenografts. **E** Sections of xenografts were prepared and subjected to IHC analysis for detecting Ki67 index. Scale bar = 50 μm. Data were presented as mean with standard deviation. *P < 0.05, **P < 0.01, ***P < 0.001.

Given the good results obtained by in vitro cell experiments, next, we constructed mouse xenograft models by subcutaneously transplanting DU 145 and PC-3 cells from the shCDKL3 and shCtrl groups into mice. From the tumor growth curve of the animal model, the growth rate of xenografts formed by shCDKL3 cells was significantly slower than that of xenografts formed by shCtrl cells (Fig. 3A). At the end-point of the experiment, tumor size detection using in vivo fluorescence imaging (using fluorescence intensity to characterize tumor size) as well as weighing and photography of tumors further showed that knockdown of CDKL3 expression not only inhibited the in vivo growth of tumors (Fig. 3B–D). Moreover, we analyzed Ki67 expression in the sections of xenografts using IHC, and its results also clearly indicated that the cell proliferation activity of tumors in the shCDKL3 group was relatively lower, which was consistent with the aforementioned results (Fig. 3F). In addition, CDKL3 expression was also detected by western blot in xenografts to assure whether the change of tumor growth resulted from CDKL3 knockdown (Figure S5).

### The exploration of downstream mechanism of CDKL3 in prostate cancer

**Fig. 4:**
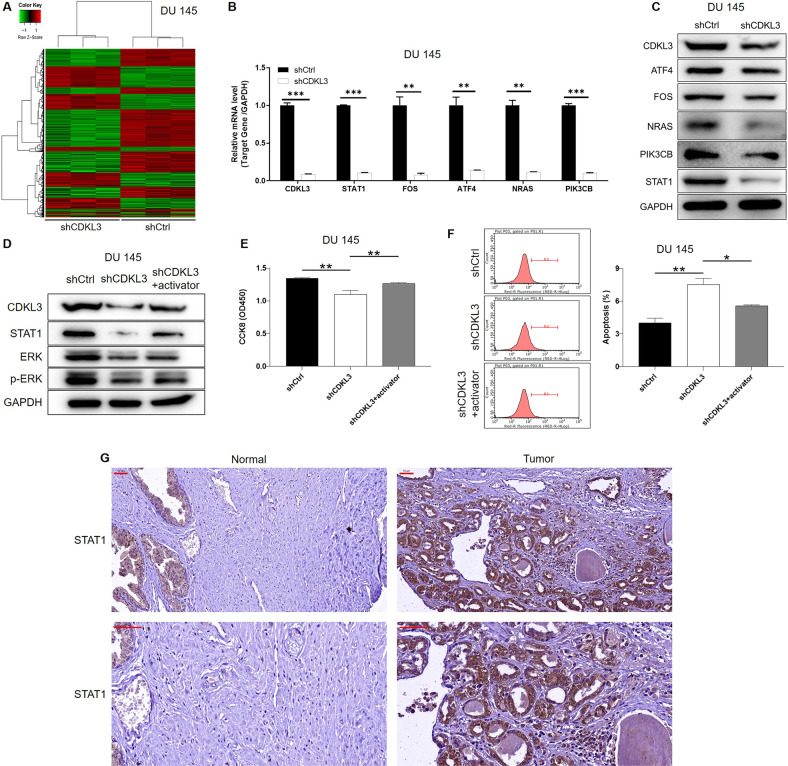
The exploration of regulatory mechanism of CDKL3 in prostate cancer. **A** A heatmap was drawn based on the significantly differentially expressed genes identified by a gene microarray analysis of DU 145 cells with or without CDKL3 knockdown. **B** The mRNA level of the selected ERK signaling pathway-related candidates was detected by qPCR in shCtrl and shCDKL3 DU 145 cells for verification. **C** The protein level of the selected candidates after qPCR verification was detected by western blot in shCtrl and shCDKL3 DU 145 cells for further verification. **D** The expression of STAT1, ERK, and p-ERK was detected in shCtrl, shCDKL3 and ERK activator (TBHQ, 20 μM) treated shCDKL3 DU 145 cells. **E** CCK8 assay was performed to evaluate the proliferation rate of indicated groups of DU 145 cells. **F** Flow cytometry was applied to assess the apoptosis of indicated groups of DU 145 cells. **G** The IHC analysis using STAT1 antibody was performed to show the protein level of STAT1 in prostate cancer tissues and para-carcinoma normal tissues. Scale bar = 50 μm. Data were presented as mean with standard deviation. *P < 0.05, **P < 0.01, ***P < 0.001.

Although the above results have fully indicated the role of CDKL3 in prostate cancer progression, its downstream regulatory mechanism remains unclear, which is the goal of our further study. Therefore, we applied a genechip to obtain and differentially analyze the transcriptome expression profiles of DU 145 cells in the shCtrl (*n* = 3) and shCDKL3 (*n* = 3) groups (Fig. 4A and S6A). According to the screening threshold of absolute expression fold change >1.3 while FDR < 0.05, a total of 1066 significantly upregulated genes and 1502 significantly downregulated genes were found and analyzed for enrichment in downstream tumor-related physiological functions as well as signaling pathways (Figure S6B, C). Notably, although not in the top rank, ERK signaling pathway was found to be enriched downstream of CDKL3. Therefore, several ERK-related factors that are significantly regulated by CDKL3 were selected for examination by qPCR and western blot, indicating that STAT1 expression was apparently decreased on both mRNA and protein levels in CDKL3 knockdown DU 145 cells (Fig. 4B, C). Moreover, it was demonstrated that CDKL3 knockdown could induce the de-activation of ERK pathway, which could be reversed by TBHQ (ERK activator) (Fig. 4D and S7A). Accordingly, the inhibition of cell proliferation and promotion of cell apoptosis induced by CDKL3 knockdown could both be attenuated by activating ERK pathway (Fig. 4E, F and S7B, C). Based on the above results, we concluded that STAT1 may be one of the key molecules mediating the regulation of prostate cancer by CDKL3, and its role in prostate cancer was also confirmed by its upregulation in prostate cancer tissues compared with adjacent normal tissues (Fig. 4G and Table S5) and its association with tumor size (Tables S6, S7).

### Knockdown of CDKL3 de-stabilizes STAT1 through promoting CBL-ubiquitination

**Fig. 5:**
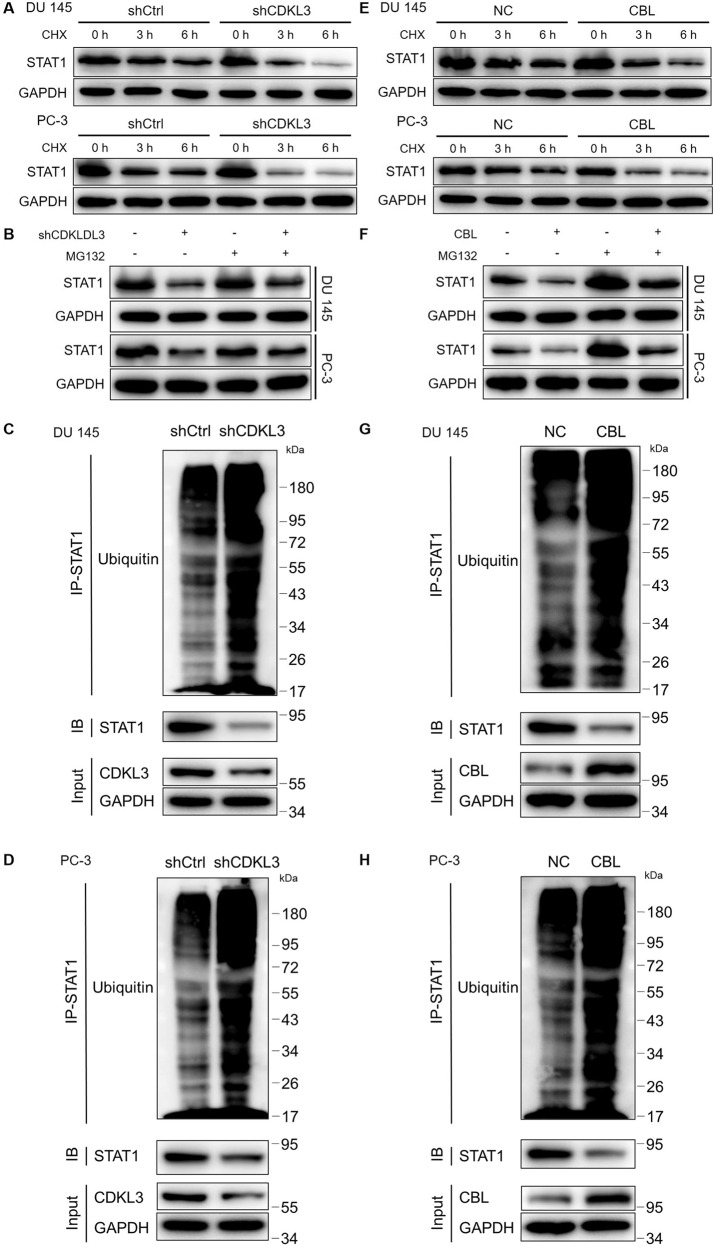
CDKL3 may regulate STAT1 expression through CBL-mediated ubiquitination. **A** A CHX-chase experiment was performed to test the protein stability of STAT1 in shCtrl and shCDKL3 DU 145 and PC-3 cells. **B** The expression of STAT1 was detected in shCtrl and shCDKL3 DU 145 and PC-3 cells with or without proteasome inhibitor MG132. **C**, **D** The ubiquitination levels in shCtrl and shCDKL3 DU 145 (**C**) and PC-3 (**D**) cells were assessed after precipitating immune-complex using indicated antibodies. **E** A CHX-chase experiment was performed to test the protein stability of STAT1 in NC and CBL overexpression DU 145 and PC-3 cells. **F** The expression of STAT1 was detected in NC and CBL overexpression DU 145 and PC-3 cells with or without proteasome inhibitor MG132. **G**, **H** The ubiquitination levels in NC and CBL overexpression DU 145 (**G**) and PC-3 (**H**) cells were assessed after precipitating immune-complex using indicated antibodies.

In the process of trying to explore the molecular mechanism of CDKL3 regulating STAT1 expression, we found out by accident that CDKL3 knockdown could decrease the protein stability of STAT1 (Fig. 5A), in which CHX (20 μM) was used to treat DU 145 and PC-3 cells for preventing protein biosynthesis. Moreover, the fact that treatment of MG132 (20 μM), an inhibitor of proteasome, could partially eliminate CDKL3-induced change of STAT1 protein stability, indicated the involvement of ubiquitin-proteasome system (UPS) (Fig. 5B). Indeed, protein ubiquitination assay showed that the ubiquitination level of STAT1 was dramatically elevated in CDKL3 knockdown cells (Fig. 5C, D). The above phenomenon suggested that CDKL3 may regulate STAT1 protein level by affecting its ubiquitination modification and the following proteasome-related degradation. Therefore, we consulted ubibrowser [18] for predicting the upstream E3 ligase of STAT1 (Figure S8A), and the validity of CBL as E3 ligase of STAT1 was verified by CHX-chase experiment (Fig. 5E), MG132 treatment experiments (Fig. 5F) and protein ubiquitination assay as for CDKL3 (Fig. 5G, H), showing the promotion of STAT1 ubiquitination and proteasome-relate degradation upon CBL overexpression. Furthermore, the mechanism by which CDKL3 influences the CBL-mediated ubiquitination of STAT1 was proposed to be related to the protein-protein interaction of CDKL3 and CBL (Figure S8B).

### Knockdown of STAT1 inhibits prostate cancer development and alleviated CDKL3 overexpression-induced promotion effects

**Fig. 6:**
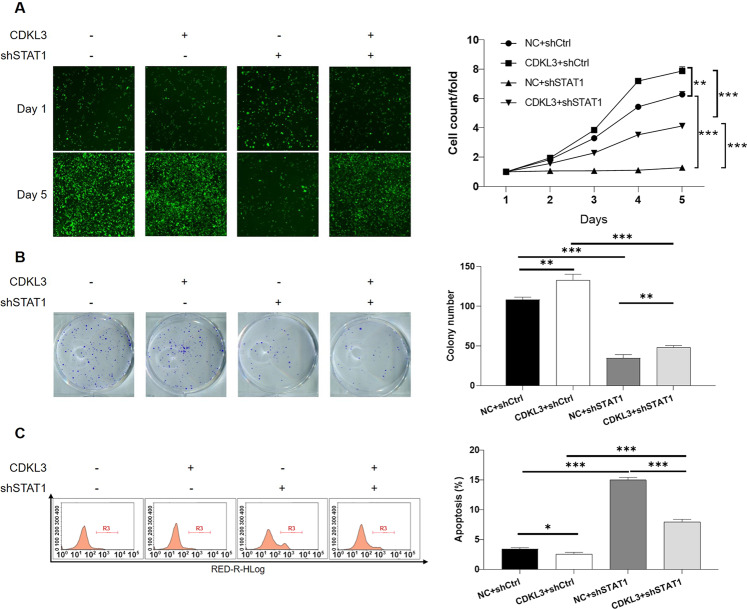
The existence of STAT1 is essential for the CDKL3-induced regulation of prostate cancer. **A** The cell proliferation rate of DU 145 cells with mere CDKL3 overexpression, mere STAT1 knockdown, or both, was evaluated by MTT assay. **B** The colony formation ability of DU 145 cells with mere CDKL3 overexpression, mere STAT1 knockdown, or both, was assessed by colony formation assay. **C** The percentage of apoptotic in DU 145 cells with mere CDKL3 overexpression, mere STAT1 knockdown, or both, was detected by flow cytometry. Data were presented as mean with standard deviation. *P* < 0.01, ***P < 0.001.

In order to determine whether STAT1 is involved in the regulation of prostate cancer by CDKL3, we constructed and screened shRNA targeting STAT1 silencing using a similar method as described previously (Figure S9) and used it, together with the corresponding control lentivirus (shCtrl) to infect DU 145 and PC-3 cells with or without CDKL3 overexpression, from which the four groups of cell models shown in Fig. S10, S11 were constructed and the expression levels of CDKL3 and STAT1 were verified. Subsequent cellular phenotyping assays showed the role of STAT1 in prostate cancer progression and its association with CDKL3. As shown in Fig. 6 and S12, knockdown of STAT1 expression inhibited the proliferation and colony formation ability of DU 145 and PC-3 cells, while inhibiting apoptosis, and these results conferred STAT1 typical characteristics of cancer-promoting factor. More importantly, the evidence provided in Fig. 6 and S12 clearly demonstrated that the enhanced cell proliferation/colony formation induced by CDKL3 overexpression as well as apoptosis inhibition is dependent on STAT1. Therefore, we can conclude that CDKL3 may promote the progression of prostate cancer through STAT1.

## Discussion

In this study, as far as we know, the participation of CDKL3 in the development of prostate cancer was investigated for the first time. The association between CDKL3 and tumor progression was clearly indicated by the CDKL3 upregulation in prostate cancer tissues and its correlation with Gleason score and tumor stage, both of which are important characteristics of prostate cancer. Because CDKL3 has similar kinase activity to MAPKs and CDKs, it has an important role in the growth and differentiation of neuronal cells, so it has received high attention in central nervous development-related research. Early studies have shown that loss-of CDKL3 function can lead to abnormal brain function by affecting neuronal process morphological development [19]. On the other hand, because of the accumulating evidence showing the critical role played by MAPKs and CDKs in the development and progression of malignant tumors, more and more studies began to focus on the functions of CDKL3 in the regulation of human cancers [14]. For example, studies by Ye et al. explored the clinical significance and regulatory effects of CDKL3 in esophageal squamous cell carcinoma (ESCC). They showed that CDKL3 exerts tumor-promotor-like functions in the development of ESCC, with upregulated expression in tumor tissues, a significant correlation with poor prognosis of patients, a distinct regulatory ability of malignant cell phenotypes [17]. Moreover, the autophagy-related gene ATG5 was subsequently identified as a potential downstream of CDKL3 in ESCC [16]. In osteosarcoma, CDKL3 was also found to be capable of affecting autophagy by activating Akt signaling pathway, as well as other downstream effects of Akt pathway such as cell growth and cell apoptosis [20]. Recently, Yan et al. demonstrated that circRNA TP53 could promote the expression of CDKL3 through competitively sponging miR-876-3p, thus promoting the development of colorectal cancer [21]. Moreover, CDKL3 was considered as a therapeutic target of curcumol, a major component extracted from the root of *Rhizoma Curcumae*, mediating the anticancer effects in cholangiocarcinoma cells [22]. Herein, the outcomes of in vitro and in vivo studies suggested similar regulatory functions of CDKL3 in prostate cancer as that in other types of human cancers. Prostate cancer cells with CDKL3 knockdown exhibited weakened proliferative activity, enhanced apoptosis, and decreased motility, while xenografts formed by CDKL3 knockdown cells showed distinctly slower growth rates than that formed by control cells. All these results suggested a key role of CDKL3 in the development of prostate cancer.

Signal transducer and activator of transcription 1 (STAT1) is an important transcription factor that can transduce cellular signals from the cytoplasm to the nucleus, thereby regulating the expression of downstream genes [23]. STAT1 has a variety of important biological functions in normal cells, such as regulating cell apoptosis and cell proliferation, stimulating the immune system, and regulating cell differentiation [23]. At the same time, STAT1 is an important member of the classical tumor regulatory signaling pathway JAK/STAT pathway and is also closely related to the ERK pathway [24]. Most studies to date have shown that STAT1 has a significant inhibitory effect on malignant tumors. Several studies have shown that the activation or expression of STAT1 is lost in cancer cells originating from various tissue types, and correspondingly has a positive correlation with the survival of patients [25]. For example, Liu et al. showed that the phosphorylated STAT1 could directly interact with the promotor of FOXM1 thus downregulating its expression, finally enhancing the sensitivity of pancreatic cancer cells to gemcitabine [25, 26]. However, many studies have shown contradictory observations, that is, STAT1 and/or p-STAT1 play a role as a cancer promoter in some malignancies and are negatively correlated with patient survival [25, 27, 28]. In colon cancer, STAT1-CCL5 axis was found to be upregulated and associated with accelerated cell proliferation [29]. Moreover, Chen et al. [30] indicated that Histone methyltransferase SETDB1 could successively upregulate the expression of STAT1 and CCND1/CDK6 pathway, thereby promoting cell proliferation of colorectal cancer cells. Although a tumor-promoting profile of PIAS1, an inhibitor of STAT1, as well as the tumor-inhibiting feature of STAT1 has been reported in prostate cancer [30], our study illustrated that STAT1 is a co-expression gene and potential downstream target of CDKL3, mediating the promotion of prostate cancer development. In tissue specimens collected from a local cohort of patients, STAT1 was characterized as an upregulated protein in tumor tissues compared with normal tissues. Knockdown of STAT1 could not only inhibit prostate cancer development in vitro, but also diminish the promotion effects induced by CDKL3 overexpression, highlighting the critical role of STAT1 as the mediator. Moreover, it has been well known that protein ubiquitination and deubiquitination play important roles in the regulation of proteins with key functions in tumor progression [31]. Correspondingly, STAT1 expression, as well as its functions in tumor development, has been reported to be regulated by protein ubiquitination [32]. In our study, it was also illustrated that CDKL3 knockdown may downregulate STAT1 protein level by elevating its CBL-mediated ubiquitination, thus promoting its proteasome-related degradation and decreasing its protein stability.

In conclusion, CDKL3 was identified to possess a STAT1-dependent tumor-promoting factor in the development and progression of prostate cancer, which could contribute to the improvement of targeted therapy for prostate cancer.

## Supplementary information

Table S1

Table S2

Table S3

Table S4

Table S5

Table S6

Table S7

Supplementary figure legends

Figure S1

Figure S2

Figure S3

Figure S4

Figure S5

Figure S6

Figure S7

Figure S8

Figure S9

Figure S10

Figure S11

Figure S12

Original Data File

Original Data File

cddis-author-contribution-form

aj-checklist

## Data Availability

All data generated or analyzed during this study are included in this published article and its supplementary information files.
