# Supplementary material for: Inhibition of CDKL3 downregulates STAT1 thus suppressing prostate cancer development
Source: Cell Death Dis. 2023 Mar 10;14(3):189. doi: 10.1038/s41419-023-05694-3 (PMC10006411; doi:10.1038/s41419-023-05694-3)
Supplement: Supplementary file 1 — Table S1 [file 41419_2023_5694_MOESM1_ESM.docx]

Table S1 Antibodies used in western blotting and IHC

| Primary antibodies | Dilution in WB | Source species | Company | Catalog No. |
| --- | --- | --- | --- | --- |
| CDKL3 | 1:1000 | Rabbit | Bioss | bs-5747r |
| GAPDH | 1:3000 | Rabbit | Bioworld | AP0063 |
| CDKL3 (co-IP) | 1:2000 | Rabbit | Thermo | PA5-72267 |
| CBL | 1:50/1:2000 | Rabbit | Proteintech | 25818-1-AP |
| HSP27 | 1:1000 | Rabbit | Abcam | ab109376 |
| Bcl-2 | 1:3000 | Rabbit | Proteintech | 12789-1-AP |
| Caspase-3 | 1:2000 | Rabbit | Abcam | ab32351 |
| ATF4 | 1:500 | Rabbit | Proteintech | 10835-1-AP |
| FOS | 1:1000 | Rabbit | CST | 2250S |
| NRAS | 1:500 | Rabbit | Abcam | ab154291 |
| PIK3CB | 1:1000 | Mouse | Proteintech | 67121-1-Ig |
| STAT1 | 1:1000 | Rabbit | Proteintech | 10144-2-AP |
| ATF4 | 1:500 | Rabbit | Proteintech | 10835-1-AP |
| ERK | 1:3000 | Rabbit | CST | 4695 |
| p-ERK | 1:500 | Rabbit | Abcam | ab201015 |
| ubiquitin | 1:2000 | Mouse | Santa Cruz | sc-8017 |
| Primary antibodies | Dilution in IHC | Source species | Company | Catalog No. |
| CDKL3 | 1:100 | Rabbit | bioss | bs-5747R |
| STAT1 | 1:200 | Rabbit | abcam | ab109320 |
| Ki67 | 1:300 | Rabbit | abcam | Ab16667 |
|  |  |  |  |  |
| Secondary antibody | Dilution |  | Company | Catalog No. |
| HRP Goat Anti-Rabbit IgG (WB) | 1:3000 |  | Beyotime | A0208 |
| HRP Goat Anti-Rabbit IgG (IHC) | 1:200 |  | Abcam | Ab111909 |
