# Supplementary material for: Inhibition of CDKL3 downregulates STAT1 thus suppressing prostate cancer development
Source: Cell Death Dis. 2023 Mar 10;14(3):189. doi: 10.1038/s41419-023-05694-3 (PMC10006411; doi:10.1038/s41419-023-05694-3)
Supplement: Supplementary file 2 — Table S2 [file 41419_2023_5694_MOESM2_ESM.docx]

Table S2 The target sequences

| Gene | No. | Target sequence (5'-3') |
| --- | --- | --- |
| CDKL3 | Pbr-10064 | GAGGAGATATCTCAGAACCAA |
| CDKL3 | Pbr-00158 | ACTAACTGTAATGGCTTGAAA |
| CDKL3 | Pbr-00159 | CACACAGTATTAGATGAGTTA |
| STAT1 | Pbr-11142 | ACAGAAAGAGCTTGACAGTAA |
| STAT1 | Pbr-11143 | GGAGGAATTGGAACAGAAATA |
| STAT1 | Pbr-11144 | GTGGCAAAGAGTGATCAGAAA |
|  |  |  |
