# Supplementary material for: Inhibition of CDKL3 downregulates STAT1 thus suppressing prostate cancer development
Source: Cell Death Dis. 2023 Mar 10;14(3):189. doi: 10.1038/s41419-023-05694-3 (PMC10006411; doi:10.1038/s41419-023-05694-3)
Supplement: Supplementary file 3 — Table S3 [file 41419_2023_5694_MOESM3_ESM.docx]

Table S3 Primers used in qPCR

| Gene | Forward primer sequence (5’-3’) | Reverse primer sequence (5’-3’) |
| --- | --- | --- |
| CDKL3 | TATCTGGGCTTTGGGCTGTA | TGGGGTGTTGAACTTGAGGA |
| GAPDH | TGACTTCAACAGCGACACCCA | CACCCTGTTGCTGTAGCCAAA |
| FOS | CAGACTACGAGGCGTCATCC | TCTGCGGGTGAGTGGTAGTA |
| ATF4 | CCCTTCACCTTCTTACAACCTC | TTCACTGCCCAGCTCTAAACTA |
| NRAS | AAACCTCAGCCAAGACCAGA | AACCCTGAGTCCCATCATCAC |
| PIK3CB | CTGCGACAGATGAGTGATGAAG | CCCTATCCTCCGATTACCAAG |
| STAT1 | CACCTACGAACATGACCCTATCA | GCTGTCTTTCCACCACAAACG |
|  |  |  |
