# Supplementary material for: Inhibition of CDKL3 downregulates STAT1 thus suppressing prostate cancer development
Source: Cell Death Dis. 2023 Mar 10;14(3):189. doi: 10.1038/s41419-023-05694-3 (PMC10006411; doi:10.1038/s41419-023-05694-3)
Supplement: Supplementary file 4 — Table S4 [file 41419_2023_5694_MOESM4_ESM.docx]

Table S4 Relationship between CDKL3 expression and tumor characteristics in patients with prostate cancer analyzed by Spearman rank correlation analysis

| Tumor characteristics | index |  |
| --- | --- | --- |
| Stage | Spearman correlation | 0.208 |
|  | Significance (two tailed) | 0.027 |
|  | n | 113 |
| Gleason Score | Spearman correlation | 0.175 |
|  | Significance (two tailed) | 0.033 |
|  | n | 149 |
