# Supplementary material for: Inhibition of CDKL3 downregulates STAT1 thus suppressing prostate cancer development
Source: Cell Death Dis. 2023 Mar 10;14(3):189. doi: 10.1038/s41419-023-05694-3 (PMC10006411; doi:10.1038/s41419-023-05694-3)
Supplement: Supplementary file 5 — Table S5 [file 41419_2023_5694_MOESM5_ESM.docx]

Table S5 Expression patterns of STAT1 in prostate cancer tissues and normal tissues revealed in immunohistochemistry analysis

| CDKL3 expression | Tumor tissue | | Normal tissue | |
| --- | --- | --- | --- | --- |
|  | Cases | Percentage | Cases | Percentage |
| Low | 23 | 59.0% | 3 | 100.0% |
| High | 16 | 41.0% | 0 | - |

*P* < 0.001
