# Supplementary material for: Inhibition of CDKL3 downregulates STAT1 thus suppressing prostate cancer development
Source: Cell Death Dis. 2023 Mar 10;14(3):189. doi: 10.1038/s41419-023-05694-3 (PMC10006411; doi:10.1038/s41419-023-05694-3)
Supplement: Supplementary file 6 — Table S6 [file 41419_2023_5694_MOESM6_ESM.docx]

Table S6 Relationship between STAT1 expression and tumor characteristics in patients with prostate cancer

| Features | No. of patients | CDKL3 expression | | *P* value |
| --- | --- | --- | --- | --- |
|  |  | low | high |  |
| All patients | 39 | 23 | 16 |  |
| Age (years) |  |  |  | 0.609 |
| ≤ 67 | 20 | 11 | 9 |  |
| > 67 | 19 | 12 | 7 |  |
| Grade |  |  |  | 0.681 |
| 2 | 33 | 19 | 14 |  |
| 3 | 6 | 4 | 2 |  |
| Tumor size |  |  |  | 0.006 |
| ≤ 2 | 11 | 10 | 1 |  |
| > 2 | 5 | 1 | 4 |  |
|  |  |  |  |  |
