# Supplementary material for: Inhibition of CDKL3 downregulates STAT1 thus suppressing prostate cancer development
Source: Cell Death Dis. 2023 Mar 10;14(3):189. doi: 10.1038/s41419-023-05694-3 (PMC10006411; doi:10.1038/s41419-023-05694-3)
Supplement: Supplementary file 7 — Table S7 [file 41419_2023_5694_MOESM7_ESM.docx]

Table S7 Relationship between STAT1 expression and tumor characteristics in patients with prostate cancer analyzed by Spearman rank correlation analysis

| Tumor characteristics | index |  |
| --- | --- | --- |
| Tumor size | Spearman correlation | 0.709 |
|  | Significance (two tailed) | 0.002 |
|  | n | 16 |
