# Supplementary material for: Inhibition of CDKL3 downregulates STAT1 thus suppressing prostate cancer development
Source: Cell Death Dis. 2023 Mar 10;14(3):189. doi: 10.1038/s41419-023-05694-3 (PMC10006411; doi:10.1038/s41419-023-05694-3)
Supplement: Supplementary file 8 — Supplementary figure legends [file 41419_2023_5694_MOESM8_ESM.docx]

**Figure S1.** The infection efficiencies of shCtrl and shCDKL3 lentiviruses in DU 145 and PC-3 cells were evaluated by fluorescence imaging.

**Figure S2.** A human apoptosis antibody array was used to detect the effects of CDKL3 knockdown on the expression levels of apoptosis-related proteins. The original and simulated diagram of the antibody arrays was shown.

**Figure S3.** The expression of Bcl-2, HSP27 and Caspase-3 was detected in shCtrl and shCDKL3 groups of PC-3 cells.

**Figure S4.** (A) The mRNA and protein levels of CDKL3 in shCtrl and shCDKL3 groups of LNcap cells were detected by qPCR and western blot, respectively. (B) The differential cell apoptosis of shCtrl and shCDKL3 groups of LNcap cells was analyzed by flow cytometry. (C) The expression of Bcl-2, HSP27 and Caspase-3 was detected in shCtrl and shCDKL3 groups of LNcap cells. Data were presented as mean with standard deviation. ** P < 0.01, *** P < 0.001

**Figure S5.** The expression of CDKL3 was detected in xenografts formed by shCtrl and shCDKL3 groups of DU 145 and PC-3 cells.

**Figure S6.** (A) A volcano plot of gene expression profiles in DU 145 cells with or without CDKL3 knockdown. The green dots represent the downregulated DEGs. The red dots represent the upregulated DEGs. (B) The enrichment of the DEGs in canonical signaling pathways was analyzed by IPA. (C) The enrichment of the DEGs in IPA disease and function was analyzed by IPA.

**Figure S7.** (A) The expression of STAT1, ERK and p-ERK was detected in shCtrl, shCDKL3 and ERK activator treated shCDKL3 PC-3 cells. (E) CCK8 assay was performed to evaluate the proliferation rate of indicated groups of PC-3 cells. (F) Flow cytometry was applied to assess the apoptosis of indicated groups of PC-3 cells.

**Figure S8.** (A) Ubibrowser database was used to predict the upstream E3 ligase of STAT1. (B) A co-IP assay was performed using indicated antibodies followed by western blot analysis with anti-CBL and anti-CDKL3 antibodies for demonstrating the protein-protein interaction between CDKL3 and CBL.

**Figure S9.** The knockdown efficiencies of shSTAT1-1, shSTAT1-2, shSTAT1-3 were detected by qPCR.

**Figure S10.** (A) The mRNA expression of CDKL3 and STAT1 in DU 145 cells with indicated treatment was detected by qPCR. (B The protein level of CDKL3 and STAT1 in DU 145 cells with indicated treatment was detected by WB. Data were presented as mean with standard deviation. *** P < 0.001

**Figure S11.** (A) The mRNA expression of CDKL3 and STAT1 in PC-3 cells with indicated treatment was detected by qPCR. (B) The protein level of CDKL3 and STAT1 in PC-3 cells with indicated treatment was detected by WB. Data were presented as mean with standard deviation. *** P < 0.001

**Figure S12.** (A) The cell proliferation rate of PC-3 cells with mere CDKL3 overexpression, mere STAT1 knockdown, or both, was evaluated by MTT assay. (B) The colony formation ability of PC-3 cells with mere CDKL3 overexpression, mere STAT1 knockdown, or both, was assessed by colony formation assay. (C) The percentage of apoptotic in PC-3 cells with mere CDKL3 overexpression, mere STAT1 knockdown, or both, was detected by flow cytometry. Data were presented as mean with standard deviation. * P < 0.05, *** P < 0.001
