# Supplementary figures and images for: Inhibition of CDKL3 downregulates STAT1 thus suppressing prostate cancer development
Source: Cell Death Dis. 2023 Mar 10;14(3):189. doi: 10.1038/s41419-023-05694-3 (PMC10006411; doi:10.1038/s41419-023-05694-3)

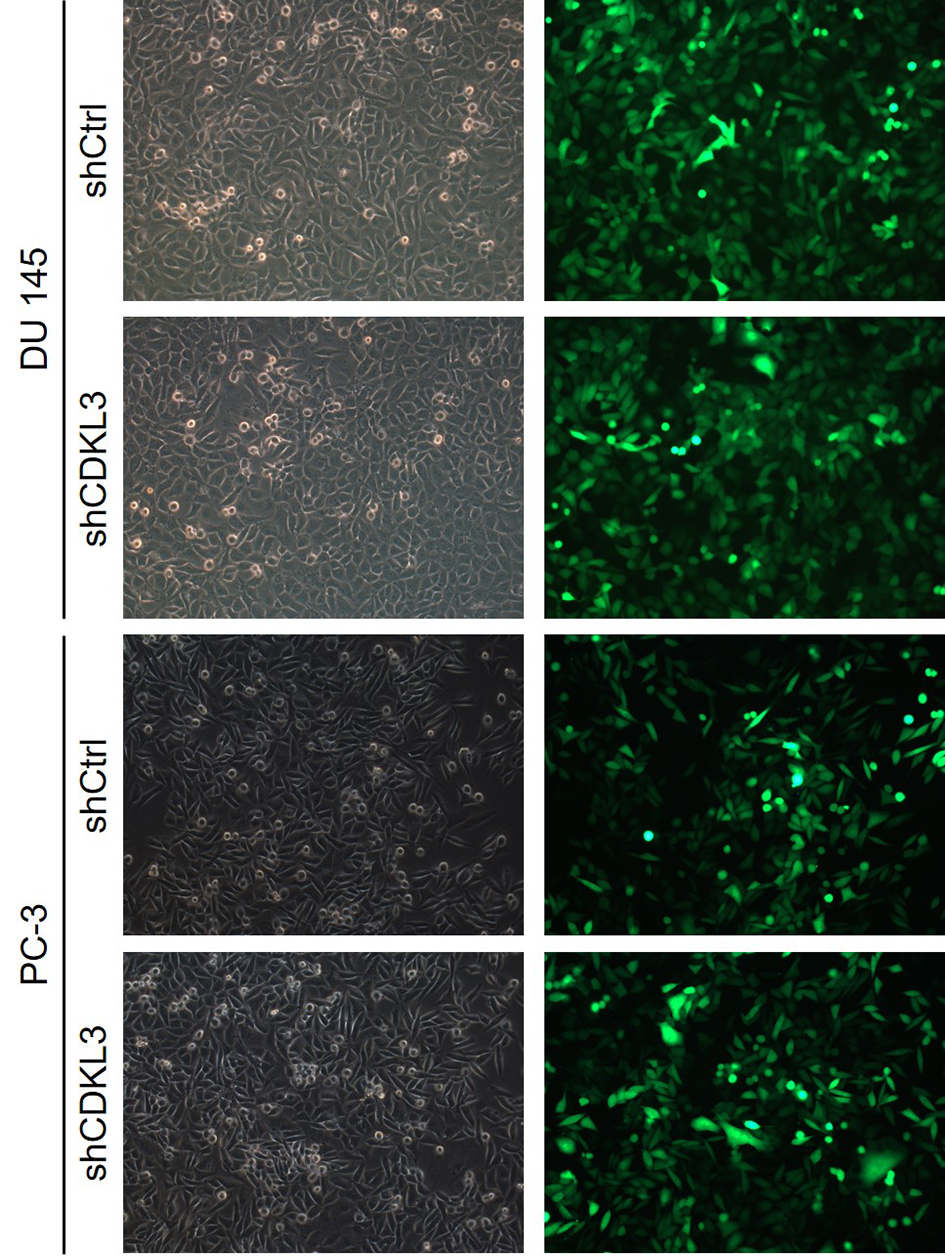

Supplement: Supplementary file 9 — Figure S1 [file 41419_2023_5694_MOESM9_ESM.tif]

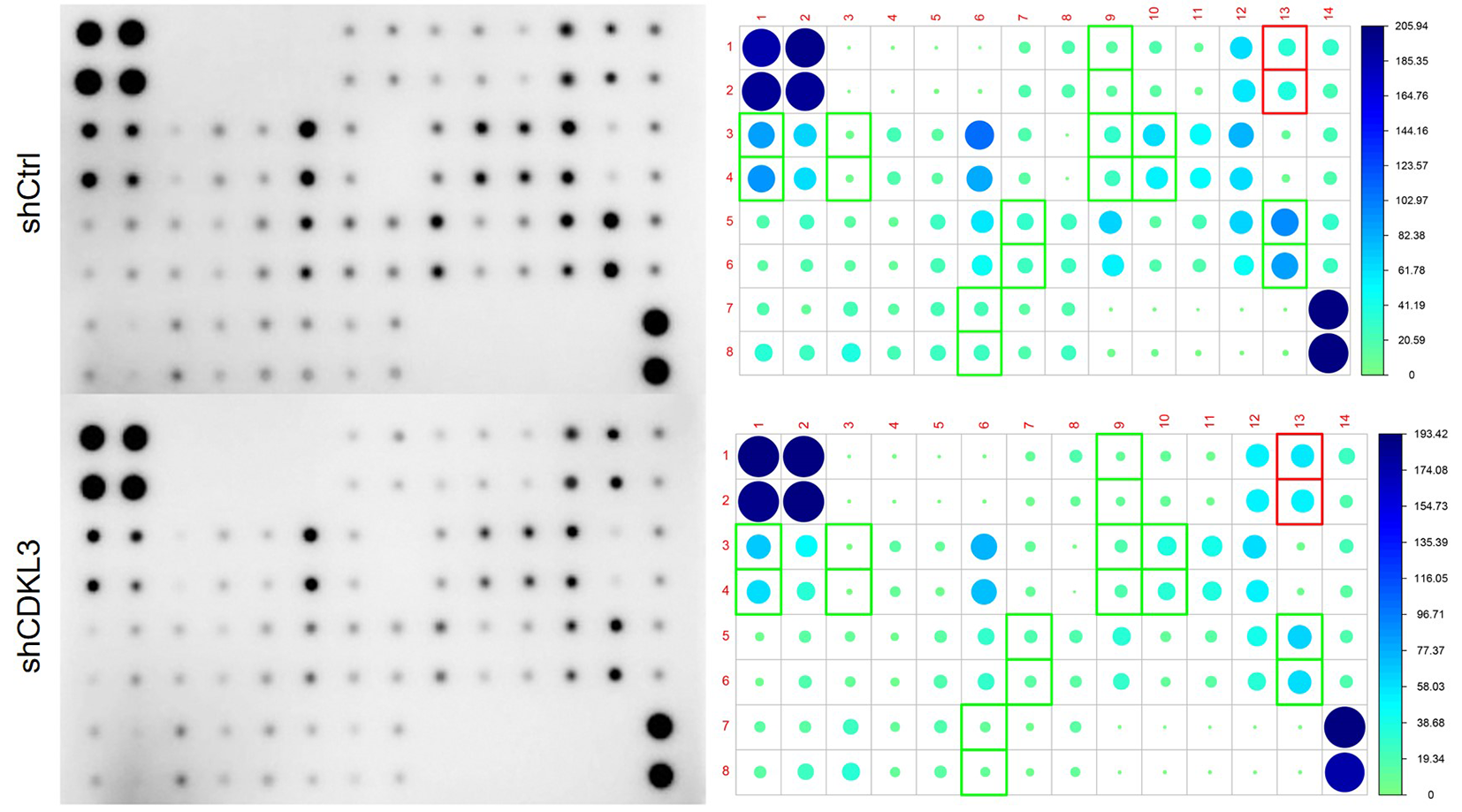

Supplement: Supplementary file 10 — Figure S2 [file 41419_2023_5694_MOESM10_ESM.tif]

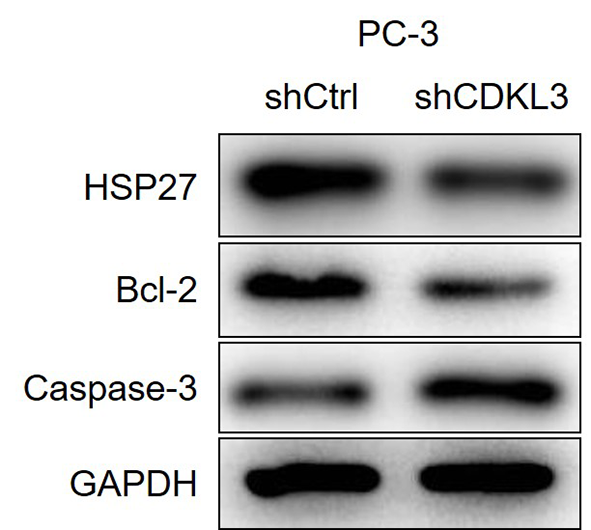

Supplement: Supplementary file 11 — Figure S3 [file 41419_2023_5694_MOESM11_ESM.tif]

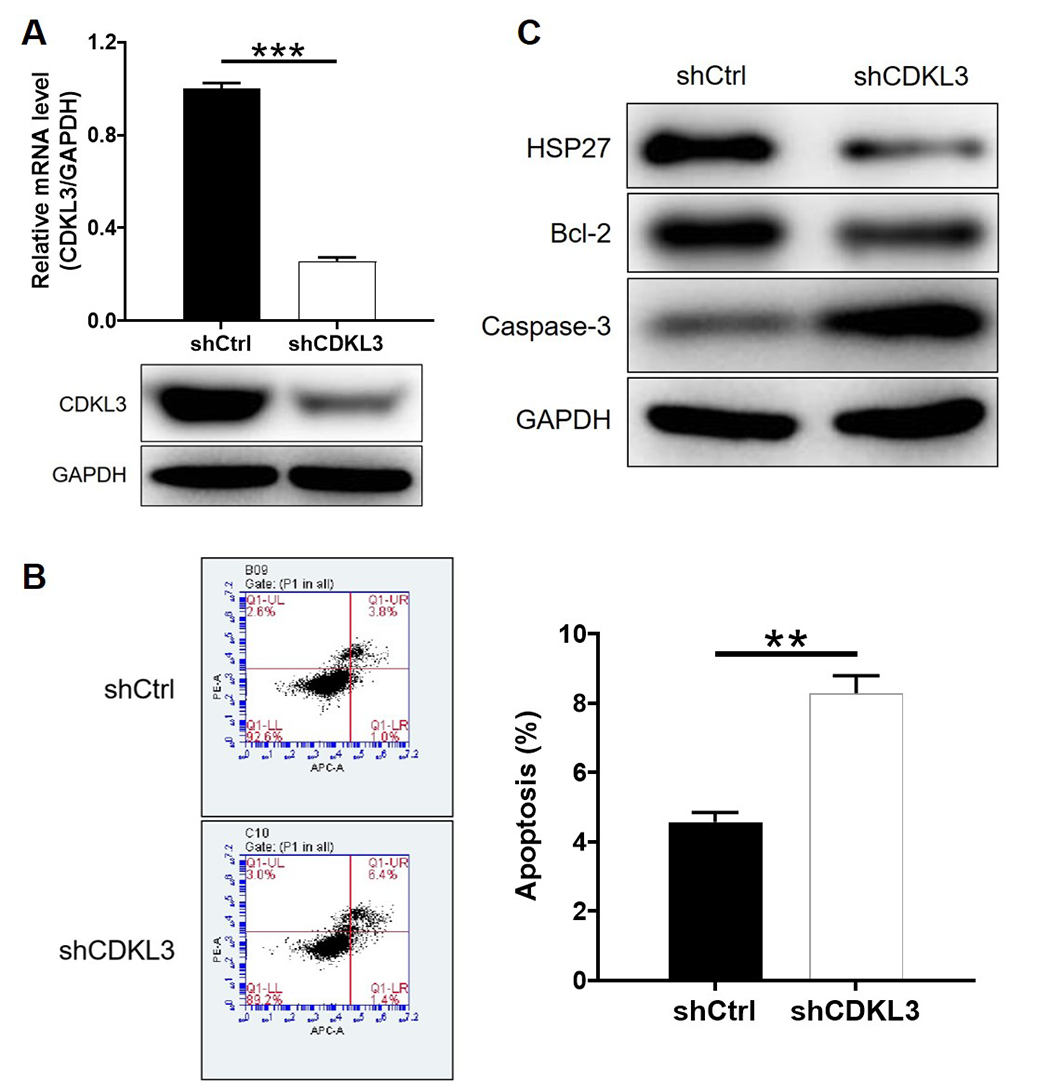

Supplement: Supplementary file 12 — Figure S4 [file 41419_2023_5694_MOESM12_ESM.tif]

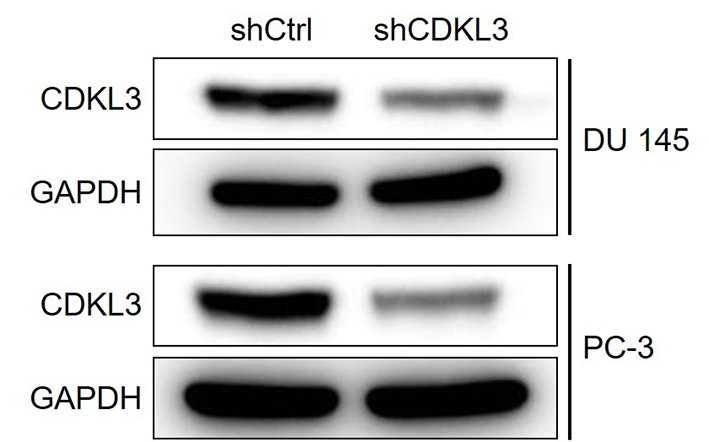

Supplement: Supplementary file 13 — Figure S5 [file 41419_2023_5694_MOESM13_ESM.tif]

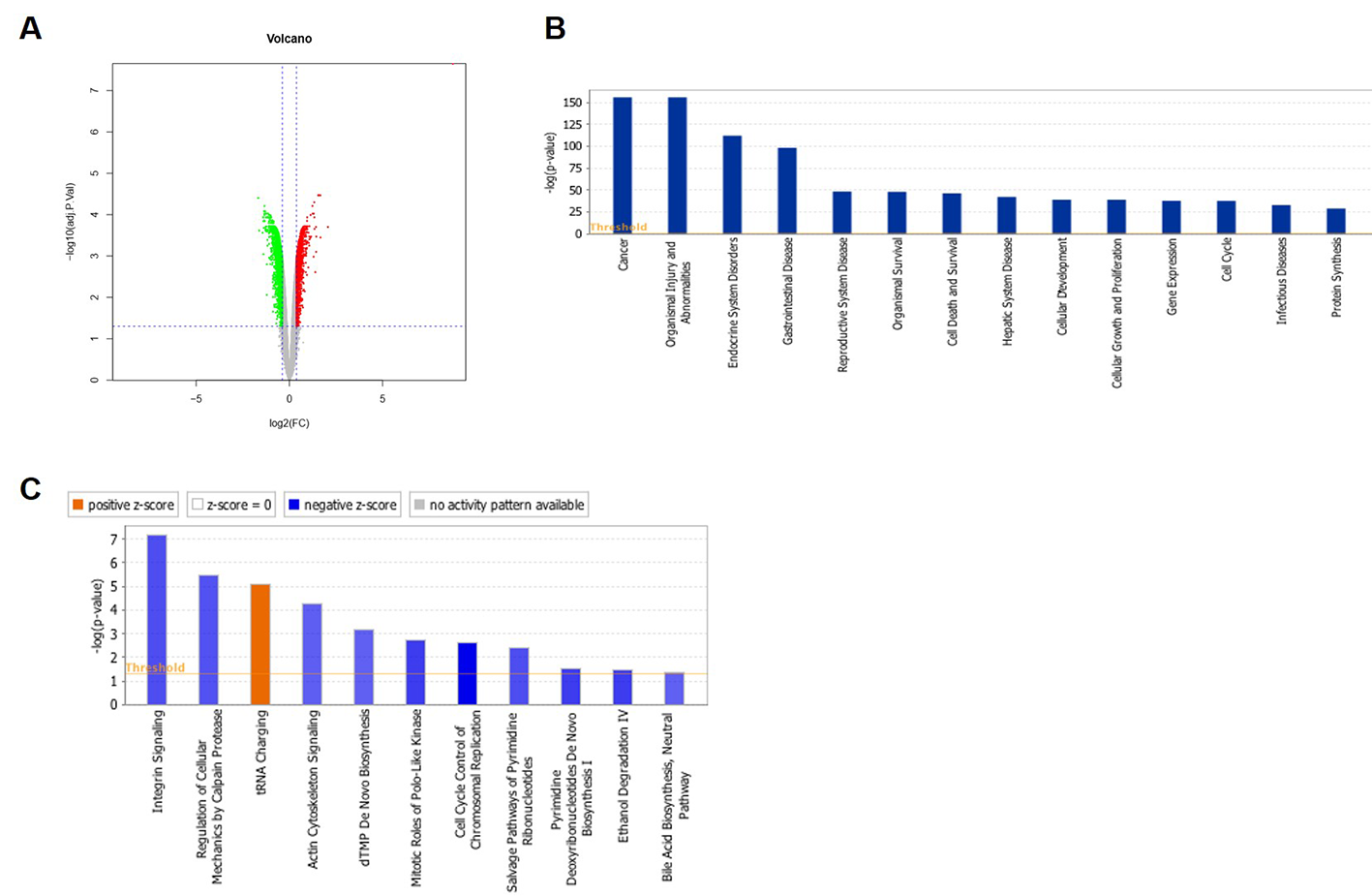

Supplement: Supplementary file 14 — Figure S6 [file 41419_2023_5694_MOESM14_ESM.tif]

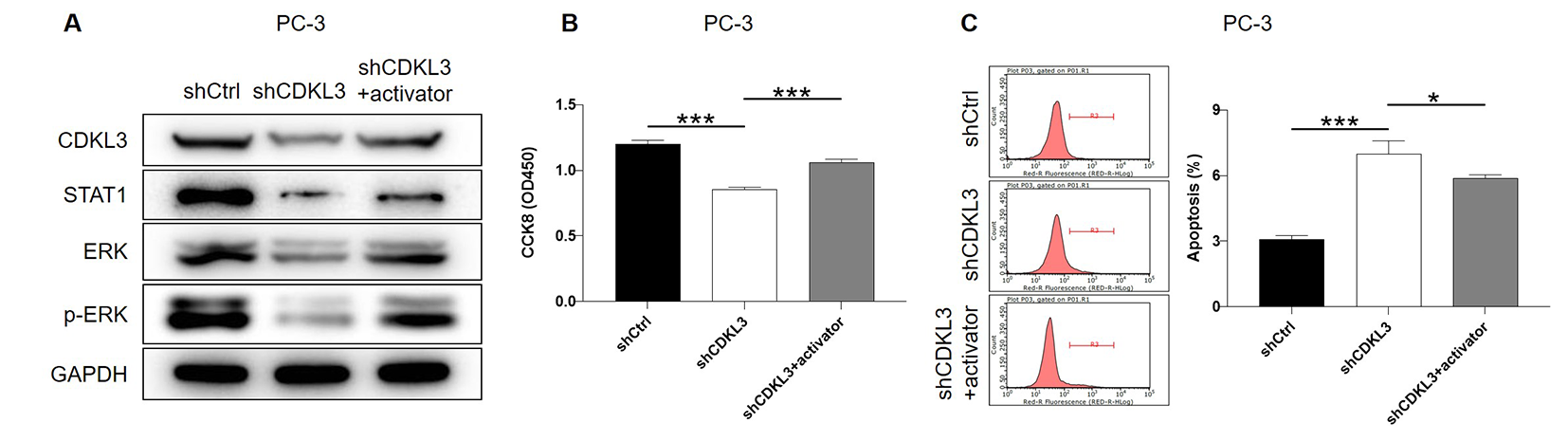

Supplement: Supplementary file 15 — Figure S7 [file 41419_2023_5694_MOESM15_ESM.tif]

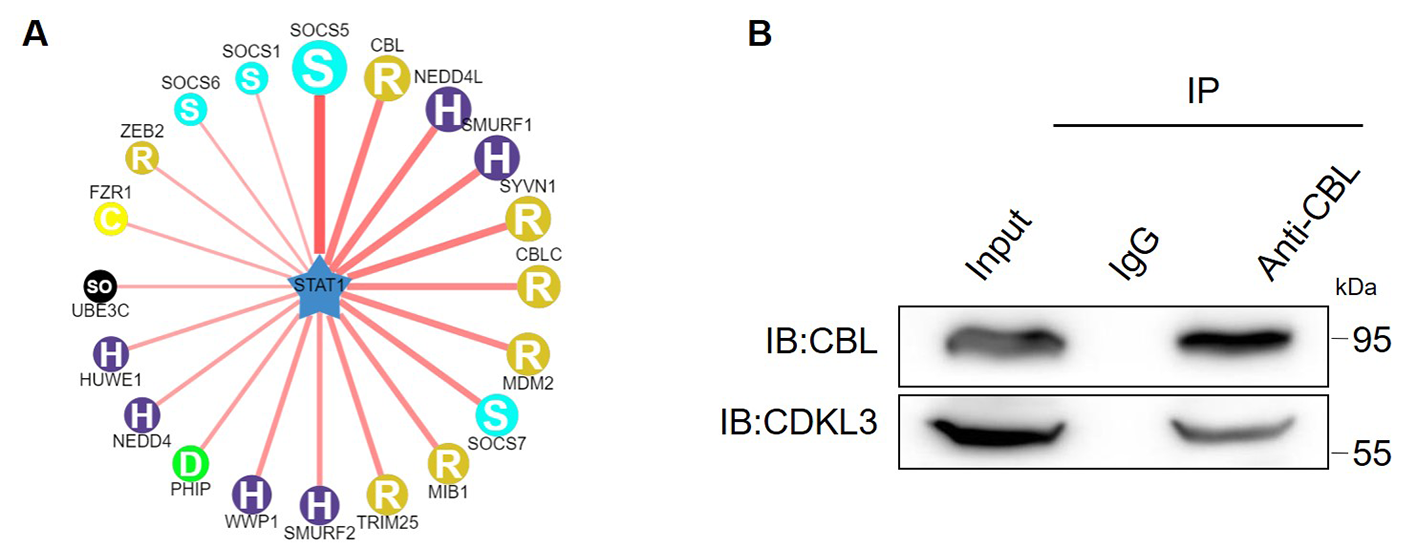

Supplement: Supplementary file 16 — Figure S8 [file 41419_2023_5694_MOESM16_ESM.tif]

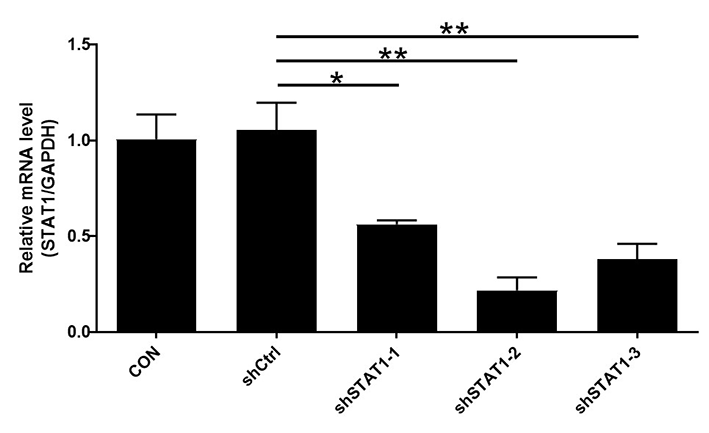

Supplement: Supplementary file 17 — Figure S9 [file 41419_2023_5694_MOESM17_ESM.tif]

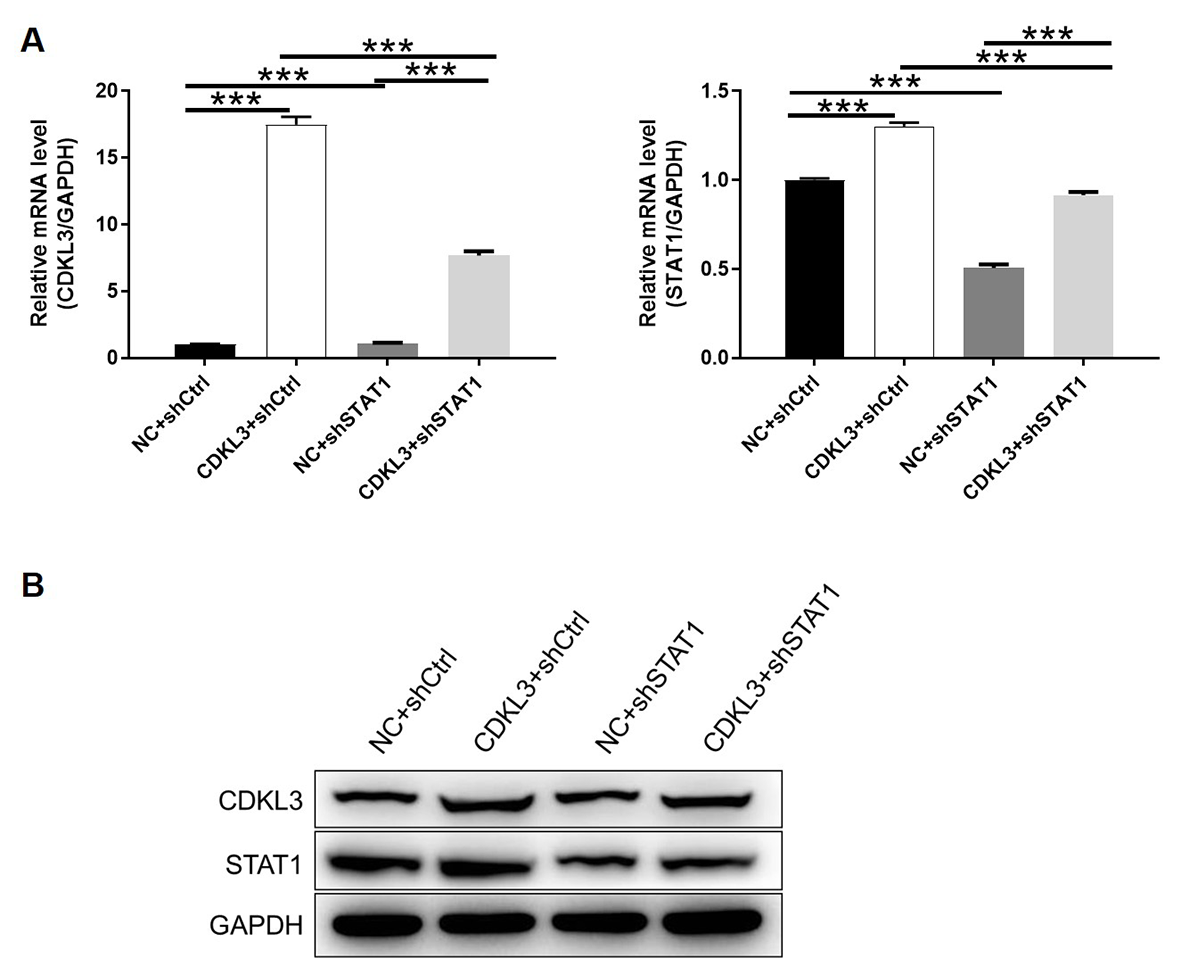

Supplement: Supplementary file 18 — Figure S10 [file 41419_2023_5694_MOESM18_ESM.tif]

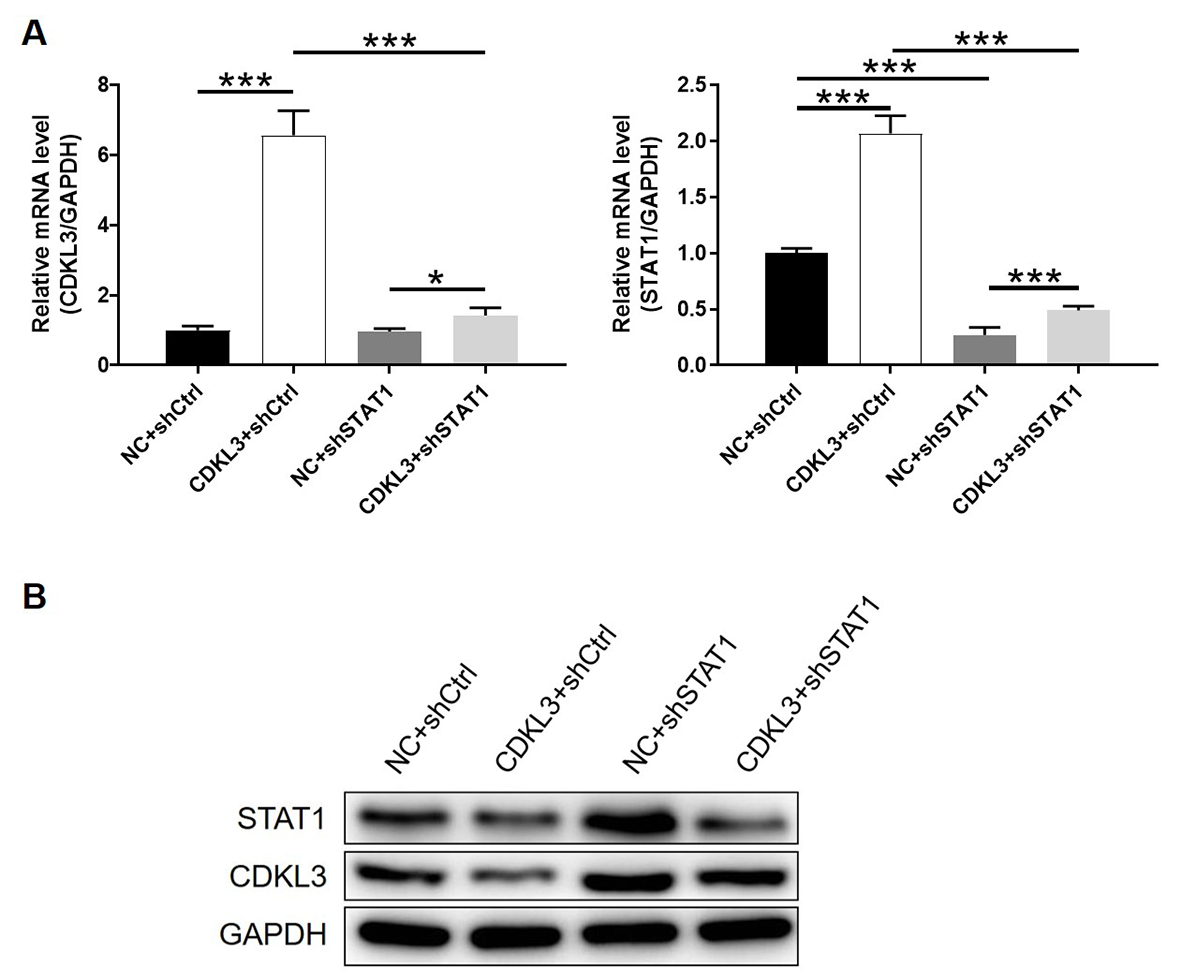

Supplement: Supplementary file 19 — Figure S11 [file 41419_2023_5694_MOESM19_ESM.tif]

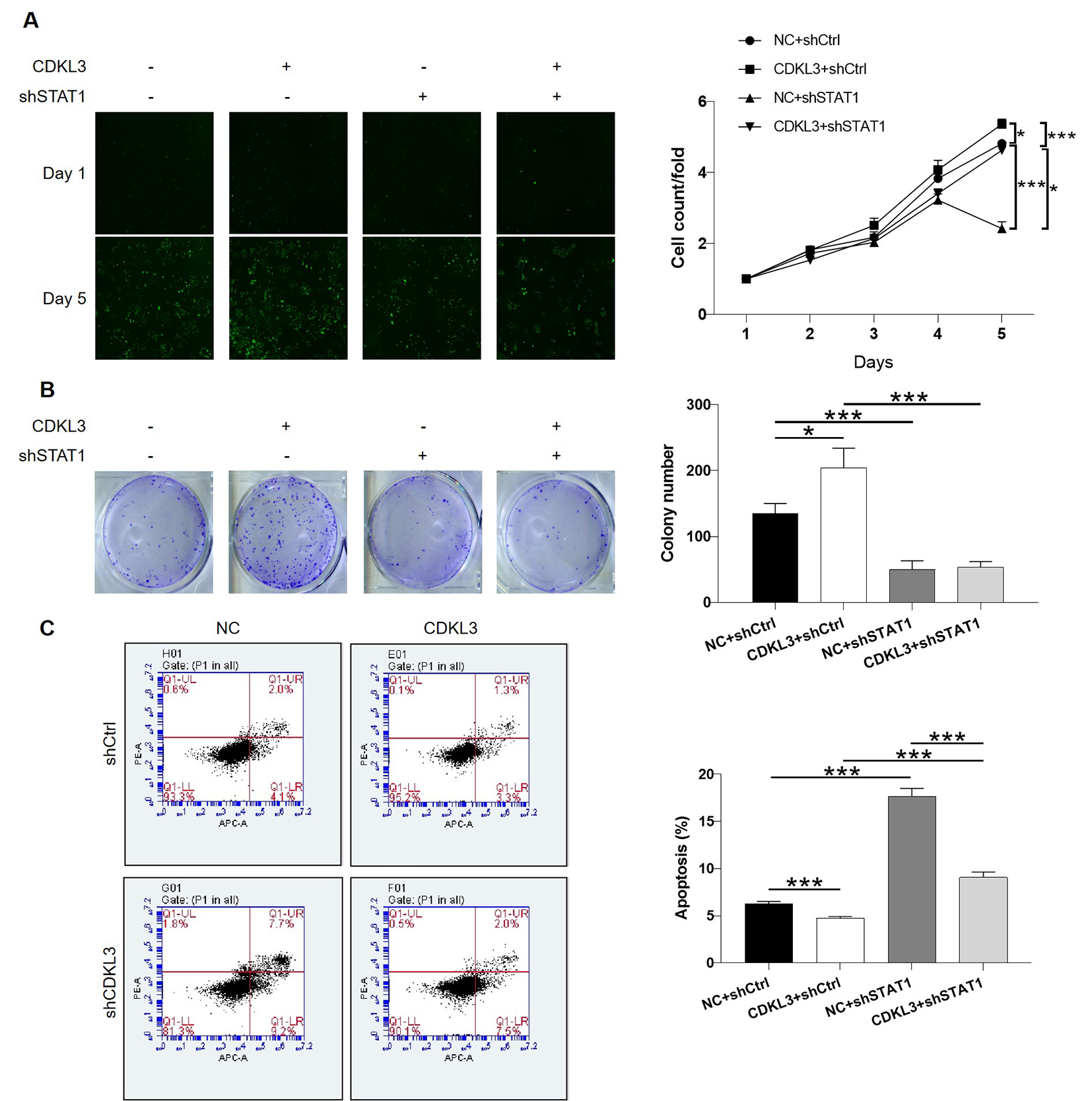

Supplement: Supplementary file 20 — Figure S12 [file 41419_2023_5694_MOESM20_ESM.tif]
