# Supplementary material for: Inhibition of CDKL3 downregulates STAT1 thus suppressing prostate cancer development
Source: Cell Death Dis. 2023 Mar 10;14(3):189. doi: 10.1038/s41419-023-05694-3 (PMC10006411; doi:10.1038/s41419-023-05694-3)
Supplement: Supplementary file 21 — Original Data File [file 41419_2023_5694_MOESM21_ESM.pdf]

Figure 1B

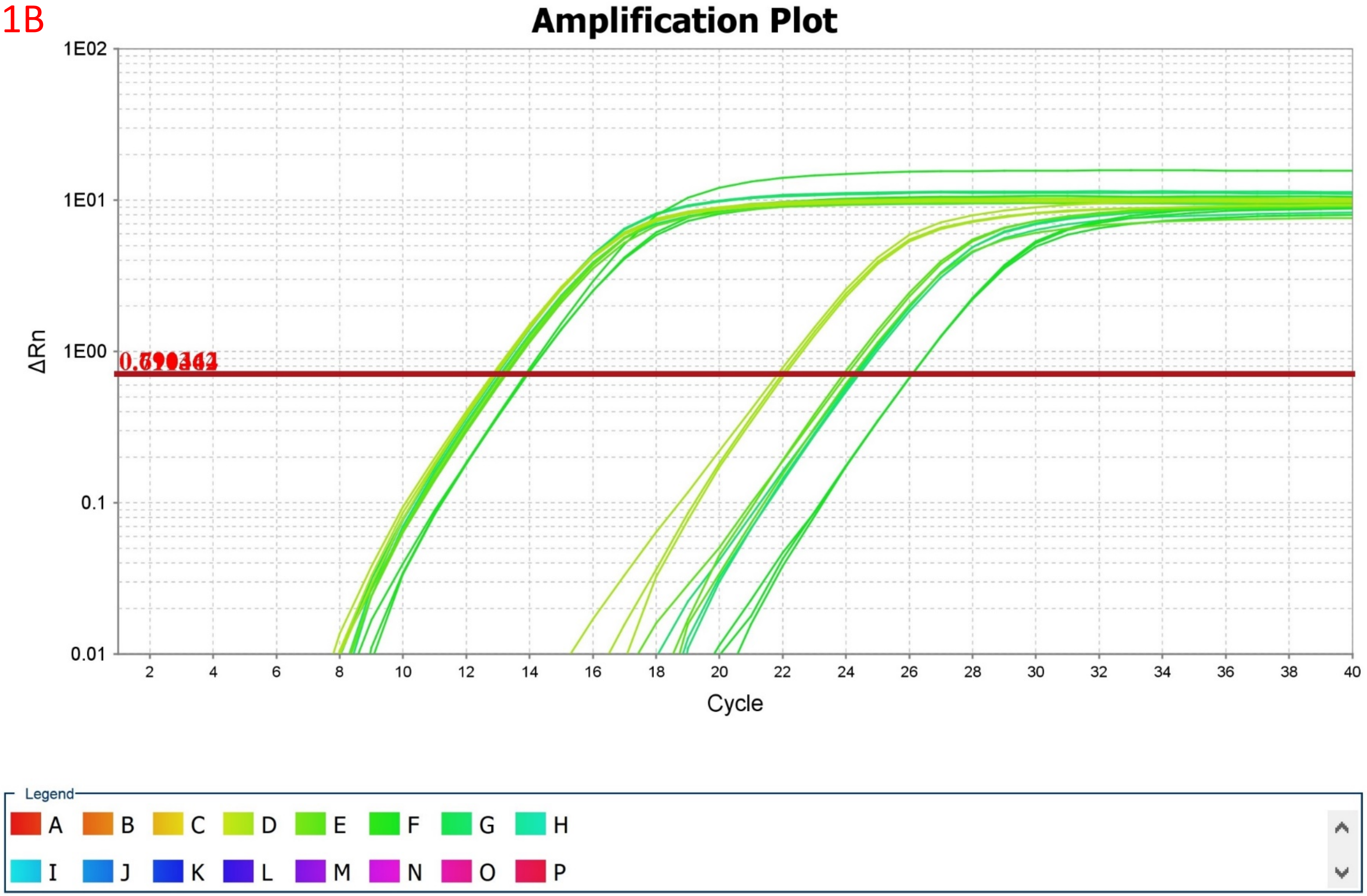

Amplification Plot
1E02
1E01
1E00
0.1
0.01
ΔRn
2
4
6
8
10
12
14
16
18
20
22
24
26
28
30
32
34
36
38
40
Cycle
Legend
A
B
C
D
E
F
G
H
I
J
K
L
M
N
O
P

Figure 1B

Block Type	384-Well Block
Calibration B	Yes
Calibration B	2020-08-14 10:25:44 AM CST
Calibration F	Yes
Calibration F	2020-08-14 11:18:18 AM CST
Calibration N	Yes
Calibration N	2020-08-14 10:47:55 AM CST
Calibration F	Yes
Calibration F	2020-08-14 10:19:00 AM CST
Calibration F	Yes
Calibration F	2020-08-14 11:03:25 AM CST
Calibration S	Yes
Calibration S	2020-08-14 11:10:52 AM CST
Calibration T	Yes
Calibration T	2020-08-14 10:55:44 AM CST
Calibration U	Yes
Calibration U	2020-08-14 10:33:02 AM CST
Calibration V	Yes
Calibration V	2020-08-14 10:40:30 AM CST
Chemistry	SYBR_GREEN
Experiment Barcode	
Experiment Comments	
Experiment	C:\Users\gonghq\Desktop\2021-07-13CDJ.eds
Experiment	2021-07-13CDJ
Experiment	2021-07-13 23:45:50 PM CST
Experiment	Comparative Cт (ΔΔCт)
Experiment User Name	
Instrument N	278882256
Instrument S	278882256
Instrument T	ViiA 7
Passive Ref	ROX
Quantificatio	Ct
Signal Smo	true
Stage/ Cycl	Stage 2, Step 2

Well	Well Positio	Omit	Sample Name	Target Name	Task	Reporter	Quencher	Quantity	Quantity Me	Quantity SD	RQ	RQ Min	RQ Max	CT	Ct Mean	Ct SD
91	D19	FALSE	WPMY	H-GAPDH	UNKNOWN	SYBR	None							13.866158		
92	D20	FALSE	WPMY	H-GAPDH	UNKNOWN	SYBR	None							13.900924		
93	D21	FALSE	WPMY	H-GAPDH	UNKNOWN	SYBR	None							13.920371		
94	D22	FALSE	WPMY	H-CDKL3	UNKNOWN	SYBR	None							26.093311		
95	D23	FALSE	WPMY	H-CDKL3	UNKNOWN	SYBR	None							26.0872		
96	D24	FALSE	WPMY	H-CDKL3	UNKNOWN	SYBR	None							26.075817		
115	E19	FALSE	DU 145	H-GAPDH	UNKNOWN	SYBR	None							13.168142		
116	E20	FALSE	DU 145	H-GAPDH	UNKNOWN	SYBR	None							13.079791		
117	E21	FALSE	DU 145	H-GAPDH	UNKNOWN	SYBR	None							13.227659		
118	E22	FALSE	DU 145	H-CDKL3	UNKNOWN	SYBR	None							24.033661		
119	E23	FALSE	DU 145	H-CDKL3	UNKNOWN	SYBR	None							23.932343		
120	E24	FALSE	DU 145	H-CDKL3	UNKNOWN	SYBR	None							24.215359		
139	F19	FALSE	PC-3	H-GAPDH	UNKNOWN	SYBR	None							12.799695		
140	F20	FALSE	PC-3	H-GAPDH	UNKNOWN	SYBR	None							12.850623		
141	F21	FALSE	PC-3	H-GAPDH	UNKNOWN	SYBR	None							12.898605		
142	F22	FALSE	PC-3	H-CDKL3	UNKNOWN	SYBR	None							21.860949		
143	F23	FALSE	PC-3	H-CDKL3	UNKNOWN	SYBR	None							22.068514		
144	F24	FALSE	PC-3	H-CDKL3	UNKNOWN	SYBR	None							21.984205		
163	G19	FALSE	LNcap	H-GAPDH	UNKNOWN	SYBR	None							12.915323		
164	G20	FALSE	LNcap	H-GAPDH	UNKNOWN	SYBR	None							12.940732		
165	G21	FALSE	LNcap	H-GAPDH	UNKNOWN	SYBR	None							13.026218		
166	G22	FALSE	LNcap	H-CDKL3	UNKNOWN	SYBR	None							24.292953		
167	G23	FALSE	LNcap	H-CDKL3	UNKNOWN	SYBR	None							24.352571		
168	G24	FALSE	LNcap	H-CDKL3	UNKNOWN	SYBR	None							24.415634		

Analysis Ty	Singleplex
Endogenous	H-SLMO2
RQ Min/Max	95.0
Reference S	Sample 1

Figure 1B

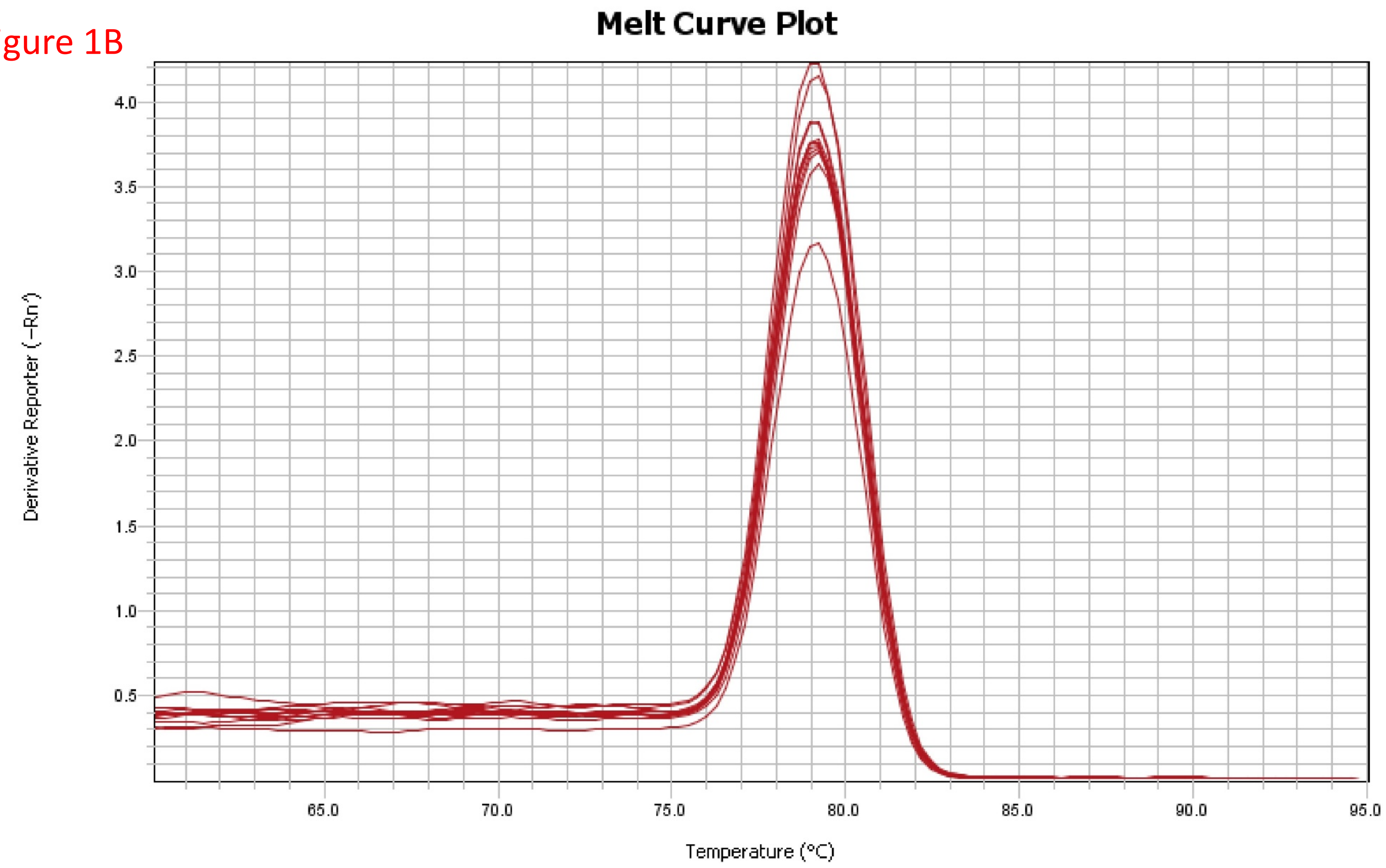
Melt Curve Plot
Derivative Reporter (−Rn')
4.0
3.5
3.0
2.5
2.0
1.5
1.0
0.5
65.0
70.0
75.0
80.0
85.0
90.0
95.0
Temperature (°C)

Figure 1B

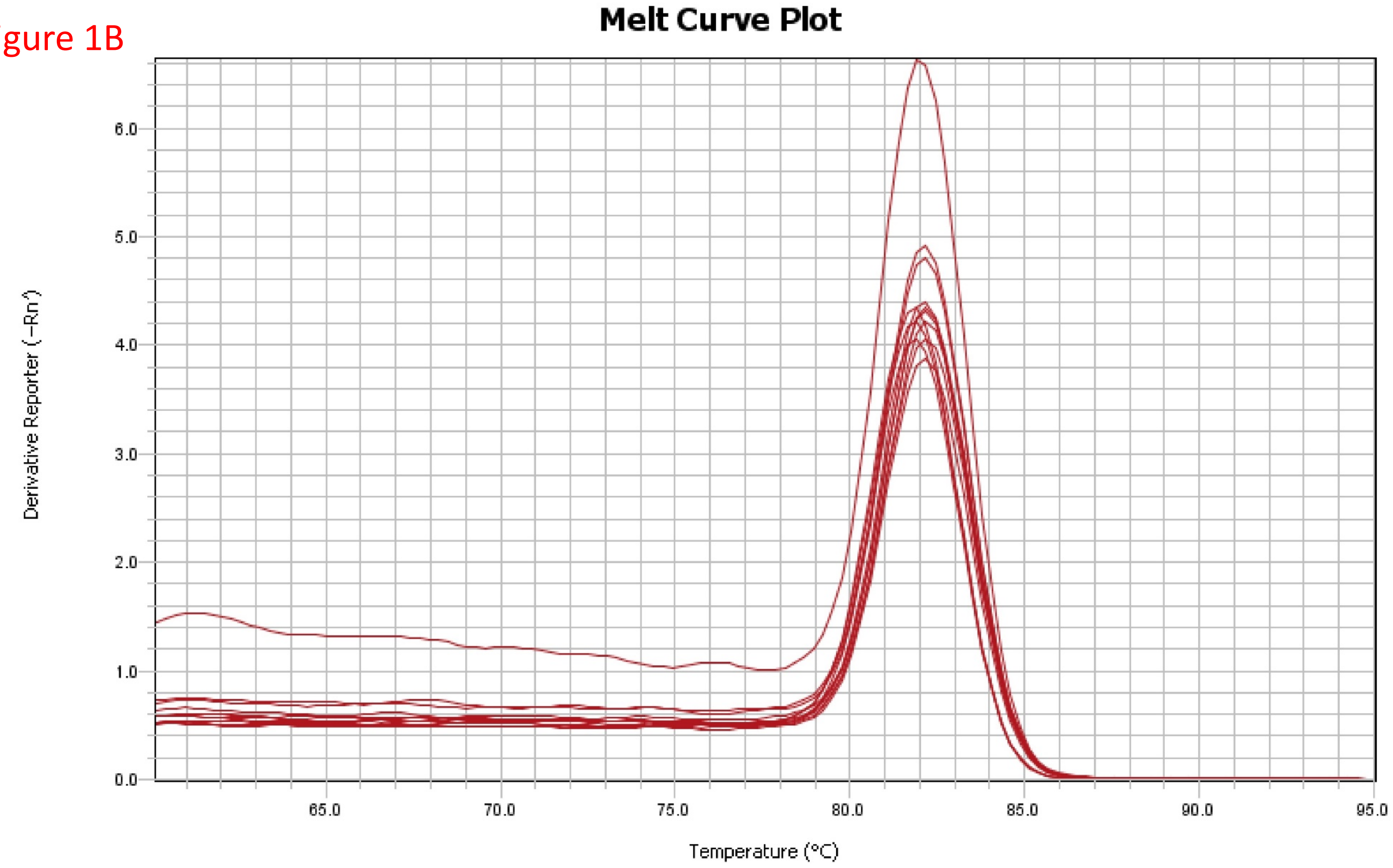
Melt Curve Plot
Derivative Reporter (−Rn')
6.0
5.0
4.0
3.0
2.0
1.0
0.0
65.0
70.0
75.0
80.0
85.0
90.0
95.0
Temperature (°C)

Figure 1C

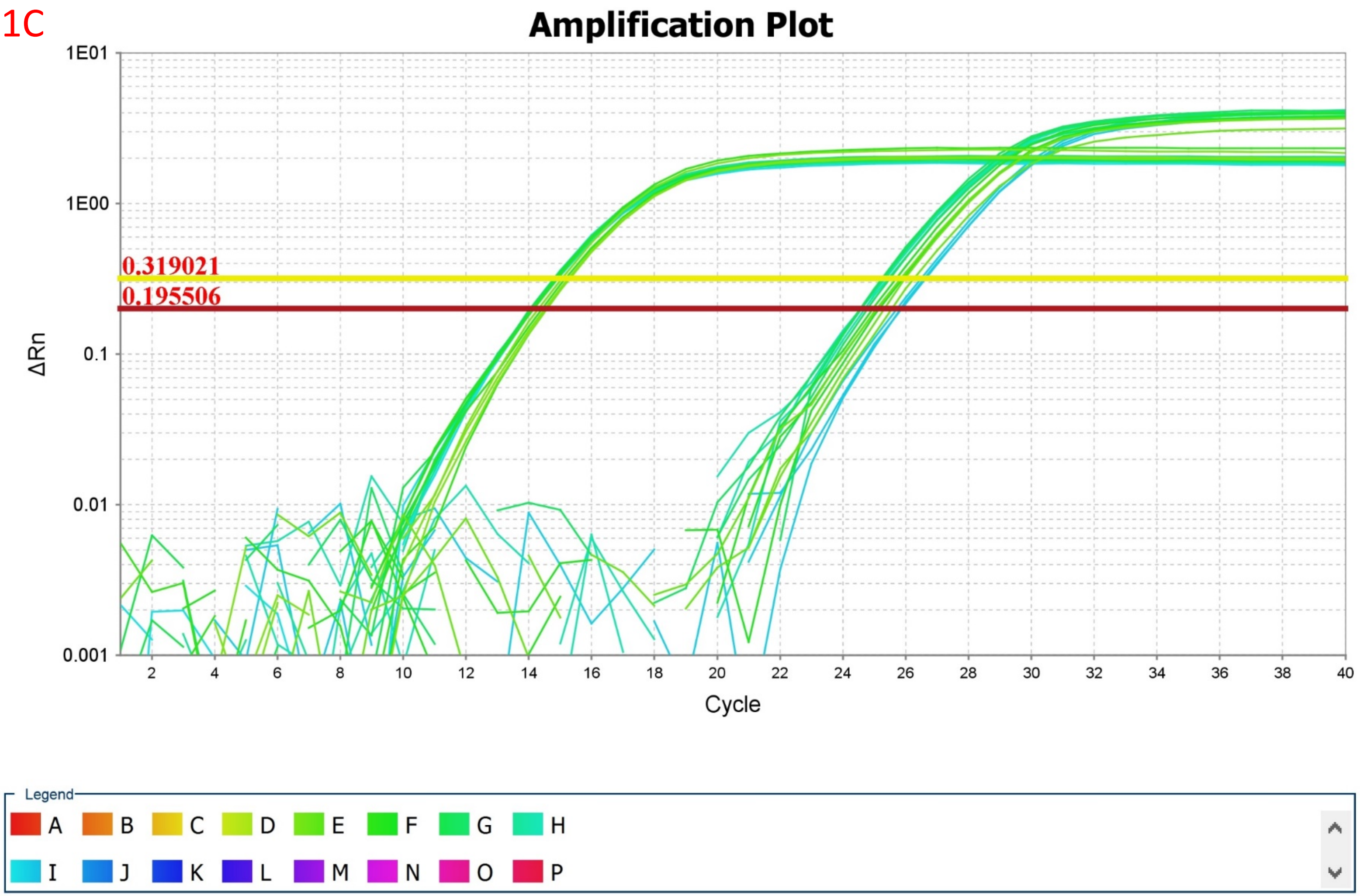
Amplification Plot
1E01
1E00
0.1
0.01
0.001
ΔRn
0.319021
0.195506
2
4
6
8
10
12
14
16
18
20
22
24
26
28
30
32
34
36
38
40
Cycle
Legend
A
B
C
D
E
F
G
H
I
J
K
L
M
N
O
P

Figure 1C

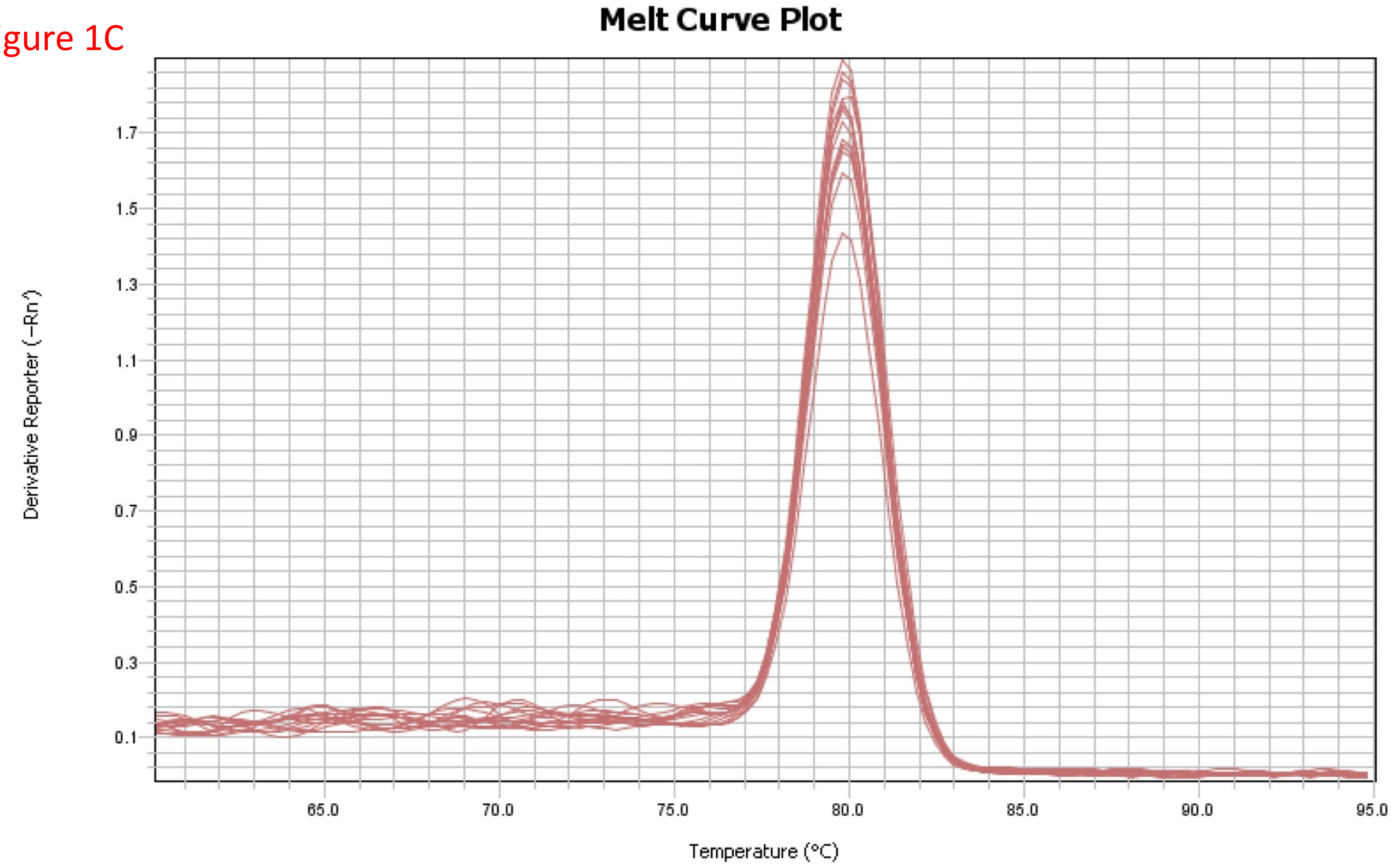
Melt Curve Plot
1.7
1.5
1.3
1.1
0.9
0.7
0.5
0.3
0.1
Derivative Reporter (−Rn')
65.0
70.0
75.0
80.0
85.0
90.0
95.0
Temperature (°C)

Figure 1C

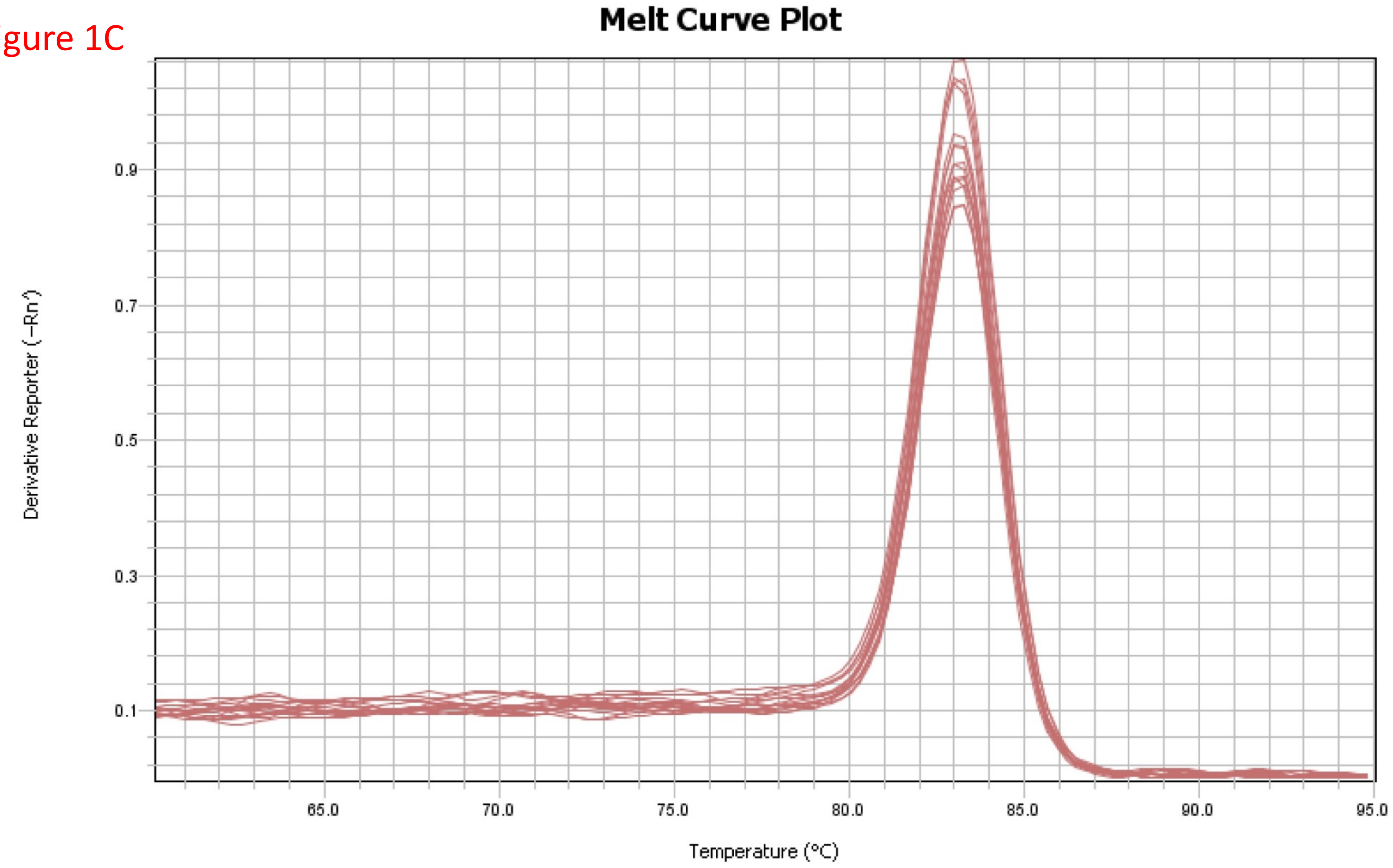
Melt Curve Plot
Derivative Reporter (−Rn')
0.9
0.7
0.5
0.3
0.1
65.0
70.0
75.0
80.0
85.0
90.0
95.0
Temperature (°C)

Figure 1C

Block Typ	384-Well Block																
Calibratior	Yes																
Calibratior	2016-01-20 10:12:38 AM CST																
Calibratior	Yes																
Calibratior	2016-01-20 10:28:26 AM CST																
Calibratior	Yes																
Calibratior	2016-01-20 10:44:55 AM CST																
Calibratior	Yes																
Calibratior	2016-01-20 10:05:17 AM CST																
Calibratior	Yes																
Calibratior	2016-01-20 11:25:36 AM CST																
Calibratior	Yes																
Calibratior	2016-01-20 10:35:50 AM CST																
Calibratior	Yes																
Calibratior	2016-01-20 11:02:33 AM CST																
Calibratior	Yes																
Calibratior	2016-01-20 10:20:14 AM CST																
Calibratior	Yes																
Calibratior	2016-01-20 10:54:46 AM CST																
Chemistry	SYBR_GREEN																
Experiment Barcode																	
Experiment Comments																	
Experimer	C:\Users\Administrator\Desktop\1\2019-05-29 L1.eds																
Experimer	2019-05-31 S1																
Experimer	2019-05-31 20:30:32 PM CST																
Experimer	Comparative C_T ($\Delta\Delta C_T$)																
Experiment User Name																	
Instrumen	278882256																
Instrumen	278882256																
Instrumen	ViiA 7																
Passive R	ROX																
Quantifica	Ct																
Signal Sm	true																
Stage/ Cy	Stage 2, Step 2																
Well	Well Posit	Omit	Sample Name	Target Name	Task	Reporter	Quencher	Quantity	Quantity M	Quantity S	RQ	RQ Min	RQ Max	CT	Ct Mean	Ct SD	Delta Ct
97	E1	FALSE	DU 145 CON	H-GAPDH	UNKNOW	SYBR	None							14.268			
98	E2	FALSE	DU 145 CON	H-GAPDH	UNKNOW	SYBR	None							14.455			
99	E3	FALSE	DU 145 CON	H-GAPDH	UNKNOW	SYBR	None							14.505			
109	E13	FALSE	DU 145 CON	H-CDKL3	UNKNOW	SYBR	None							25.852			
110	E14	FALSE	DU 145 CON	H-CDKL3	UNKNOW	SYBR	None							25.989			
111	E15	FALSE	DU 145 CON	H-CDKL3	UNKNOW	SYBR	None							26.261			
121	F1	FALSE	DU 145-shCtrl	H-GAPDH	UNKNOW	SYBR	None							14.102			
122	F2	FALSE	DU 145-shCtrl	H-GAPDH	UNKNOW	SYBR	None							14.355			
123	F3	FALSE	DU 145-shCtrl	H-GAPDH	UNKNOW	SYBR	None							14.501			
133	F13	FALSE	DU 145-shCtrl	H-CDKL3	UNKNOW	SYBR	None							25.705			
134	F14	FALSE	DU 145-shCtrl	H-CDKL3	UNKNOW	SYBR	None							25.864			
135	F15	FALSE	DU 145-shCtrl	H-CDKL3	UNKNOW	SYBR	None							25.937			
145	G1	FALSE	DU 145-shCDKL3-2	H-GAPDH	UNKNOW	SYBR	None							13.986			
146	G2	FALSE	DU 145-shCDKL3-2	H-GAPDH	UNKNOW	SYBR	None							13.989			
147	G3	FALSE	DU 145-shCDKL3-2	H-GAPDH	UNKNOW	SYBR	None							14.021			
157	G13	FALSE	DU 145-shCDKL3-2	H-CDKL3	UNKNOW	SYBR	None							25.245			
158	G14	FALSE	DU 145-shCDKL3-2	H-CDKL3	UNKNOW	SYBR	None							25.262			
159	G15	FALSE	DU 145-shCDKL3-2	H-CDKL3	UNKNOW	SYBR	None							25.396			
169	H1	FALSE	DU 145-shCDKL3-3	H-GAPDH	UNKNOW	SYBR	None							13.993			
170	H2	FALSE	DU 145-shCDKL3-3	H-GAPDH	UNKNOW	SYBR	None							14.047			
171	H3	FALSE	DU 145-shCDKL3-3	H-GAPDH	UNKNOW	SYBR	None							14.067			
181	H13	FALSE	DU 145-shCDKL3-3	H-CDKL3	UNKNOW	SYBR	None							25.329			
182	H14	FALSE	DU 145-shCDKL3-3	H-CDKL3	UNKNOW	SYBR	None							25.333			
183	H15	FALSE	DU 145-shCDKL3-3	H-CDKL3	UNKNOW	SYBR	None							25.459			
193	I1	FALSE	DU 145-shCDKL3-1	H-GAPDH	UNKNOW	SYBR	None							13.888			
194	I2	FALSE	DU 145-shCDKL3-1	H-GAPDH	UNKNOW	SYBR	None							13.956			
195	I3	FALSE	DU 145-shCDKL3-1	H-GAPDH	UNKNOW	SYBR	None							13.993			
205	I13	FALSE	DU 145-shCDKL3-1	H-CDKL3	UNKNOW	SYBR	None							26.510			
206	I14	FALSE	DU 145-shCDKL3-1	H-CDKL3	UNKNOW	SYBR	None							26.620			
207	I15	FALSE	DU 145-shCDKL3-1	H-CDKL3	UNKNOW	SYBR	None							26.648			
Analysis T	Singleplex																
Endogenous Control																	
RQ Min/M	95.0																
Reference Sample																	

Figure 2A

DU 145

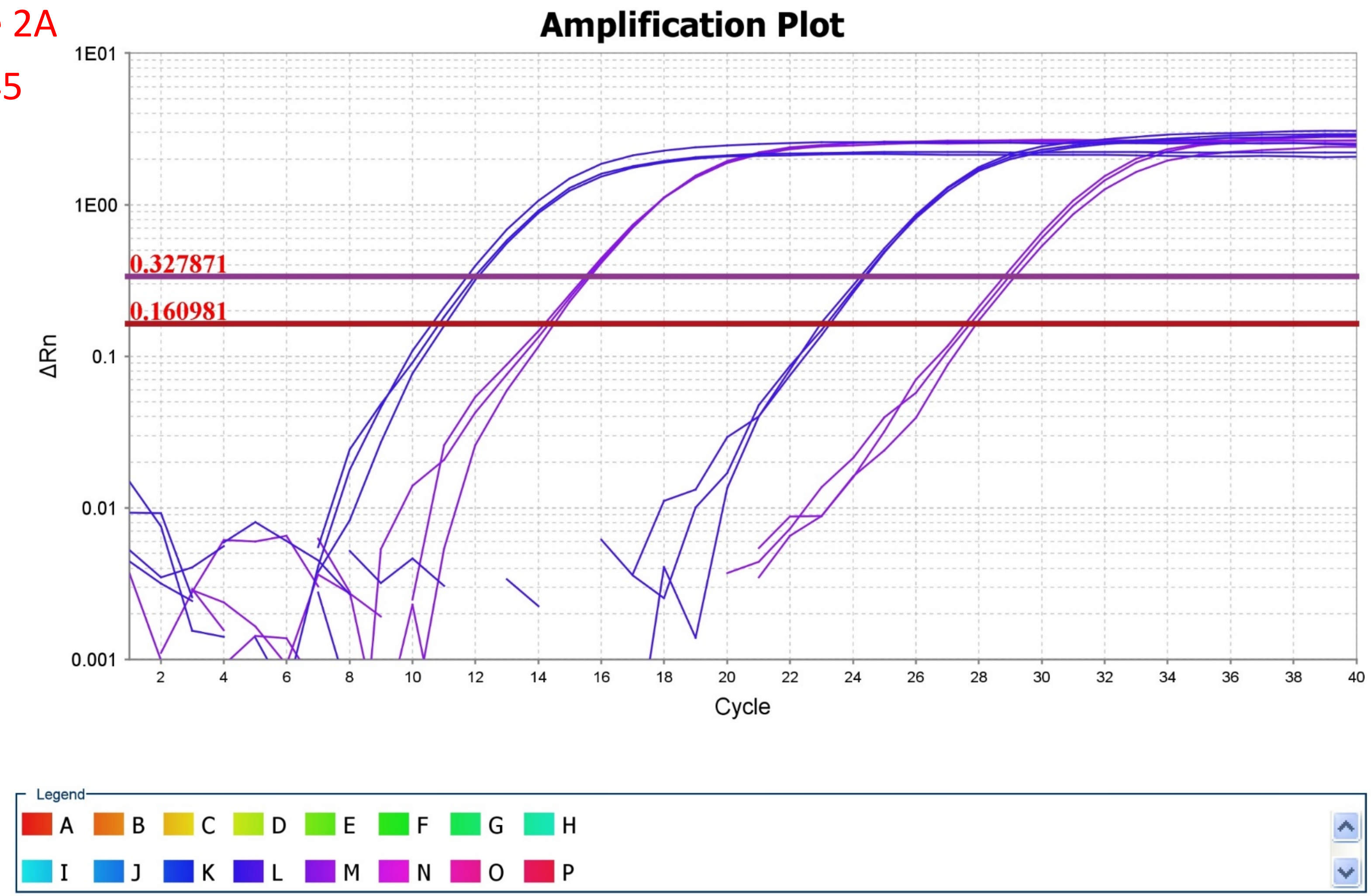

Figure 2A

DU 145

Block Type	96-Well Block (0.2mL)																	
Calibration	Yes																	
Calibration	2016-01-20 11:59:47 AM CST																	
Calibration	Yes																	
Calibration	2016-01-20 12:52:05 PM CST																	
Calibration	Yes																	
Calibration	2016-01-20 12:14:34 PM CST																	
Calibration	Yes																	
Calibration	2016-01-20 11:53:09 AM CST																	
Calibration	Yes																	
Calibration	2016-01-20 12:29:32 PM CST																	
Calibration	Yes																	
Calibration	2016-01-20 12:21:57 PM CST																	
Calibration	Yes																	
Calibration	2016-01-20 12:37:06 PM CST																	
Calibration	Yes																	
Calibration	2016-01-20 12:07:07 PM CST																	
Calibration	Yes																	
Calibration	2016-01-20 12:44:41 PM CST																	
Chemistry	SYBR_GREEN																	
Experiment	Barcode																	
Experiment	Comments																	
Experiment	File Name																	
Experimen	2018-06-19 162105																	
Experimen	2018-06-20 02:11:57 AM CST																	
Experimen	Comparative Cт (ΔΔCт)																	
Experiment	User Name																	
Instrument	278882256																	
Instrument	278882256																	
Instrument	ViiA 7																	
Passive Re	ROX																	
Quantificat	Ct																	
Signal Sm	true																	
Stage/ Cyc	Stage 2, Step 2																	
Well	Well Posit	Omit	Sample Name	Target Name	Task	Reporter	Quencher	Quantity	Quantity M	Quantity S	RQ	RQ Min	RQ Max	CT	Ct Mean	Ct SD	Delta Ct	Delta Ct M
271	L7	FALSE	DU 145-shCtrl	H-GAPDH	UNKNOWI	SYBR	None							10.810				
272	L8	FALSE	DU 146-shCtrl	H-GAPDH	UNKNOWI	SYBR	None							10.969				
273	L9	FALSE	DU 147-shCtrl	H-GAPDH	UNKNOWI	SYBR	None							10.608				
274	L10	FALSE	DU 148-shCtrl	H-CDKL3	UNKNOWI	SYBR	None							24.260				
275	L11	FALSE	DU 149-shCtrl	H-CDKL3	UNKNOWI	SYBR	None							24.313				
276	L12	FALSE	DU 150-shCtrl	H-CDKL3	UNKNOWI	SYBR	None							24.151				
295	M7	FALSE	DU 145-shCDKL3	H-GAPDH	UNKNOWI	SYBR	None							14.480				
296	M8	FALSE	DU 145-shCDKL3	H-GAPDH	UNKNOWI	SYBR	None							14.179				
297	M9	FALSE	DU 145-shCDKL3	H-GAPDH	UNKNOWI	SYBR	None							14.315				
298	M10	FALSE	DU 145-shCDKL3	H-CDKL3	UNKNOWI	SYBR	None							29.063				
299	M11	FALSE	DU 145-shCDKL3	H-CDKL3	UNKNOWI	SYBR	None							28.900				
300	M12	FALSE	DU 145-shCDKL3	H-CDKL3	UNKNOWI	SYBR	None							28.749				
Analysis T	Singleplex																	
Endogenous	Control																	
RQ Min/Ma	95.0																	
Reference	Sample																	

Figure 2A
DU 145

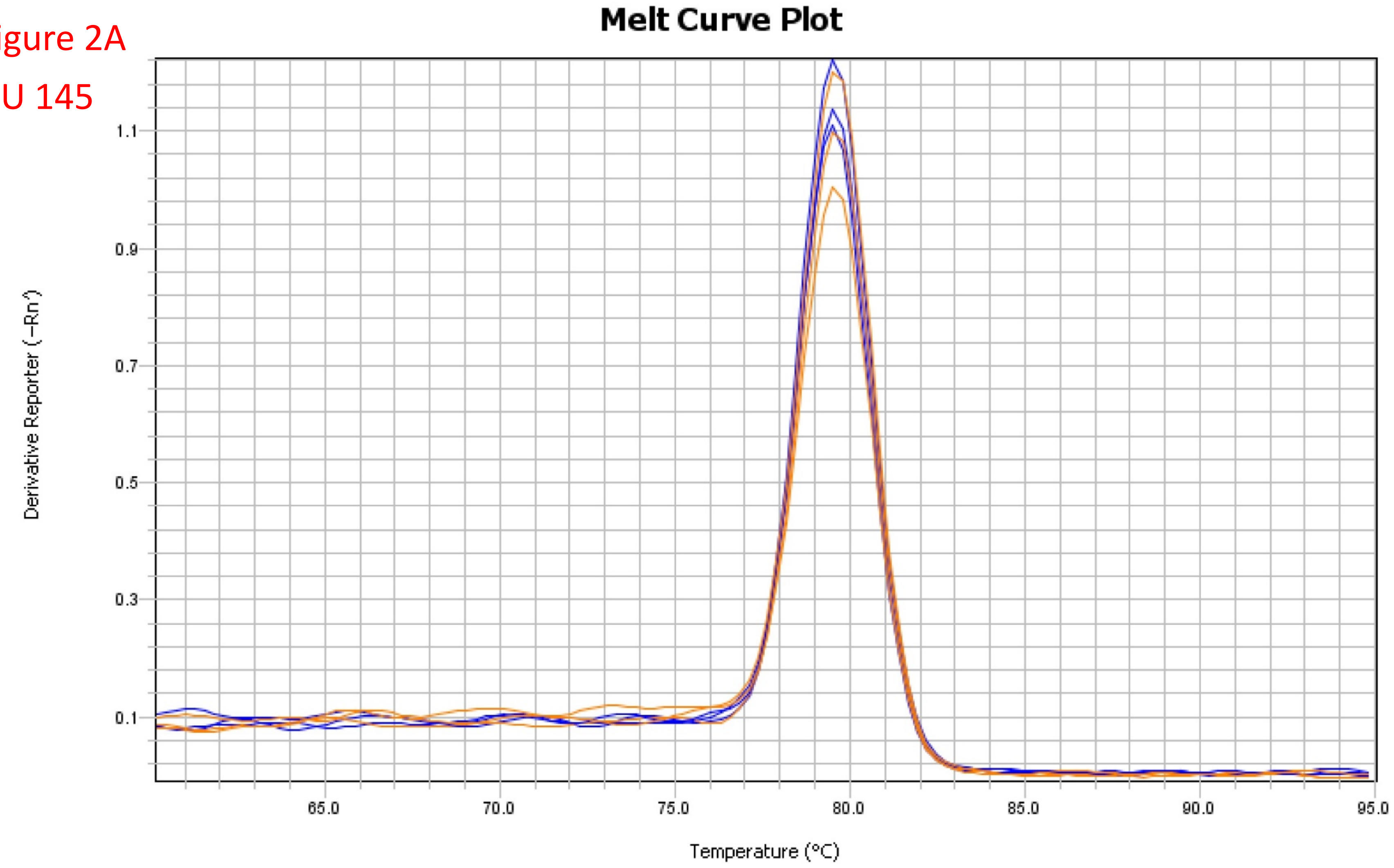

Figure 2A

DU 145

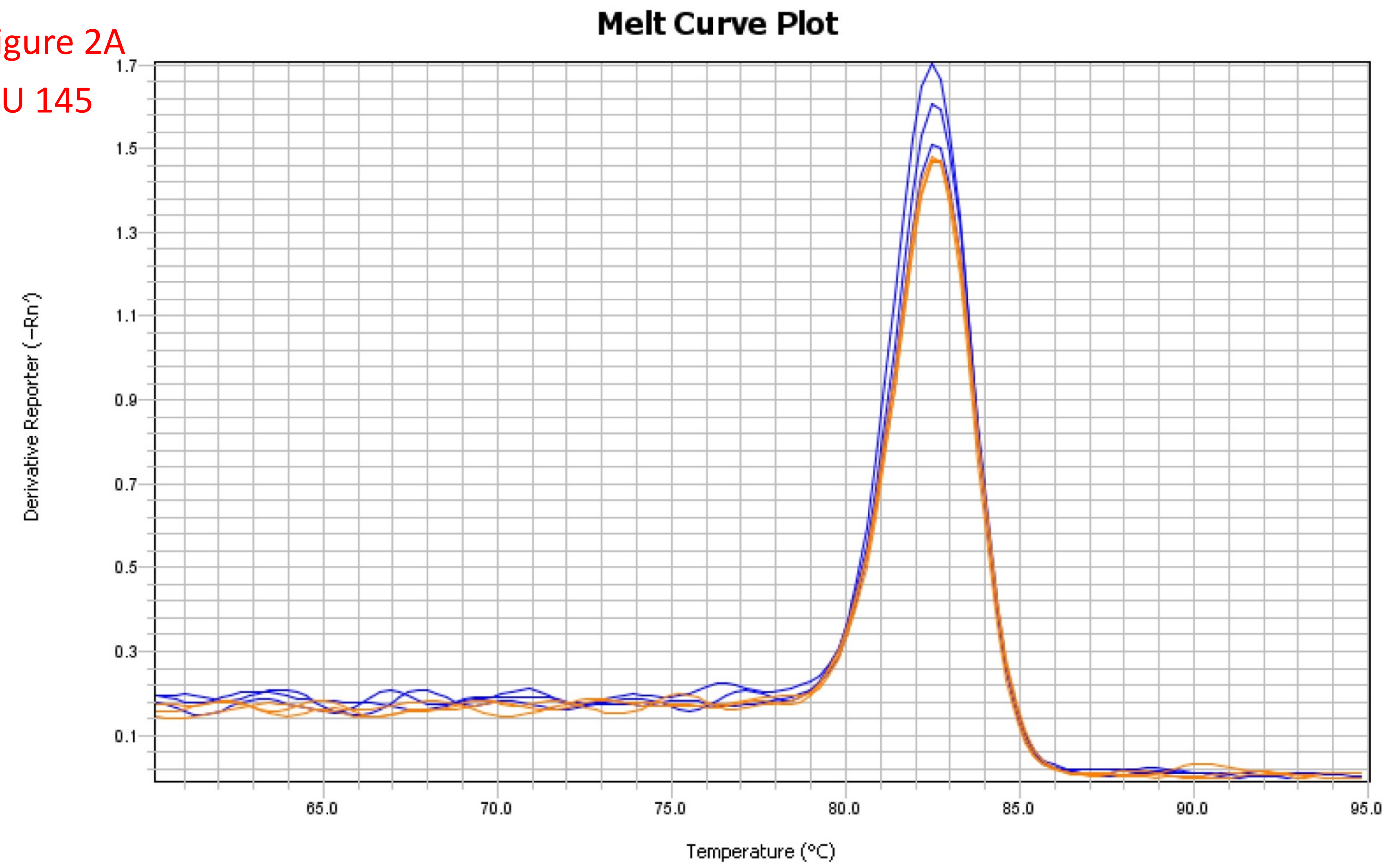

Figure 2A

PC-3

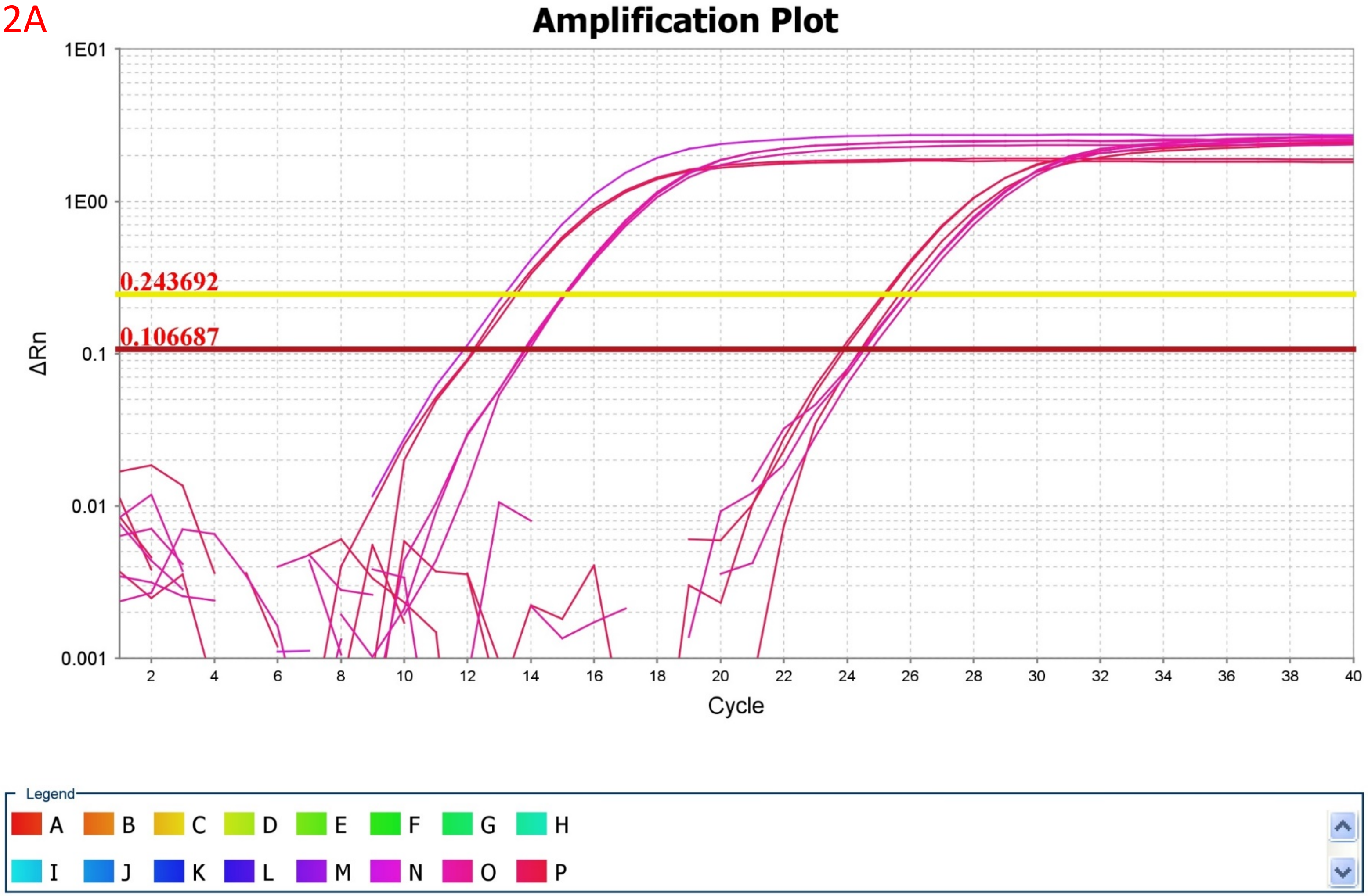

Figure 2A

PC-3

Block Type	384-Well Block
Calibration	Yes
Calibration	2016-01-20 10:12:38 AM CST
Calibration	Yes
Calibration	2016-01-20 10:28:26 AM CST
Calibration	Yes
Calibration	2016-01-20 10:44:55 AM CST
Calibration	Yes
Calibration	2016-01-20 10:05:17 AM CST
Calibration	Yes
Calibration	2016-01-20 11:25:36 AM CST
Calibration	Yes
Calibration	2016-01-20 10:35:50 AM CST
Calibration	Yes
Calibration	2016-01-20 11:02:33 AM CST
Calibration	Yes
Calibration	2016-01-20 10:20:14 AM CST
Calibration	Yes
Calibration	2016-01-20 10:54:46 AM CST
Chemistry	SYBR_GREEN
Experiment Barcode	
Experiment Comments	
Experiment File Name	
Experimen	2018-07-27 151603
Experimen	2018-07-28 00:36:49 AM CST
Experimen	Comparative Cт (ΔΔCт)
Experiment User Name	
Instrument	278882256
Instrument	278882256
Instrument	ViiA 7
Passive Re	ROX
Quantificat	Ct
Signal Sm	true
Stage/ Cyc	Stage 2, Step 2

Well	Well Posit	Omit	Sample Name	Target Name	Task	Reporter	Quencher	Quantity	Quantity M	Quantity S	RQ	RQ Min	RQ Max	CT	Ct Mean	Ct SD	Delta Ct
343	O7	FALSE	PC-3-shCtrl	H-GAPDH	UNKNOWN	SYBR	None							13.923			
344	O8	FALSE	PC-3-shCtrl	H-GAPDH	UNKNOWN	SYBR	None							13.958			
345	O9	FALSE	PC-3-shCtrl	H-GAPDH	UNKNOWN	SYBR	None							14.032			
346	O10	FALSE	PC-3-shCtrl	H-CDKL3	UNKNOWN	SYBR	None							25.857			
347	O11	FALSE	PC-3-shCtrl	H-CDKL3	UNKNOWN	SYBR	None							25.838			
348	O12	FALSE	PC-3-shCtrl	H-CDKL3	UNKNOWN	SYBR	None							26.040			
367	P7	FALSE	PC-3-shCDKL3	H-GAPDH	UNKNOWN	SYBR	None							12.241			
368	P8	FALSE	PC-3-shCDKL3	H-GAPDH	UNKNOWN	SYBR	None							12.375			
369	P9	FALSE	PC-3-shCDKL3	H-GAPDH	UNKNOWN	SYBR	None							11.927			
370	P10	FALSE	PC-3-shCDKL3	H-CDKL3	UNKNOWN	SYBR	None							25.088			
371	P11	FALSE	PC-3-shCDKL3	H-CDKL3	UNKNOWN	SYBR	None							25.499			
372	P12	FALSE	PC-3-shCDKL3	H-CDKL3	UNKNOWN	SYBR	None							25.163			

Analysis T	Singleplex
Endogenous Control	
RQ Min/Ma	95.0
Reference Sample	

Figure 2A

PC-3

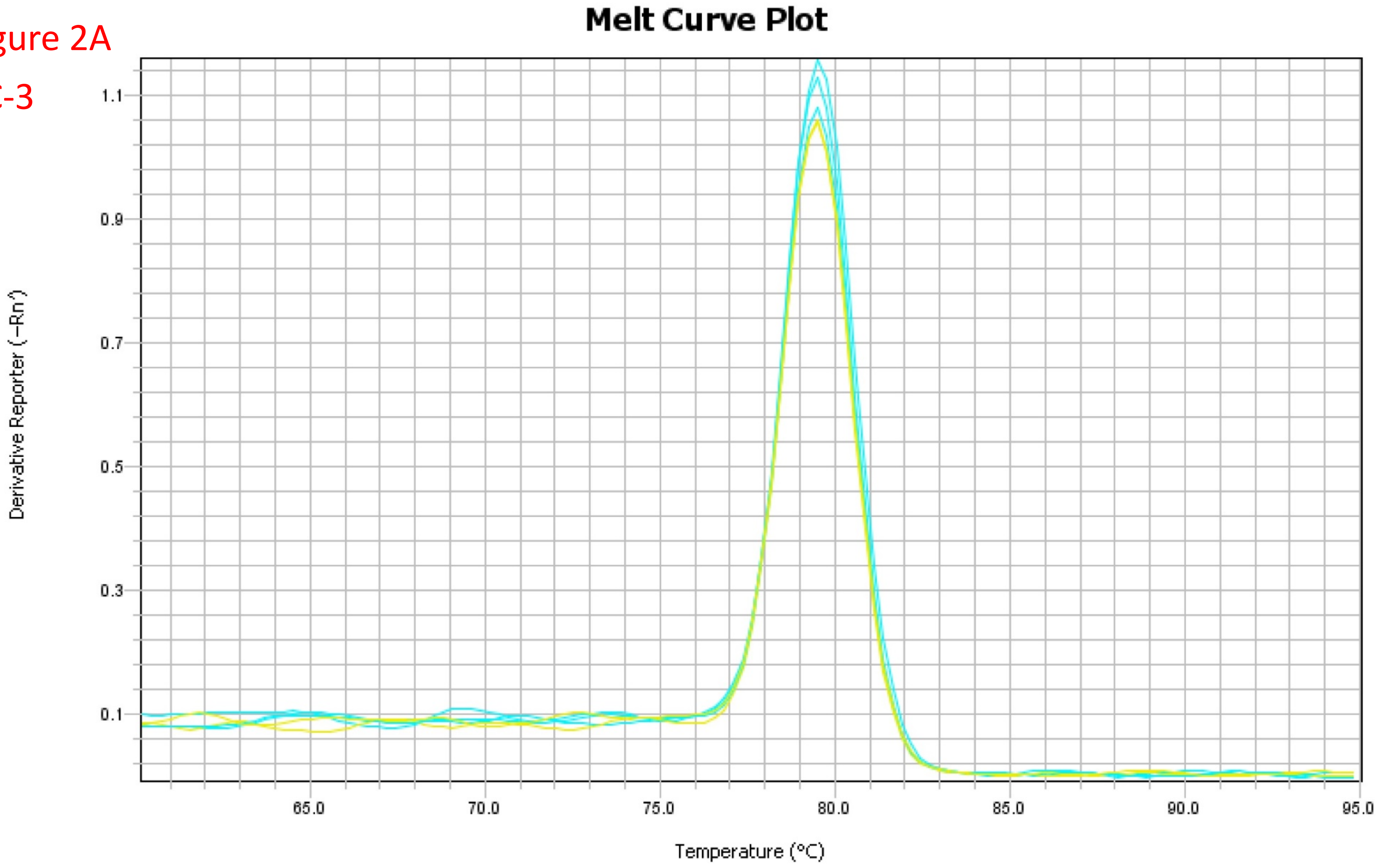

Figure 2A

PC-3

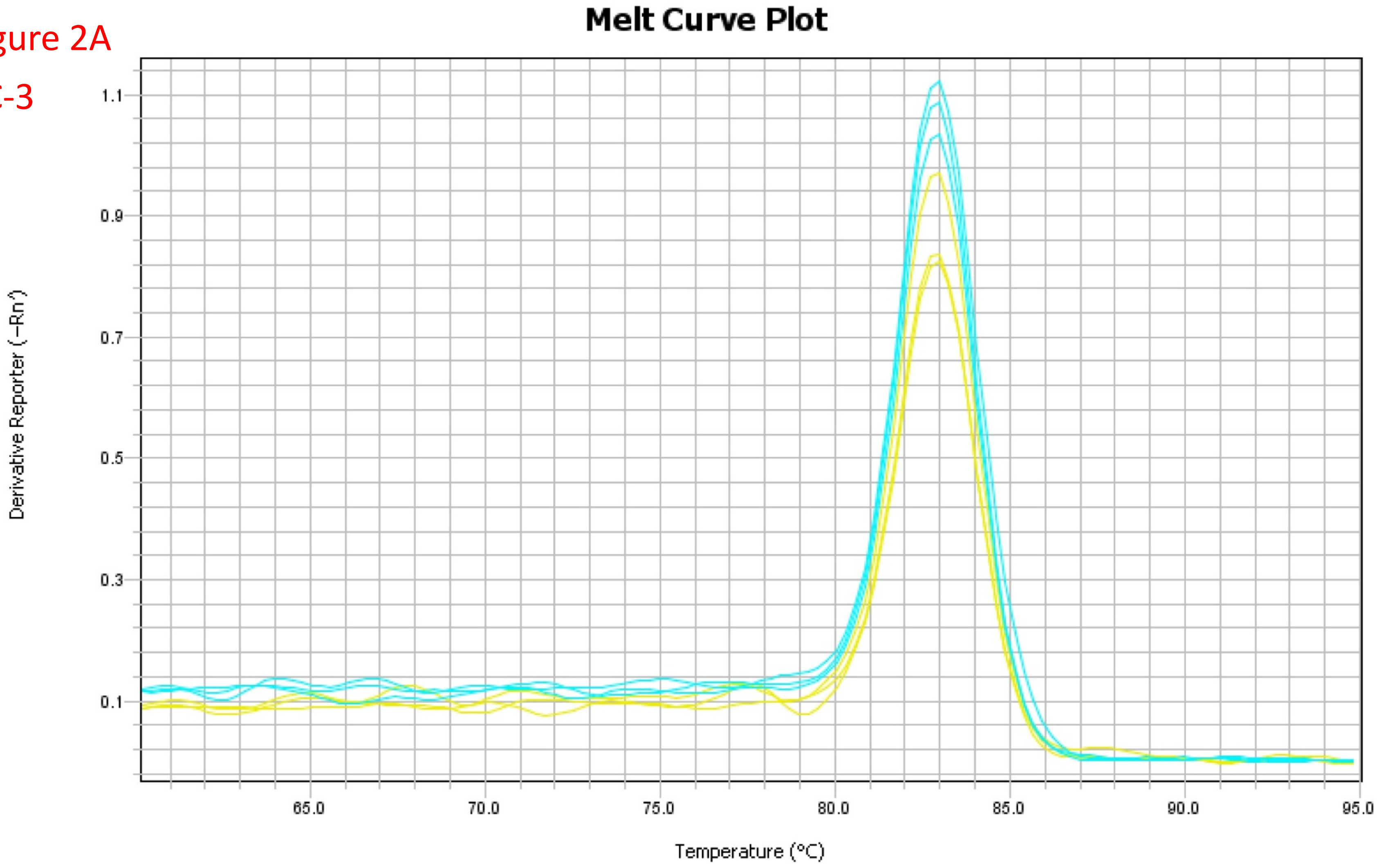

Figure 2A

DU 145

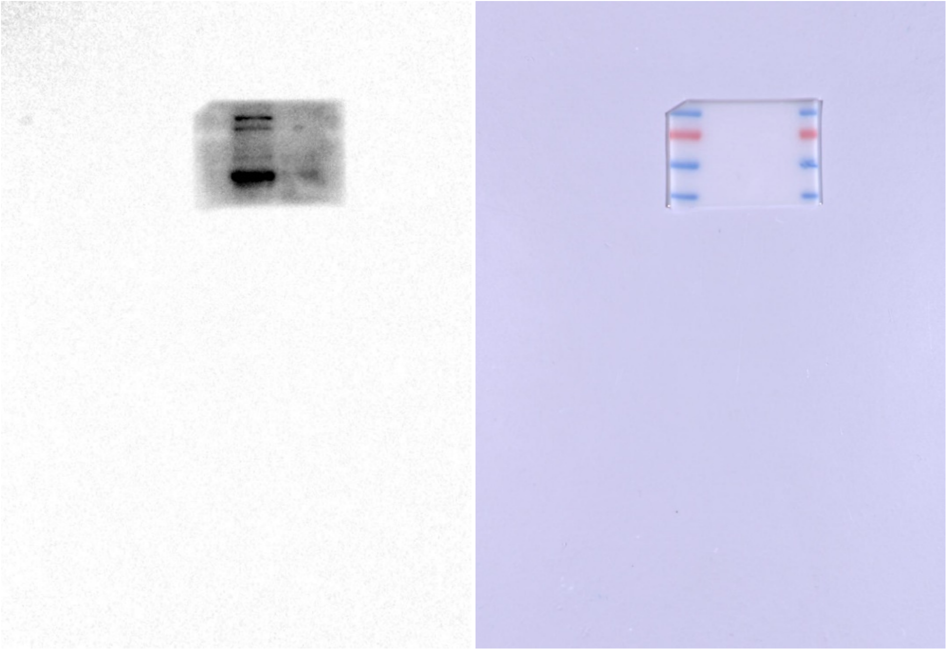

DU145 CDKL3

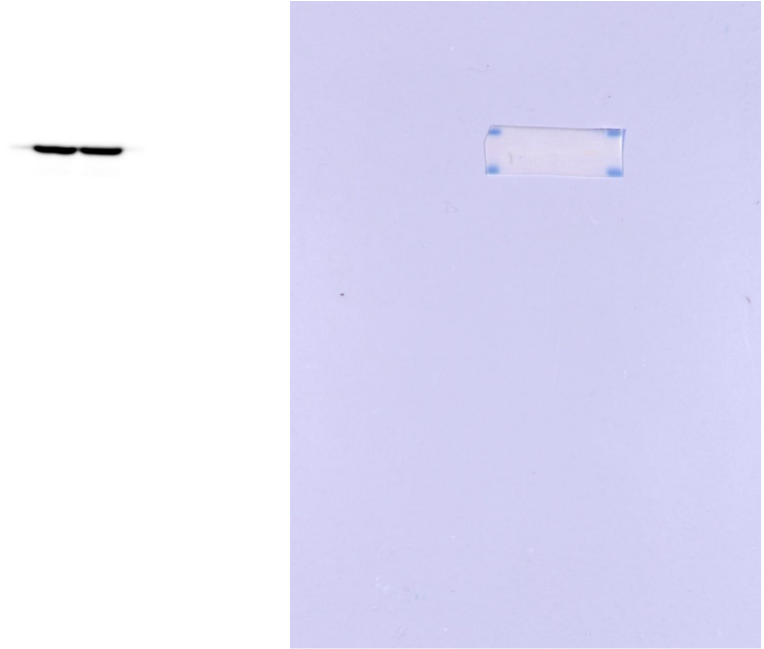

DU145 GAPDH

Figure 2A

PC-3

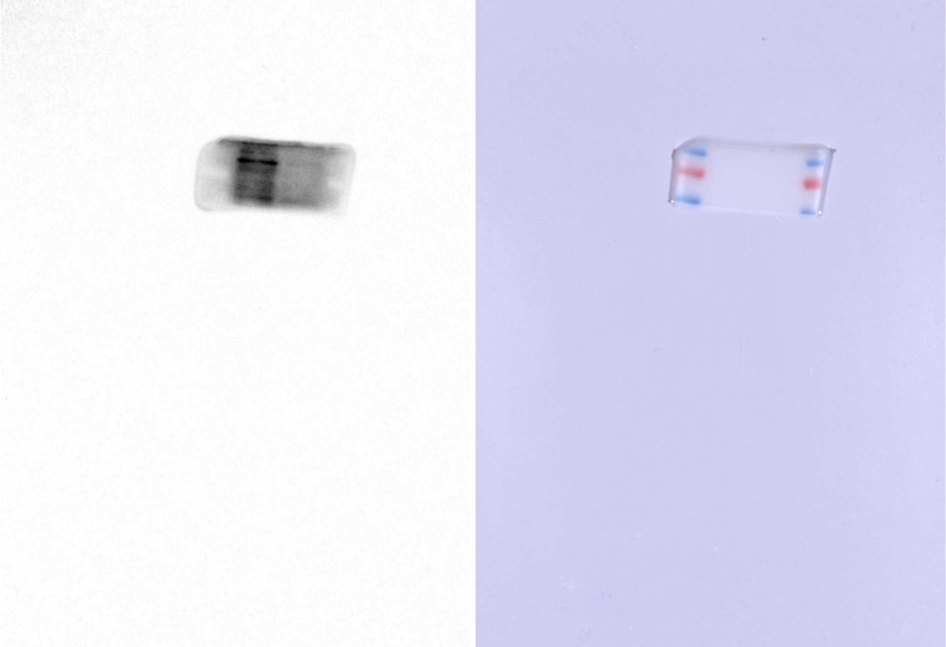

PC-3 CDKL3

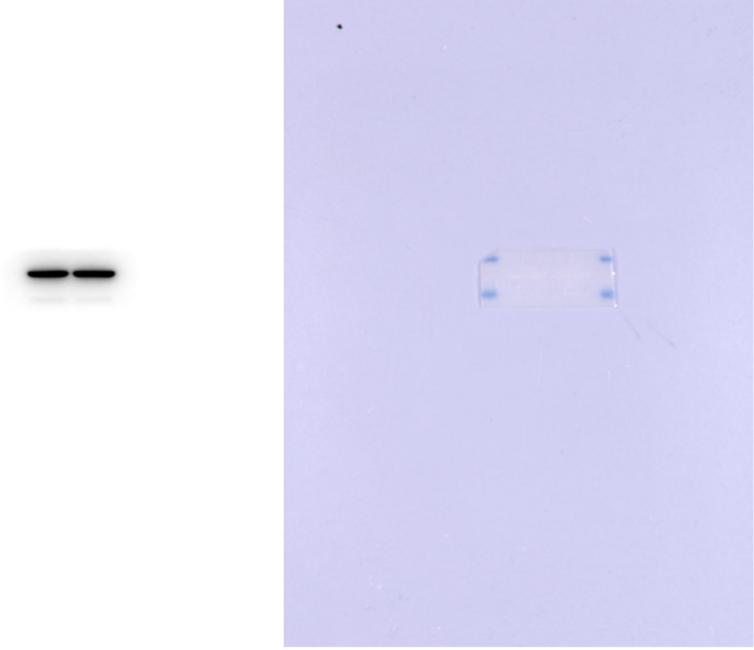

PC-3 GAPDH

Figure 2G and S3 DU 145

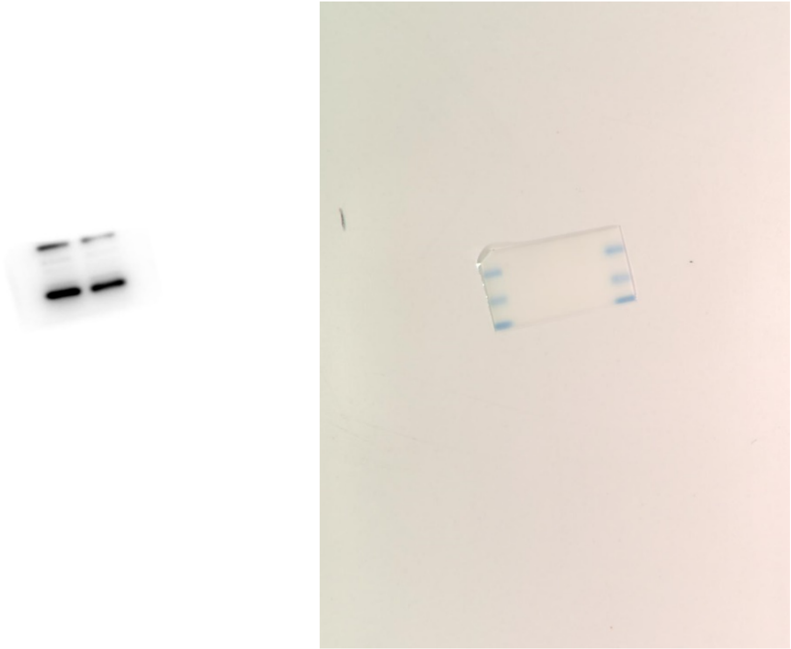

Bcl-2

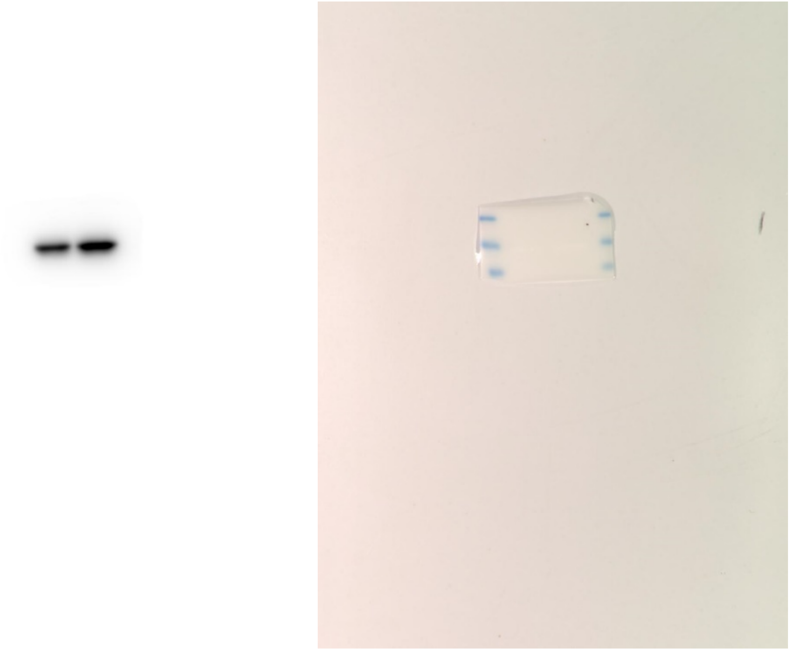

Caspase-3

Figure 2G and S3 DU 145

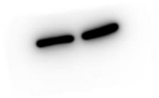

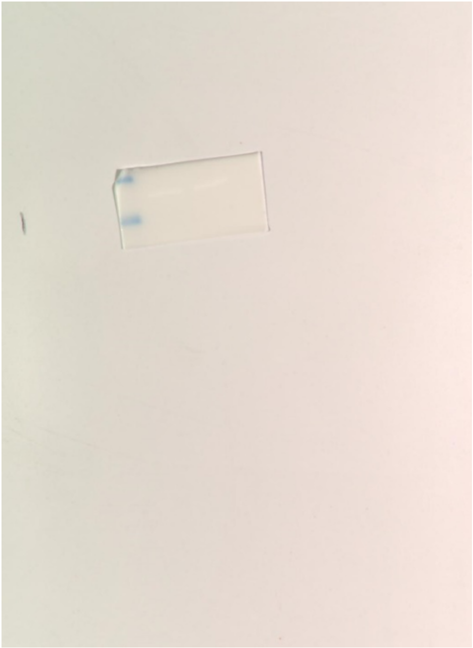

GAPDH

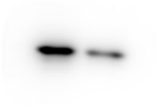

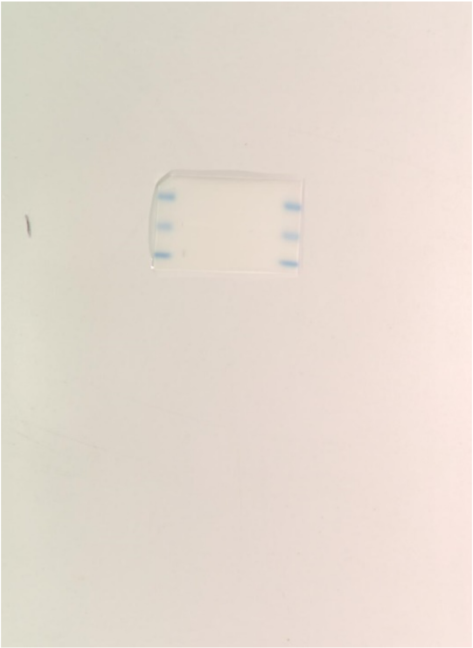

HSP27

Figure 2G and S3 PC-3

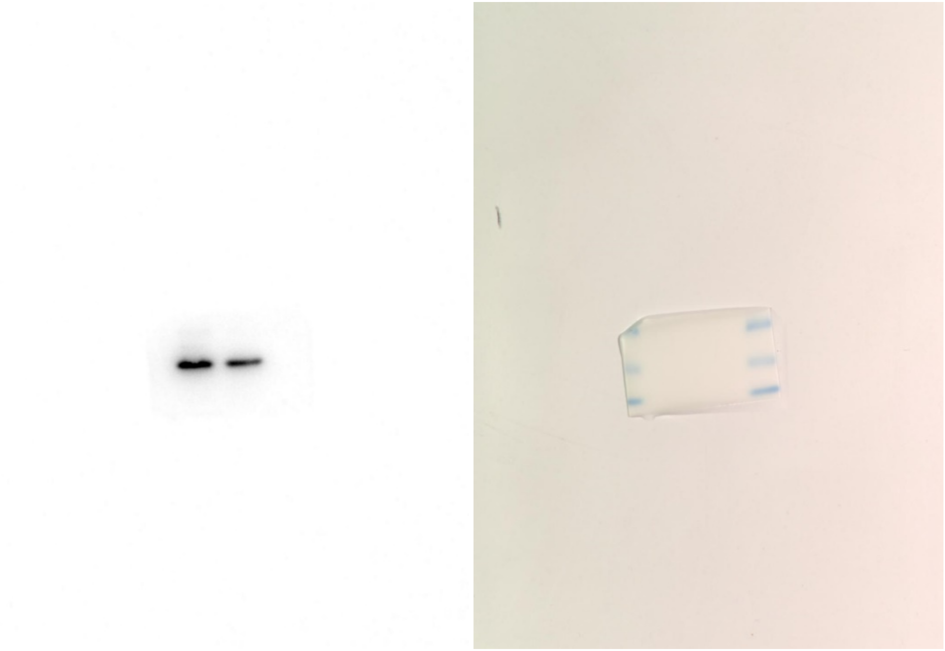

Bcl-2

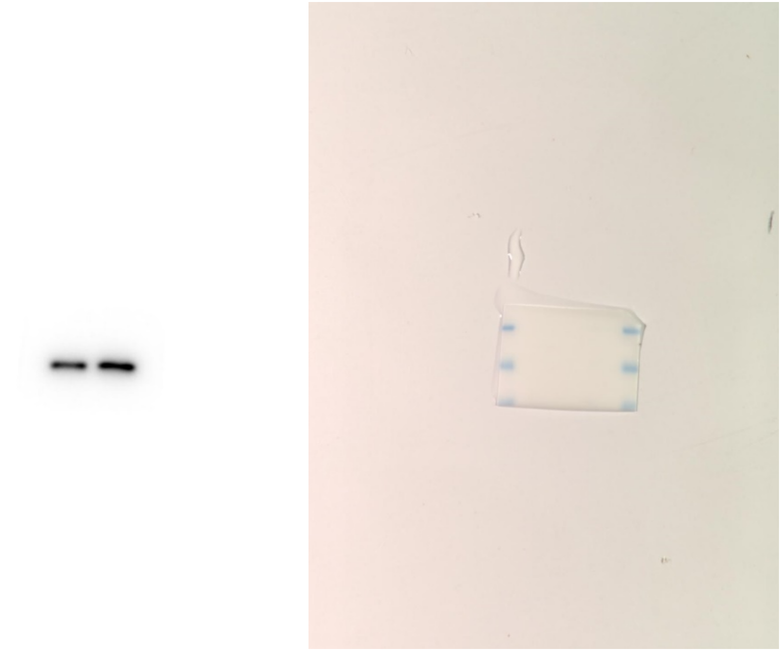

Caspase-3

Figure 2G and S3 PC-3

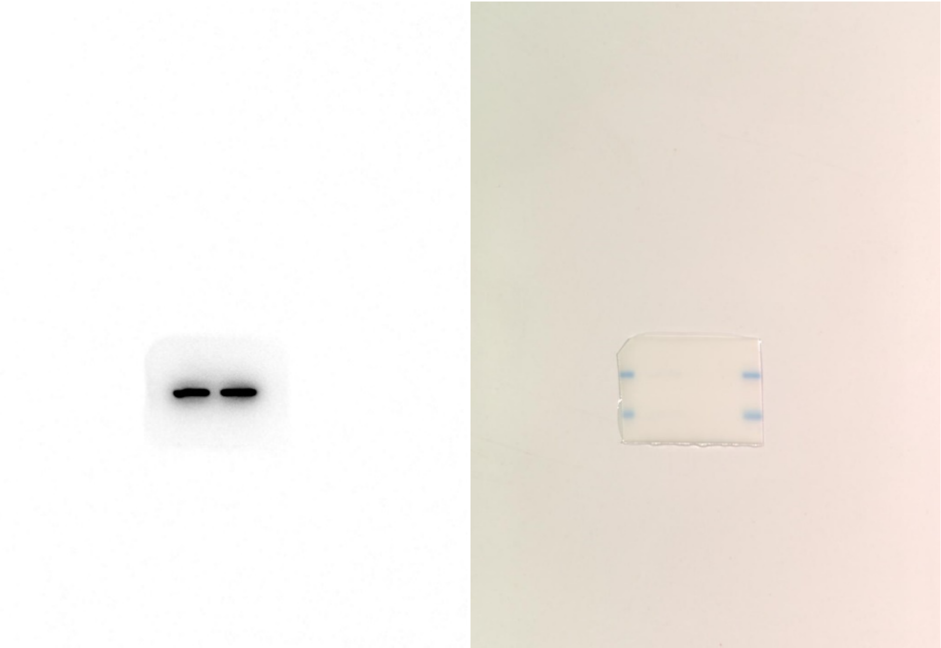

GAPDH

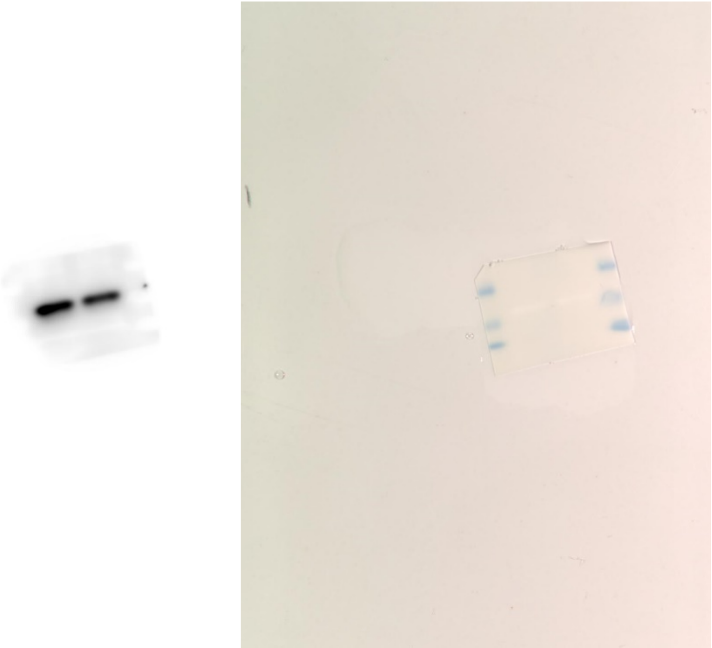

HSP27

Figure 4B

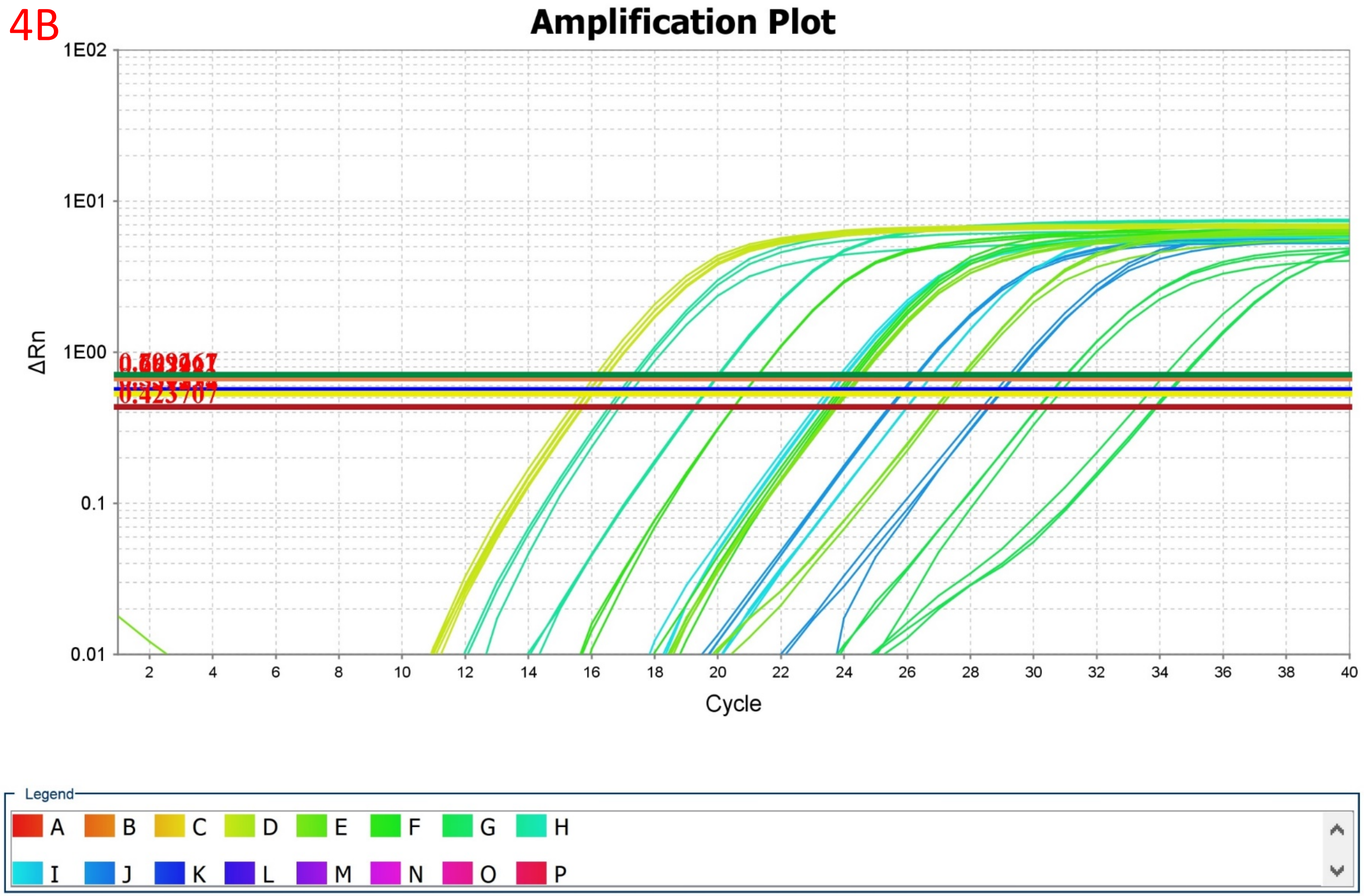
Amplification Plot
1E02
1E01
1E00
0.1
0.01
ΔRn
2
4
6
8
10
12
14
16
18
20
22
24
26
28
30
32
34
36
38
40
Cycle
Legend
A
B
C
D
E
F
G
H
I
J
K
L
M
N
O
P

Figure 4B

Block Type	384-Well Block
Calibration E	No
Calibration E	2022-08-01 16:00:17 PM CST
Calibration F	No
Calibration F	2022-08-01 16:39:53 PM CST
Calibration N	No
Calibration N	2022-08-01 16:56:18 PM CST
Calibration F	No
Calibration F	2022-08-01 15:53:01 PM CST
Calibration F	No
Calibration F	2022-08-01 17:25:54 PM CST
Calibration S	No
Calibration S	2022-08-01 17:03:33 PM CST
Calibration T	No
Calibration T	2022-08-01 17:10:48 PM CST
Calibration U	No
Calibration U	2022-08-01 16:16:55 PM CST
Calibration V	No
Calibration V	2022-08-01 16:48:55 PM CST
Chemistry	SYBR_GREEN
Experiment	Barcode
Experiment	Comments
Experiment	C:\Users\zhouch\Desktop\2022-08-08 ly 1.eds
Experiment	2022-08-08 ly 1
Experiment	2022-08-08 17:43:11 PM CST
Experiment	Comparative Cт (ΔΔCт)
Experiment	User Name
Instrument N	278882256
Instrument S	278882256
Instrument T	ViiA 7
Passive Ref	ROX
Quantificatic	Ct
Signal Smo	true
Stage/ Cycl	Stage 2, Step 2

Well	Well Pos	Omit	Sample N	Target Na	Task	Reporter	Quenche	Quantity	Quantity	Quantity	RQ	RQ Min	RQ Max	CT	Ct Mean	Ct SD	Delta Ct
76	D4	FALSE	DU 145 shC	H-GAPDH	UNKNOWN	SYBR	None							15.507			
77	D5	FALSE	DU 145 shC	H-GAPDH	UNKNOWN	SYBR	None							15.350			
78	D6	FALSE	DU 145 shC	H-GAPDH	UNKNOWN	SYBR	None							15.537			
100	E4	FALSE	DU 145 shC	H-CDKL3	UNKNOWN	SYBR	None							27.340			
101	E5	FALSE	DU 145 shC	H-CDKL3	UNKNOWN	SYBR	None							27.434			
102	E6	FALSE	DU 145 shC	H-CDKL3	UNKNOWN	SYBR	None							27.318			
124	F4	FALSE	DU 145 shC	H-STAT1	UNKNOWN	SYBR	None							24.258			
125	F5	FALSE	DU 145 shC	H-STAT1	UNKNOWN	SYBR	None							24.341			
126	F6	FALSE	DU 145 shC	H-STAT1	UNKNOWN	SYBR	None							24.174			
148	G4	FALSE	DU 145 shC	H-FOS	UNKNOWN	SYBR	None							33.630			
149	G5	FALSE	DU 145 shC	H-FOS	UNKNOWN	SYBR	None							34.192			
150	G6	FALSE	DU 145 shC	H-FOS	UNKNOWN	SYBR	None							34.262			
172	H4	FALSE	DU 145 shC	H-ATF4	UNKNOWN	SYBR	None							19.652			
173	H5	FALSE	DU 145 shC	H-ATF4	UNKNOWN	SYBR	None							19.621			
174	H6	FALSE	DU 145 shC	H-ATF4	UNKNOWN	SYBR	None							19.673			
196	I4	FALSE	DU 145 shC	H-NRAS	UNKNOWN	SYBR	None							26.675			
197	I5	FALSE	DU 145 shC	H-NRAS	UNKNOWN	SYBR	None							26.691			
198	I6	FALSE	DU 145 shC	H-NRAS	UNKNOWN	SYBR	None							26.680			
220	J4	FALSE	DU 145 shC	H-PIK3CB	UNKNOWN	SYBR	None							29.328			
221	J5	FALSE	DU 145 shC	H-PIK3CB	UNKNOWN	SYBR	None							29.402			
222	J6	FALSE	DU 145 shC	H-PIK3CB	UNKNOWN	SYBR	None							29.181			

Analysis Ty	Singleplex
Endogenous	H-TGFBR1
RQ Min/Max	95.0
Reference S	Sample 1

Figure 4B

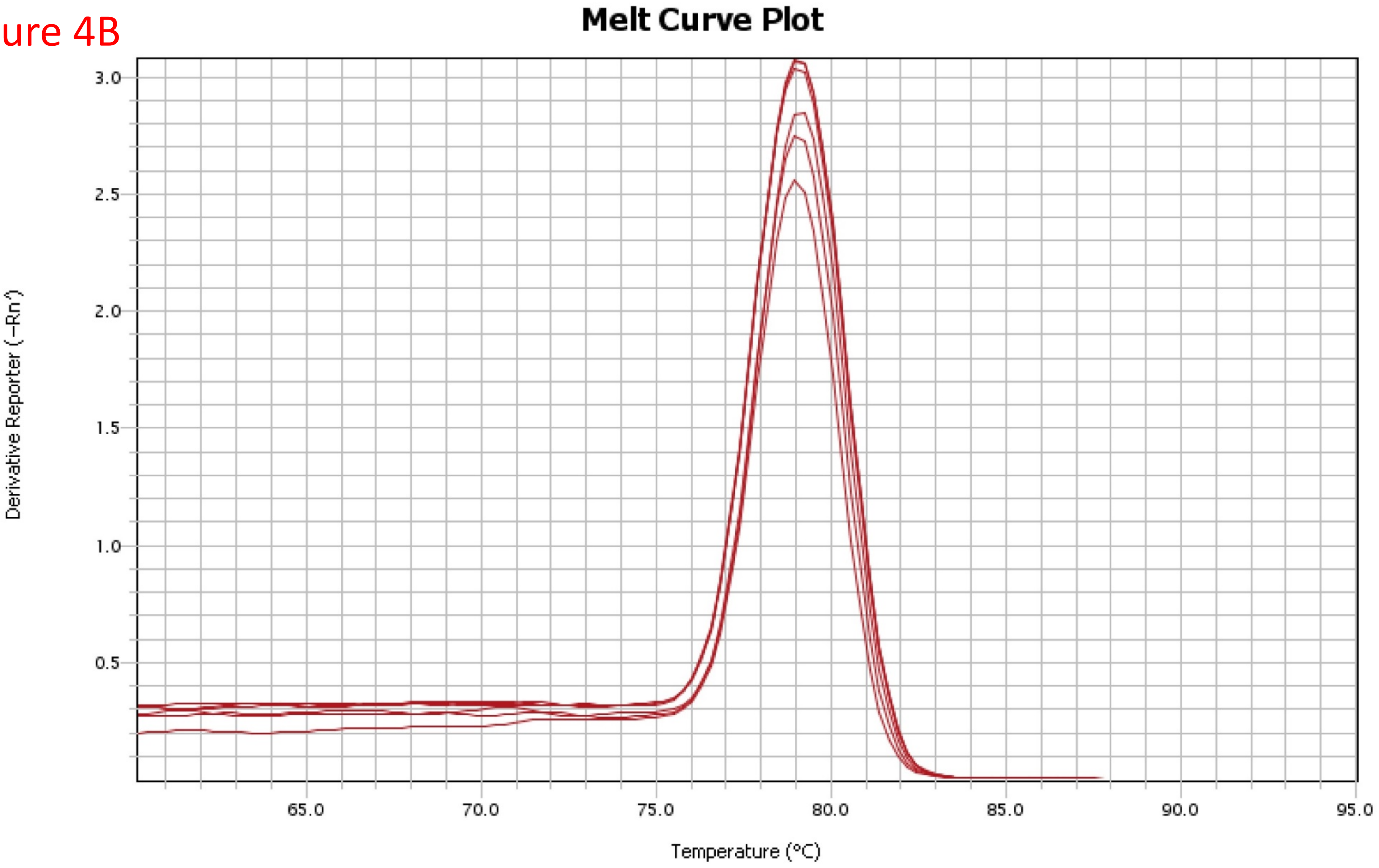

Melt Curve Plot
3.0
2.5
2.0
1.5
1.0
0.5
Derivative Reporter (−Rn')
65.0
70.0
75.0
80.0
85.0
90.0
95.0
Temperature (°C)

Figure 4B
Figure 4B

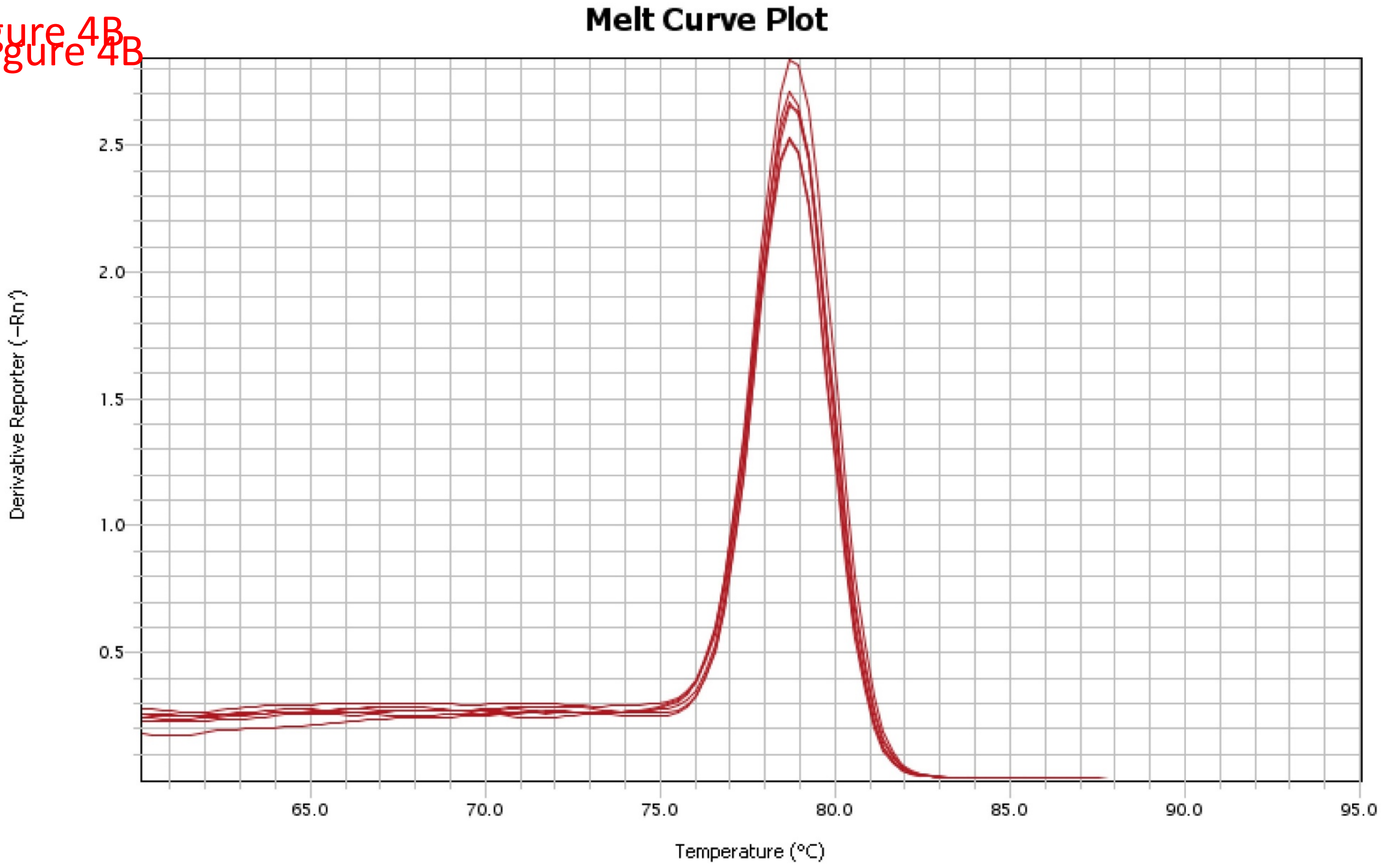

Figure 4B

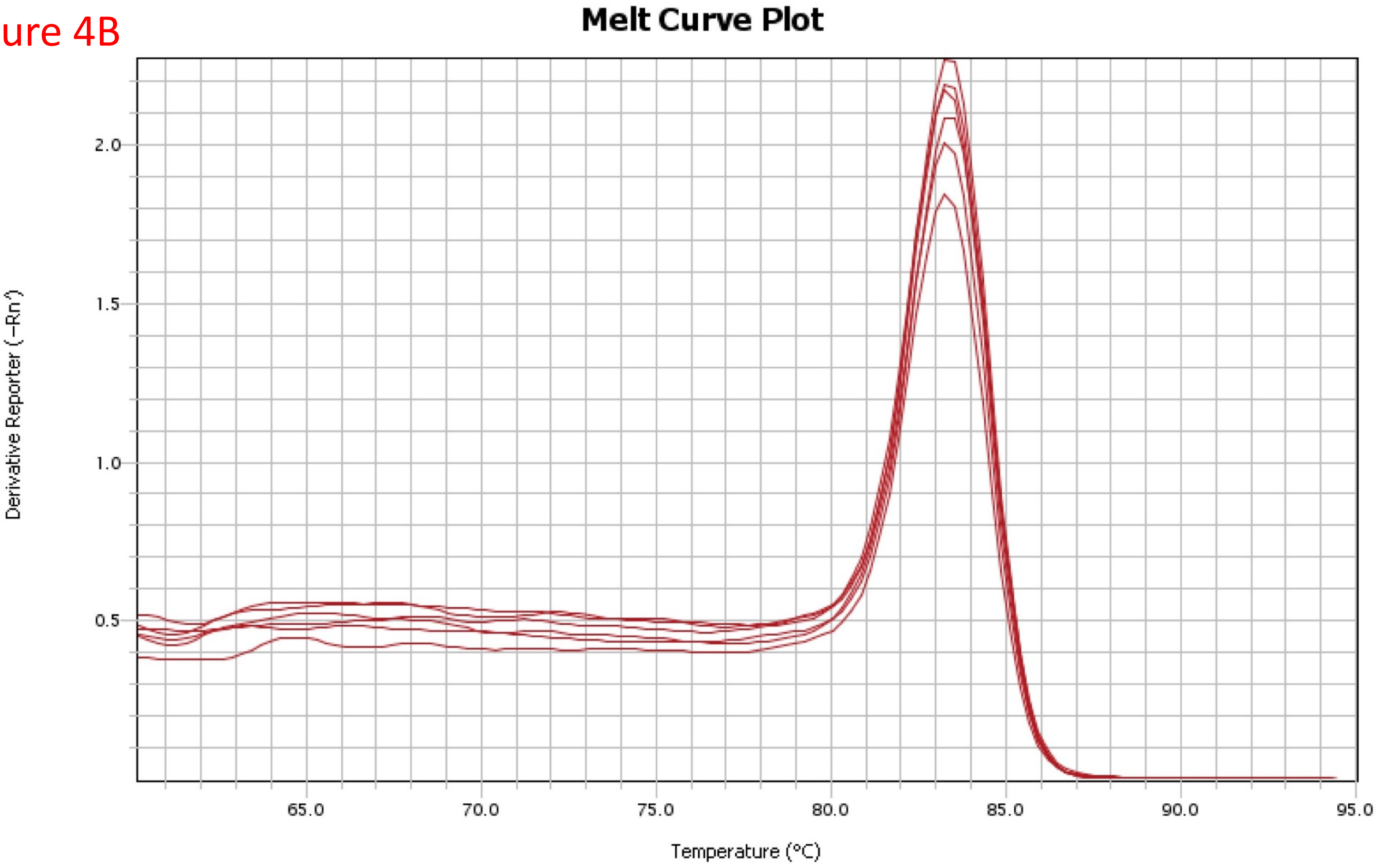
Melt Curve Plot
Derivative Reporter (−Rn')
2.0
1.5
1.0
0.5
65.0
70.0
75.0
80.0
85.0
90.0
95.0
Temperature (°C)

Figure 4B

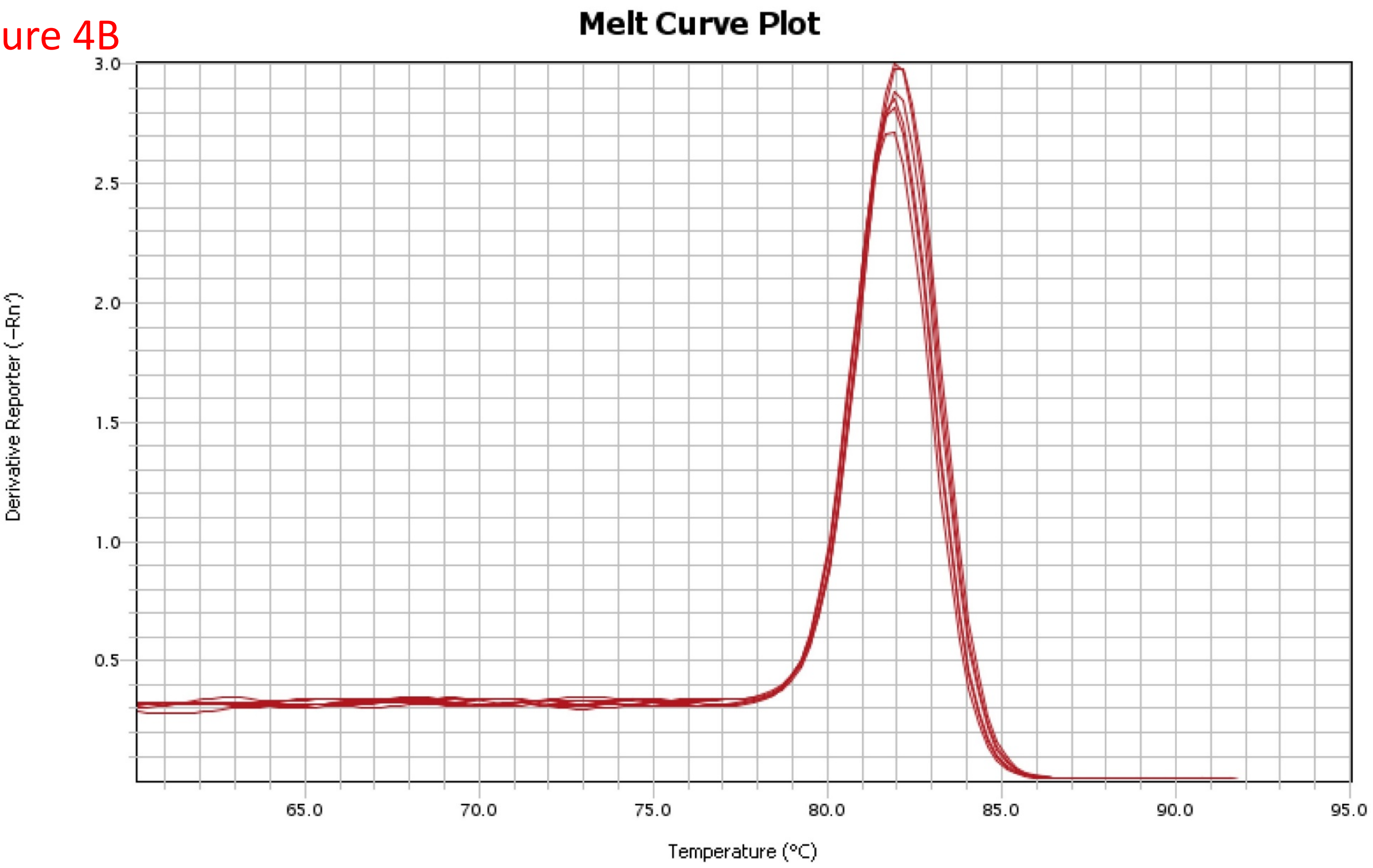
Melt Curve Plot
3.0
2.5
2.0
1.5
1.0
0.5
Derivative Reporter (−Rn')
65.0
70.0
75.0
80.0
85.0
90.0
95.0
Temperature (°C)

Figure 4B

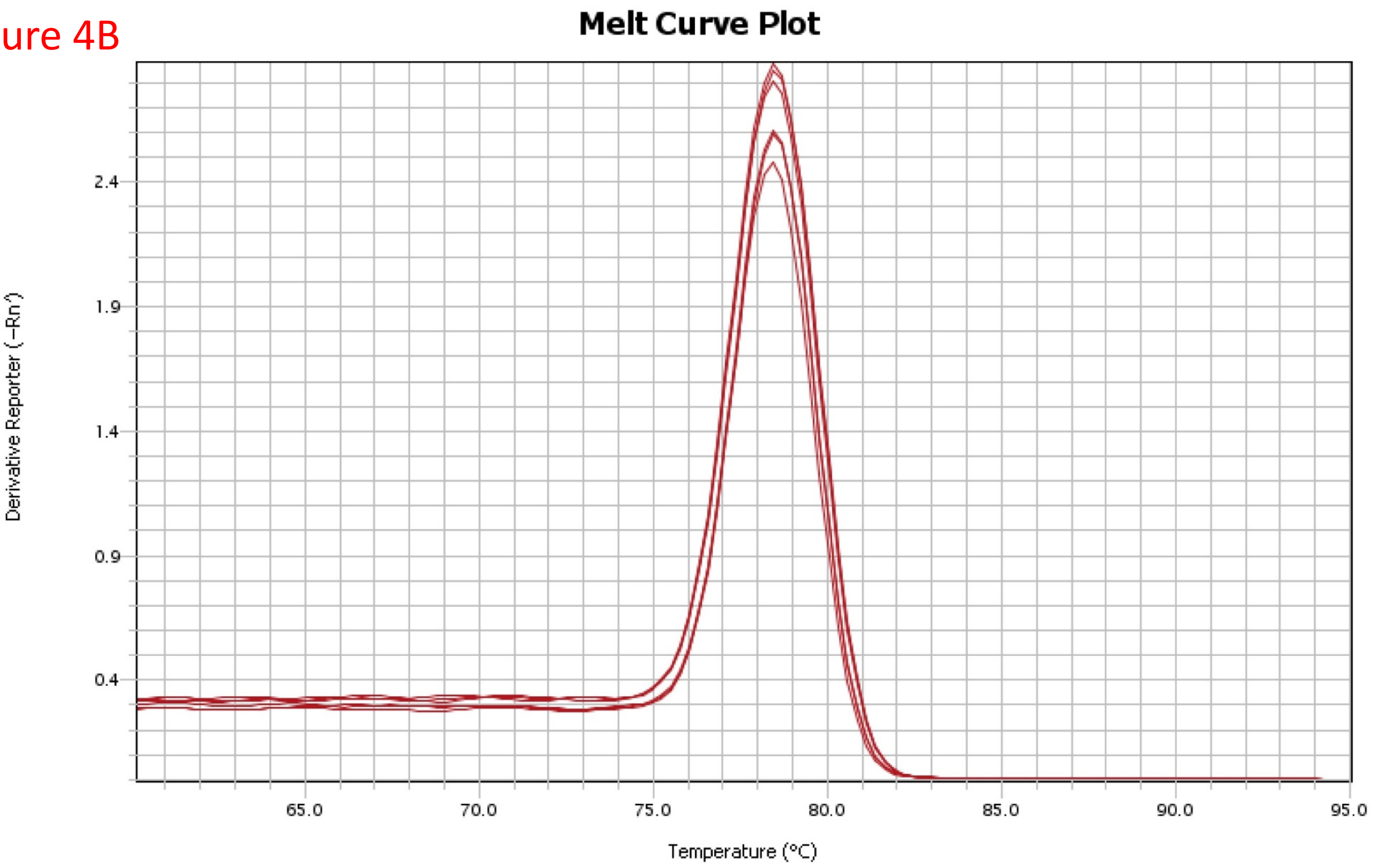

Melt Curve Plot
2.4
1.9
1.4
0.9
0.4
Derivative Reporter (−Rn')
65.0
70.0
75.0
80.0
85.0
90.0
95.0
Temperature (°C)

Figure 4B

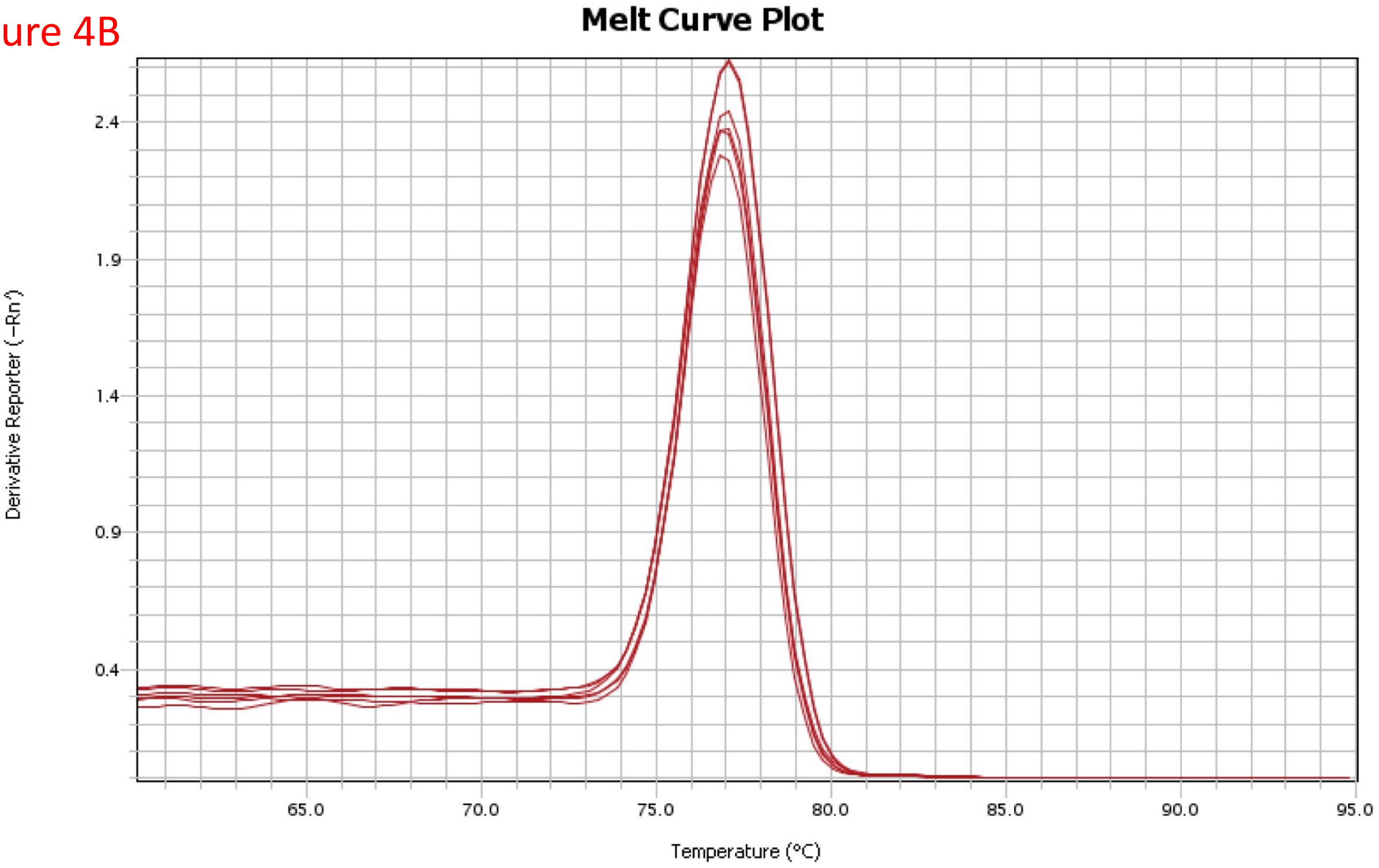
Melt Curve Plot
2.4
1.9
1.4
0.9
0.4
Derivative Reporter (−Rn')
65.0
70.0
75.0
80.0
85.0
90.0
95.0
Temperature (°C)

Figure 4B

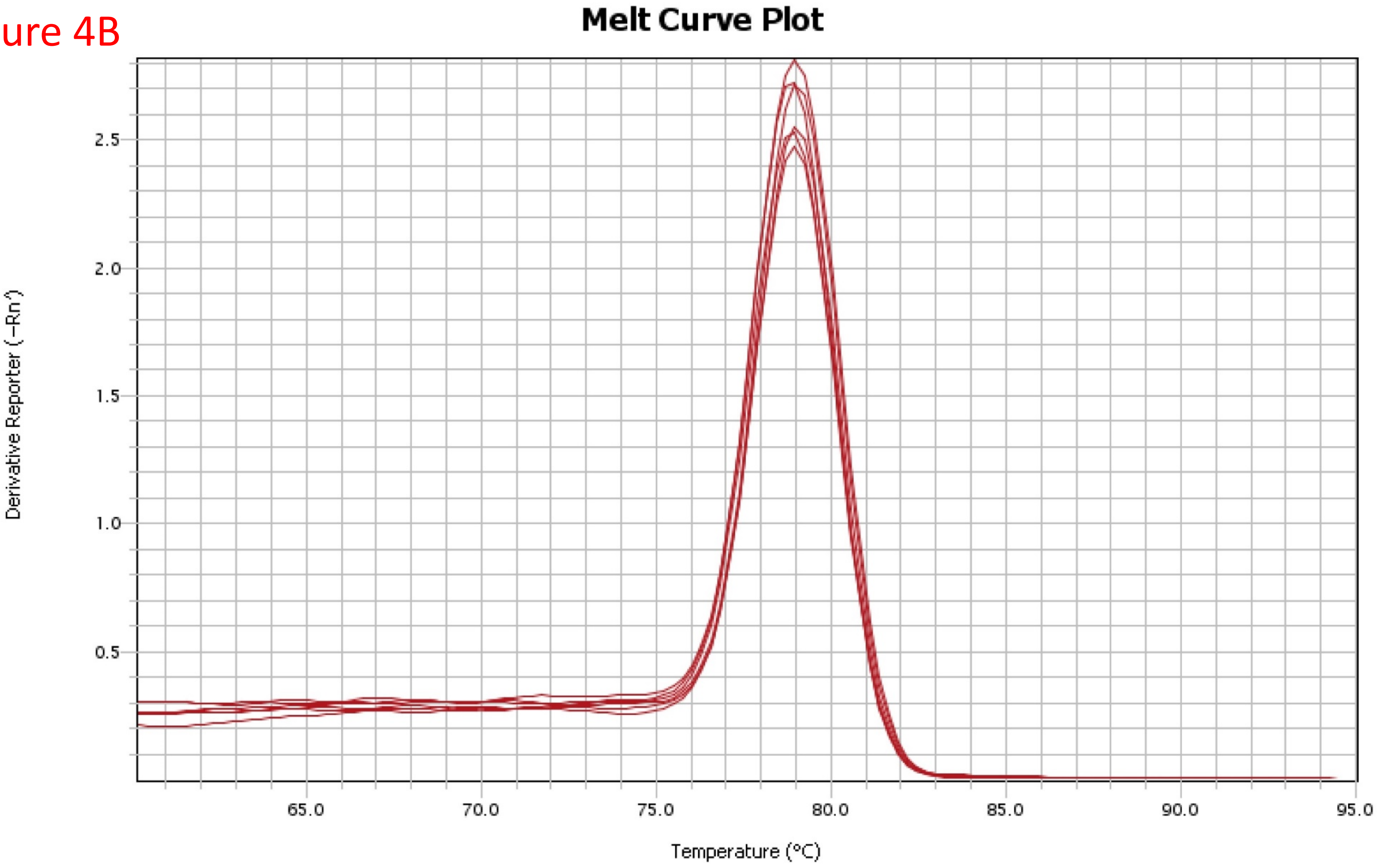
Melt Curve Plot
Derivative Reporter (−Rn')
2.5
2.0
1.5
1.0
0.5
65.0
70.0
75.0
80.0
85.0
90.0
95.0
Temperature (°C)

Figure 4C

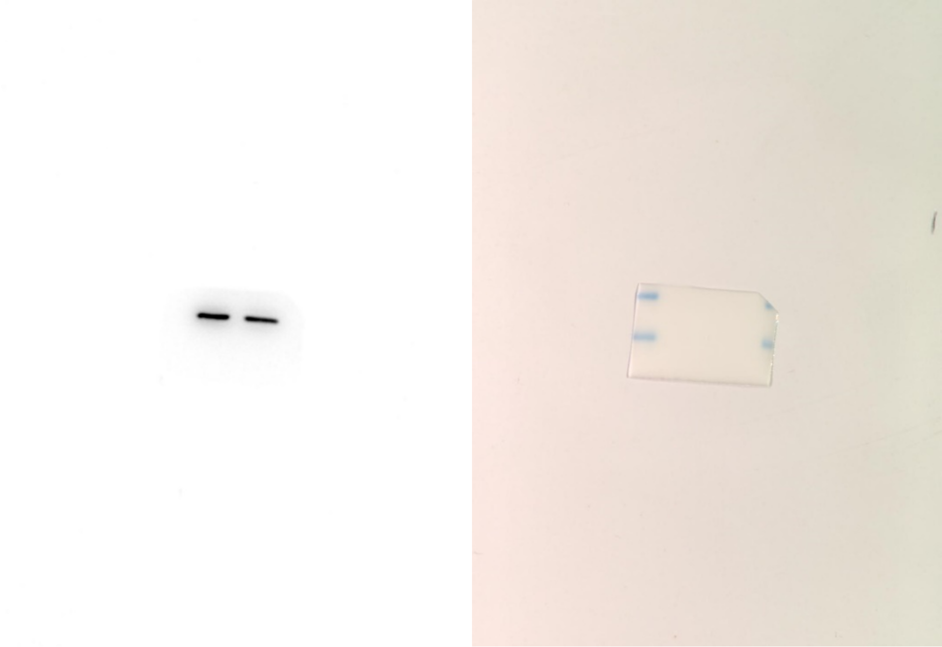

ATF4

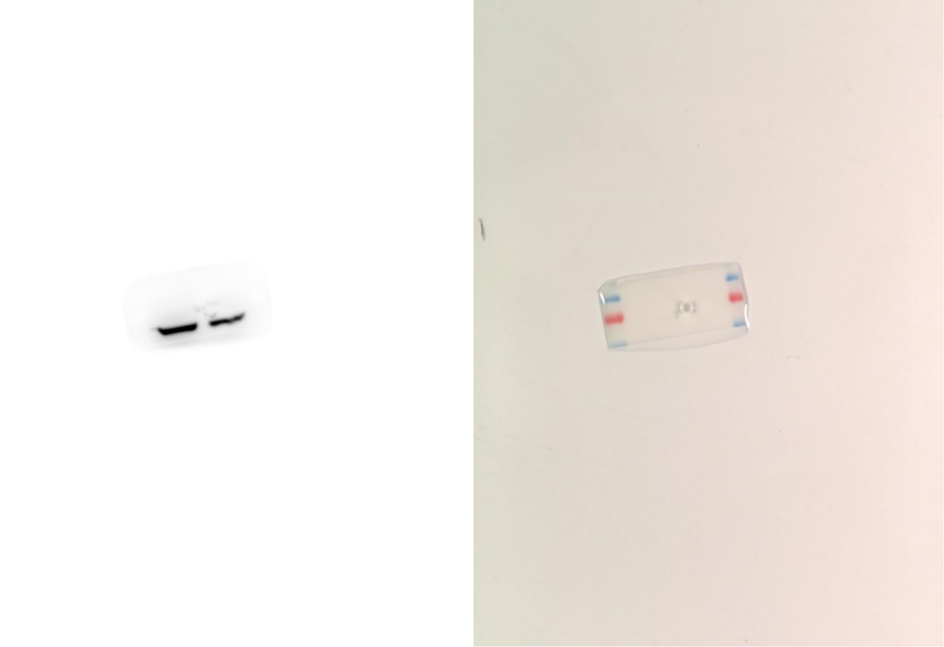

CDKL3

Figure 4C

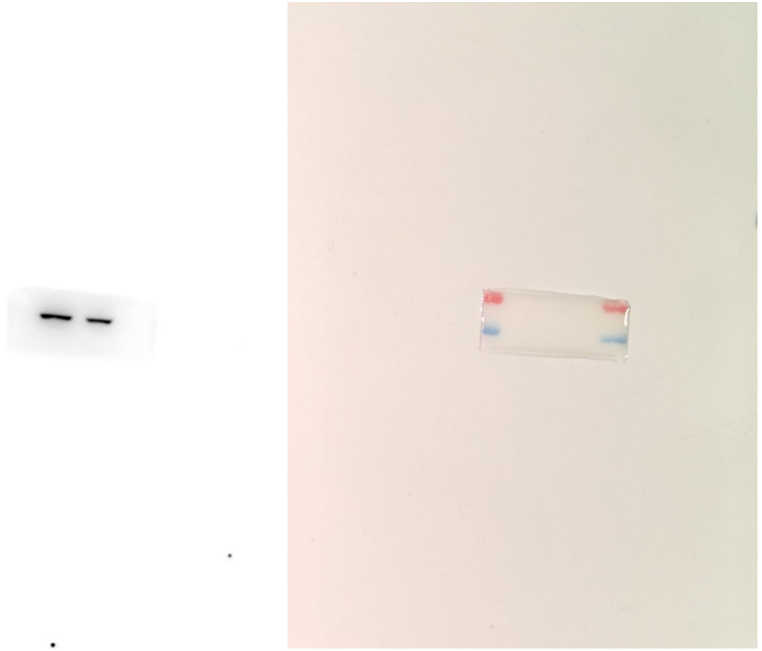

FOS

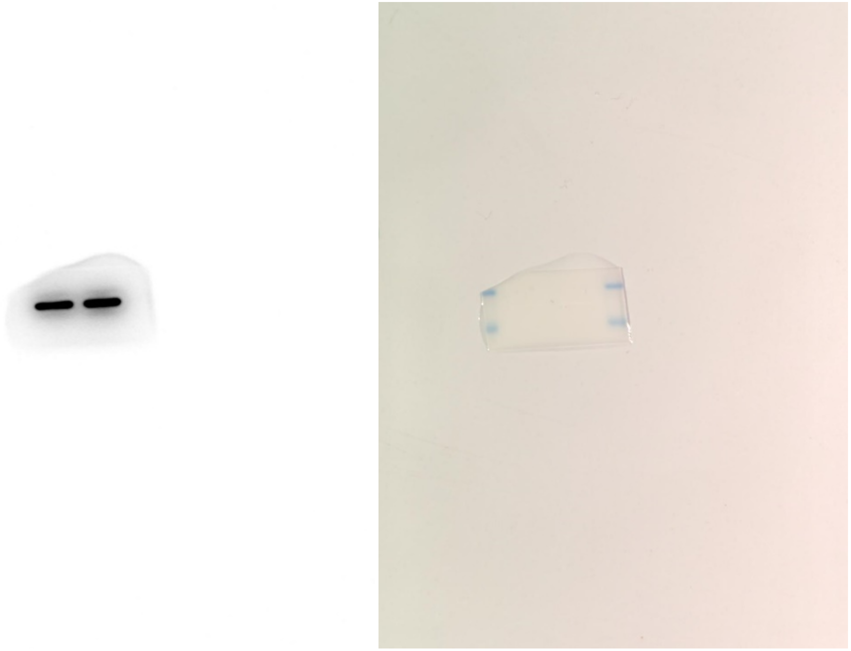

GAPDH

Figure 4C

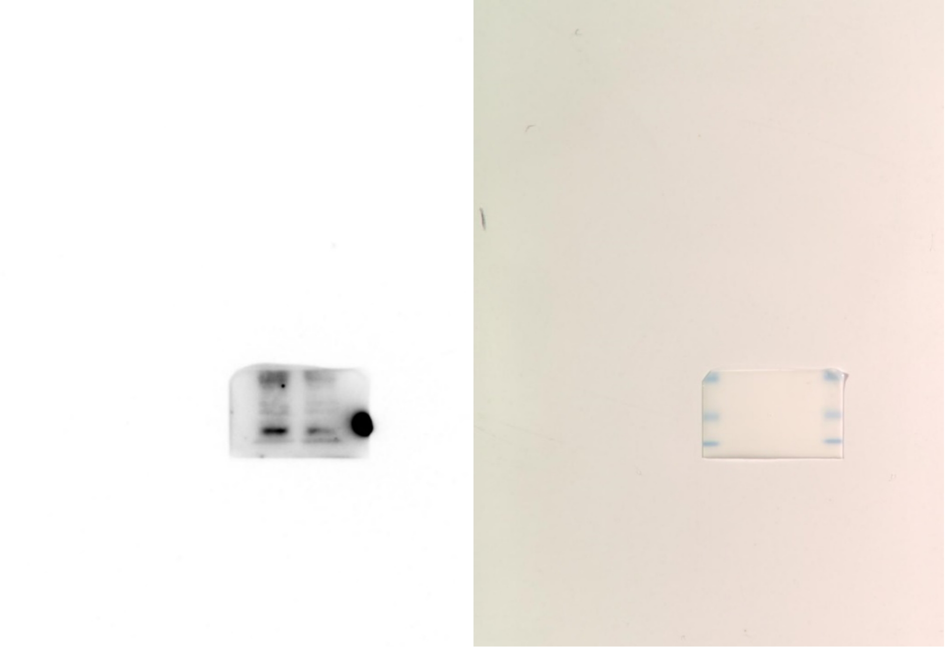

NRAS

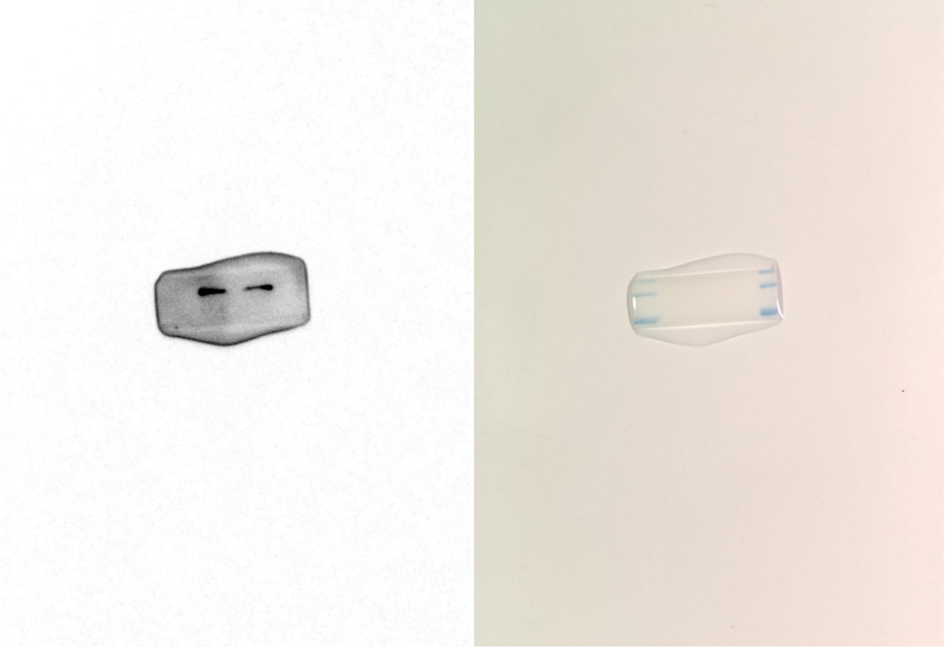

PIK3CB

Figure 4C

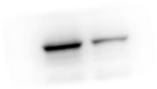

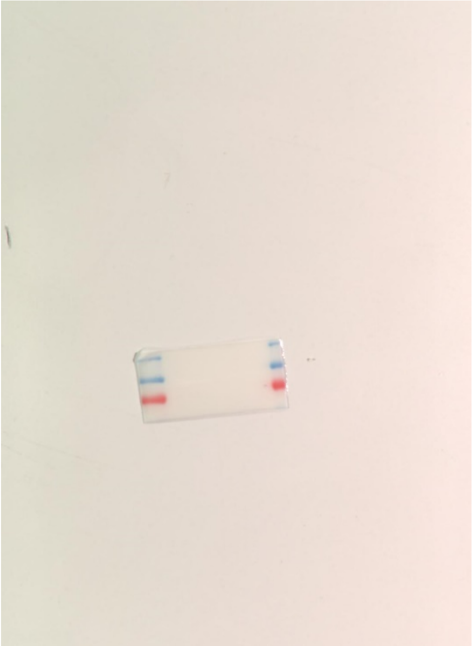

STAT1

Figure 4D and S7A

DU 145

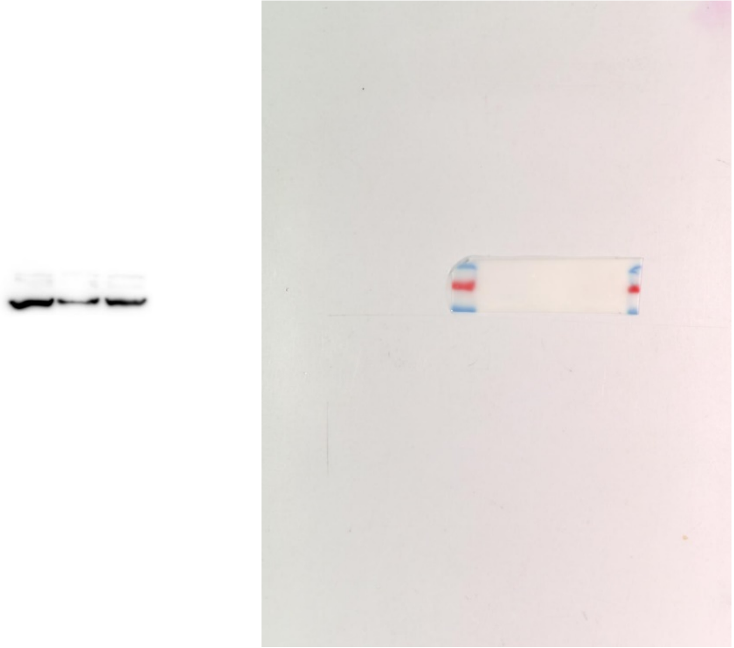

CDKL3

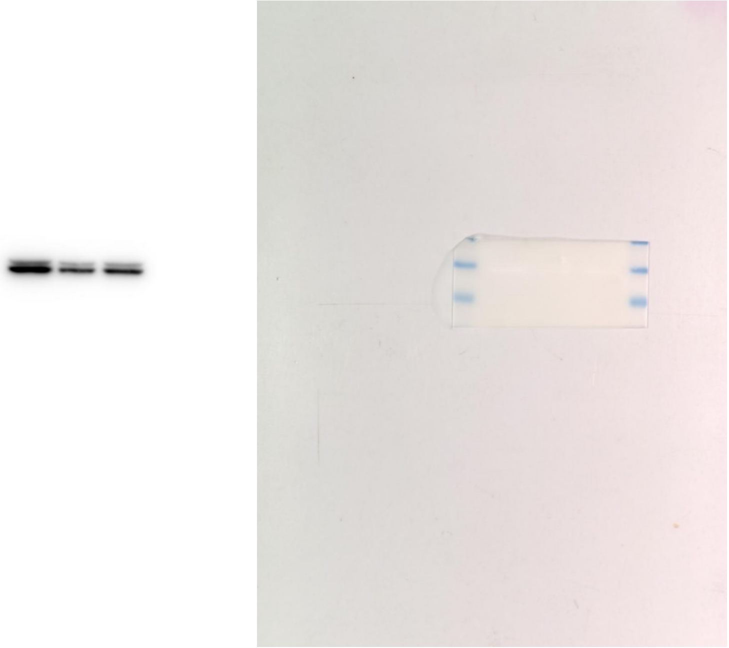

ERK

Figure 4D and S7A

DU 145

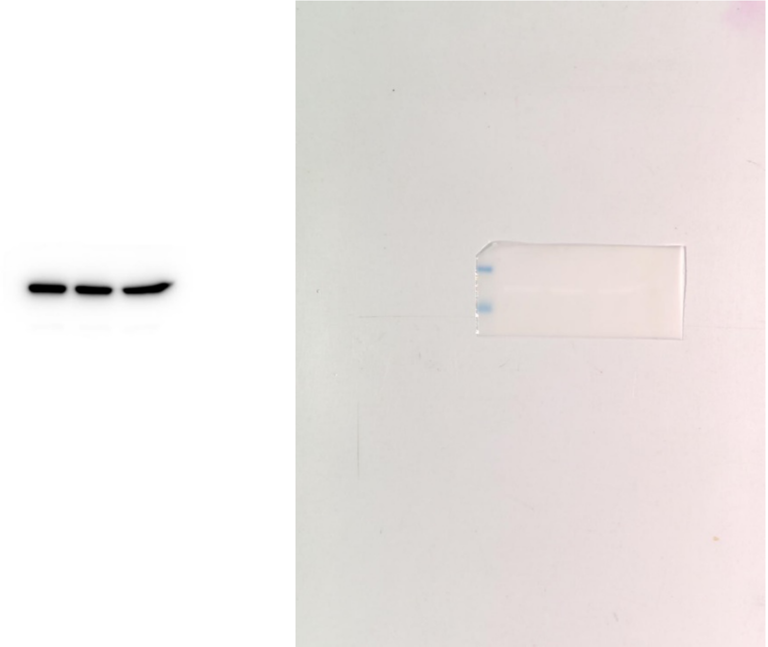

GAPDH

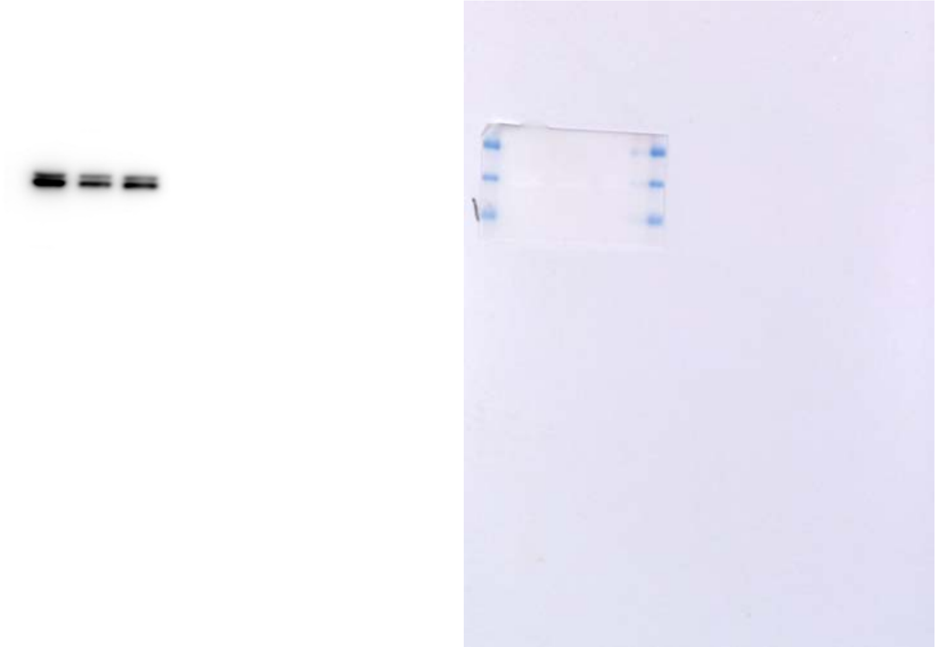

p-ERK

Figure 4D and S7A

DU 145

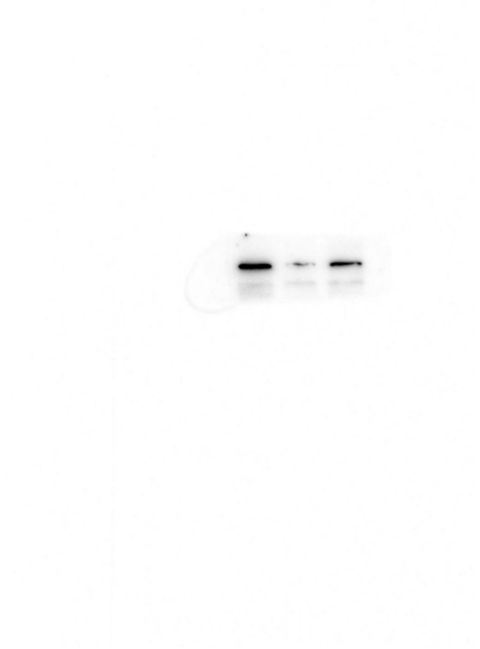

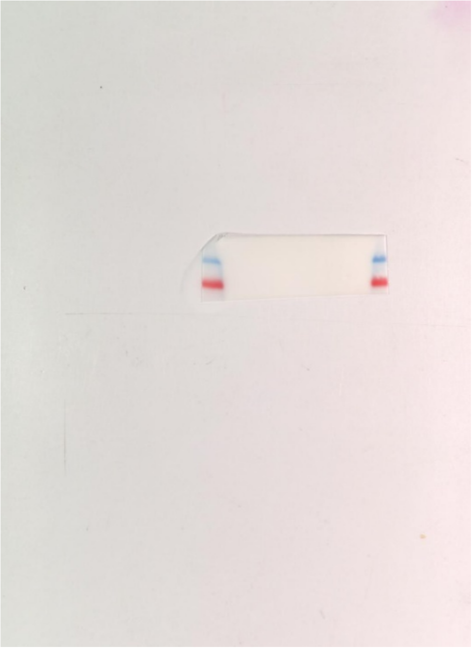

STAT1

Figure 4D and S7A

PC-3

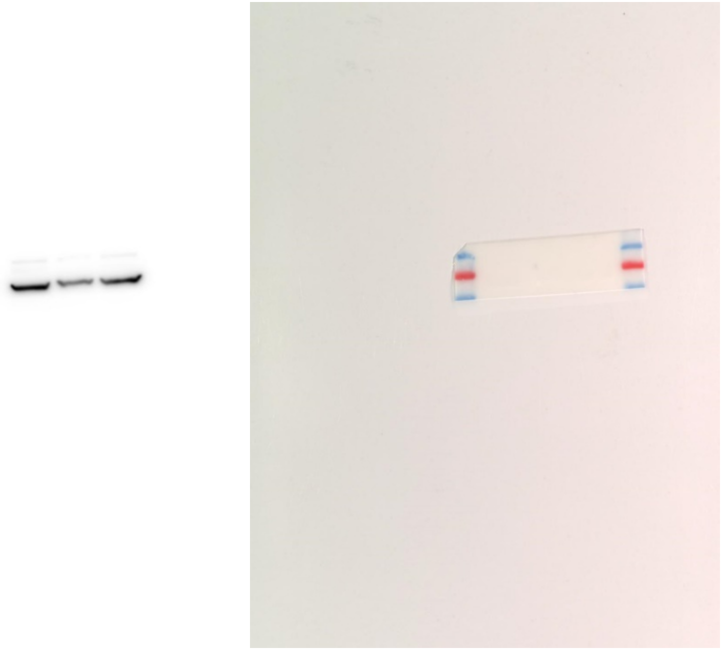

CDKL3

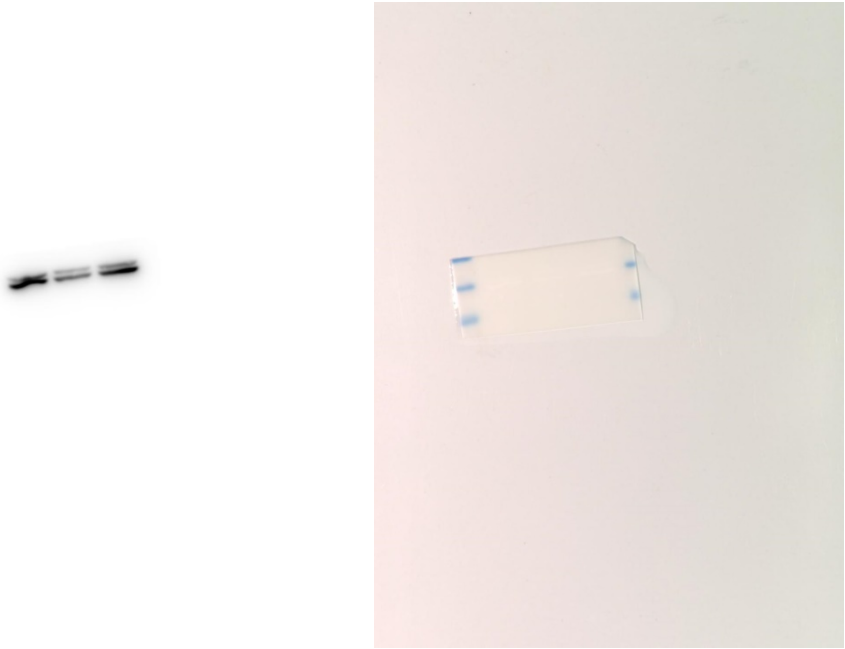

ERK

Figure 4D and S7A

PC-3

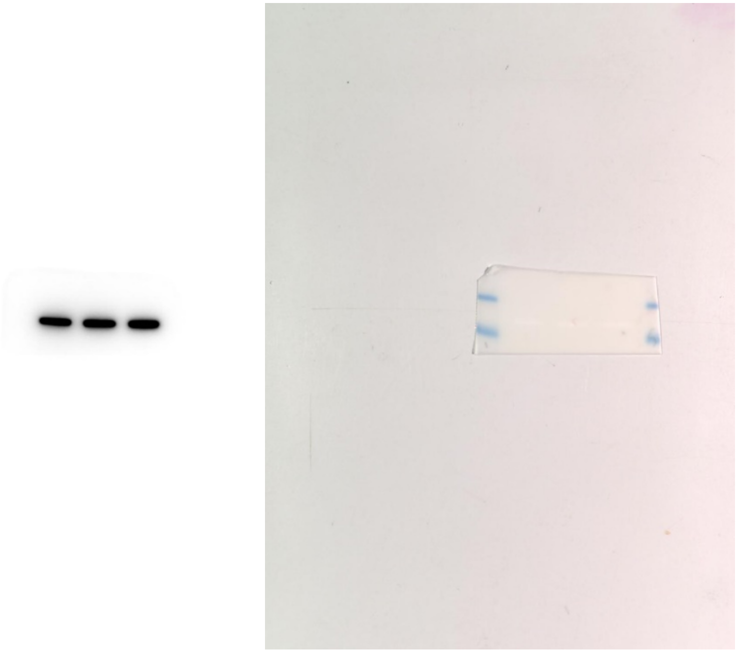

GAPDH

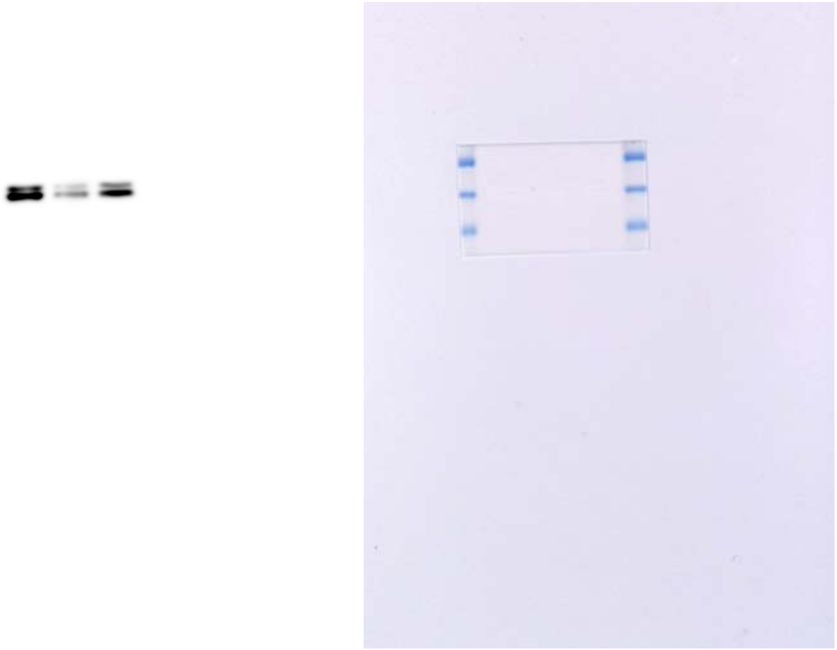

p-ERK

Figure 4D and S7A

PC-3

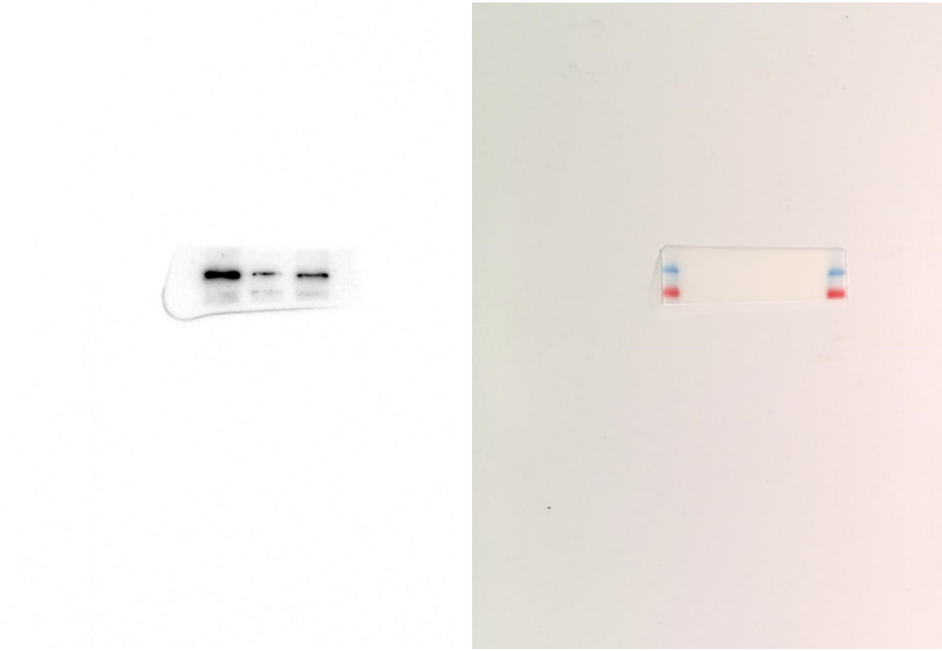

STAT1

Figure 5A DU 145

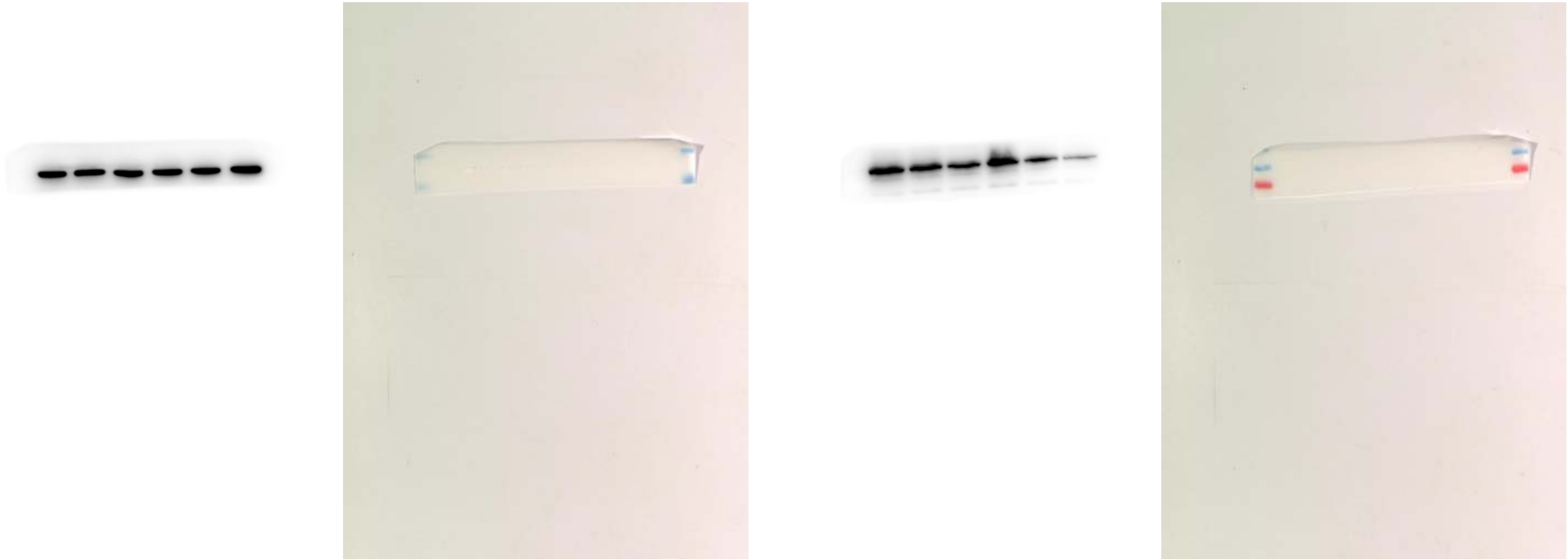

GAPDH STAT1

Figure 5A PC-3

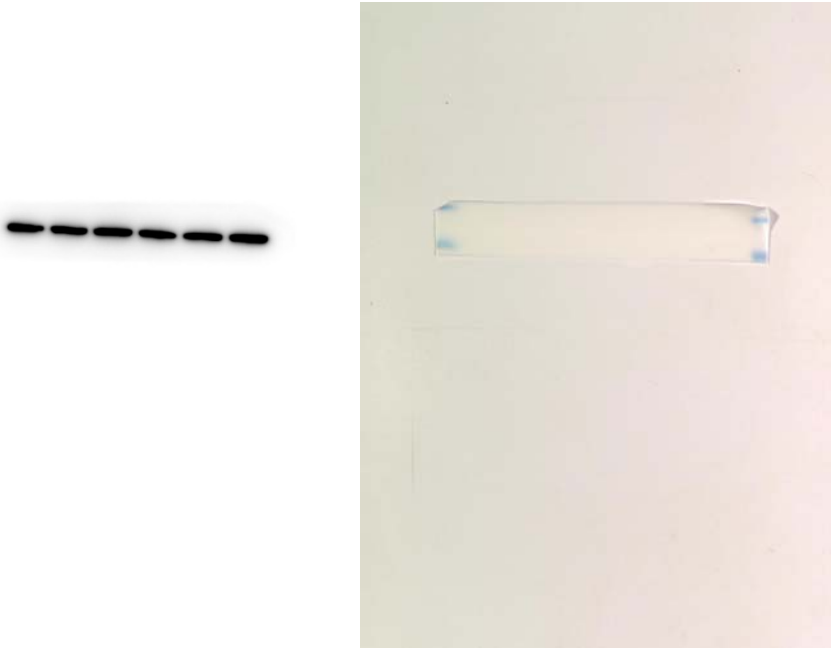

GAPDH

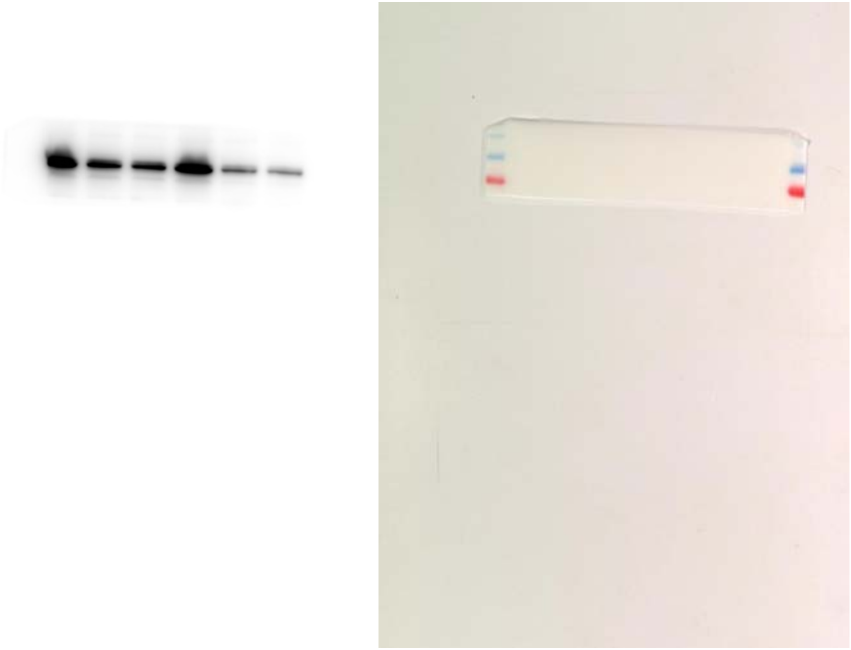

STAT1

Figure 5B DU 145

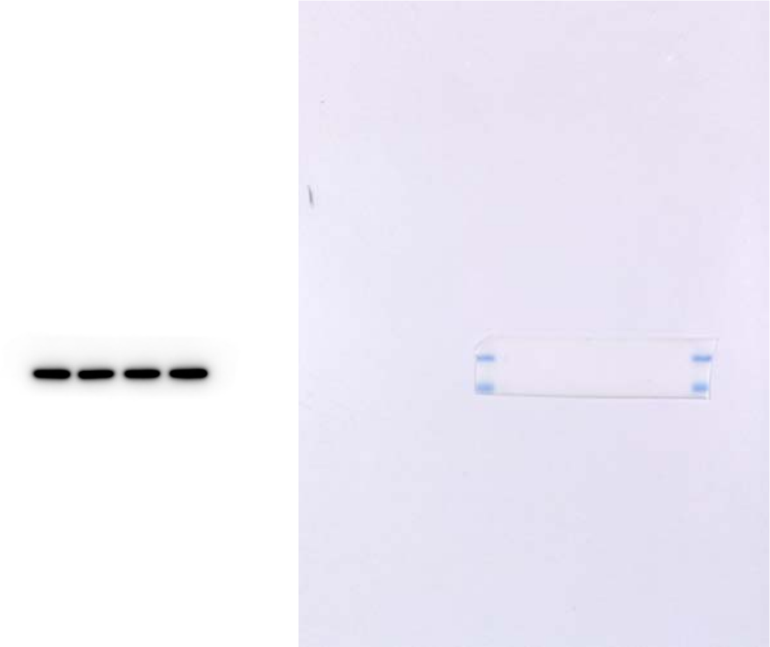

GAPDH

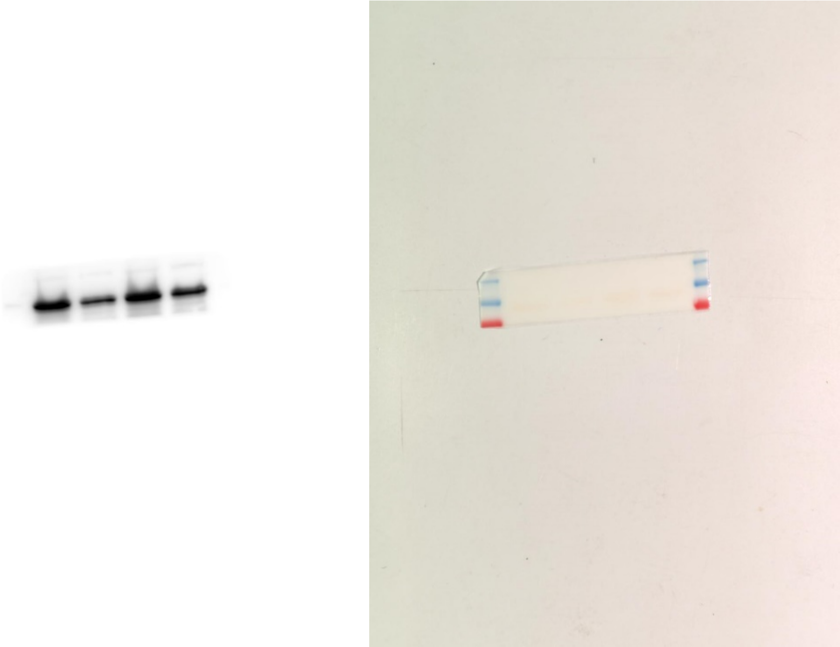

STAT1

Figure 5B PC-3

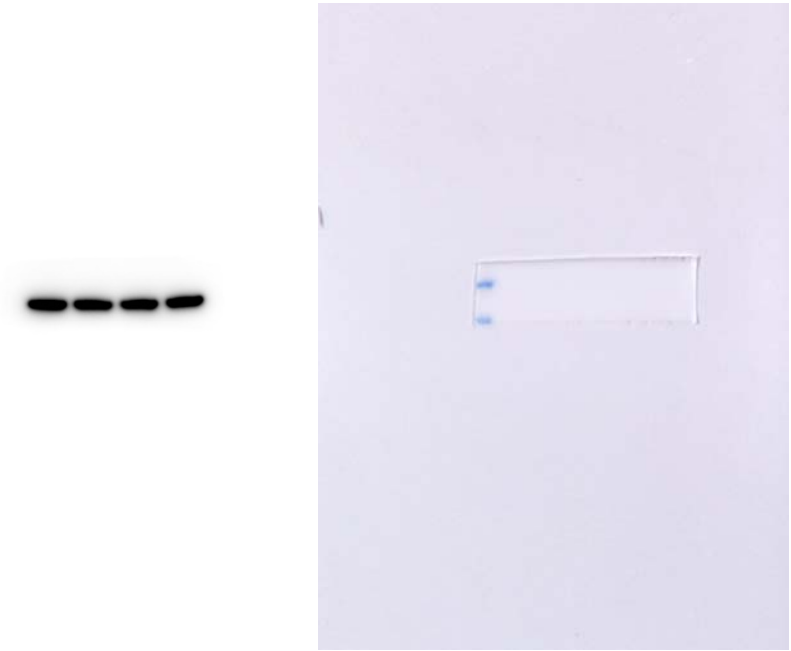

GAPDH

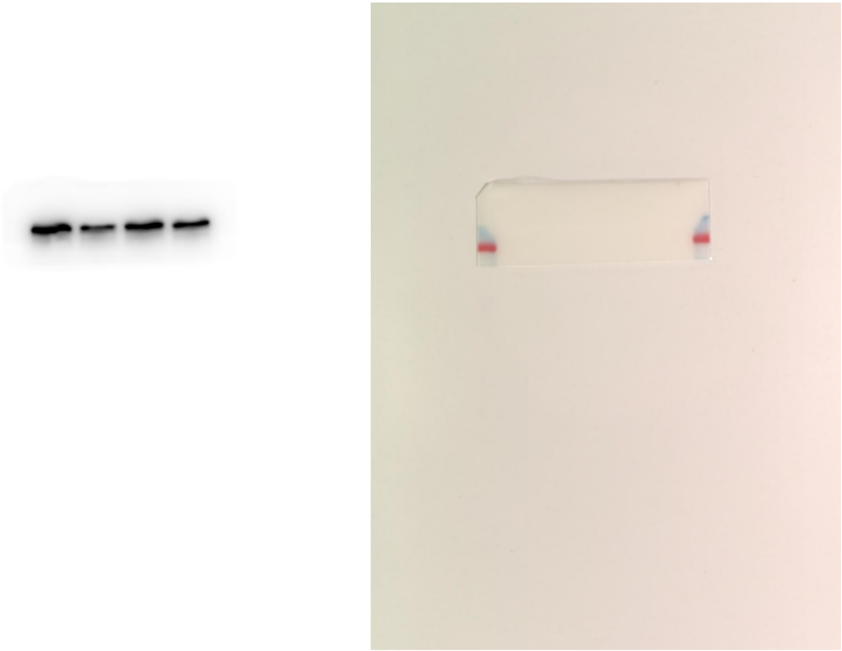

STAT1

Figure 5C Input

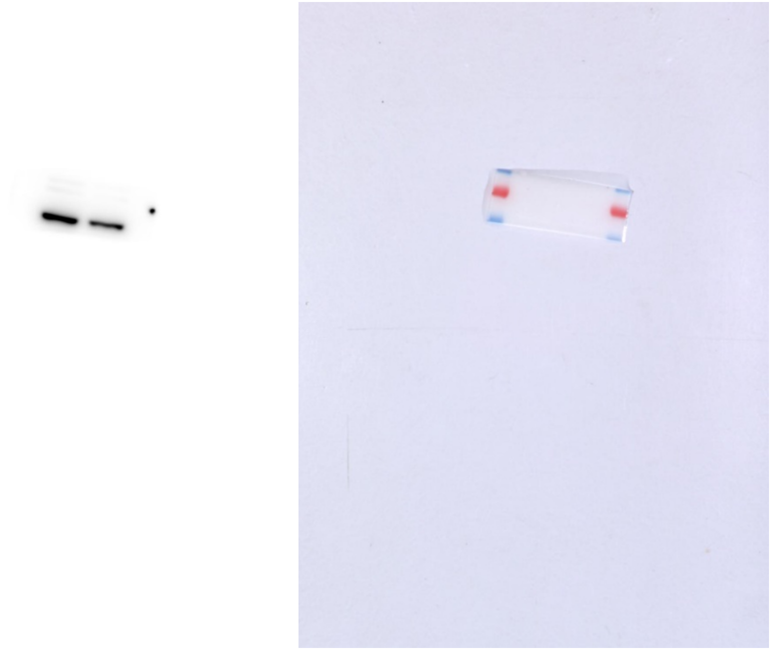

CDKL3

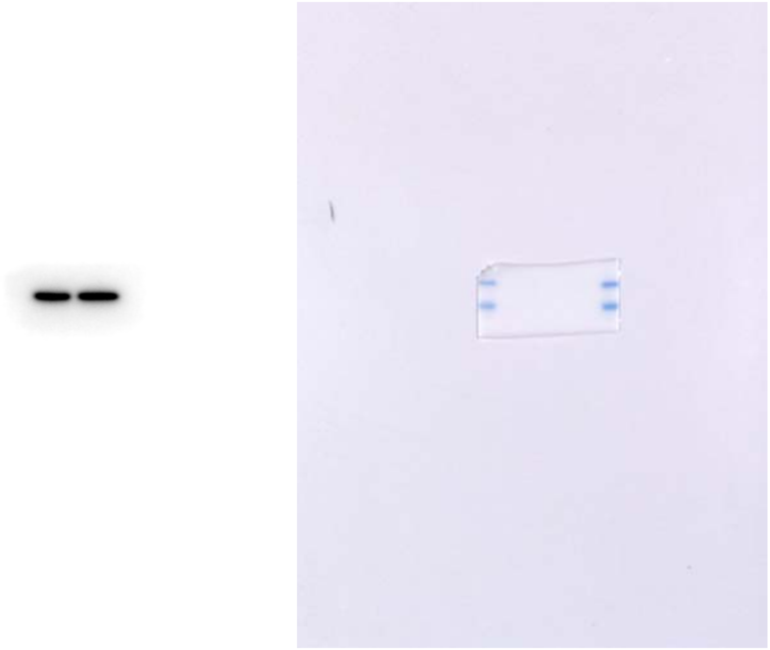

GAPDH

Figure 5C IP STAT1

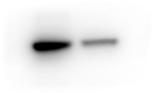

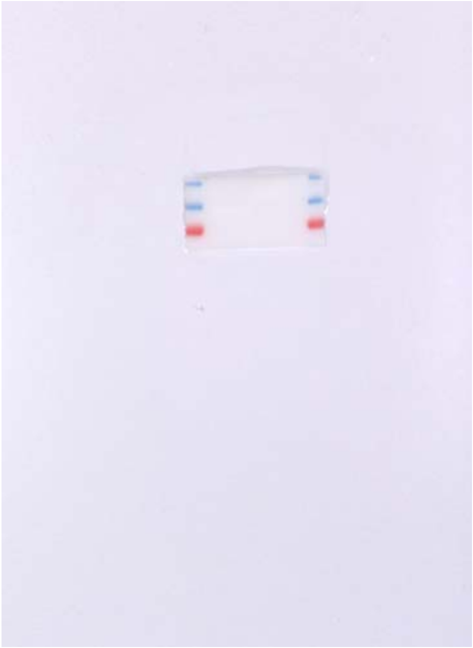

STAT1

Figure 5C IP UB

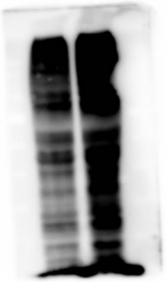

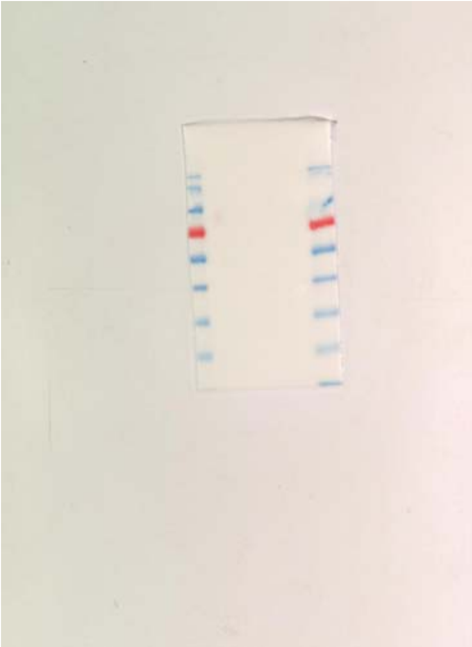

Ubiquitin

Figure 5D Input

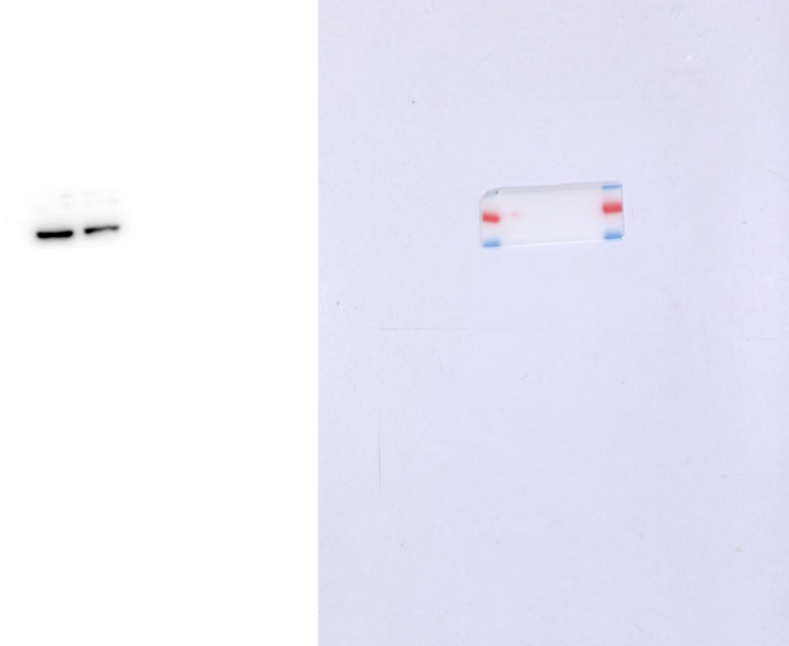

CDKL3

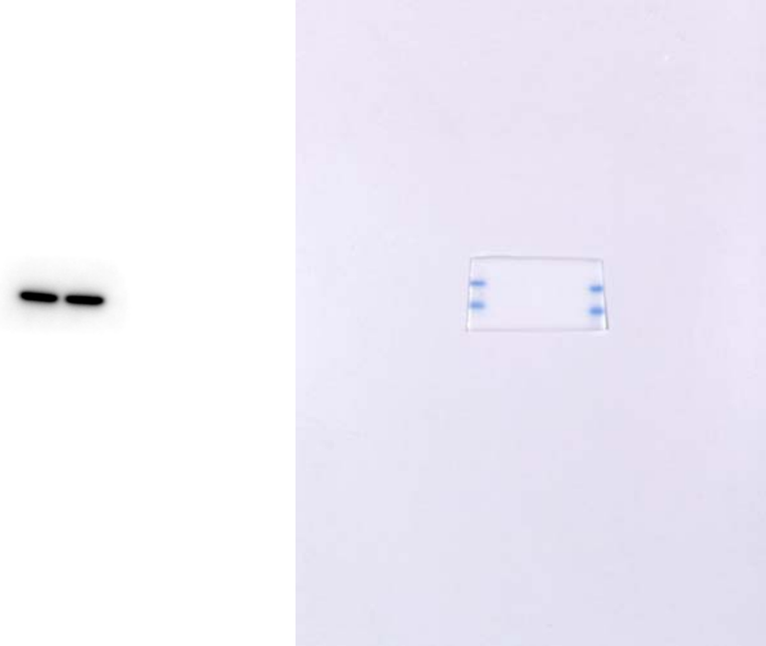

GAPDH

Figure 5D

IP STAT1

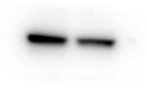

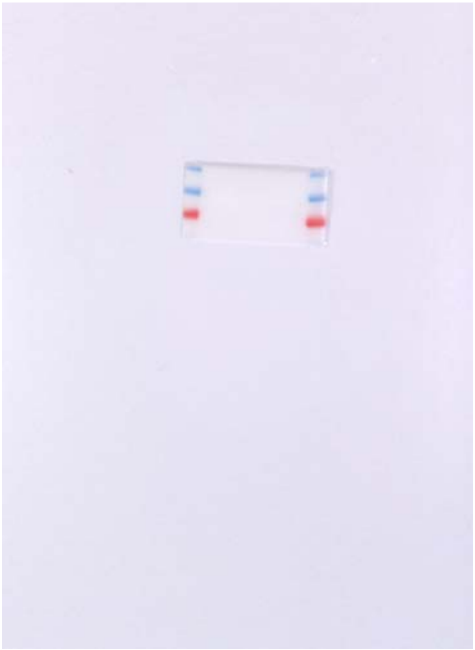

STAT1

Figure 5D IP UB

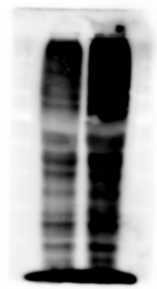

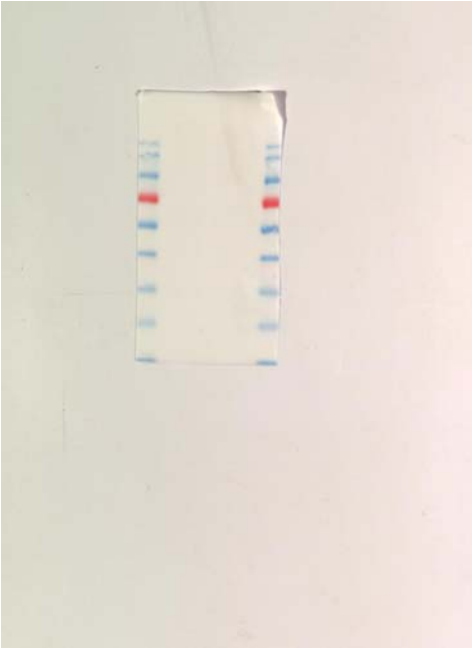

Ubiquitin

Figure 5E DU 145

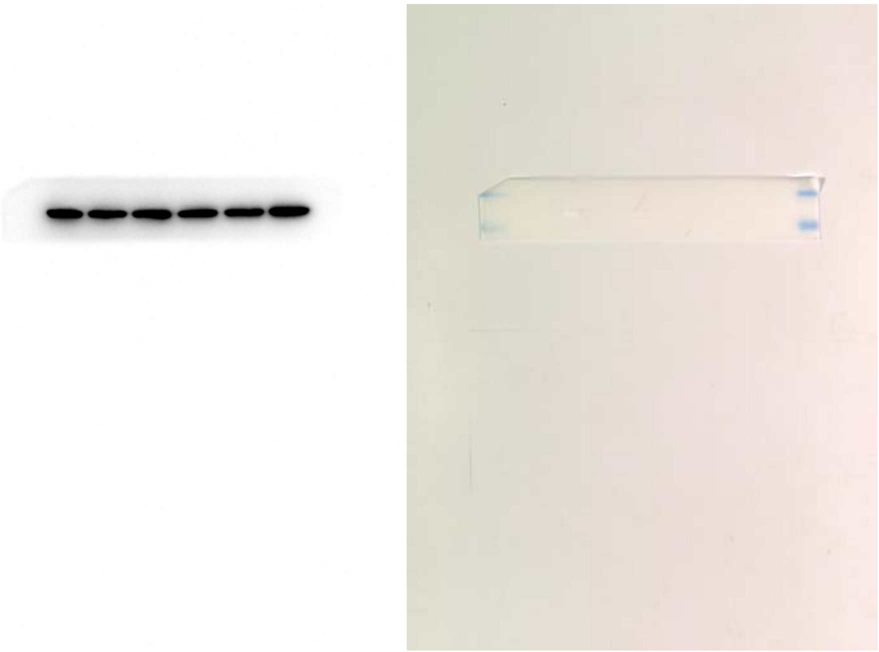

GAPDH

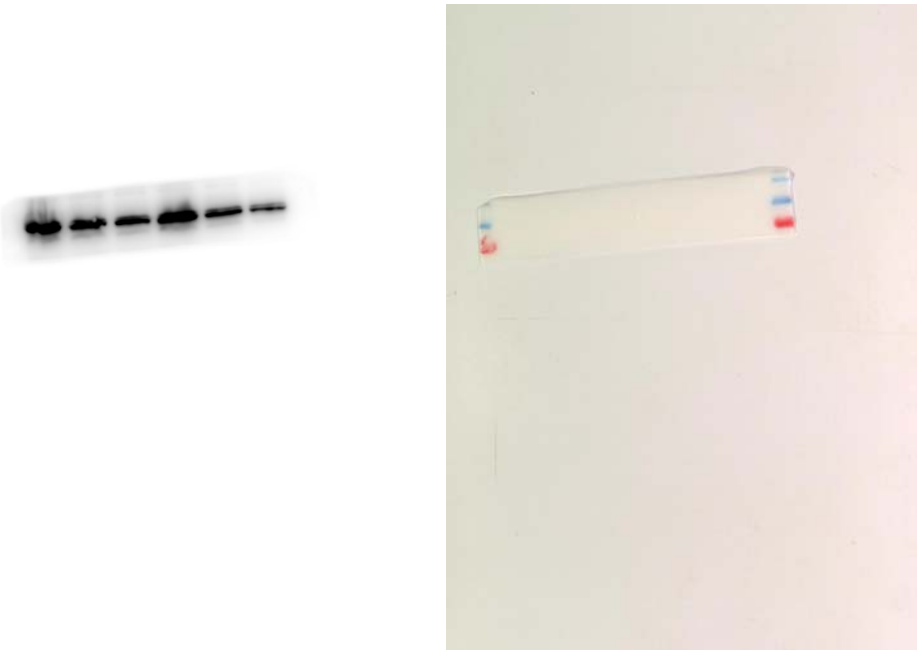

STAT1

Figure 5E PC-3

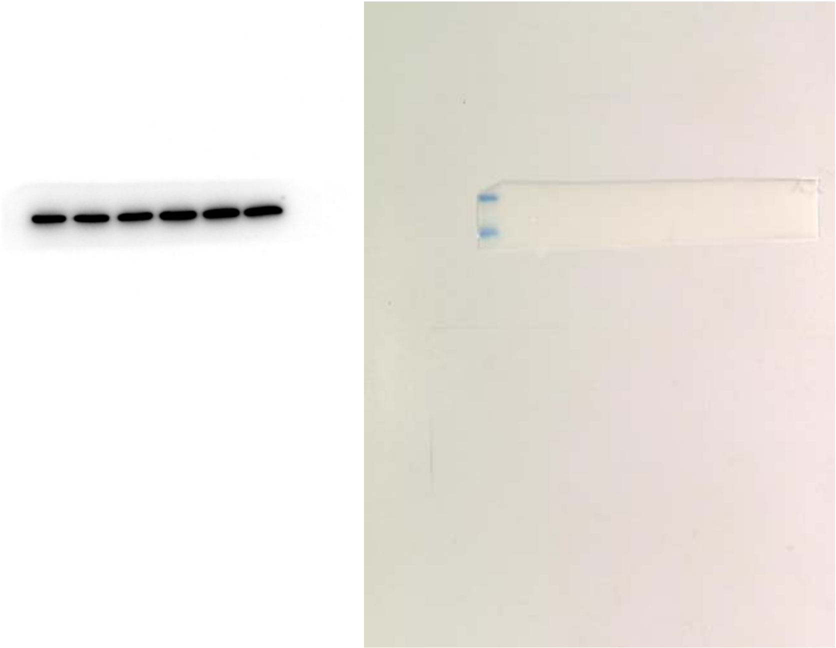

GAPDH

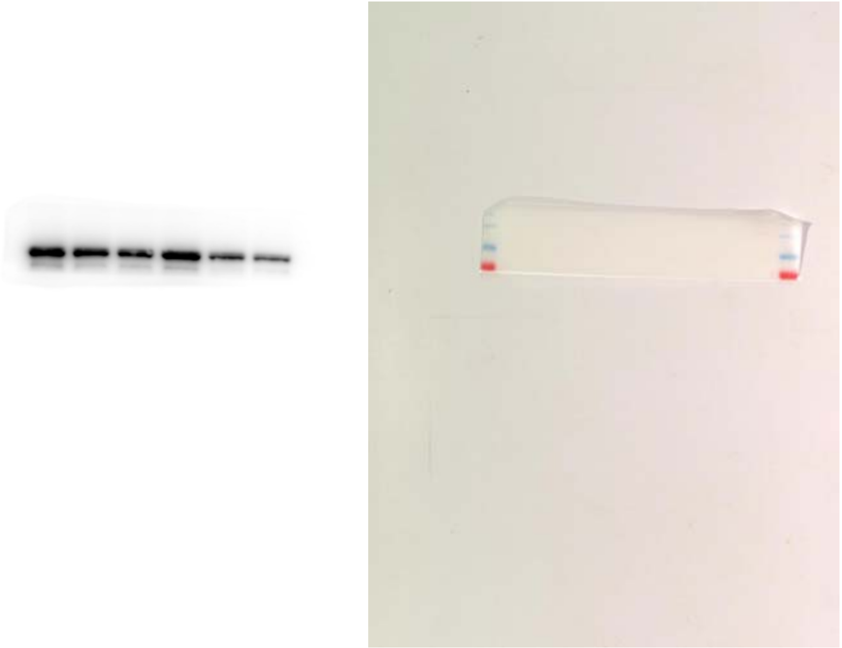

STAT1

Figure 5F DU 145

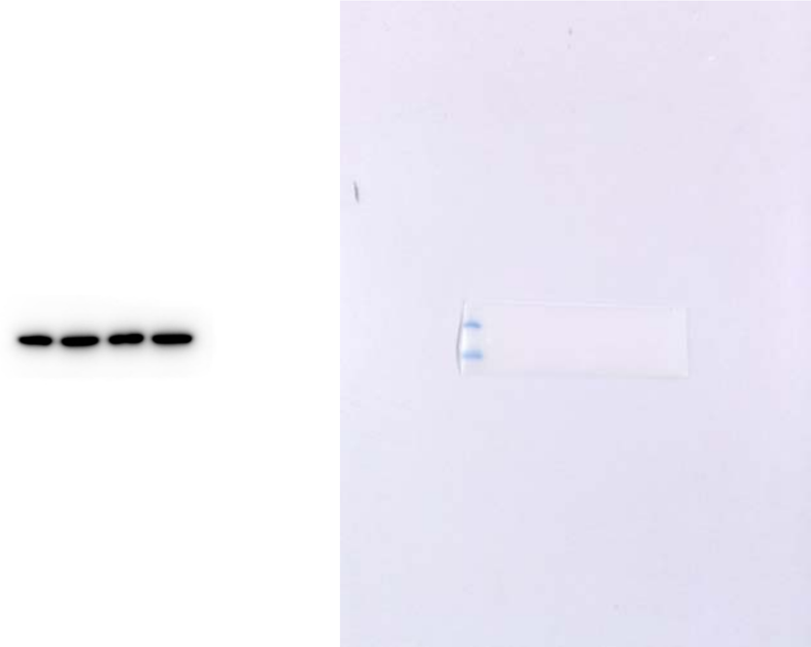

GAPDH

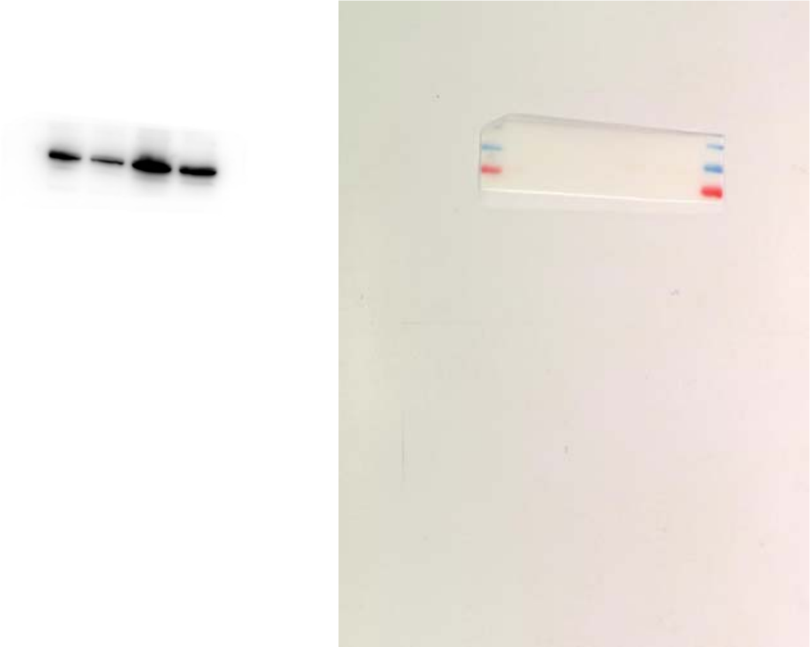

STAT1

Figure 5F PC-3

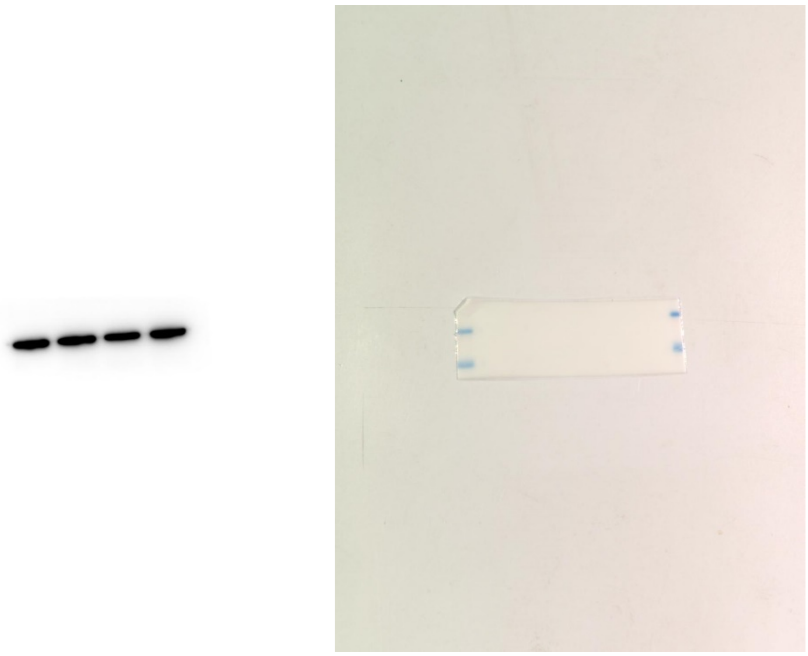

GAPDH

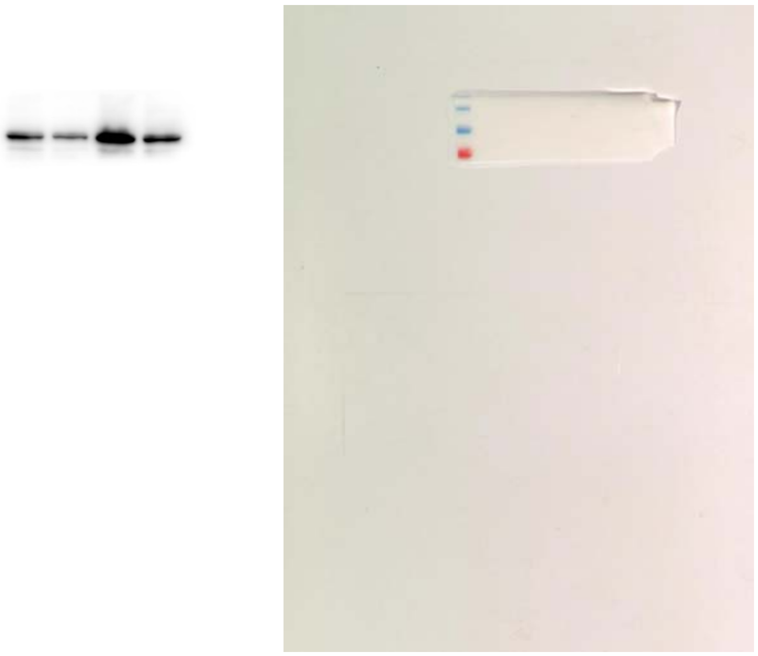

STAT1

Figure 5G Input

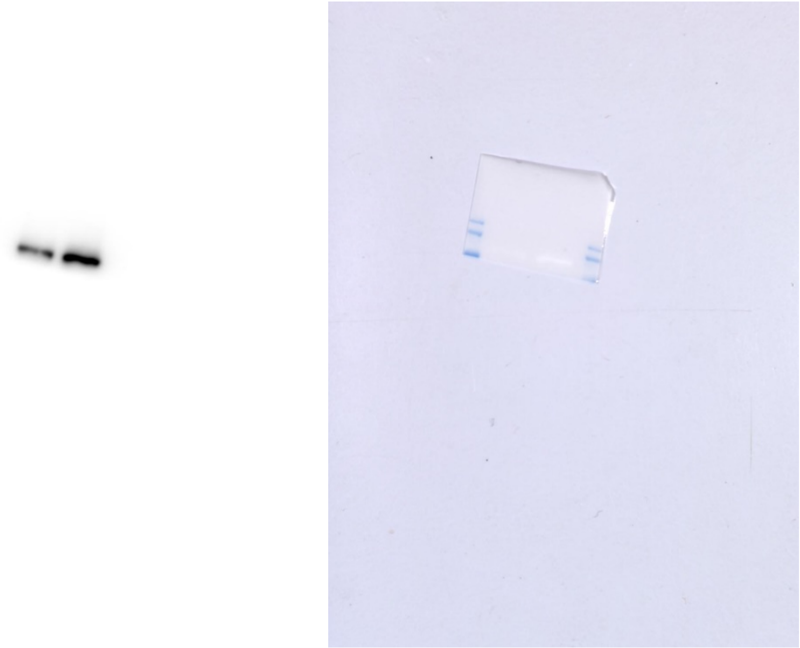

CBL

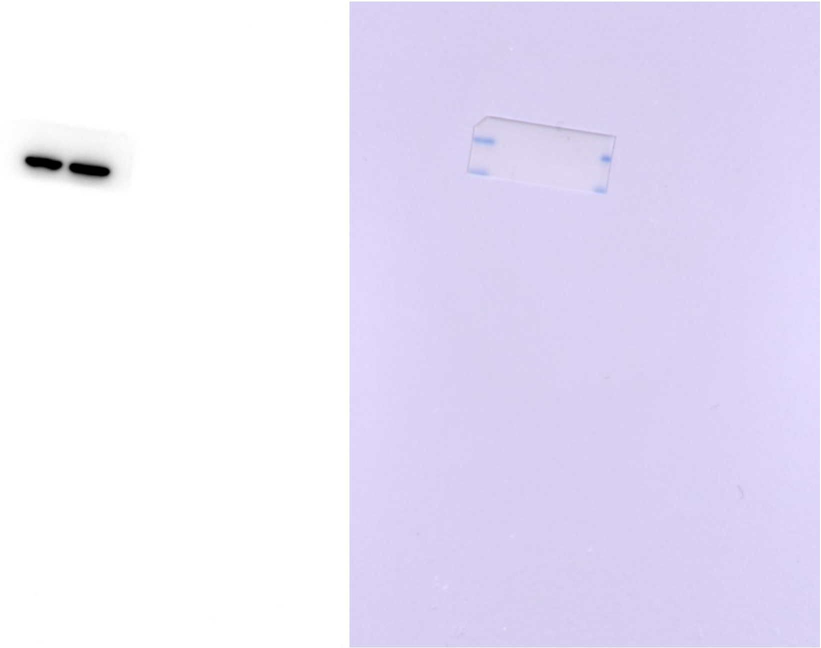

GAPDH

Figure 5G IP STAT1

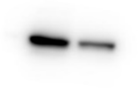

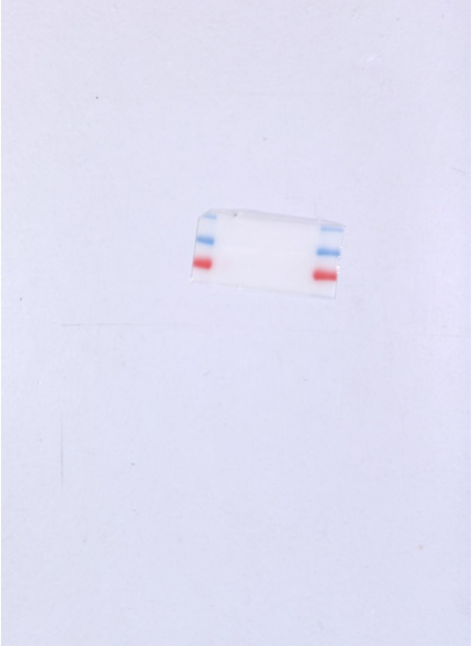

STAT1

Figure 5G IP UB

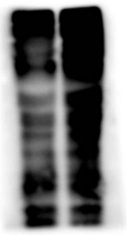

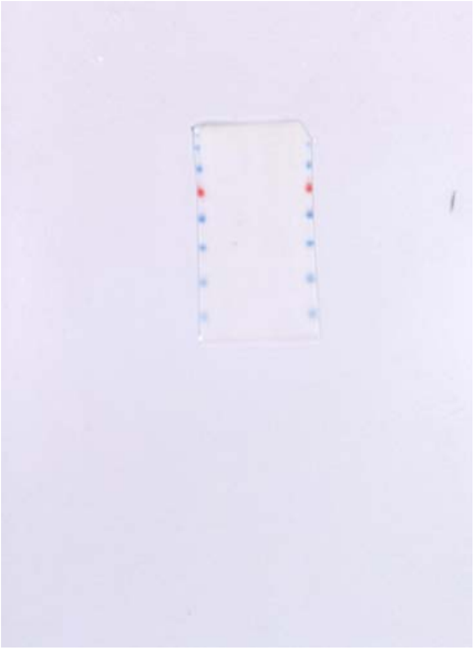

Ubiquitin

Figure 5H Input

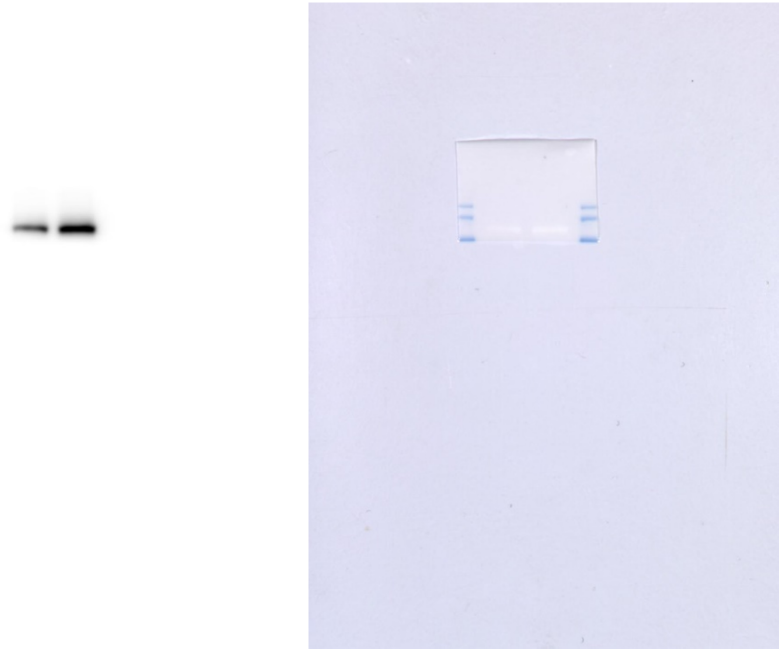

CBL

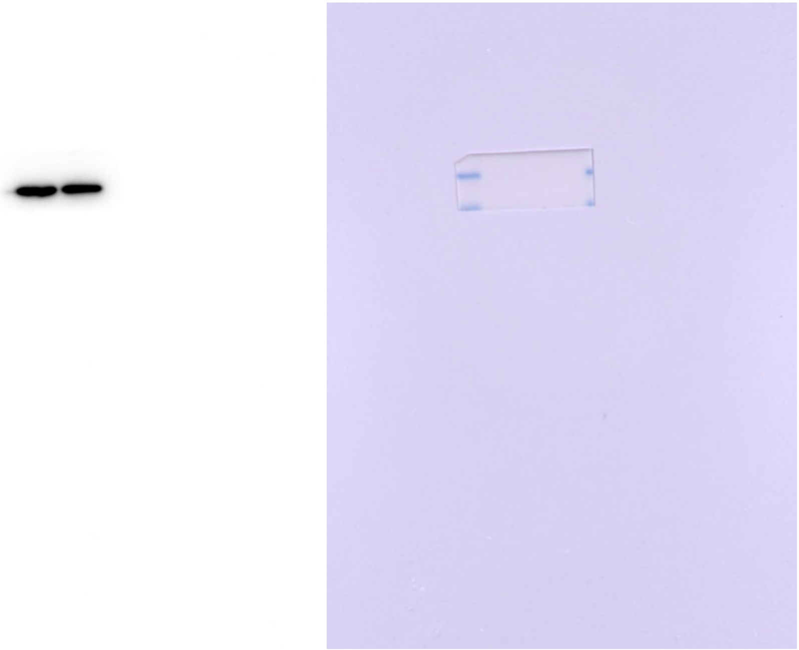

GAPDH

Figure 5H IP STAT1

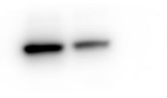

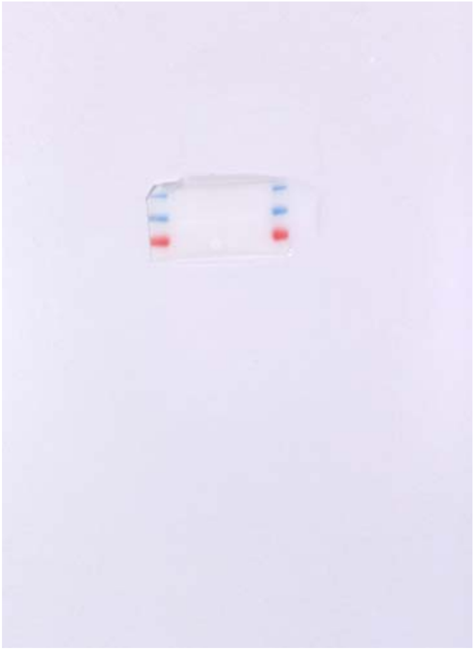

STAT1

Figure 5H IP UB

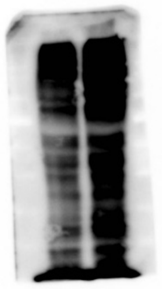

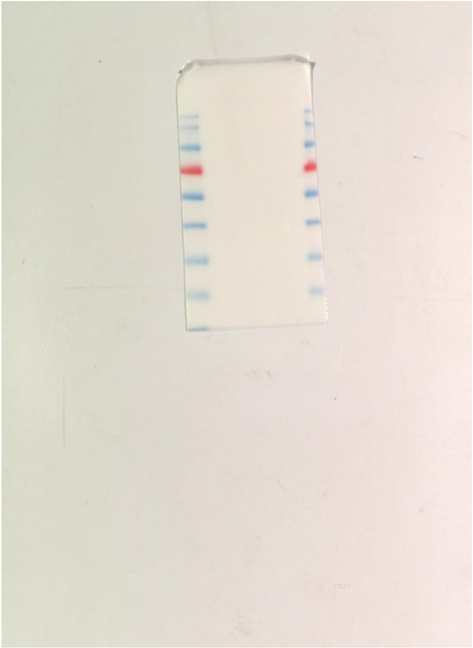

Ubiquitin

Figure S4A

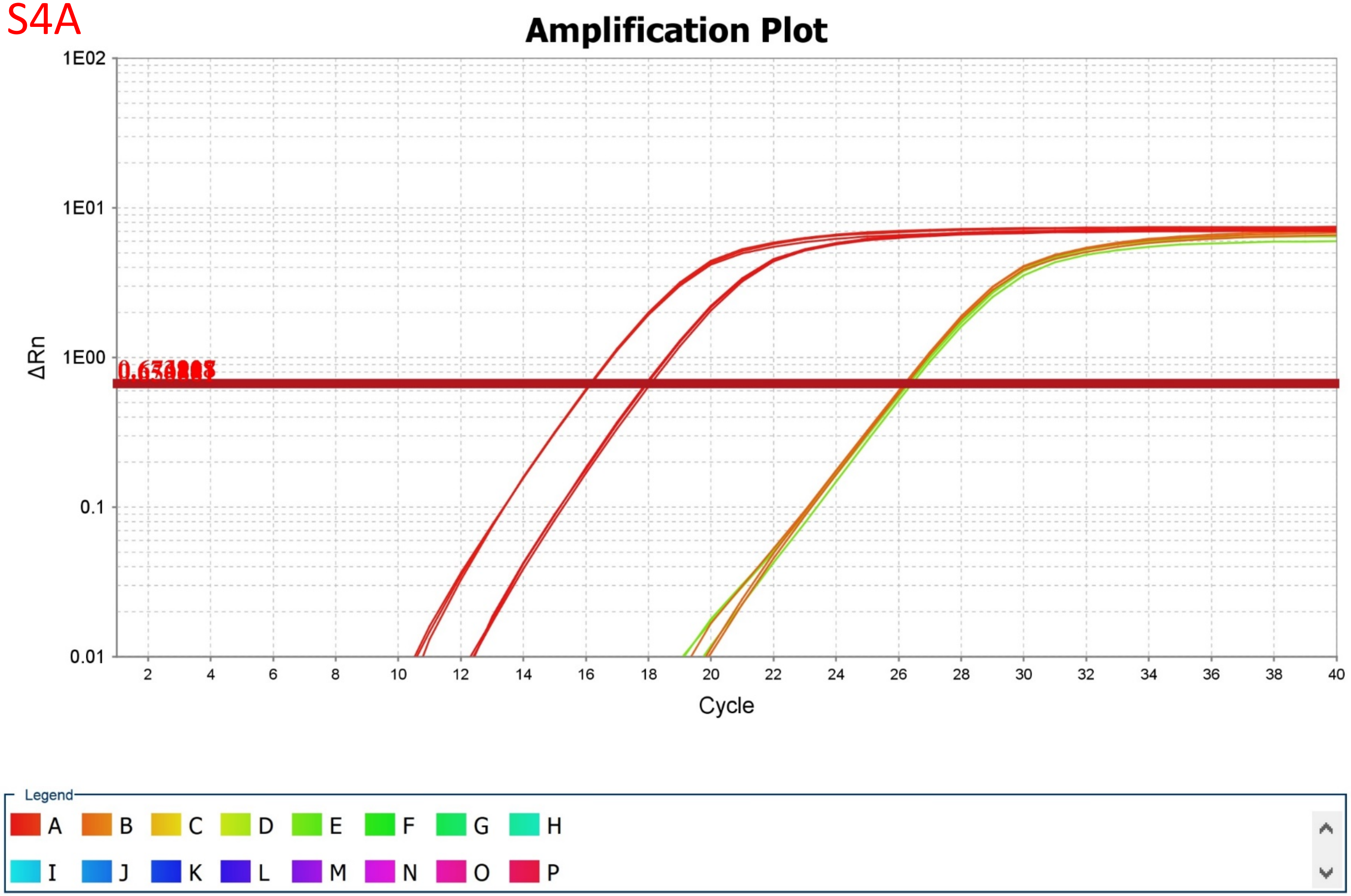

Amplification Plot
1E02
1E01
1E00
0.1
0.01
ΔRn
2
4
6
8
10
12
14
16
18
20
22
24
26
28
30
32
34
36
38
40
Cycle
Legend
A
B
C
D
E
F
G
H
I
J
K
L
M
N
O
P

Figure S4A

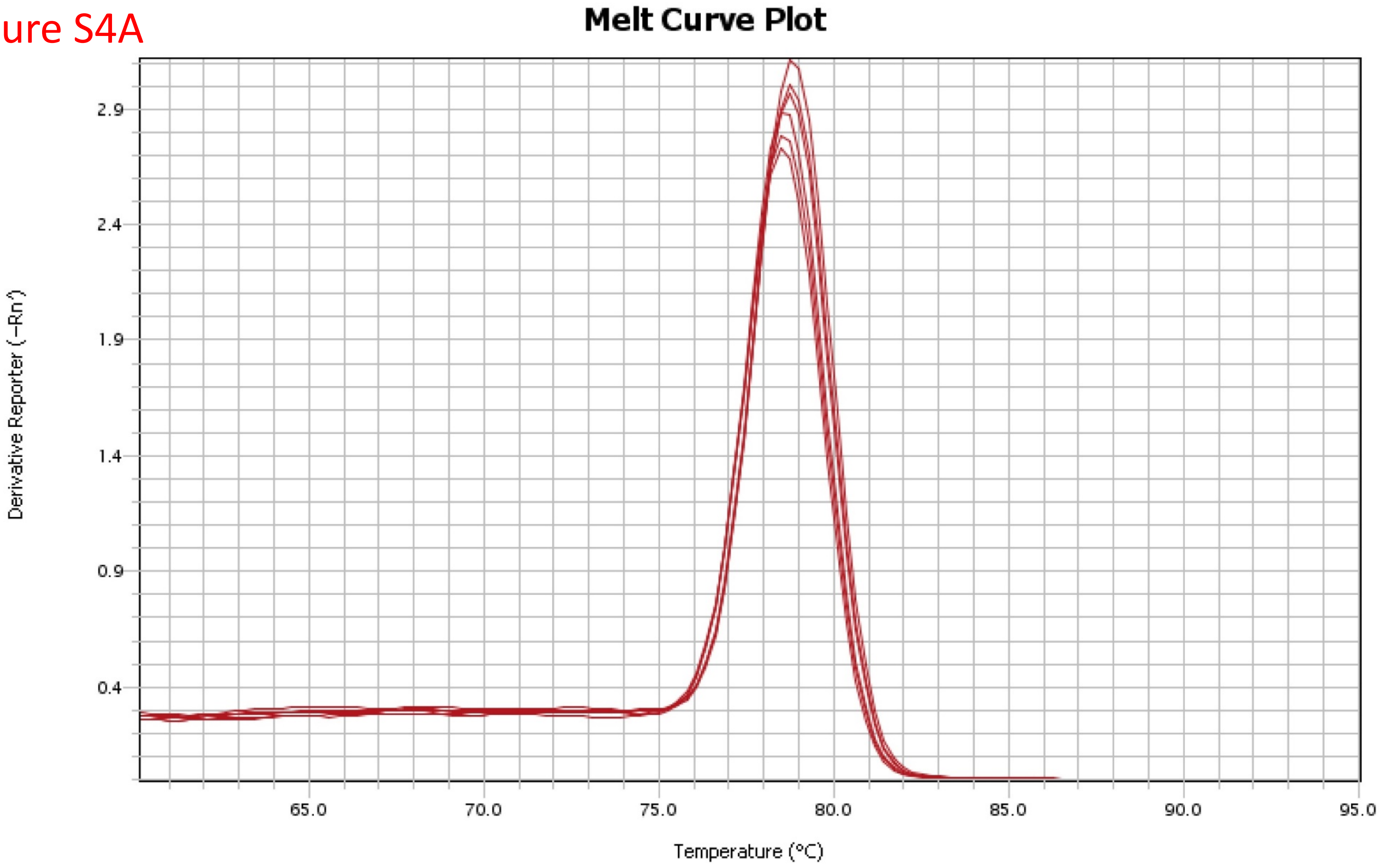
Melt Curve Plot
Derivative Reporter (−Rn')
2.9
2.4
1.9
1.4
0.9
0.4
65.0
70.0
75.0
80.0
85.0
90.0
95.0
Temperature (°C)

Figure S4A

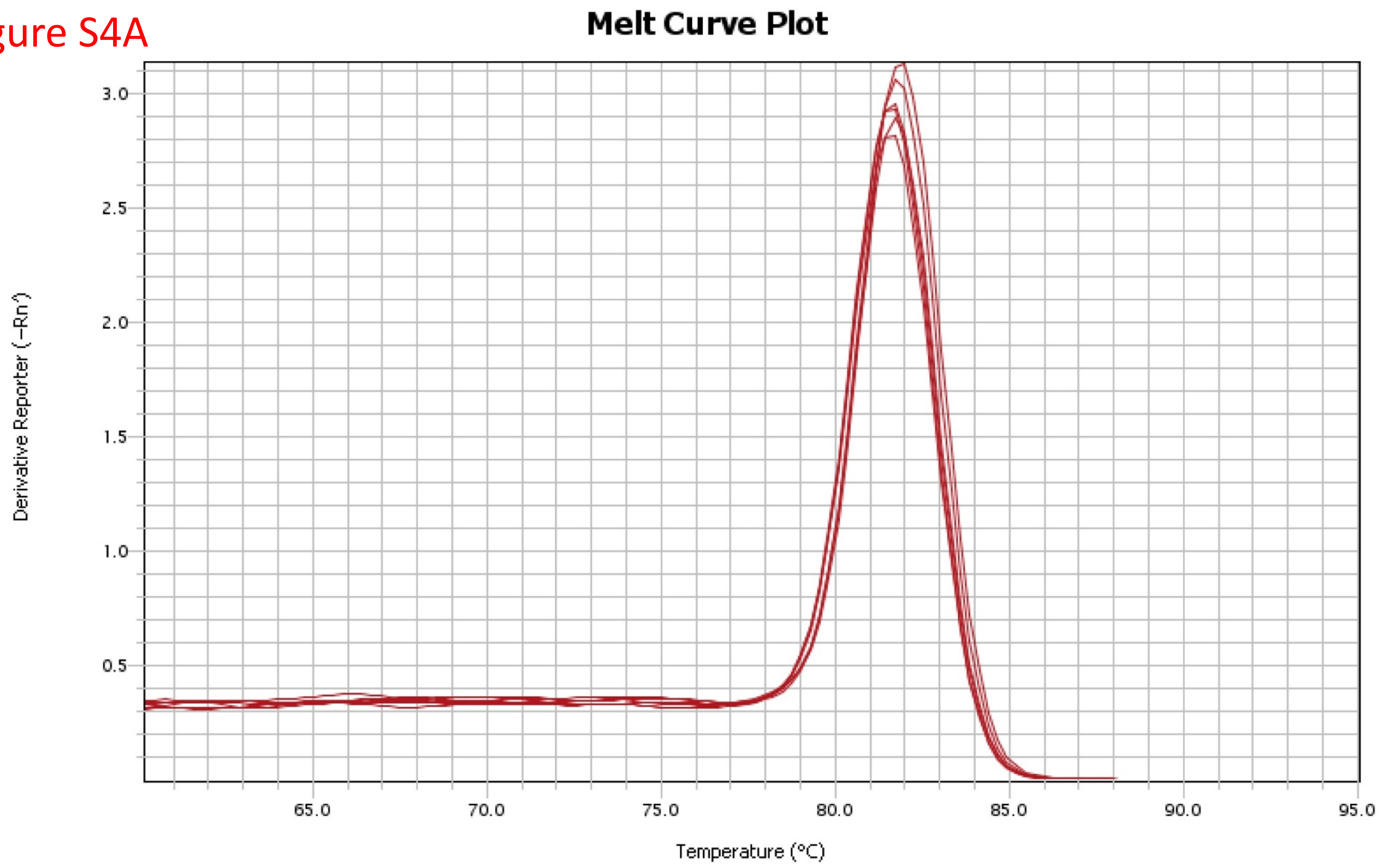

Melt Curve Plot
3.0
2.5
2.0
1.5
1.0
0.5
Derivative Reporter (−Rn')
65.0
70.0
75.0
80.0
85.0
90.0
95.0
Temperature (°C)

Figure S4A

Block Type	384-Well Block
Calibration E	No
Calibration E	2022-08-01 16:00:17 PM CST
Calibration F	No
Calibration F	2022-08-01 16:39:53 PM CST
Calibration I	No
Calibration I	2022-08-01 16:56:18 PM CST
Calibration F	No
Calibration F	2022-08-01 15:53:01 PM CST
Calibration F	No
Calibration F	2022-08-01 17:25:54 PM CST
Calibration S	No
Calibration S	2022-08-01 17:03:33 PM CST
Calibration T	No
Calibration T	2022-08-01 17:10:48 PM CST
Calibration U	No
Calibration U	2022-08-01 16:16:55 PM CST
Calibration V	No
Calibration V	2022-08-01 16:48:55 PM CST
Chemistry	SYBR_GREEN
Experiment	Barcode
Experiment	Comments
Experiment	C:\Users\zhouch\Desktop\2022-12-14 2.eds
Experiment	2022-12-14 2
Experiment	2022-12-14 18:04:19 PM CST
Experiment	Comparative Cт (ΔΔCт)
Experiment	User Name
Instrument N	278882256
Instrument S	278882256
Instrument T	ViiA 7
Passive Ref	ROX
Quantificatio	Ct
Signal Smoo	true
Stage/ Cycl	Stage 2, Step 2

Well	Well Positio	Omit	Sample Nar	Target Nam	Task	Reporter	Quencher	Quantity	Quantity Me	Quantity SD	RQ	RQ Min	RQ Max	CT	Ct Mean	Ct SD	Delta Ct
1	A1	FALSE	LNcap shCt	H-GAPDH	UNKNOWN	SYBR	None							17.904			
2	A2	FALSE	LNcap shCt	H-GAPDH	UNKNOWN	SYBR	None							17.860			
3	A3	FALSE	LNcap shCt	H-GAPDH	UNKNOWN	SYBR	None							17.994			
25	B1	FALSE	LNcap shCt	H-CDKL3	UNKNOWN	SYBR	None							26.235			
26	B2	FALSE	LNcap shCt	H-CDKL3	UNKNOWN	SYBR	None							26.176			
27	B3	FALSE	LNcap shCt	H-CDKL3	UNKNOWN	SYBR	None							26.174			
4	A4	FALSE	LNcap shCD	H-GAPDH	UNKNOWN	SYBR	None							16.093			
5	A5	FALSE	LNcap shCD	H-GAPDH	UNKNOWN	SYBR	None							16.096			
6	A6	FALSE	LNcap shCD	H-GAPDH	UNKNOWN	SYBR	None							16.061			
28	B4	FALSE	LNcap shCD	H-CDKL3	UNKNOWN	SYBR	None							26.411			
29	B5	FALSE	LNcap shCD	H-CDKL3	UNKNOWN	SYBR	None							26.310			
30	B6	FALSE	LNcap shCD	H-CDKL3	UNKNOWN	SYBR	None							26.231			

Analysis Ty	Singleplex
Endogenous	H-HSP27
RQ Min/Max	95.0
Reference S	Sample 1

Figure S4A

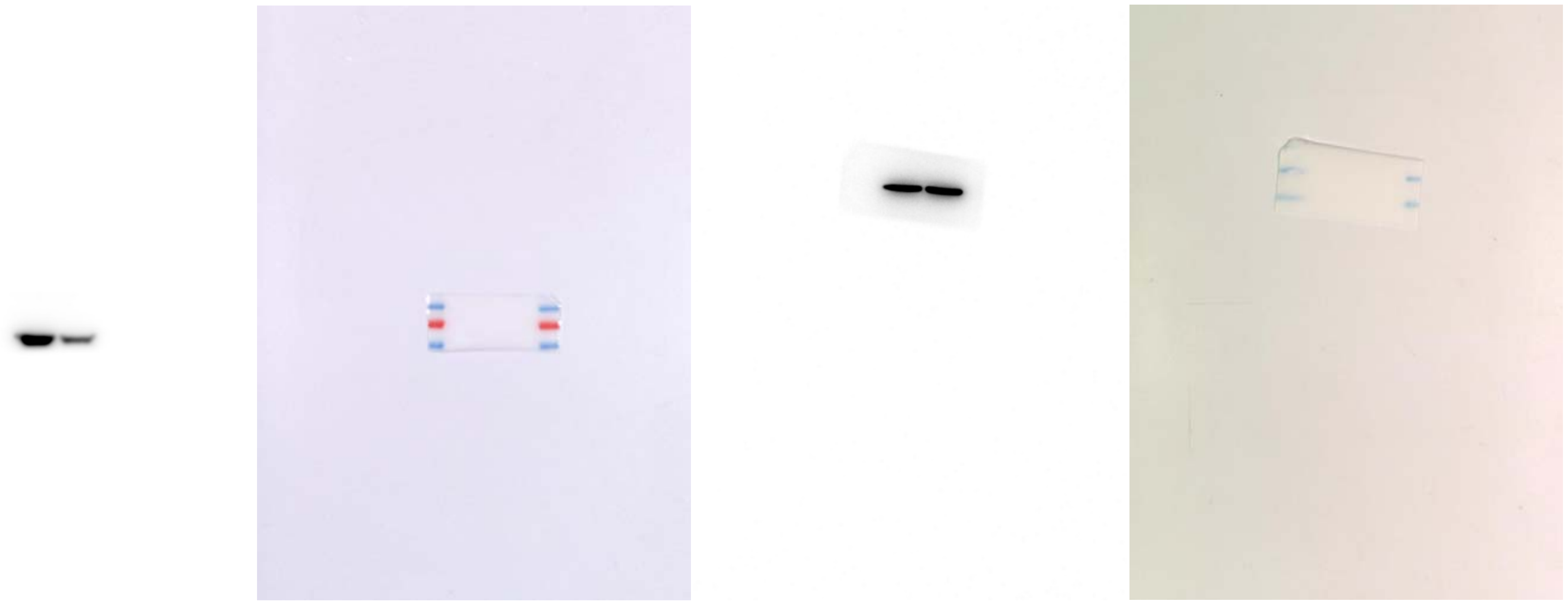

CDKL3

GAPDH

Figure S4C

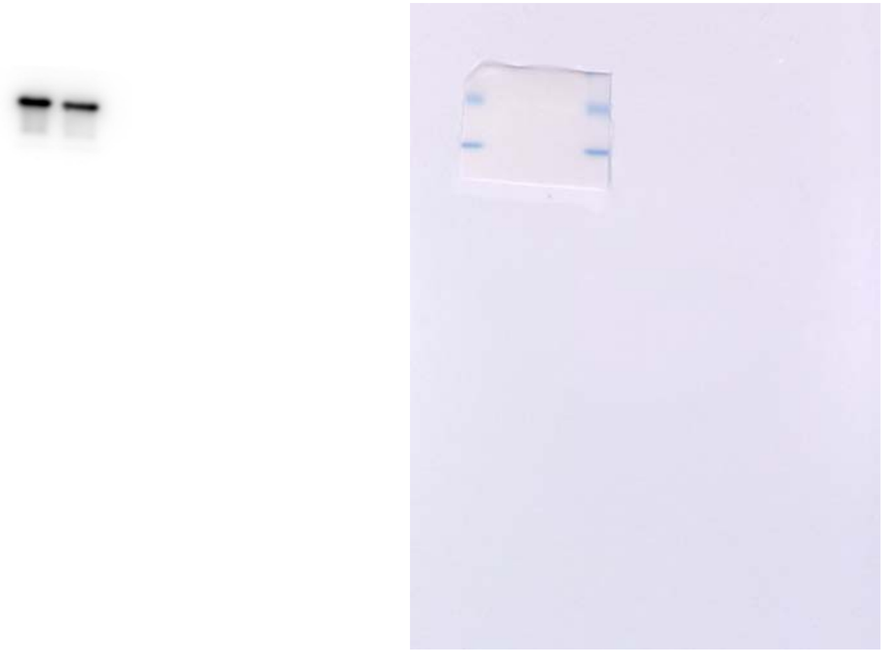

Bcl-2

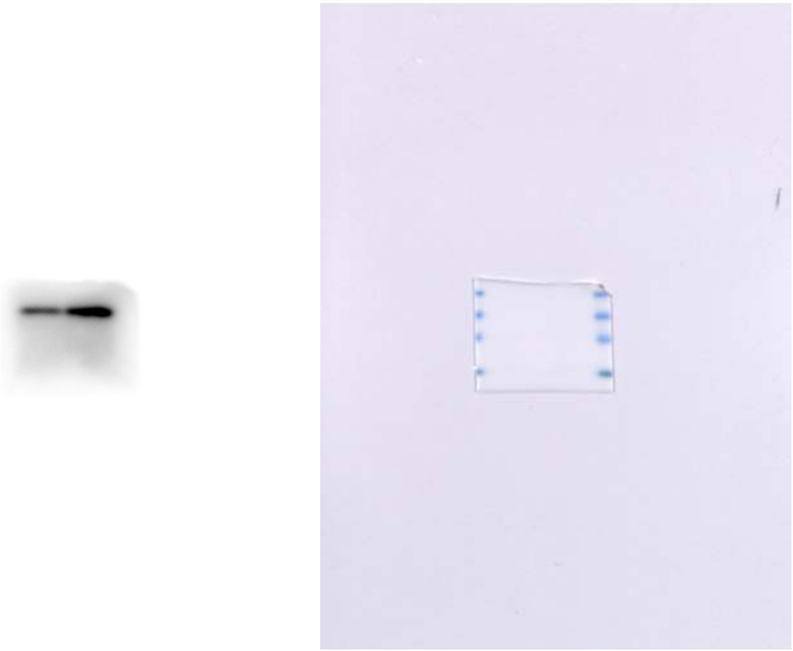

Caspase-7

Figure S4C

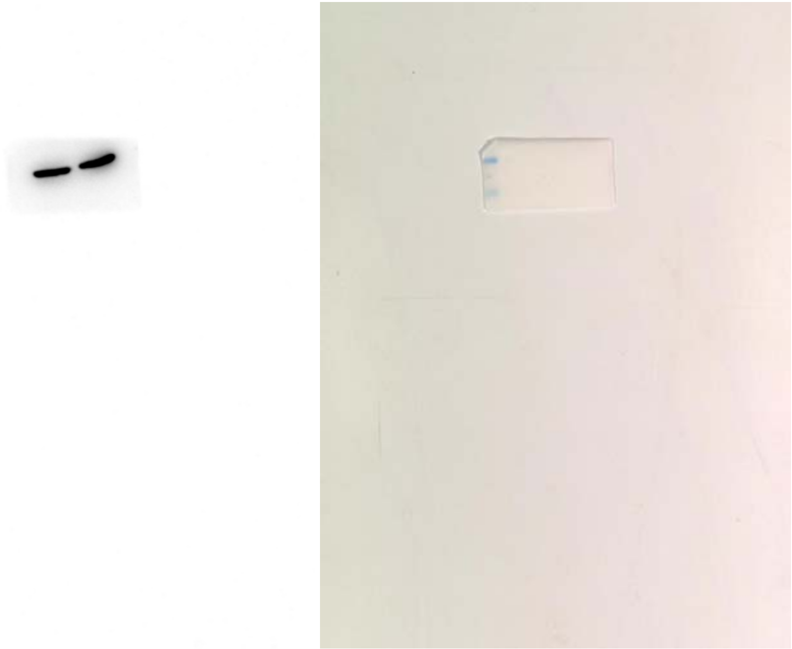

GAPDH

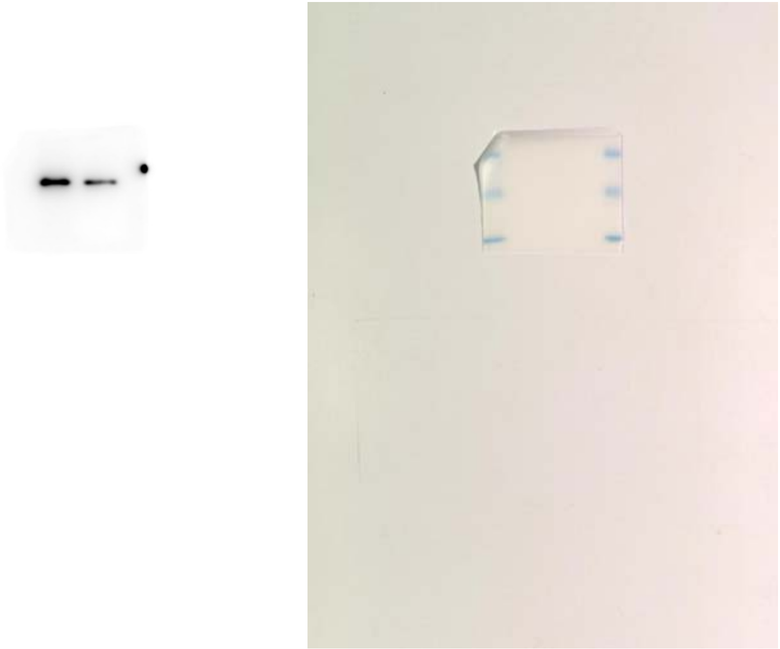

HSP27

Figure S5 DU 145

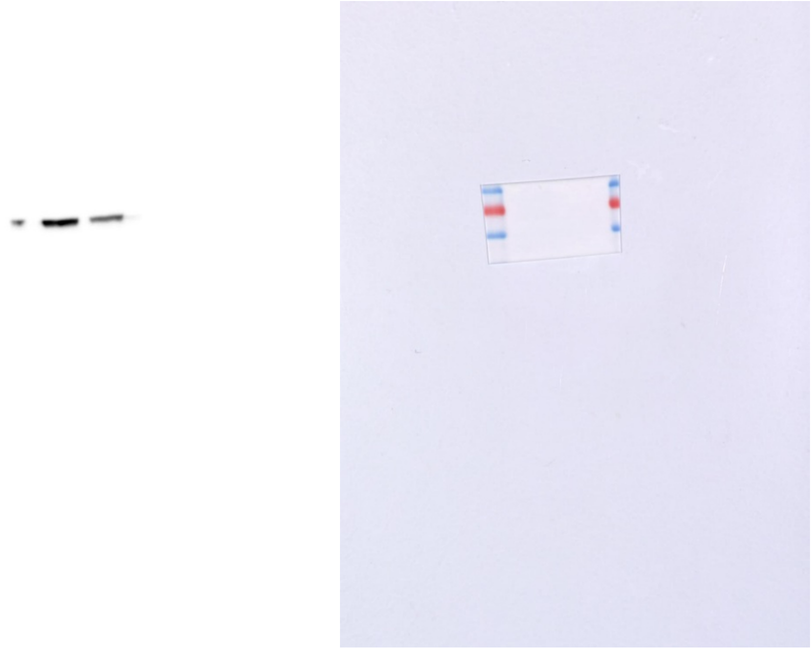

CDKL3

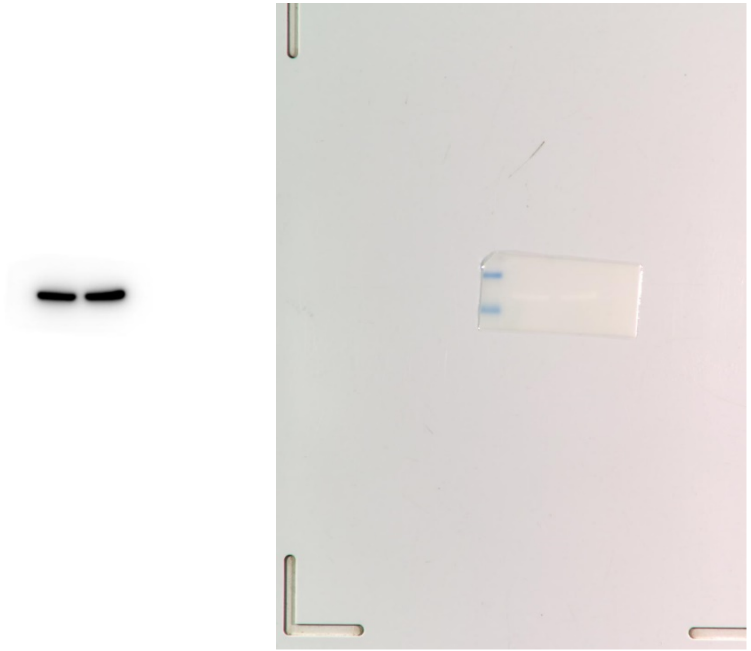

GAPDH

Figure S5 PC-3

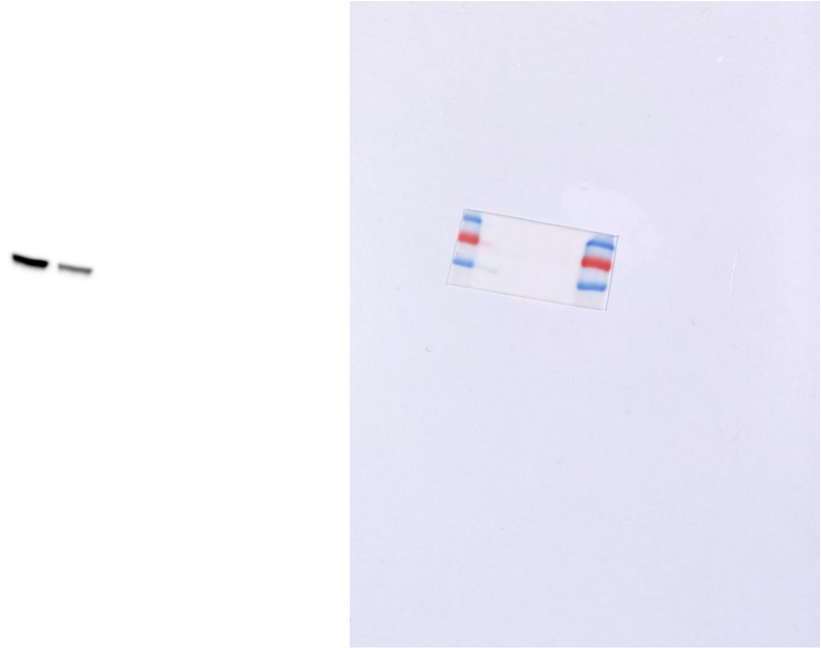

CDKL3

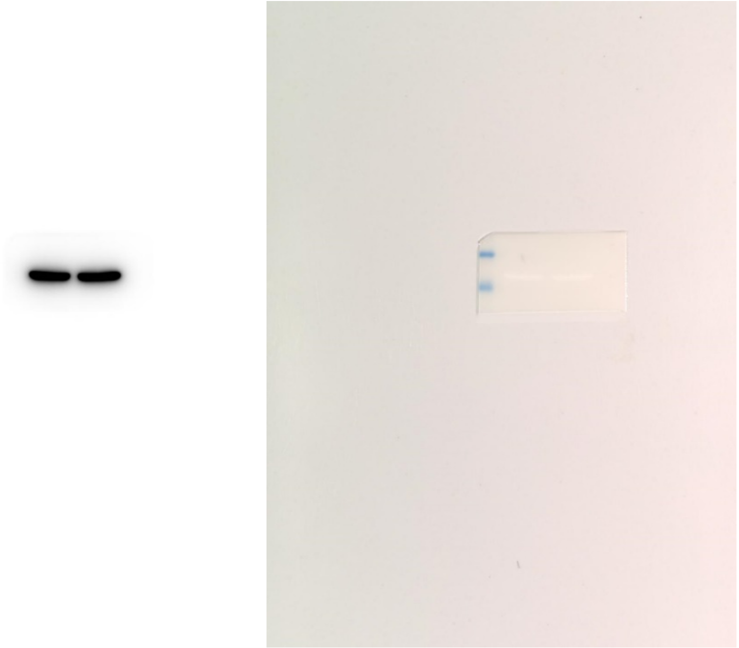

GAPDH

Figure S8

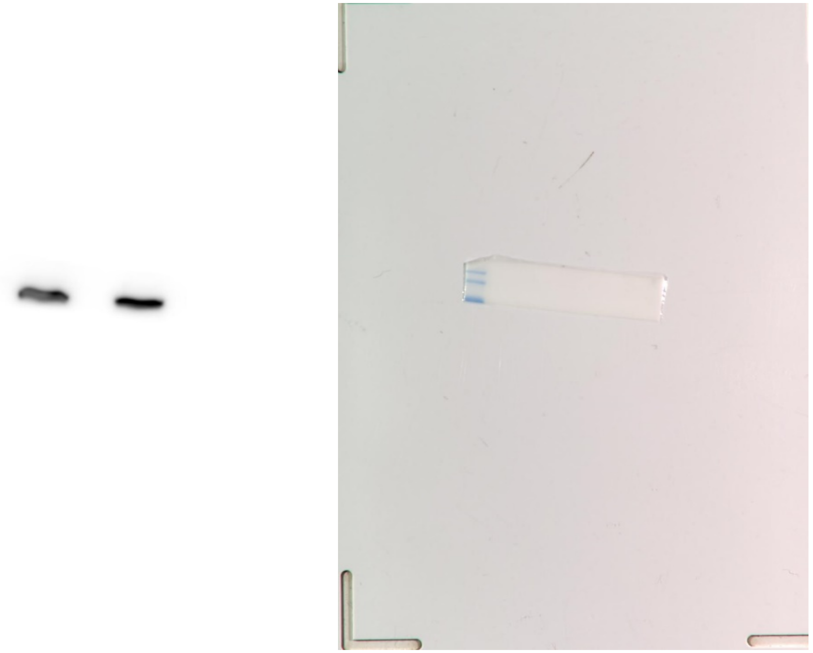

CBL

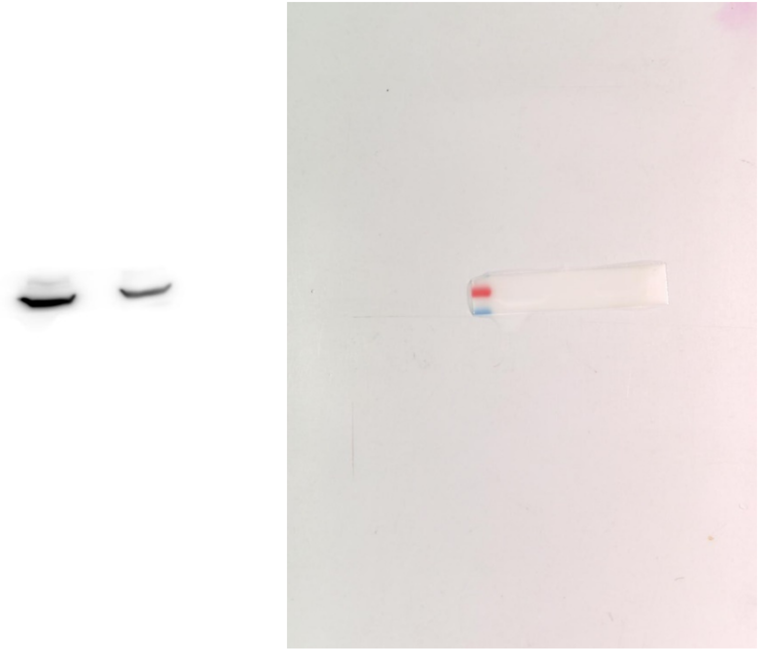

CDKL3

Figure S9

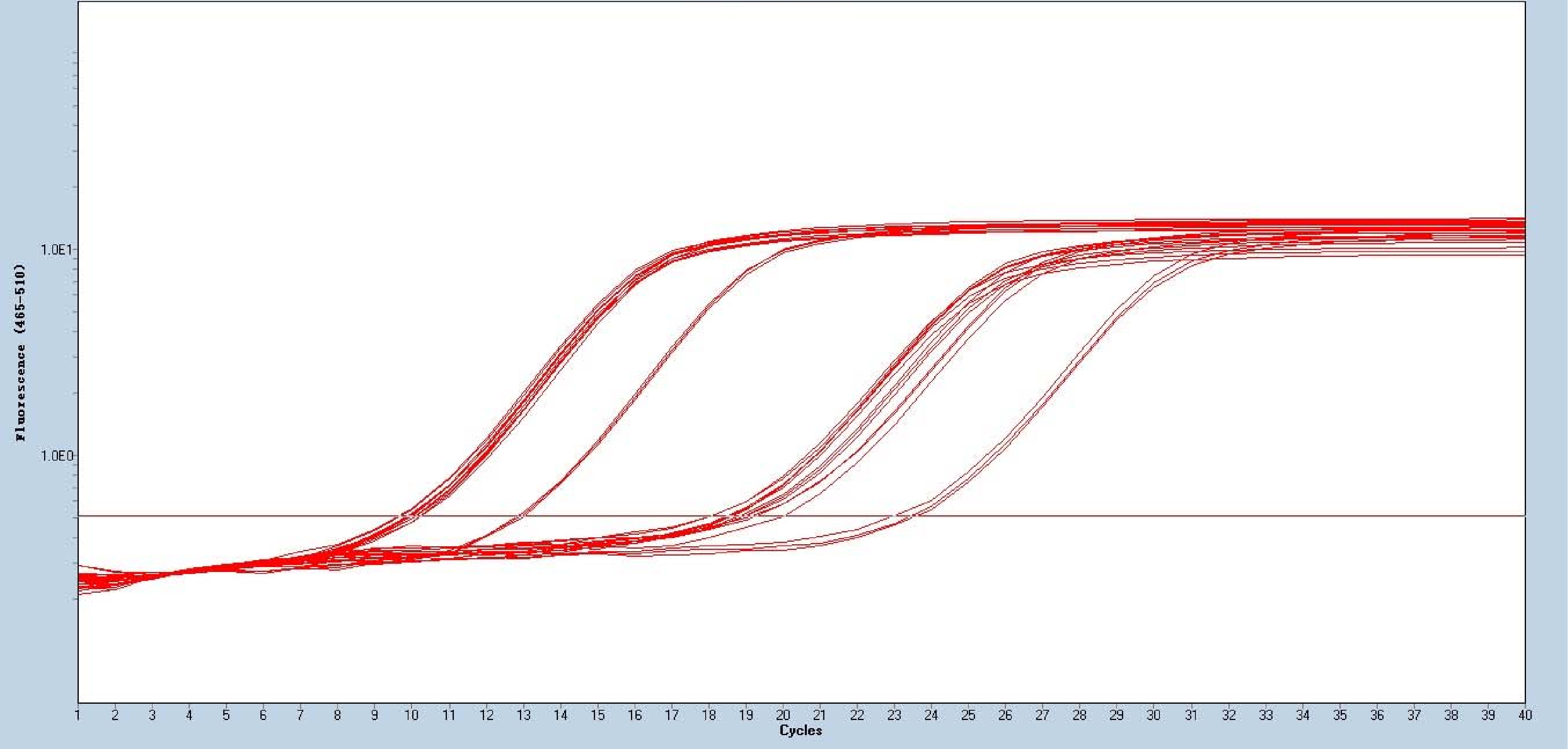
Amplification Curves
Fluorescence (465-510)
1.0E1
1.0E0
Cycles
1
2
3
4
5
6
7
8
9
10
11
12
13
14
15
16
17
18
19
20
21
22
23
24
25
26
27
28
29
30
31
32
33
34
35
36
37
38
39
40

Figure S9

Experiment: 20200804 ZHANGZ	Selected Filter: SYBR Green I / HRM Dye (465-510)							
Color	Pos	Include	Name	target gen	Cp	Tm1	Concentrat	Standard
255	D19	TRUE	DU 145 CON	H-GAPDH	10.3	82.08		0
255	D20	TRUE	DU 145 CON	H-GAPDH	10.37	82.13		0
255	D21	TRUE	DU 145 CON	H-GAPDH	10.28	82.26		0
255	D22	TRUE	DU 145 CON	H-STAT1	18.6	79.32		0
255	D23	TRUE	DU 145 CON	H-STAT1	18.37	79.42		0
255	D24	TRUE	DU 145 CON	H-STAT1	18.73	79.42		0
255	E19	TRUE	DU 145 shCtrl	H-GAPDH	10.43	82.13		0
255	E20	TRUE	DU 145 shCtrl	H-GAPDH	10.34	82.24		0
255	E21	TRUE	DU 145 shCtrl	H-GAPDH	10.29	82.26		0
255	E22	TRUE	DU 145 shCtrl	H-STAT1	18.32	79.33		0
255	E23	TRUE	DU 145 shCtrl	H-STAT1	18.68	79.37		0
255	E24	TRUE	DU 145 shCtrl	H-STAT1	18.61	79.44		0
255	F19	TRUE	DU 145 shSTAT1-1	H-GAPDH	9.77	82.26		0
255	F20	TRUE	DU 145 shSTAT1-1	H-GAPDH	9.92	82.21		0
255	F21	TRUE	DU 145 shSTAT1-1	H-GAPDH	9.71	82.25		0
255	F22	TRUE	DU 145 shSTAT1-1	H-STAT1	18.82	79.29		0
255	F23	TRUE	DU 145 shSTAT1-1	H-STAT1	18.92	79.3		0
255	F24	TRUE	DU 145 shSTAT1-1	H-STAT1	18.9	79.38		0
255	G19	TRUE	DU 145 shSTAT1-2	H-GAPDH	12.98	82.21		0
255	G20	TRUE	DU 145 shSTAT1-2	H-GAPDH	12.93	82.2		0
255	G21	TRUE	DU 145 shSTAT1-2	H-GAPDH	12.87	82.29		0
255	G22	TRUE	DU 145 shSTAT1-2	H-STAT1	22.94	79.34		0
255	G23	TRUE	DU 145 shSTAT1-2	H-STAT1	23.59	79.33		0
255	G24	TRUE	DU 145 shSTAT1-2	H-STAT1	23.74	79.33		0
255	H19	TRUE	DU 145 shSTAT1-3	H-GAPDH	9.89	82.31		0
255	H20	TRUE	DU 145 shSTAT1-3	H-GAPDH	9.95	82.3		0
255	H21	TRUE	DU 145 shSTAT1-3	H-GAPDH	9.98	82.25		0
255	H22	TRUE	DU 145 shSTAT1-3	H-STAT1	19.43	79.38		0
255	H23	TRUE	DU 145 shSTAT1-3	H-STAT1	19.41	79.38		0
255	H24	TRUE	DU 145 shSTAT1-3	H-STAT1	19.99	79.31		0

Figure S9

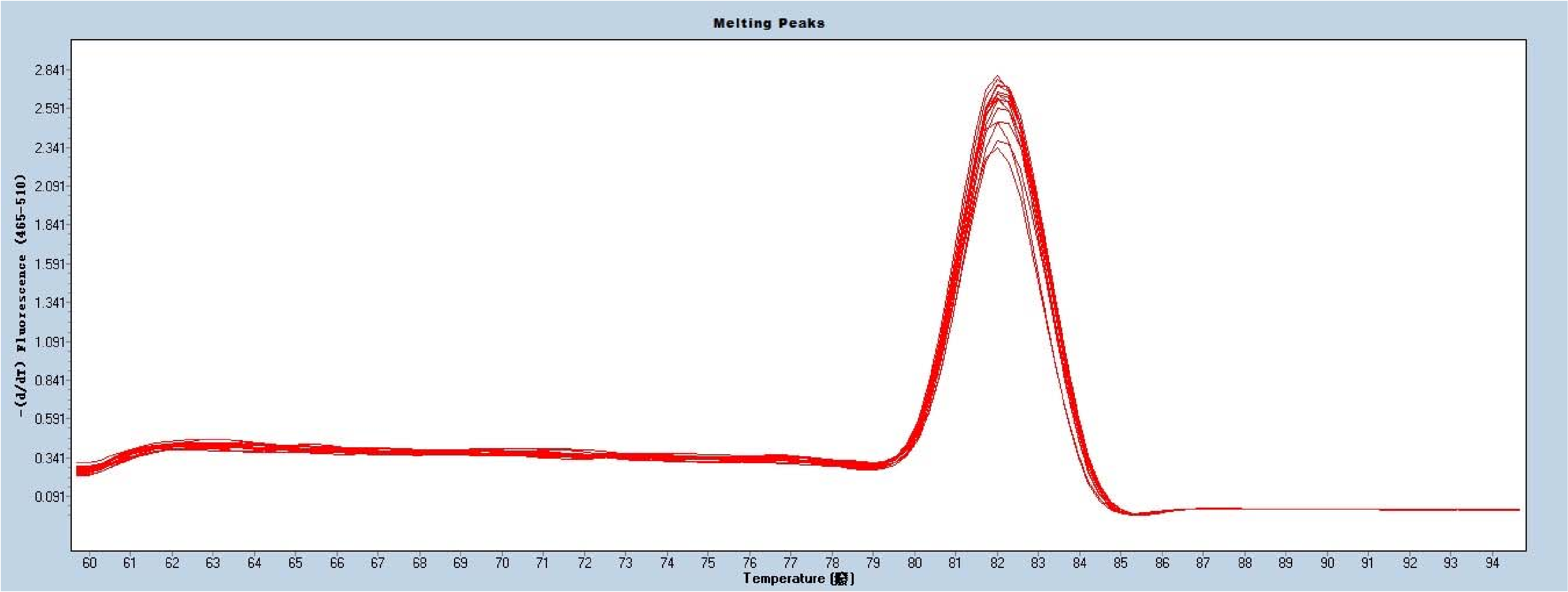
Melting Peaks
-(d/dT) Fluorescence (465-510)
2.841
2.591
2.341
2.091
1.841
1.591
1.341
1.091
0.841
0.591
0.341
0.091
60 61 62 63 64 65 66 67 68 69 70 71 72 73 74 75 76 77 78 79 80 81 82 83 84 85 86 87 88 89 90 91 92 93 94
Temperature

Figure S9

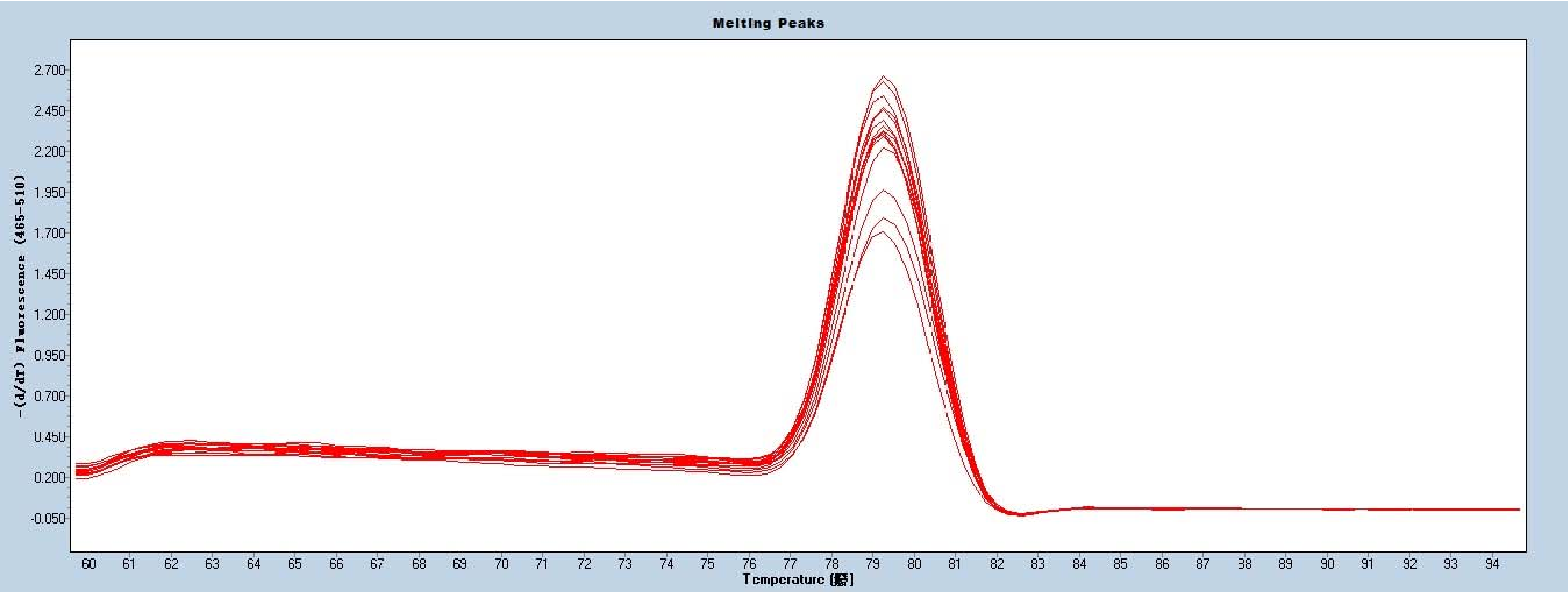

Melting Peaks
-(d/dT) Fluorescence (465-510)
2.700
2.450
2.200
1.950
1.700
1.450
1.200
0.950
0.700
0.450
0.200
-0.050
60
61
62
63
64
65
66
67
68
69
70
71
72
73
74
75
76
77
78
79
80
81
82
83
84
85
86
87
88
89
90
91
92
93
94
Temperature

Figure S10A

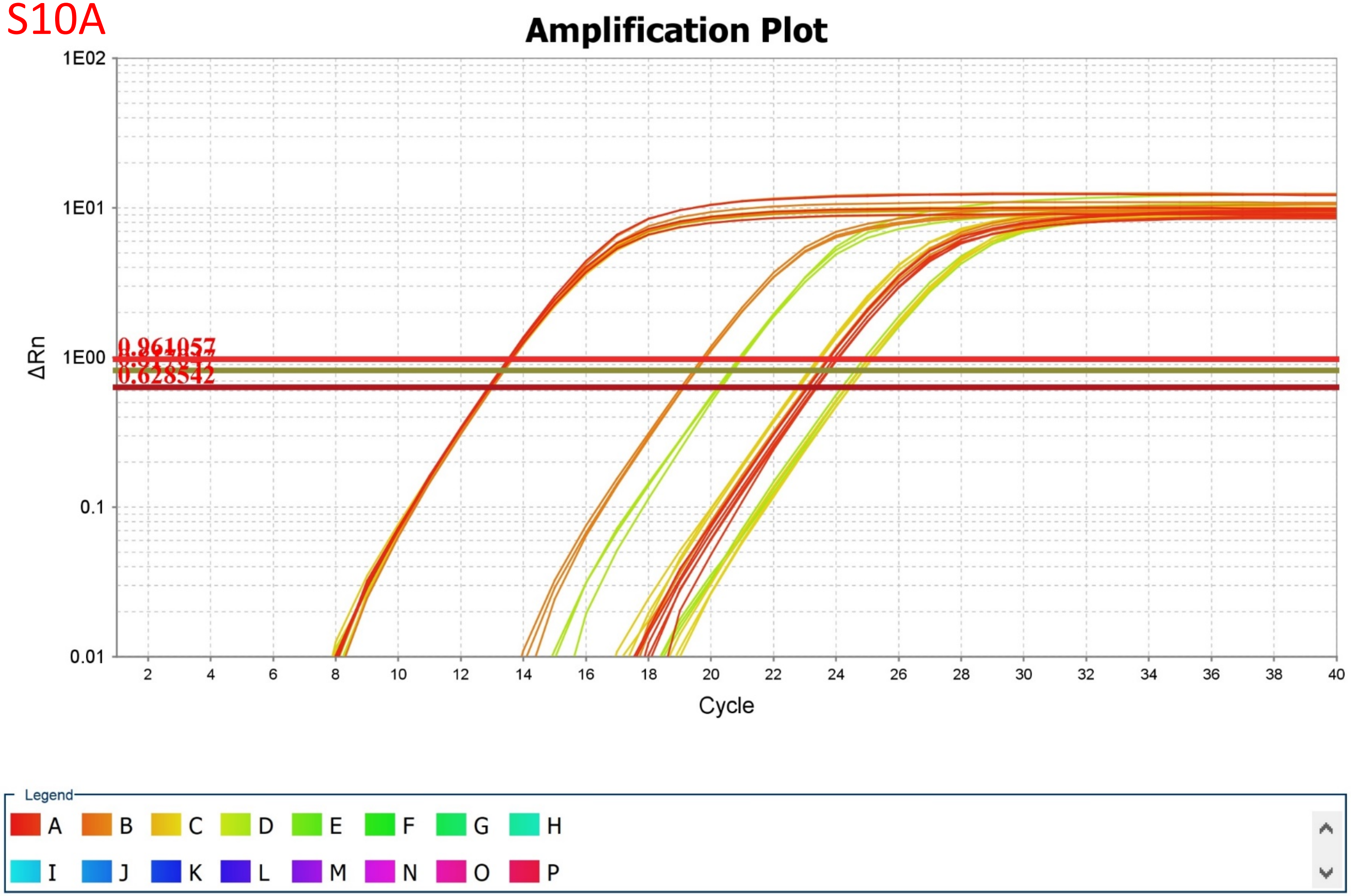
Amplification Plot
1E02
1E01
1E00
0.1
0.01
ΔRn
0.961057
0.628542
2
4
6
8
10
12
14
16
18
20
22
24
26
28
30
32
34
36
38
40
Cycle
Legend
A
B
C
D
E
F
G
H
I
J
K
L
M
N
O
P

Figure S10A

Block Type	384-Well Block
Calibration E	Yes
Calibration E	2020-08-14 10:25:44 AM CST
Calibration F	Yes
Calibration F	2020-08-14 11:18:18 AM CST
Calibration N	Yes
Calibration N	2020-08-14 10:47:55 AM CST
Calibration F	Yes
Calibration F	2020-08-14 10:19:00 AM CST
Calibration F	Yes
Calibration F	2020-08-14 11:03:25 AM CST
Calibration S	Yes
Calibration S	2020-08-14 11:10:52 AM CST
Calibration T	Yes
Calibration T	2020-08-14 10:55:44 AM CST
Calibration U	Yes
Calibration U	2020-08-14 10:33:02 AM CST
Calibration V	Yes
Calibration V	2020-08-14 10:40:30 AM CST
Chemistry	SYBR_GREEN
Experiment Barcode	
Experiment Comments	
Experiment	C:\Users\gonghq\Desktop\2021-07-20CDJ.eds
Experiment	2021-07-20CDJ
Experiment	2021-07-20 23:54:57 PM CST
Experiment	Comparative Cт (ΔΔCт)
Experiment User Name	
Instrument N	278882256
Instrument S	278882256
Instrument T	ViiA 7
Passive Ref	ROX
Quantificatic	Ct
Signal Smo	true
Stage/ Cycl	Stage 2, Step 2

Well	Well Positic	Omit	Sample Name	Target Nam	Task	Reporter	Quencher	Quantity	Quantity Me	Quantity SD	RQ	RQ Min	RQ Max	CT	Ct Mean
13	A13	FALSE	DU 145 NC(OE+KD)	H-GAPDH	UNKNOWN	SYBR	None							12.869518	
14	A14	FALSE	DU 145 NC(OE+KD)	H-GAPDH	UNKNOWN	SYBR	None							12.921873	
15	A15	FALSE	DU 145 NC(OE+KD)	H-GAPDH	UNKNOWN	SYBR	None							12.878902	
16	A16	FALSE	DU 145 NC(OE+KD)	H-CDKL3	UNKNOWN	SYBR	None							23.46899	
17	A17	FALSE	DU 145 NC(OE+KD)	H-CDKL3	UNKNOWN	SYBR	None							23.495674	
18	A18	FALSE	DU 145 NC(OE+KD)	H-CDKL3	UNKNOWN	SYBR	None							23.629553	
19	A19	FALSE	DU 145 NC(OE+KD)	H-STAT1	UNKNOWN	SYBR	None							23.985607	
20	A20	FALSE	DU 145 NC(OE+KD)	H-STAT1	UNKNOWN	SYBR	None							23.965137	
21	A21	FALSE	DU 145 NC(OE+KD)	H-STAT1	UNKNOWN	SYBR	None							23.990973	
37	B13	FALSE	DU 145 CDKL3+NC-shSTAT1	H-GAPDH	UNKNOWN	SYBR	None							12.978873	
38	B14	FALSE	DU 145 CDKL3+NC-shSTAT1	H-GAPDH	UNKNOWN	SYBR	None							12.940309	
39	B15	FALSE	DU 145 CDKL3+NC-shSTAT1	H-GAPDH	UNKNOWN	SYBR	None							13.004471	
40	B16	FALSE	DU 145 CDKL3+NC-shSTAT1	H-CDKL3	UNKNOWN	SYBR	None							19.434507	
41	B17	FALSE	DU 145 CDKL3+NC-shSTAT1	H-CDKL3	UNKNOWN	SYBR	None							19.506077	
42	B18	FALSE	DU 145 CDKL3+NC-shSTAT1	H-CDKL3	UNKNOWN	SYBR	None							19.525265	
43	B19	FALSE	DU 145 CDKL3+NC-shSTAT1	H-STAT1	UNKNOWN	SYBR	None							23.667553	
44	B20	FALSE	DU 145 CDKL3+NC-shSTAT1	H-STAT1	UNKNOWN	SYBR	None							23.69375	
45	B21	FALSE	DU 145 CDKL3+NC-shSTAT1	H-STAT1	UNKNOWN	SYBR	None							23.716137	
61	C13	FALSE	DU 145 STAT1+NC-shCDKL3	H-GAPDH	UNKNOWN	SYBR	None							13.00252	
62	C14	FALSE	DU 145 STAT1+NC-shCDKL3	H-GAPDH	UNKNOWN	SYBR	None							12.963161	
63	C15	FALSE	DU 145 STAT1+NC-shCDKL3	H-GAPDH	UNKNOWN	SYBR	None							12.926838	
64	C16	FALSE	DU 145 STAT1+NC-shCDKL3	H-CDKL3	UNKNOWN	SYBR	None							23.484057	
65	C17	FALSE	DU 145 STAT1+NC-shCDKL3	H-CDKL3	UNKNOWN	SYBR	None							23.490641	
66	C18	FALSE	DU 145 STAT1+NC-shCDKL3	H-CDKL3	UNKNOWN	SYBR	None							23.412188	
67	C19	FALSE	DU 145 STAT1+NC-shCDKL3	H-STAT1	UNKNOWN	SYBR	None							24.974489	
68	C20	FALSE	DU 145 STAT1+NC-shCDKL3	H-STAT1	UNKNOWN	SYBR	None							25.07188	
69	C21	FALSE	DU 145 STAT1+NC-shCDKL3	H-STAT1	UNKNOWN	SYBR	None							25.072912	
85	D13	FALSE	DU 145 CDKL3+STAT1	H-GAPDH	UNKNOWN	SYBR	None							12.928718	
86	D14	FALSE	DU 145 CDKL3+STAT1	H-GAPDH	UNKNOWN	SYBR	None							12.985093	
87	D15	FALSE	DU 145 CDKL3+STAT1	H-GAPDH	UNKNOWN	SYBR	None							12.970517	
88	D16	FALSE	DU 145 CDKL3+STAT1	H-CDKL3	UNKNOWN	SYBR	None							20.608906	
89	D17	FALSE	DU 145 CDKL3+STAT1	H-CDKL3	UNKNOWN	SYBR	None							20.730221	
90	D18	FALSE	DU 145 CDKL3+STAT1	H-CDKL3	UNKNOWN	SYBR	None							20.654099	
91	D19	FALSE	DU 145 CDKL3+STAT1	H-STAT1	UNKNOWN	SYBR	None							24.146981	
92	D20	FALSE	DU 145 CDKL3+STAT1	H-STAT1	UNKNOWN	SYBR	None							24.19354	
93	D21	FALSE	DU 145 CDKL3+STAT1	H-STAT1	UNKNOWN	SYBR	None							24.193612	

Figure S10A

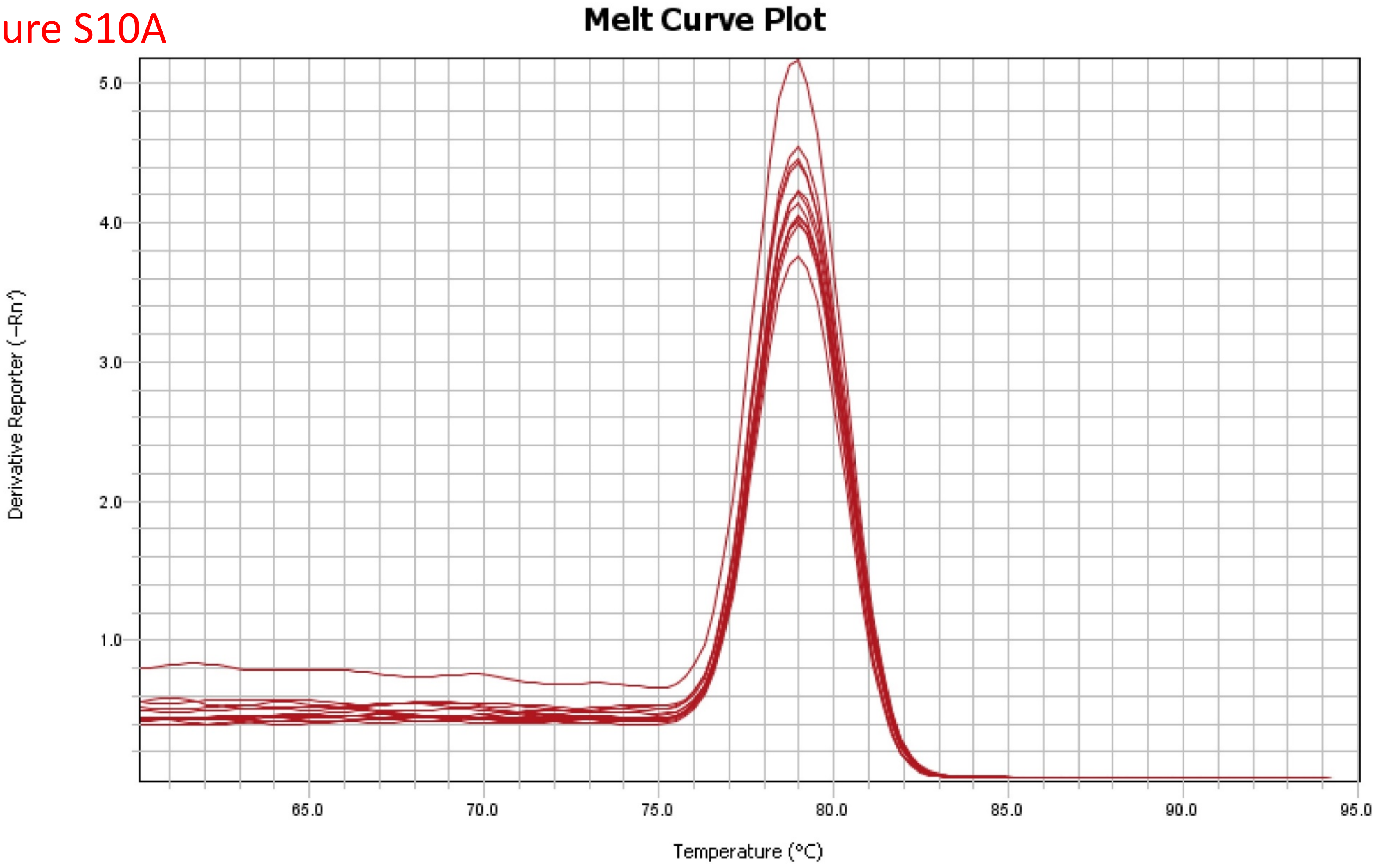
Melt Curve Plot
5.0
4.0
3.0
2.0
1.0
Derivative Reporter (−Rn')
65.0
70.0
75.0
80.0
85.0
90.0
95.0
Temperature (°C)

Figure S10A

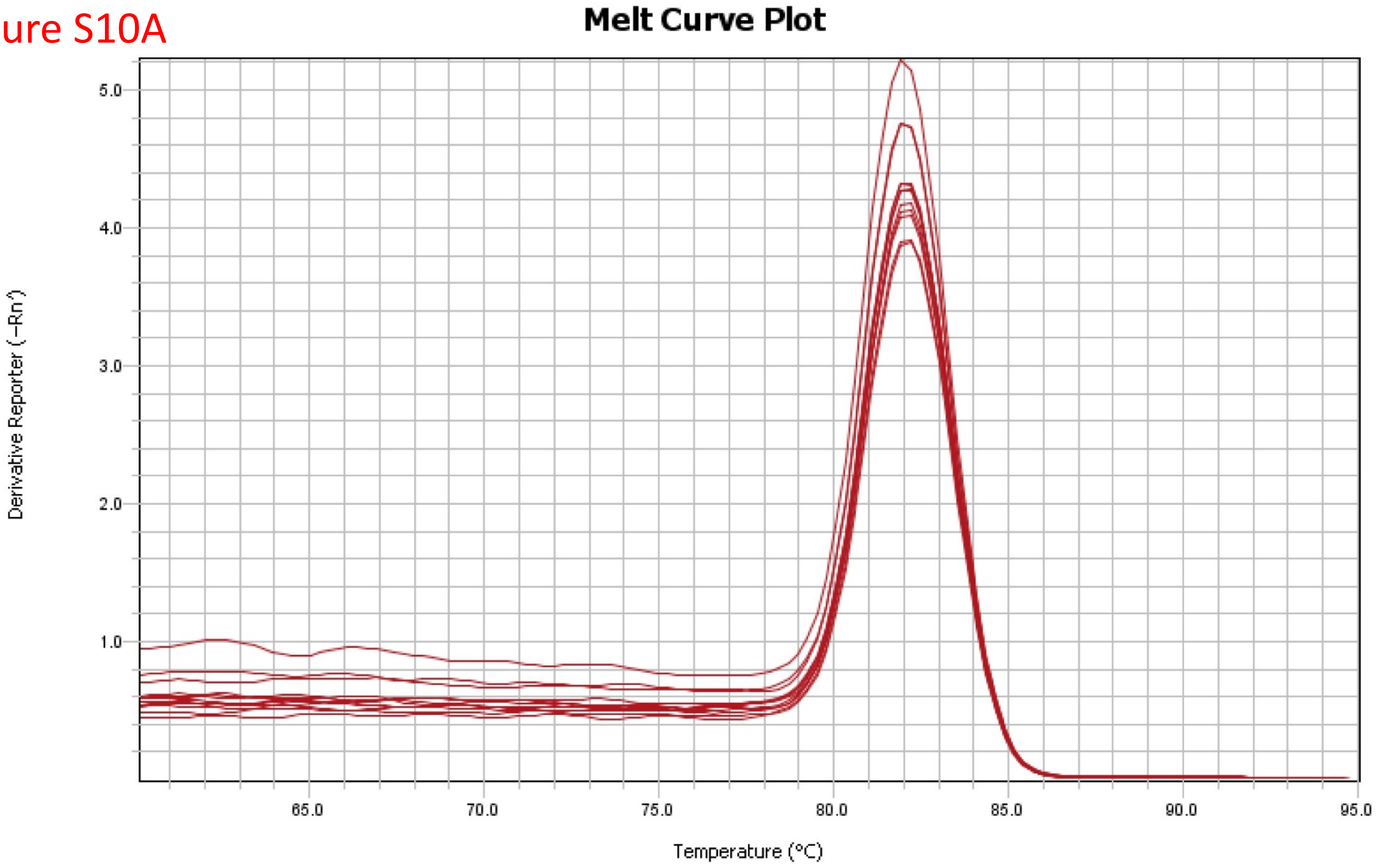

Melt Curve Plot
Derivative Reporter (−Rn')
5.0
4.0
3.0
2.0
1.0
65.0
70.0
75.0
80.0
85.0
90.0
95.0
Temperature (°C)

Figure S10A

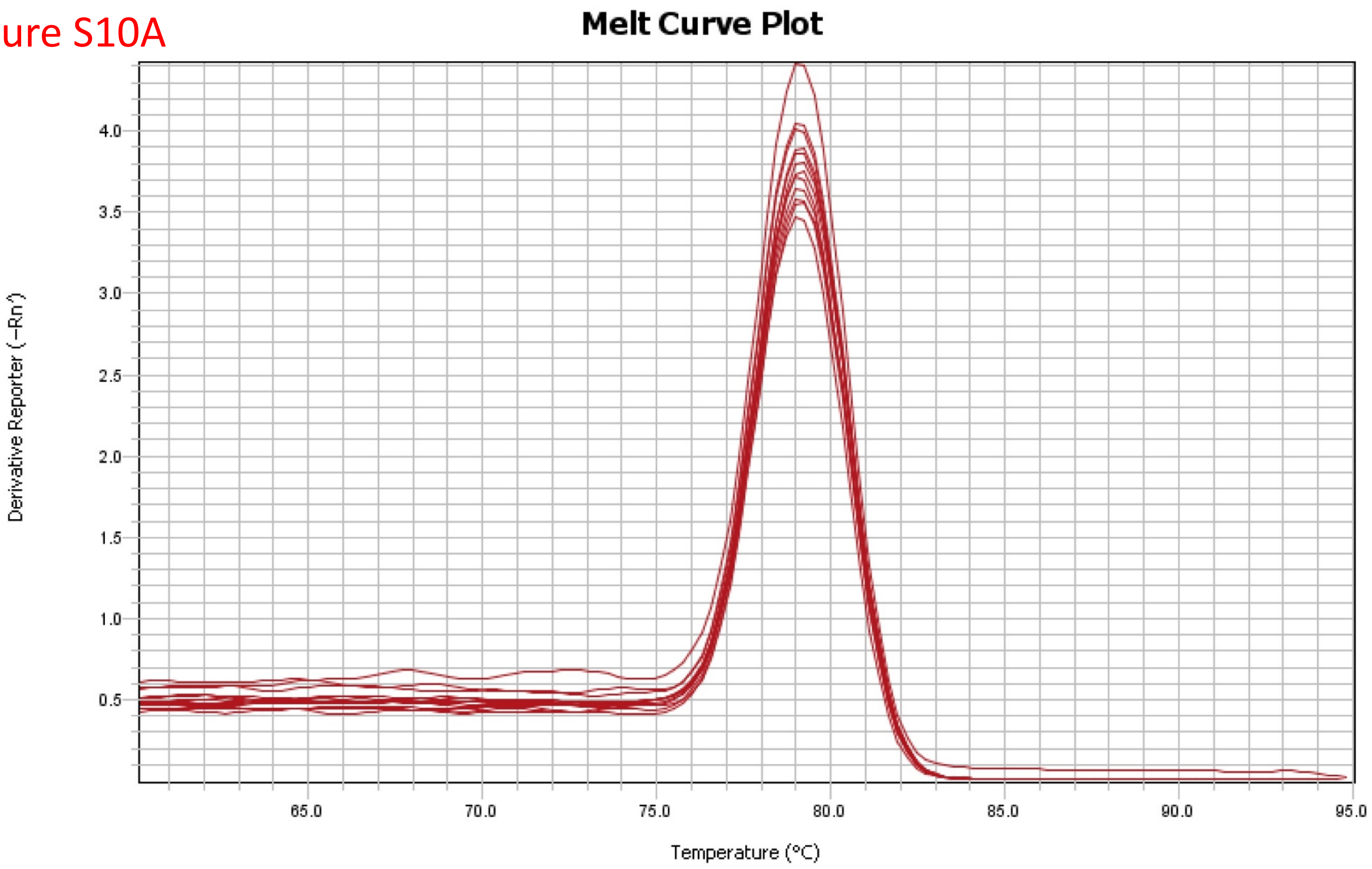
Melt Curve Plot
Derivative Reporter (−Rn')
4.0
3.5
3.0
2.5
2.0
1.5
1.0
0.5
65.0
70.0
75.0
80.0
85.0
90.0
95.0
Temperature (°C)

Figure S10B

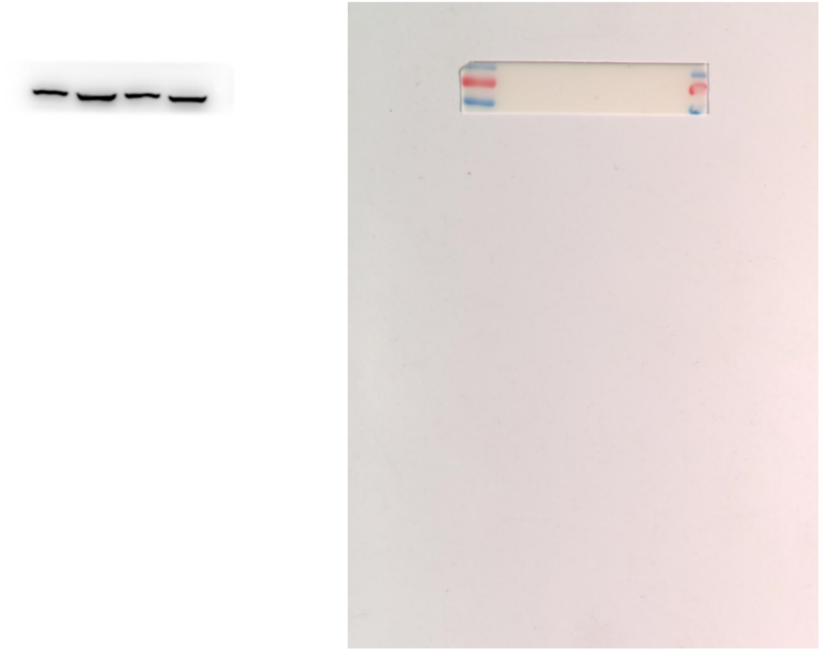

CDKL3

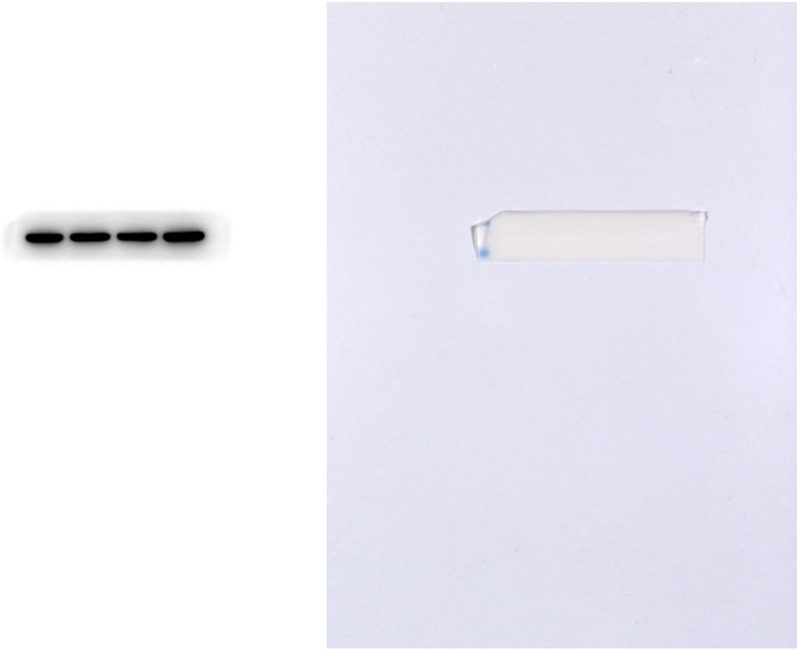

GAPDH

Figure S10B

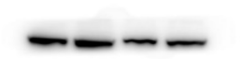

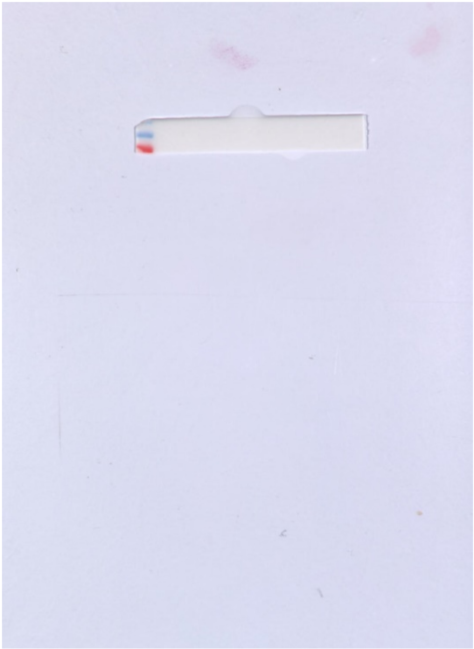

STAT1

Figure S11A

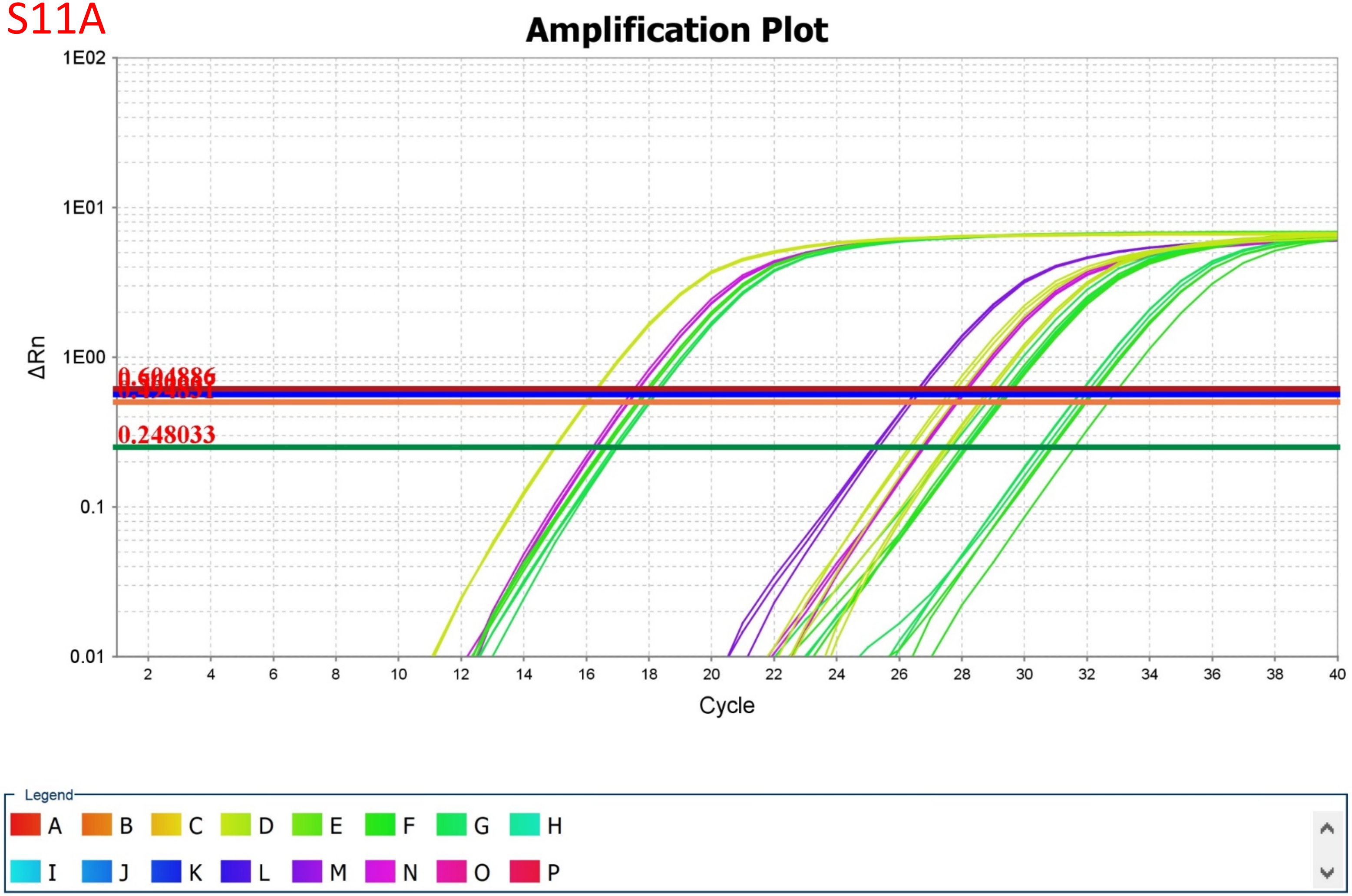
Amplification Plot
1E02
1E01
1E00
0.1
0.01
ΔRn
0.604886
0.248033
2 4 6 8 10 12 14 16 18 20 22 24 26 28 30 32 34 36 38 40
Cycle
Legend
A B C D E F G H
I J K L M N O P

Figure S11A

Block Type	384-Well Block
Calibration E	No
Calibration E	2022-08-01 16:00:17 PM CST
Calibration F	No
Calibration F	2022-08-01 16:39:53 PM CST
Calibration N	No
Calibration N	2022-08-01 16:56:18 PM CST
Calibration F	No
Calibration F	2022-08-01 15:53:01 PM CST
Calibration F	No
Calibration F	2022-08-01 17:25:54 PM CST
Calibration S	No
Calibration S	2022-08-01 17:03:33 PM CST
Calibration T	No
Calibration T	2022-08-01 17:10:48 PM CST
Calibration U	No
Calibration U	2022-08-01 16:16:55 PM CST
Calibration V	No
Calibration V	2022-08-01 16:48:55 PM CST
Chemistry	SYBR_GREEN
Experiment Barcode	
Experiment Comments	
Experiment	C:\Users\zhouch\Desktop\2022-11-16 11-15-1.eds
Experiment	2022-11-16 11-15-1
Experiment	2022-11-16 16:34:23 PM CST
Experiment	Comparative Cт (ΔΔCт)
Experiment User Name	
Instrument N	278882256
Instrument S	278882256
Instrument T	ViiA 7
Passive Ref	ROX
Quantificatio	Ct
Signal Smo	true
Stage/ Cycl	Stage 2, Step 2

Well	Well Positic	Omit	Sample Name	Target Nam	Task	Reporter	Quencher	Quantity	Quantity Me	Quantity SD	RQ	RQ Min	RQ Max	CT	Ct Mean
73	D1	FALSE	PC-3 NC(OE+KD)	H-GAPDH	UNKNOWN	SYBR	None							16.306	
74	D2	FALSE	PC-3 NC(OE+KD)	H-GAPDH	UNKNOWN	SYBR	None							16.250	
75	D3	FALSE	PC-3 NC(OE+KD)	H-GAPDH	UNKNOWN	SYBR	None							16.280	
76	D4	FALSE	PC-3 NC(OE+KD)	H-CDKL3	UNKNOWN	SYBR	None							27.662	
77	D5	FALSE	PC-3 NC(OE+KD)	H-CDKL3	UNKNOWN	SYBR	None							27.525	
78	D6	FALSE	PC-3 NC(OE+KD)	H-CDKL3	UNKNOWN	SYBR	None							27.895	
79	D7	FALSE	PC-3 NC(OE+KD)	H-STAT1	UNKNOWN	SYBR	None							27.633	
80	D8	FALSE	PC-3 NC(OE+KD)	H-STAT1	UNKNOWN	SYBR	None							27.543	
81	D9	FALSE	PC-3 NC(OE+KD)	H-STAT1	UNKNOWN	SYBR	None							27.491	
97	E1	FALSE	PC-3 CDKL3+NC(KD)	H-GAPDH	UNKNOWN	SYBR	None							17.638	
98	E2	FALSE	PC-3 CDKL3+NC(KD)	H-GAPDH	UNKNOWN	SYBR	None							17.488	
99	E3	FALSE	PC-3 CDKL3+NC(KD)	H-GAPDH	UNKNOWN	SYBR	None							17.622	
100	E4	FALSE	PC-3 CDKL3+NC(KD)	H-CDKL3	UNKNOWN	SYBR	None							26.231	
101	E5	FALSE	PC-3 CDKL3+NC(KD)	H-CDKL3	UNKNOWN	SYBR	None							26.377	
102	E6	FALSE	PC-3 CDKL3+NC(KD)	H-CDKL3	UNKNOWN	SYBR	None							26.258	
103	E7	FALSE	PC-3 CDKL3+NC(KD)	H-STAT1	UNKNOWN	SYBR	None							27.838	
104	E8	FALSE	PC-3 CDKL3+NC(KD)	H-STAT1	UNKNOWN	SYBR	None							27.846	
105	E9	FALSE	PC-3 CDKL3+NC(KD)	H-STAT1	UNKNOWN	SYBR	None							27.761	
121	F1	FALSE	PC-3 shSTAT1+NC(OE)	H-GAPDH	UNKNOWN	SYBR	None							17.915	
122	F2	FALSE	PC-3 shSTAT1+NC(OE)	H-GAPDH	UNKNOWN	SYBR	None							17.979	
123	F3	FALSE	PC-3 shSTAT1+NC(OE)	H-GAPDH	UNKNOWN	SYBR	None							17.905	
124	F4	FALSE	PC-3 shSTAT1+NC(OE)	H-CDKL3	UNKNOWN	SYBR	None							29.489	
125	F5	FALSE	PC-3 shSTAT1+NC(OE)	H-CDKL3	UNKNOWN	SYBR	None							29.443	
126	F6	FALSE	PC-3 shSTAT1+NC(OE)	H-CDKL3	UNKNOWN	SYBR	None							29.242	
127	F7	FALSE	PC-3 shSTAT1+NC(OE)	H-STAT1	UNKNOWN	SYBR	None							30.846	
128	F8	FALSE	PC-3 shSTAT1+NC(OE)	H-STAT1	UNKNOWN	SYBR	None							31.618	
129	F9	FALSE	PC-3 shSTAT1+NC(OE)	H-STAT1	UNKNOWN	SYBR	None							30.910	
145	G1	FALSE	PC-3 CDKL3+shSTAT1	H-GAPDH	UNKNOWN	SYBR	None							18.311	
146	G2	FALSE	PC-3 CDKL3+shSTAT1	H-GAPDH	UNKNOWN	SYBR	None							18.201	
147	G3	FALSE	PC-3 CDKL3+shSTAT1	H-GAPDH	UNKNOWN	SYBR	None							18.296	
148	G4	FALSE	PC-3 CDKL3+shSTAT1	H-CDKL3	UNKNOWN	SYBR	None							28.981	
149	G5	FALSE	PC-3 CDKL3+shSTAT1	H-CDKL3	UNKNOWN	SYBR	None							29.226	
150	G6	FALSE	PC-3 CDKL3+shSTAT1	H-CDKL3	UNKNOWN	SYBR	None							29.357	
151	G7	FALSE	PC-3 CDKL3+shSTAT1	H-STAT1	UNKNOWN	SYBR	None							30.501	
152	G8	FALSE	PC-3 CDKL3+shSTAT1	H-STAT1	UNKNOWN	SYBR	None							30.529	
153	G9	FALSE	PC-3 CDKL3+shSTAT1	H-STAT1	UNKNOWN	SYBR	None							30.703	

Figure S11A

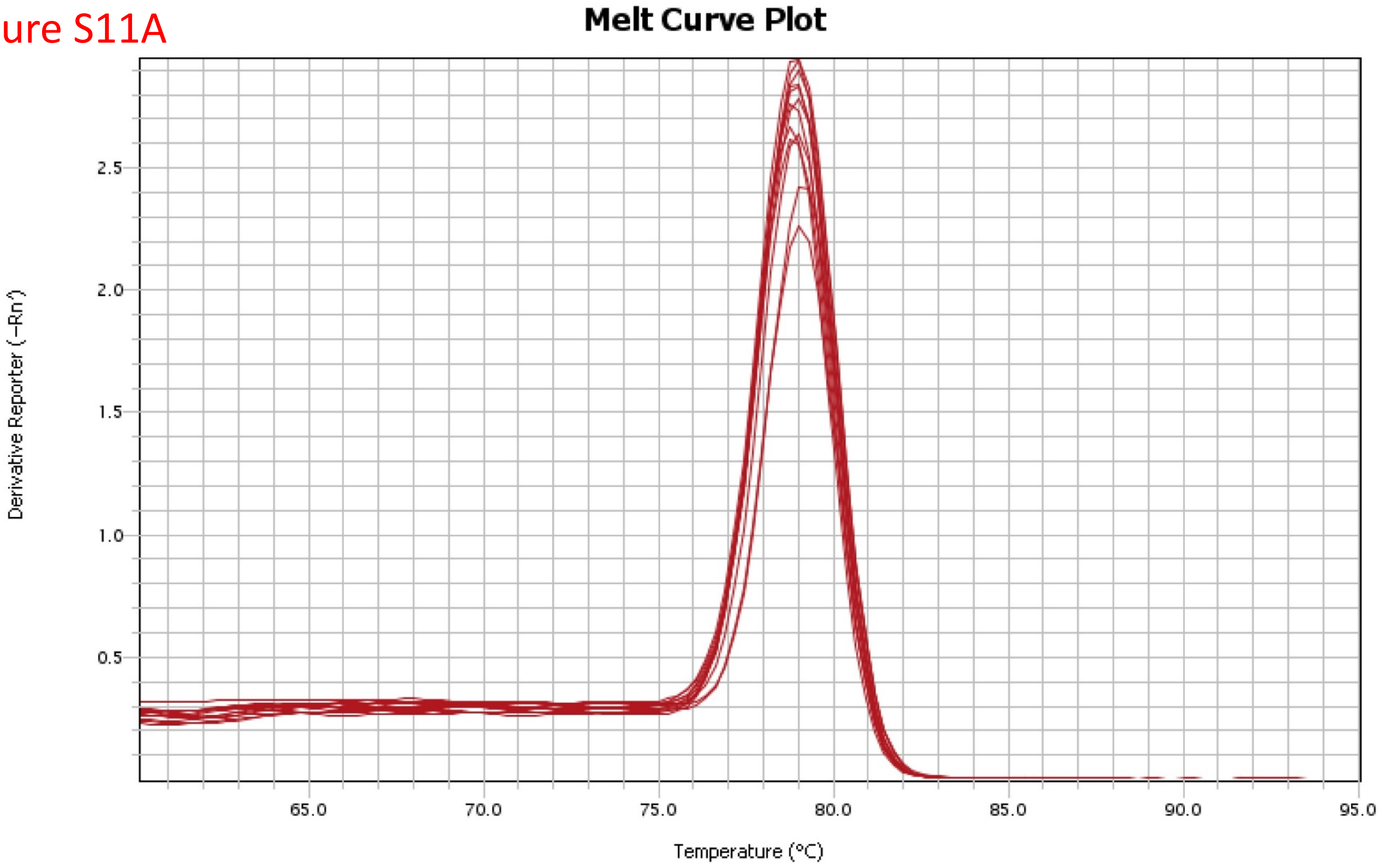
Melt Curve Plot
2.5
2.0
1.5
1.0
0.5
Derivative Reporter (−Rn')
65.0
70.0
75.0
80.0
85.0
90.0
95.0
Temperature (°C)

Figure S11A

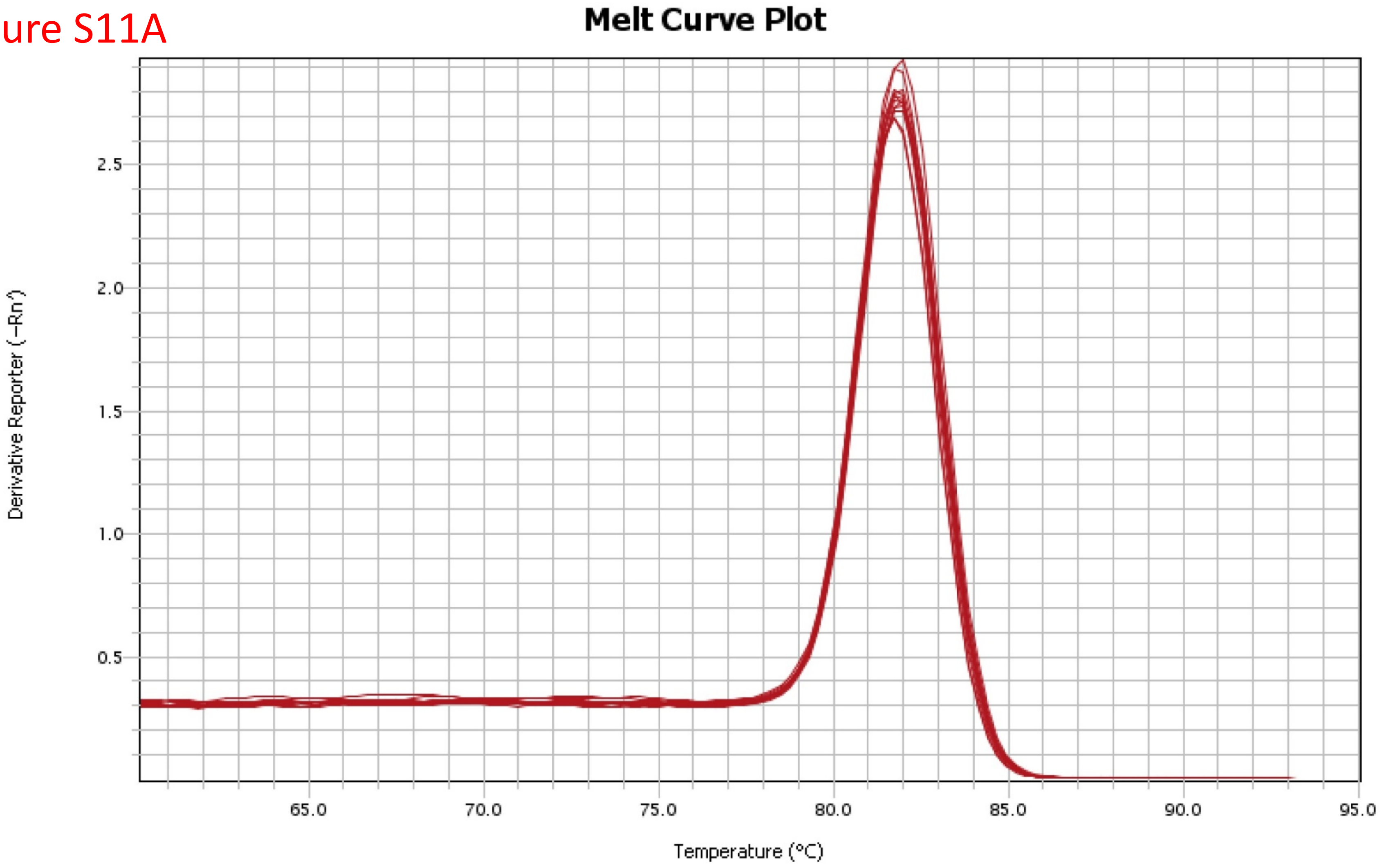
Melt Curve Plot
Derivative Reporter (−Rn')
2.5
2.0
1.5
1.0
0.5
65.0
70.0
75.0
80.0
85.0
90.0
95.0
Temperature (°C)

Figure S11A

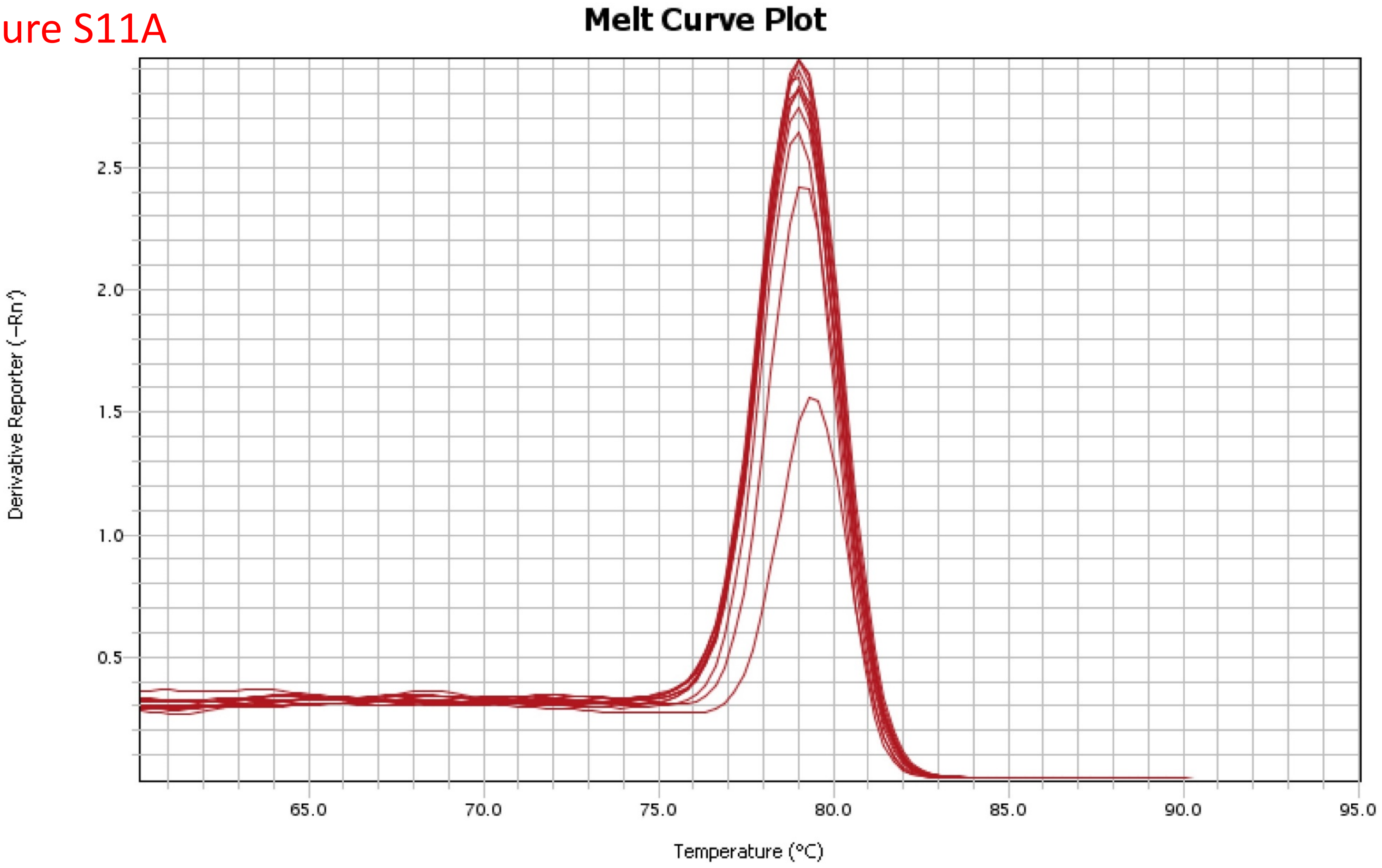
Melt Curve Plot
Derivative Reporter (−Rn')
2.5
2.0
1.5
1.0
0.5
65.0
70.0
75.0
80.0
85.0
90.0
95.0
Temperature (°C)

Figure S11B

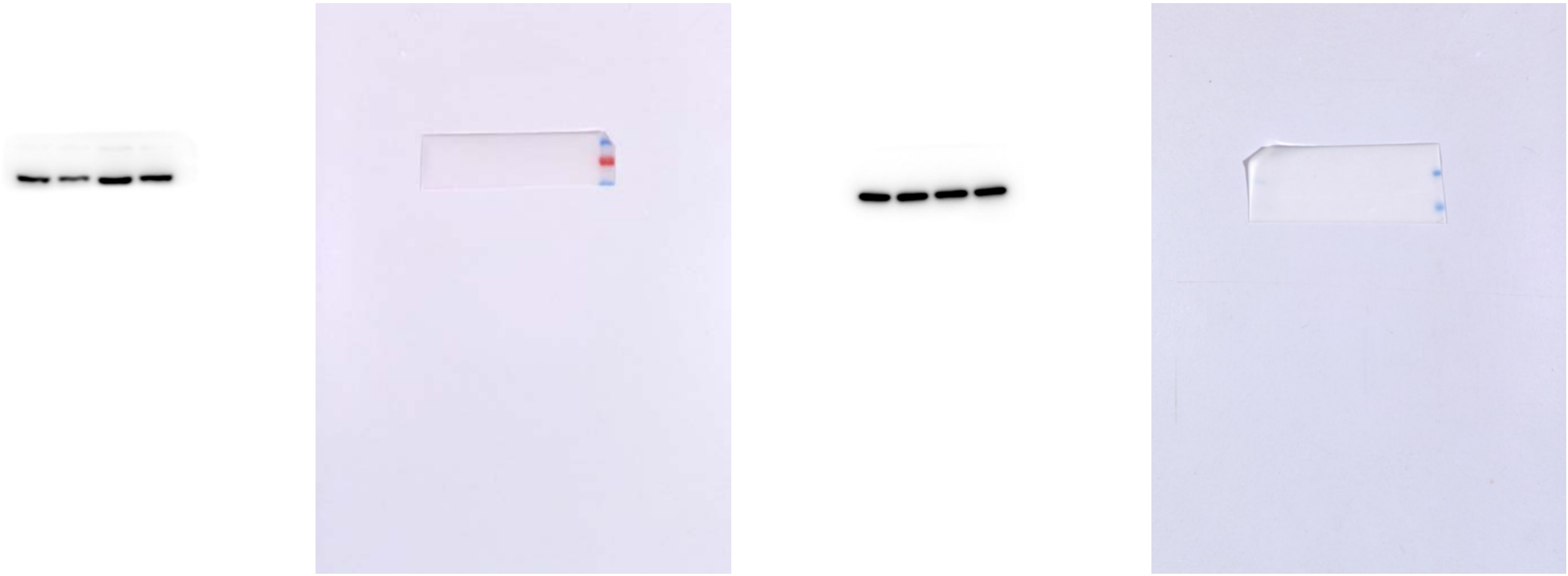

CDKL3 GAPDH

Figure S11B

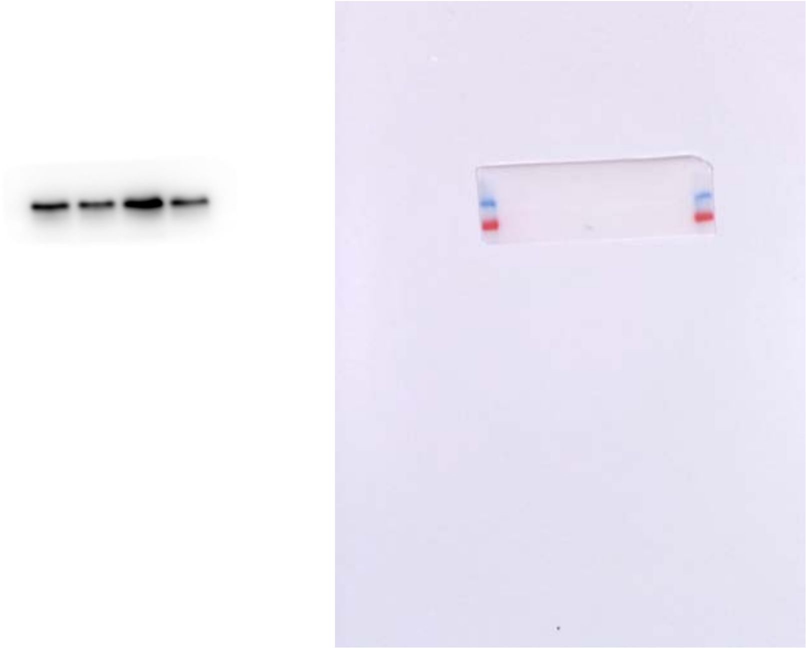

STAT1

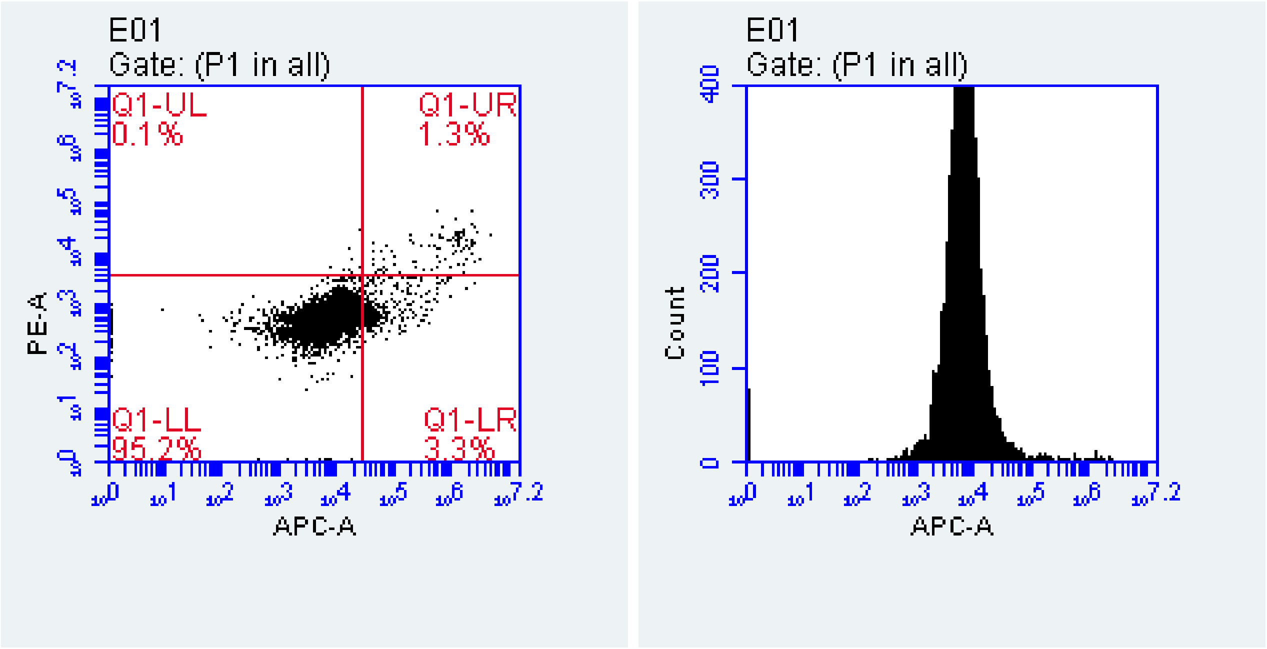
E01
Gate: (P1 in all)
Q1-UL
0.1%
Q1-UR
1.3%
Q1-LL
95.2%
Q1-LR
3.3%
PE-A
APC-A
E01
Gate: (P1 in all)
Count
APC-A

Figure S12C

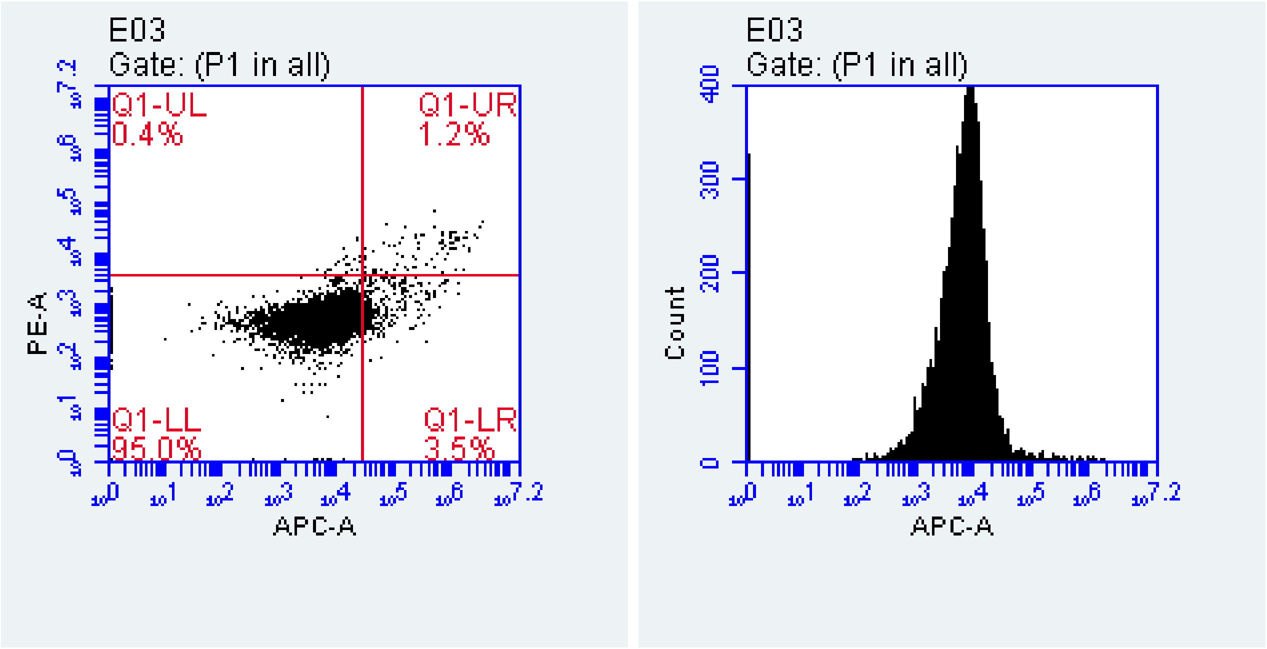
E03
Gate: (P1 in all)
Q1-UL
0.4%
Q1-UR
1.2%
Q1-LL
95.0%
Q1-LR
3.5%
PE-A
APC-A
E03
Gate: (P1 in all)
Count
APC-A

Figure S12C

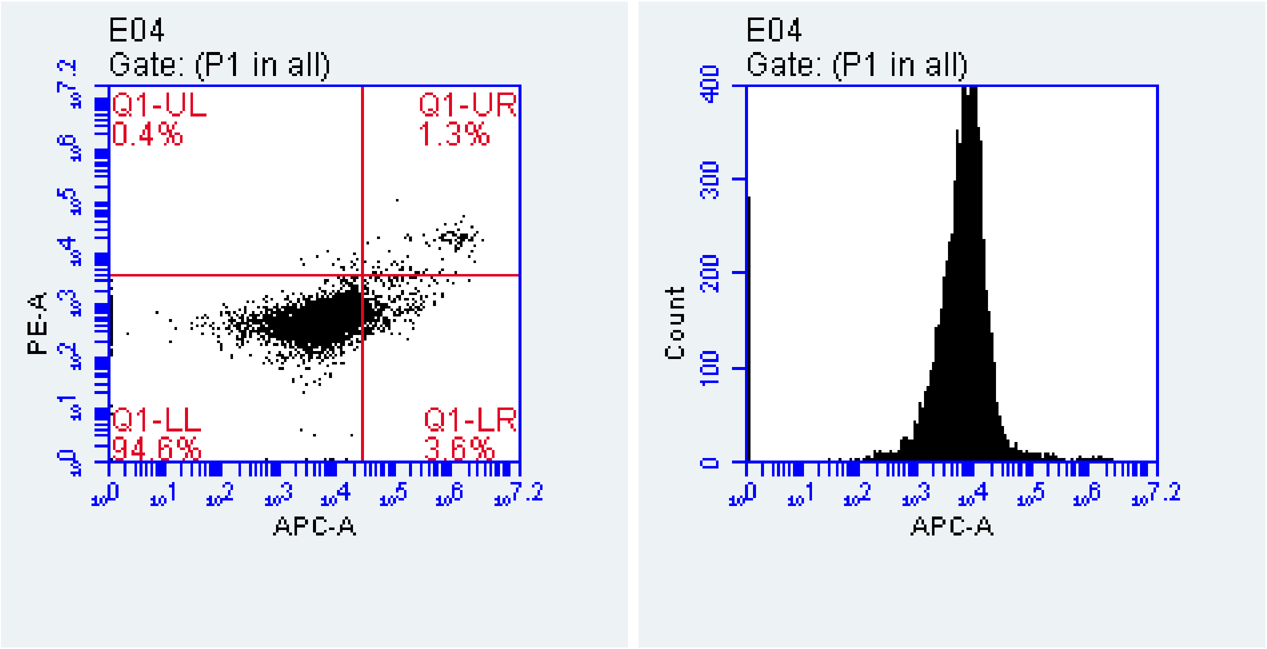
E04
Gate: (P1 in all)
Q1-UL
0.4%
Q1-UR
1.3%
Q1-LL
94.6%
Q1-LR
3.6%
PE-A
APC-A
E04
Gate: (P1 in all)
Count
APC-A

Figure S12C

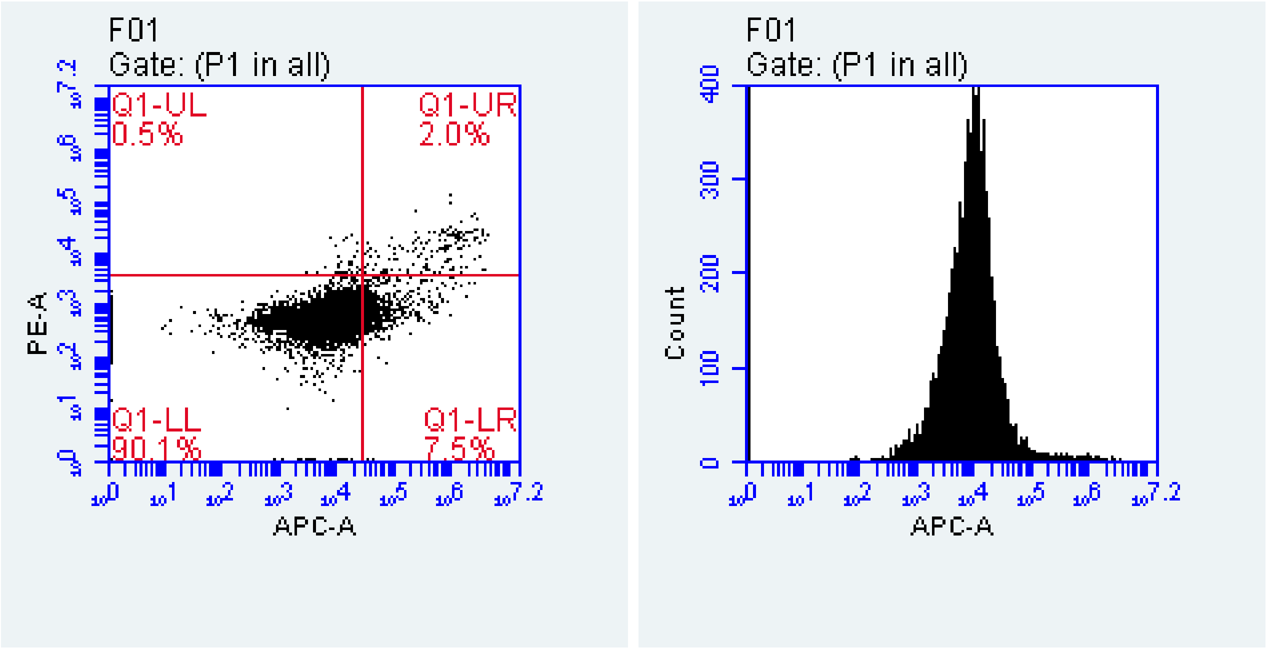
F01
Gate: (P1 in all)
Q1-UL
0.5%
Q1-UR
2.0%
Q1-LL
90.1%
Q1-LR
7.5%
PE-A
APC-A
F01
Gate: (P1 in all)
Count
APC-A

Figure S12C

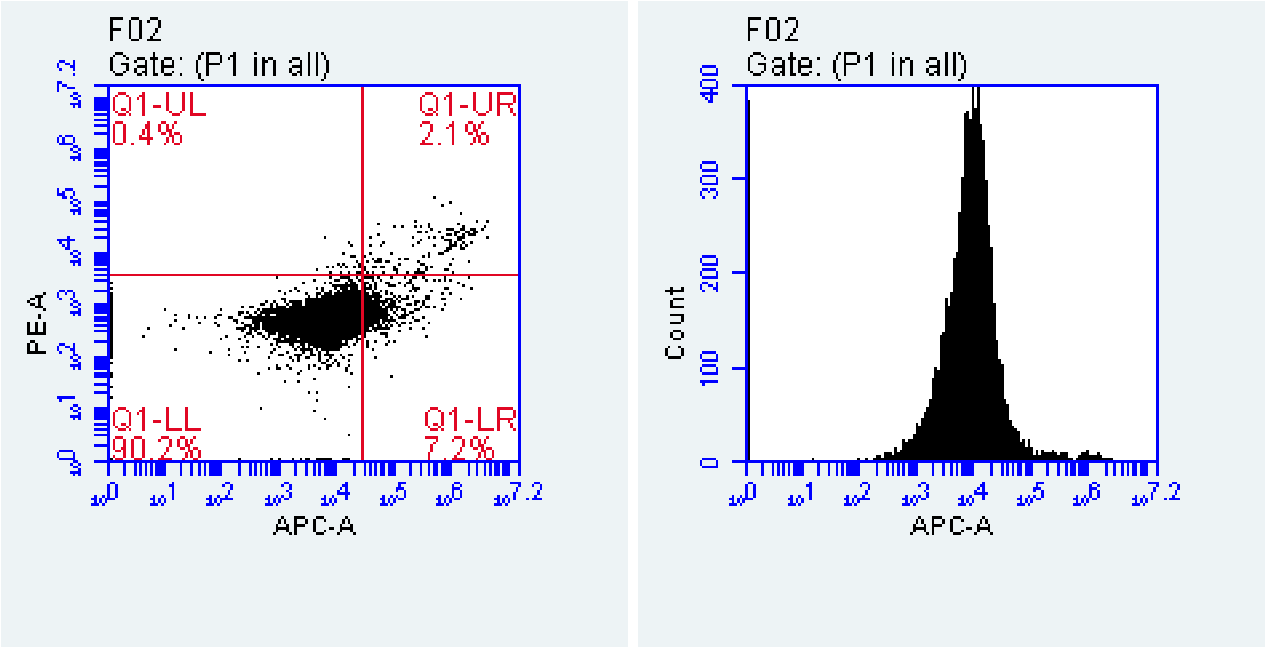
F02
Gate: (P1 in all)
Q1-UL
0.4%
Q1-UR
2.1%
Q1-LL
90.2%
Q1-LR
7.2%
PE-A
APC-A
F02
Gate: (P1 in all)
Count
APC-A

Figure S12C

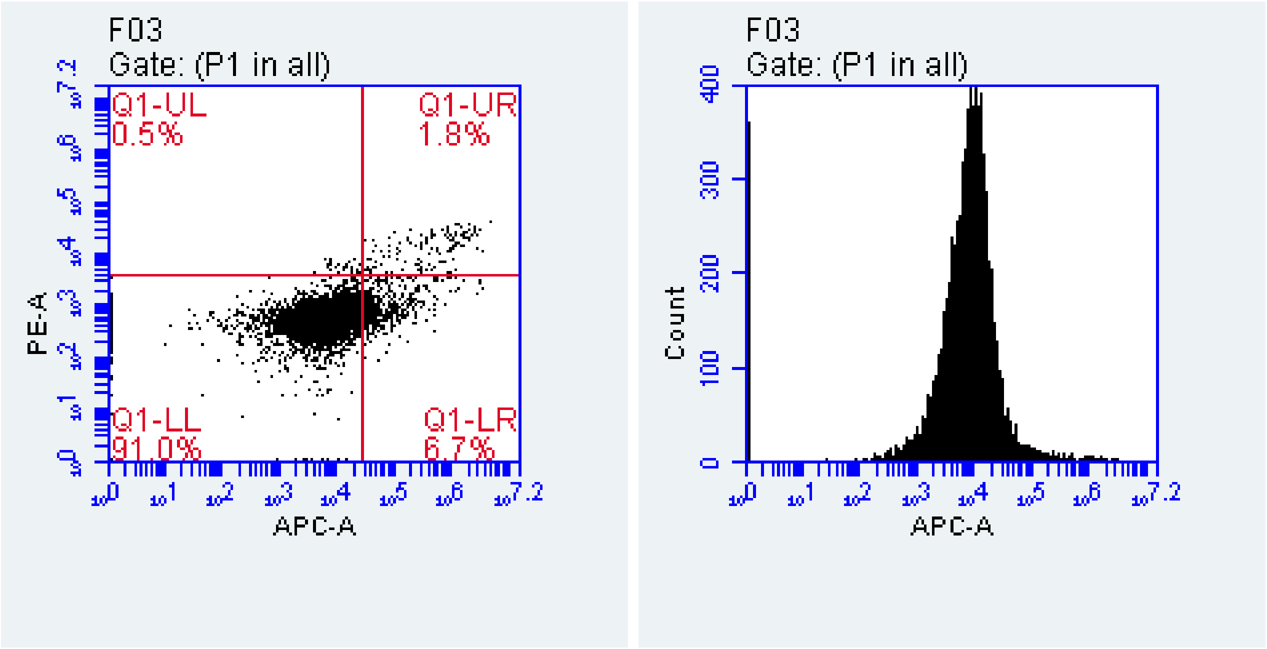

F03
Gate: (P1 in all)
Q1-UL
0.5%
Q1-UR
1.8%
Q1-LL
91.0%
Q1-LR
6.7%
PE-A
APC-A
F03
Gate: (P1 in all)
Count
APC-A

Figure S12C

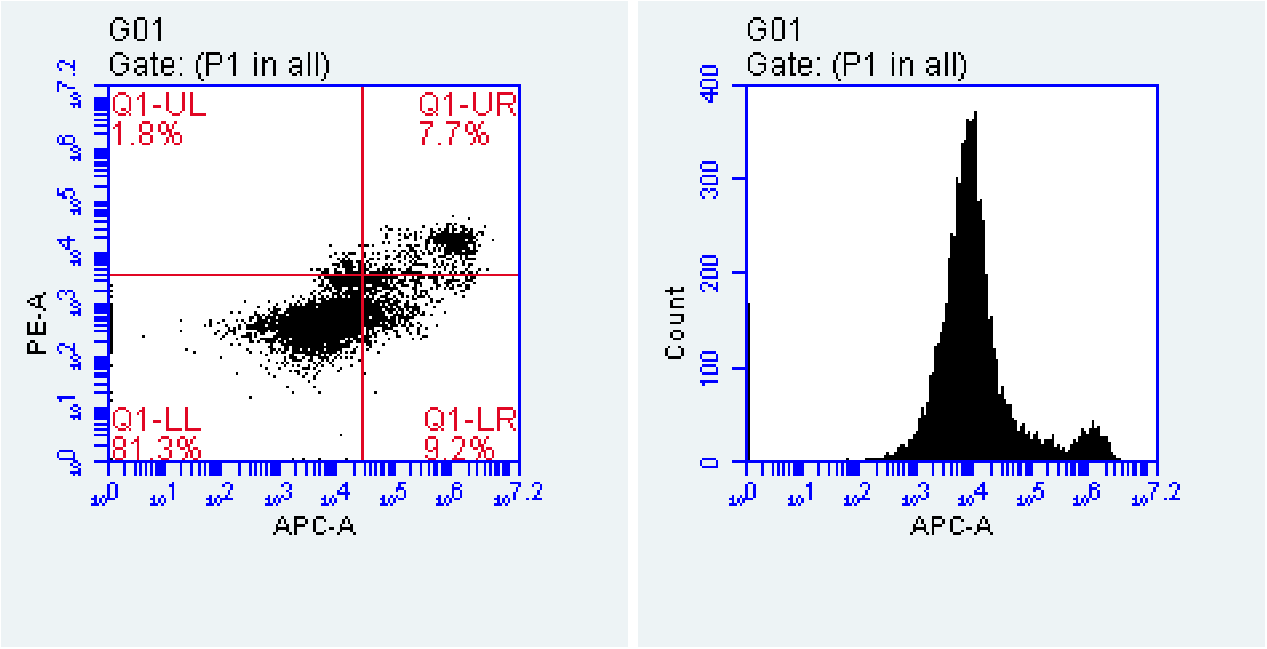
G01
Gate: (P1 in all)
Q1-UL
1.8%
Q1-UR
7.7%
Q1-LL
81.3%
Q1-LR
9.2%
PE-A
APC-A
G01
Gate: (P1 in all)
Count
APC-A

Figure S12C

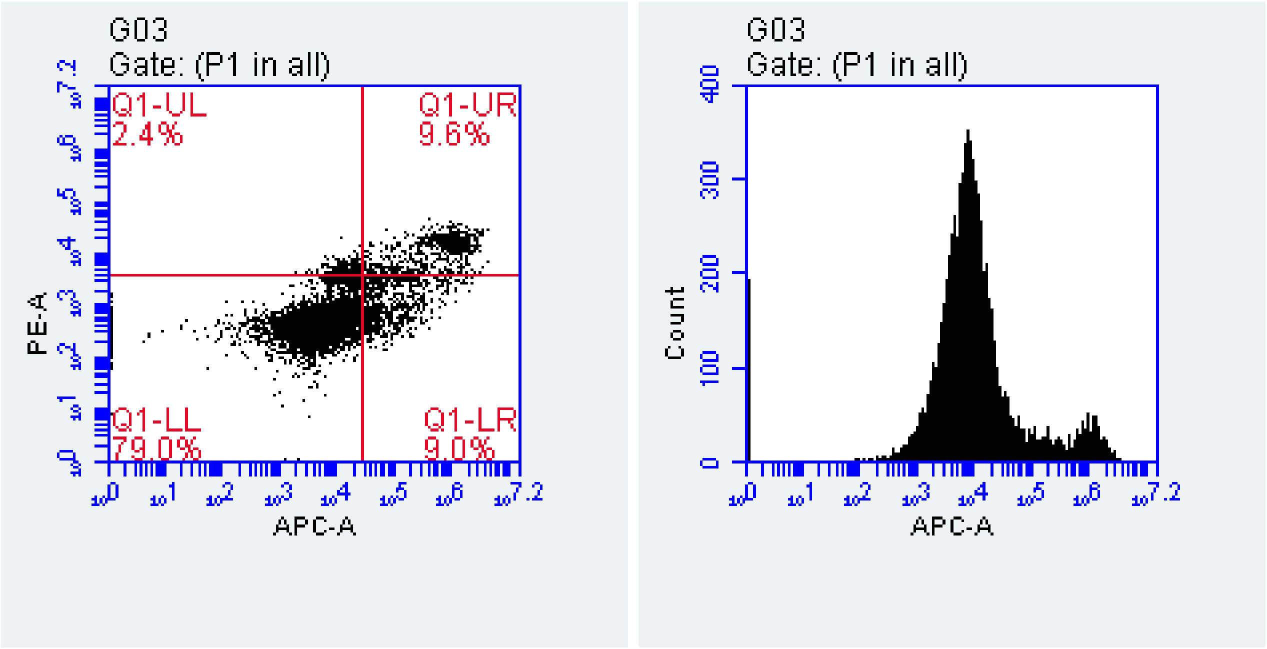
G03
Gate: (P1 in all)
Q1-UL
2.4%
Q1-UR
9.6%
Q1-LL
79.0%
Q1-LR
9.0%
PE-A
APC-A
G03
Gate: (P1 in all)
Count
APC-A

Figure S12C

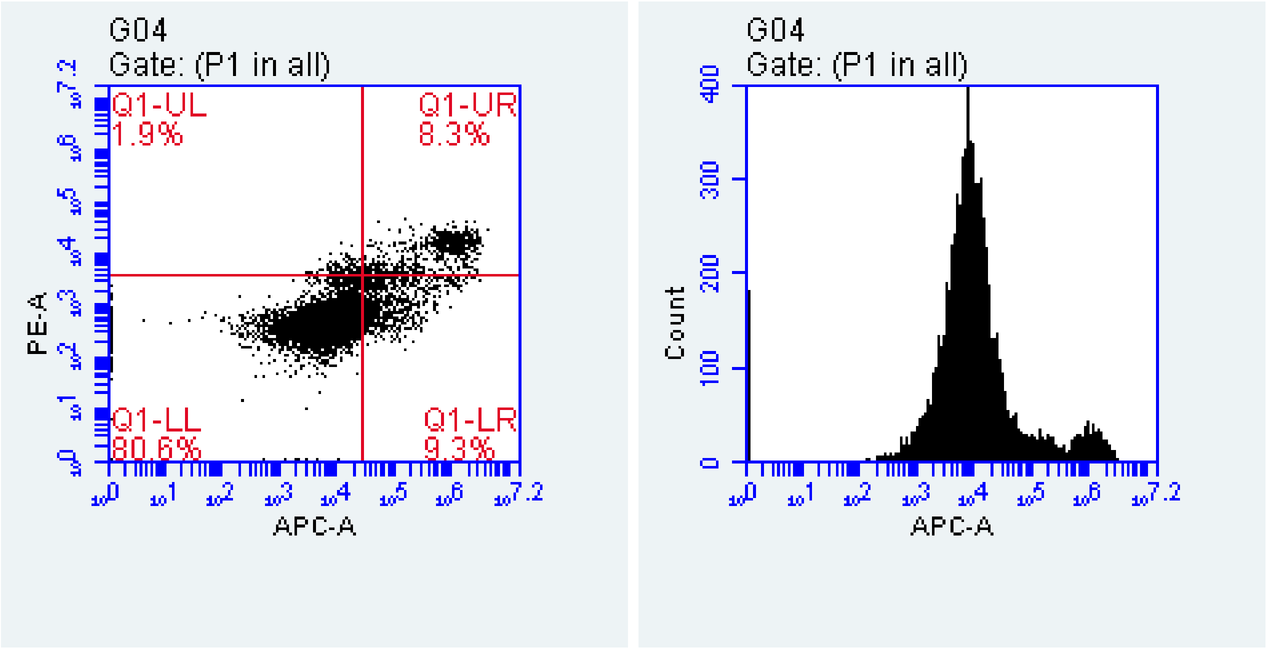
G04
Gate: (P1 in all)
Q1-UL
1.9%
Q1-UR
8.3%
Q1-LL
80.6%
Q1-LR
9.3%
PE-A
APC-A
G04
Gate: (P1 in all)
Count
APC-A

Figure S12C

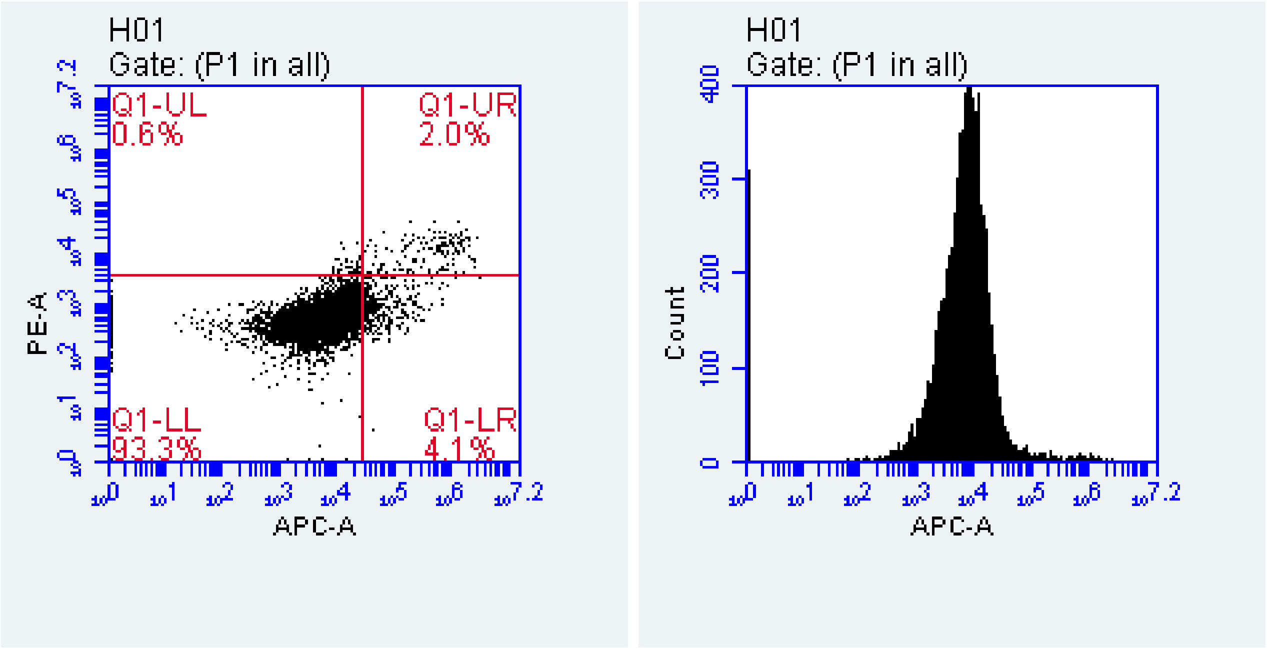
H01
Gate: (P1 in all)
Q1-UL
0.6%
Q1-UR
2.0%
Q1-LL
93.3%
Q1-LR
4.1%
PE-A
APC-A
H01
Gate: (P1 in all)
Count
APC-A

Figure S12C

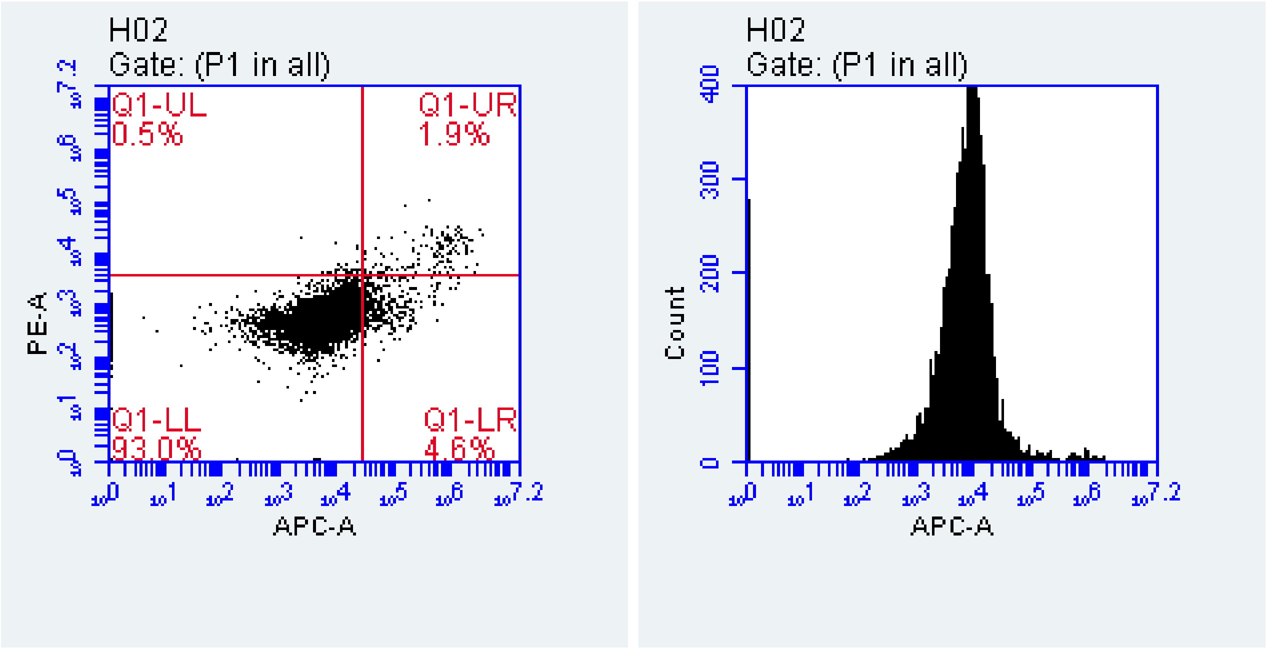
H02
Gate: (P1 in all)
Q1-UL
0.5%
Q1-UR
1.9%
Q1-LL
93.0%
Q1-LR
4.6%
PE-A
APC-A
H02
Gate: (P1 in all)
Count
APC-A

Figure S12C

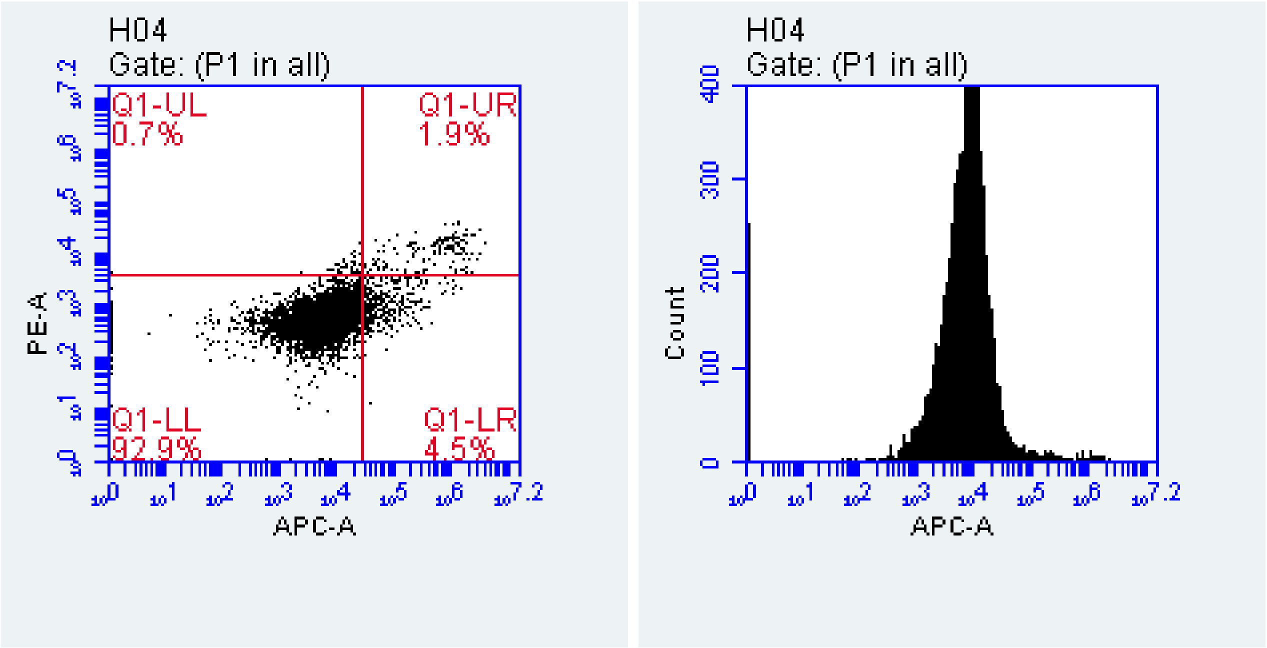
H04
Gate: (P1 in all)
Q1-UL
0.7%
Q1-UR
1.9%
Q1-LL
92.9%
Q1-LR
4.5%
PE-A
APC-A
H04
Gate: (P1 in all)
Count
APC-A

Figure S12C

Plot 3: E01: Gated on (P1 in all)	Count	Events / µL	% of This Plot	% of All	Mean APC-A	Mean PE-A	CV APC-A	CV PE-A	Median APC-A	Median PE-A
This Plot	8,000	2000	100.00%	59.04%	26,216.71	800.55	673.12%	268.35%		
Q1-UL	11	3	0.14%	0.08%	19,599.45	7,853.27	33.28%	88.05%		
Q1-UR	107	27	1.34%	0.79%	1,071,593.33	14,724.82	93.10%	76.95%		
Q1-LL	7,619	1905	95.24%	56.23%	7,395.59	581.28	64.34%	53.08%		
Q1-LR	263	66	3.29%	1.94%	146,428.13	1,192.78	183.73%	79.30%		

Figure S12C

Plot 4: E01: Gated on (P1 in all)	Count	Events / µL	% of This Plot	% of All	Mean APC-A	CV APC-A	Median APC-A
This Plot	8,000	2000	100.00%	59.04%	26,216.71	673.12%	

Figure S12C

Plot 3: E02: Gated on (P1 in all)	Count	Events / µL	% of This Plot	% of All	Mean APC-A	Mean PE-A	CV APC-A	CV PE-A	Median APC-A	Median PE-A
This Plot	8,000	2000	100.00%	56.09%	24,319.33	812.36	650.85%	311.54%		
Q1-UL	38	10	0.48%	0.27%	16,723.03	4,964.97	43.58%	19.64%		
Q1-UR	107	27	1.34%	0.75%	882,339.07	15,554.50	101.71%	99.87%		
Q1-LL	7,552	1888	94.40%	52.95%	7,429.04	568.81	77.50%	54.74%		
Q1-LR	303	76	3.79%	2.12%	143,250.13	1,155.92	215.17%	79.97%		

Figure S12C

Plot 4: E02: Gated on (P1 in all)	Count	Events / µL	% of This Plot	% of All	Mean APC-A	CV APC-A	Median APC-A
This Plot	8,000	2000	100.00%	56.09%	24,319.33	650.85%	

Plot 3: E03: Gated on (P1 in all)	Count	Events / µL	% of This Plot	% of All	Mean APC-A	Mean PE-A	CV APC-A	CV PE-A	Median APC-A	Median PE-A
This Plot	8,000	2667	100.00%	56.40%	20,602.45	766.56	631.17%	253.75%		
Q1-UL	28	9	0.35%	0.20%	15,718.96	6,589.75	49.03%	58.21%		
Q1-UR	95	32	1.19%	0.67%	740,722.91	14,292.66	110.84%	72.51%		
Q1-LL	7,596	2532	94.95%	53.55%	7,451.16	559.84	77.21%	55.37%		
Q1-LR	281	94	3.51%	1.98%	133,138.09	1,201.48	183.92%	72.66%		

Plot 4: E03: Gated on (P1 in all)	Count	Events / µL	% of This Plot	% of All	Mean APC-A	CV APC-A	Median APC-A
This Plot	8,000	2667	100.00%	56.40%	20,602.45	631.17%	

Plot 3: E04: Gated on (P1 in all)	Count	Events / µL	% of This Plot	% of All	Mean APC-A	Mean PE-A	CV APC-A	CV PE-A	Median APC-A	Median PE-A
This Plot	8,000	2000	100.00%	55.02%	25,108.88	817.50	632.19%	287.26%		
Q1-UL	35	9	0.44%	0.24%	16,418.83	5,988.20	51.05%	40.17%		
Q1-UR	104	26	1.30%	0.72%	1,032,166.30	16,318.40	85.10%	77.24%		
Q1-LL	7,570	1893	94.62%	52.07%	7,608.10	566.51	75.25%	54.82%		
Q1-LR	291	73	3.64%	2.00%	121,504.35	1,184.98	156.95%	74.09%		

Plot 4: E04: Gated on (P1 in all)	Count	Events / µL	% of This Plot	% of All	Mean APC-A	CV APC-A	Median APC-A
This Plot	8,000	2000	100.00%	55.02%	25,108.88	632.19%	

Plot 3: F01: Gated on (P1 in all)	Count	Events / µL	% of This Plot	% of All	Mean APC-A	Mean PE-A	CV APC-A	CV PE-A	Median APC-A	Median PE-A
This Plot	8,000	571	100.00%	46.67%	36,738.50	984.47	589.67%	316.58%		
Q1-UL	37	3	0.46%	0.22%	14,981.08	7,830.38	38.57%	69.39%		
Q1-UR	157	11	1.96%	0.92%	1,007,038.64	15,810.36	107.48%	98.76%		
Q1-LL	7,208	515	90.10%	42.05%	8,939.29	619.99	75.04%	53.91%		
Q1-LR	598	43	7.48%	3.49%	118,418.51	1,061.77	200.81%	69.64%		

Plot 4: F01: Gated on (P1 in all)	Count	Events / µL	% of This Plot	% of All	Mean APC-A	CV APC-A	Median APC-A
This Plot	8,000	571	100.00%	46.67%	36,738.50	589.67%	

Plot 3: F02: Gated on (P1 in all)	Count	Events / µL	% of This Plot	% of All	Mean APC-A	Mean PE-A	CV APC-A	CV PE-A	Median APC-A	Median PE-A
This Plot	8,000	2667	100.00%	52.79%	35,345.82	1,034.68	559.78%	321.51%		
Q1-UL	33	11	0.41%	0.22%	16,769.97	6,358.91	42.18%	58.92%		
Q1-UR	170	57	2.12%	1.12%	893,509.84	17,255.27	105.31%	88.91%		
Q1-LL	7,218	2406	90.22%	47.63%	9,046.61	625.59	73.84%	55.21%		
Q1-LR	579	193	7.24%	3.82%	112,293.72	1,068.59	198.31%	69.33%		

Plot 4: F02: Gated on (P1 in all)	Count	Events / µL	% of This Plot	% of All	Mean APC-A	CV APC-A	Median APC-A
This Plot	8,000	2667	100.00%	52.79%	35,345.82	559.78%	

Plot 3: F03: Gated on (P1 in all)	Count	Events / µL	% of This Plot	% of All	Mean APC-A	Mean PE-A	CV APC-A	CV PE-A	Median APC-A	Median PE-A
This Plot	8,000	2667	100.00%	53.12%	32,121.36	934.25	594.08%	247.54%		
Q1-UL	43	14	0.54%	0.29%	16,888.42	5,078.14	43.81%	22.19%		
Q1-UR	140	47	1.75%	0.93%	873,845.72	15,256.56	106.32%	59.08%		
Q1-LL	7,283	2428	91.04%	48.36%	8,789.95	623.82	74.16%	55.20%		
Q1-LR	534	178	6.68%	3.55%	130,878.32	1,079.54	259.93%	72.01%		

Plot 4: F03: Gated on (P1 in all)	Count	Events / µL	% of This Plot	% of All	Mean APC-A	CV APC-A	Median APC-A
This Plot	8,000	2667	100.00%	53.12%	32,121.36	594.08%	

Plot 3: F04: Gated on (P1 in all)	Count	Events / µL	% of This Plot	% of All	Mean APC-A	Mean PE-A	CV APC-A	CV PE-A	Median APC-A	Median PE-A
This Plot	8,000	2000	100.00%	49.80%	30,694.18	913.30	601.21%	254.74%		
Q1-UL	31	8	0.39%	0.19%	16,364.52	5,968.81	37.18%	55.45%		
Q1-UR	146	37	1.82%	0.91%	907,343.39	15,091.77	107.71%	57.38%		
Q1-LL	7,354	1839	91.92%	45.78%	8,564.18	602.88	74.08%	57.98%		
Q1-LR	469	117	5.86%	2.92%	105,742.04	1,032.78	164.79%	71.51%		

Plot 4: F04: Gated on (P1 in all)	Count	Events / µL	% of This Plot	% of All	Mean APC-A	CV APC-A	Median APC-A
This Plot	8,000	2000	100.00%	49.80%	30,694.18	601.21%	

Plot 3: G01: Gated on (P1 in all)	Count	Events / µL	% of This Plot	% of All	Mean APC-A	Mean PE-A	CV APC-A	CV PE-A	Median APC-A	Median PE-A
This Plot	8,000	348	100.00%	12.48%	104,267.18	1,786.24	366.32%	237.98%		
Q1-UL	141	6	1.76%	0.22%	15,510.78	5,416.90	42.64%	35.85%		
Q1-UR	619	27	7.74%	0.97%	1,036,385.67	14,158.76	83.16%	53.41%		
Q1-LL	6,503	283	81.29%	10.14%	8,123.83	572.18	74.71%	84.35%		
Q1-LR	737	32	9.21%	1.15%	186,700.73	1,412.41	200.27%	74.34%		

Plot 4: G01: Gated on (P1 in all)	Count	Events / μL	% of This Plot	% of All	Mean APC-A	CV APC-A	Median APC-A
This Plot	8,000	348	100.00%	12.48%	104,267.18	366.32%	

Plot 3: G02: Gated on (P1 in all)	Count	Events / µL	% of This Plot	% of All	Mean APC-A	Mean PE-A	CV APC-A	CV PE-A	Median APC-A	Median PE-A
This Plot	8,000	242	100.00%	8.99%	131,395.46	2,240.73	307.79%	215.25%		
Q1-UL	194	6	2.43%	0.22%	14,775.88	5,213.90	44.23%	34.56%		
Q1-UR	893	27	11.16%	1.00%	945,729.54	13,649.45	80.68%	53.34%		
Q1-LL	6,127	186	76.59%	6.89%	8,189.43	577.37	76.19%	96.06%		
Q1-LR	786	24	9.83%	0.88%	195,399.54	1,511.28	180.05%	72.32%		

Plot 4: G02: Gated on (P1 in all)	Count	Events / µL	% of This Plot	% of All	Mean APC-A	CV APC-A	Median APC-A
This Plot	8,000	242	100.00%	8.99%	131,395.46	307.79%	

Plot 3: G03: Gated on (P1 in all)	Count	Events / µL	% of This Plot	% of All	Mean APC-A	Mean PE-A	CV APC-A	CV PE-A	Median APC-A	Median PE-A
This Plot	8,000	286	100.00%	10.31%	113,707.77	2,029.01	340.65%	224.58%		
Q1-UL	195	7	2.44%	0.25%	15,775.52	5,488.13	42.69%	44.48%		
Q1-UR	765	27	9.56%	0.99%	959,137.13	13,680.25	85.25%	54.60%		
Q1-LL	6,320	226	79.00%	8.15%	7,943.14	572.15	77.72%	89.54%		
Q1-LR	720	26	9.00%	0.93%	170,340.83	1,500.66	178.97%	71.17%		

Plot 4: G03: Gated on (P1 in all)	Count	Events / µL	% of This Plot	% of All	Mean APC-A	CV APC-A	Median APC-A
This Plot	8,000	286	100.00%	10.31%	113,707.77	340.65%	

Plot 3: G04: Gated on (P1 in all)	Count	Events / µL	% of This Plot	% of All	Mean APC-A	Mean PE-A	CV APC-A	CV PE-A	Median APC-A	Median PE-A
This Plot	8,000	333	100.00%	11.92%	106,426.36	1,798.10	352.60%	230.44%		
Q1-UL	150	6	1.88%	0.22%	15,413.41	5,369.56	41.81%	33.85%		
Q1-UR	661	28	8.26%	0.98%	997,000.48	13,300.92	83.28%	54.99%		
Q1-LL	6,445	269	80.56%	9.60%	8,420.05	570.43	76.30%	84.06%		
Q1-LR	744	31	9.30%	1.11%	182,546.16	1,493.30	178.36%	71.55%		

Plot 4: G04: Gated on (P1 in all)	Count	Events / μL	% of This Plot	% of All	Mean APC-A	CV APC-A	Median APC-A
This Plot	8,000	333	100.00%	11.92%	106,426.36	352.60%	

Plot 3: H01: Gated on (P1 in all)	Count	Events / µL	% of This Plot	% of All	Mean APC-A	Mean PE-A	CV APC-A	CV PE-A	Median APC-A	Median PE-A
This Plot	8,000	2667	100.00%	50.74%	26,883.73	919.11	566.43%	224.53%		
Q1-UL	48	16	0.60%	0.30%	17,967.33	6,017.81	38.70%	46.03%		
Q1-UR	158	53	1.98%	1.00%	764,933.92	12,608.32	91.71%	59.33%		
Q1-LL	7,466	2489	93.32%	47.36%	7,887.95	629.04	75.68%	62.76%		
Q1-LR	328	109	4.10%	2.08%	105,050.02	1,144.91	214.92%	70.64%		

Plot 4: H01: Gated on (P1 in all)	Count	Events / µL	% of This Plot	% of All	Mean APC-A	CV APC-A	Median APC-A
This Plot	8,000	2667	100.00%	50.74%	26,883.73	566.43%	

Plot 3: H02: Gated on (P1 in all)	Count	Events / µL	% of This Plot	% of All	Mean APC-A	Mean PE-A	CV APC-A	CV PE-A	Median APC-A	Median PE-A
This Plot	8,000	2667	100.00%	49.64%	28,230.01	937.47	555.33%	268.04%		
Q1-UL	43	14	0.54%	0.27%	15,966.98	5,714.51	40.56%	62.41%		
Q1-UR	154	51	1.92%	0.96%	809,383.80	13,593.08	91.54%	88.24%		
Q1-LL	7,438	2479	92.98%	46.15%	8,133.35	635.58	73.16%	63.37%		
Q1-LR	365	122	4.56%	2.26%	109,623.33	1,186.96	168.21%	70.27%		

Plot 4: H02: Gated on (P1 in all)	Count	Events / μL	% of This Plot	% of All	Mean APC-A	CV APC-A	Median APC-A
This Plot	8,000	2667	100.00%	49.64%	28,230.01	555.33%	

Plot 3: H03: Gated on (P1 in all)	Count	Events / µL	% of This Plot	% of All	Mean APC-A	Mean PE-A	CV APC-A	CV PE-A	Median APC-A	Median PE-A
This Plot	8,000	4000	100.00%	50.43%	26,382.64	915.74	556.47%	235.09%		
Q1-UL	42	21	0.52%	0.26%	13,713.71	5,567.24	49.91%	31.62%		
Q1-UR	147	74	1.84%	0.93%	816,690.99	14,017.81	83.51%	55.26%		
Q1-LL	7,509	3755	93.86%	47.33%	7,910.57	624.89	75.26%	62.62%		
Q1-LR	302	151	3.78%	1.90%	102,752.05	1,123.08	154.77%	69.41%		

Plot 4: H03: Gated on (P1 in all)	Count	Events / µL	% of This Plot	% of All	Mean APC-A	CV APC-A	Median APC-A
This Plot	8,000	4000	100.00%	50.43%	26,382.64	556.47%	

Plot 3: H04: Gated on (P1 in all)	Count	Events / µL	% of This Plot	% of All	Mean APC-A	Mean PE-A	CV APC-A	CV PE-A	Median APC-A	Median PE-A
This Plot	8,000	2667	100.00%	50.57%	29,213.22	912.43	573.83%	218.94%		
Q1-UL	57	19	0.71%	0.36%	16,810.53	5,365.23	44.46%	38.89%		
Q1-UR	149	50	1.86%	0.94%	881,265.21	12,570.89	92.23%	59.13%		
Q1-LL	7,433	2478	92.91%	46.98%	8,205.41	630.25	73.20%	63.27%		
Q1-LR	361	120	4.51%	2.28%	112,044.87	1,207.68	163.79%	69.87%		

Plot 4: H04: Gated on (P1 in all)	Count	Events / μL	% of This Plot	% of All	Mean APC-A	CV APC-A	Median APC-A
This Plot	8,000	2667	100.00%	50.57%	29,213.22	573.83%	

Current Date: 26-11?-2021
Instrument Serial Number: 6735146202
Software Name: InCyte
Version Number: 3.1

Well	Sample ID	Date	R1.Percent Percent for R1 (%)	R2.Percent.UL Percent for R2 gated by P01.R1 (%)	R2.Percent.UR Percent for R2 gated by P01.R1 (%)	R2.Percent.LL Percent for R2 gated by P01.R1 (%)	R2.Percent.LR Percent for R2 gated by P01.R1 (%)	R3.Percent Percent for R3 gated by P01.R1 (%)
E02	shCtrl-1	11.26.2021	17.67690159	76.86	2.42	18.38	2.34	4.78
E03	shCtrl-2	11.26.2021	16.1922342	77.21	2.54	18.1	2.15	4.69
E04	shCtrl-3	11.26.2021	14.31331854	76.09	2.5	19.1	2.31	4.82
H01	shCDKL3-1	11.26.2021	24.03961729	77.63	6.12	12.58	3.67	9.82
H02	shCDKL3-2	11.26.2021	23.03563613	77.08	6.26	12.73	3.93	10.21
H03	shCDKL3-3	11.26.2021	18.2258917	76.92	6.97	11.65	4.46	11.47

Well Number: H01 Sample ID: H01

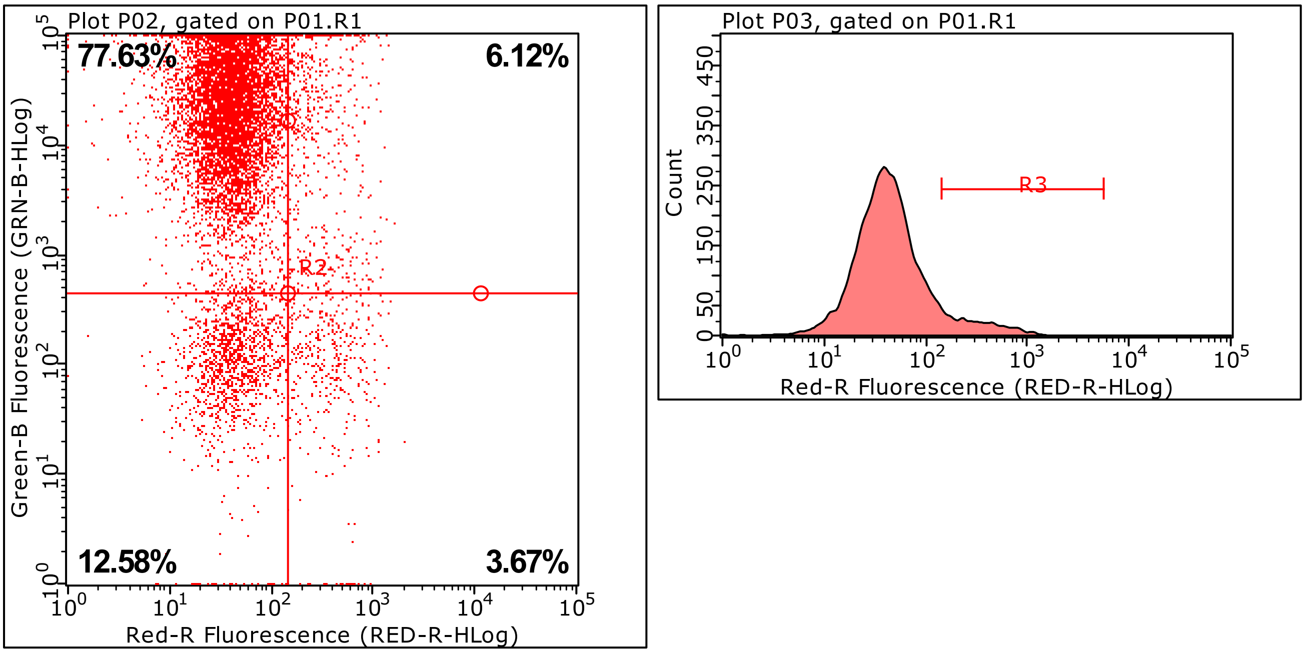

Figure 2C
DU 145 shCDKL3-1

Well	Sample ID	Date	R1.Percent Percent for R1 (%)	R2.Percent.UL Percent for R2 gated by P01.R1 (%)	R2.Percent.UR Percent for R2 gated by P01.R1 (%)	R2.Percent.LL Percent for R2 gated by P01.R1 (%)
H01	H01	11.26.2021	24.04	77.63	6.12	12.58

Well	R2.Percent.LR Percent for R2 gated by P01.R1 (%)	R3.Percent Percent for R3 gated by P01.R1 (%)
H01	3.67	9.82

Well Number: H02 Sample ID: H02

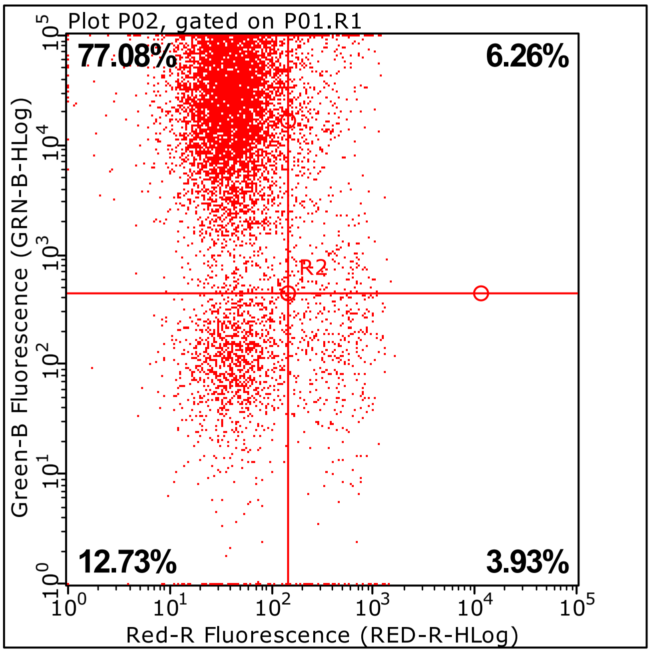

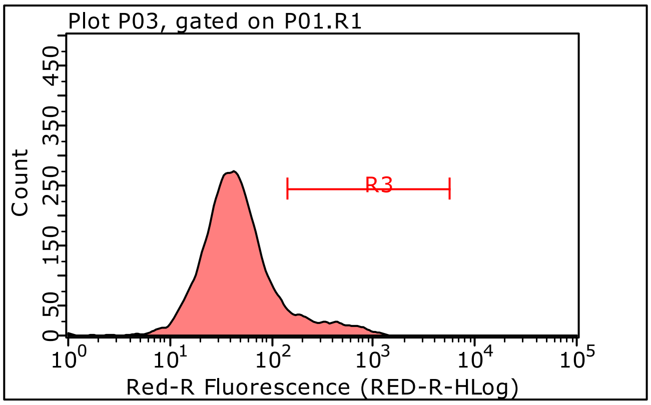

Figure 2C
DU 145 shCDKL3-2

Well	Sample ID	Date	R1.Percent Percent for R1 (%)	R2.Percent.UL Percent for R2 gated by P01.R1 (%)	R2.Percent.UR Percent for R2 gated by P01.R1 (%)	R2.Percent.LL Percent for R2 gated by P01.R1 (%)
H02	H02	11.26.2021	23.04	77.08	6.26	12.73

Well	R2.Percent.LR Percent for R2 gated by P01.R1 (%)	R3.Percent Percent for R3 gated by P01.R1 (%)
H02	3.93	10.21

Well Number: H03 Sample ID: H03

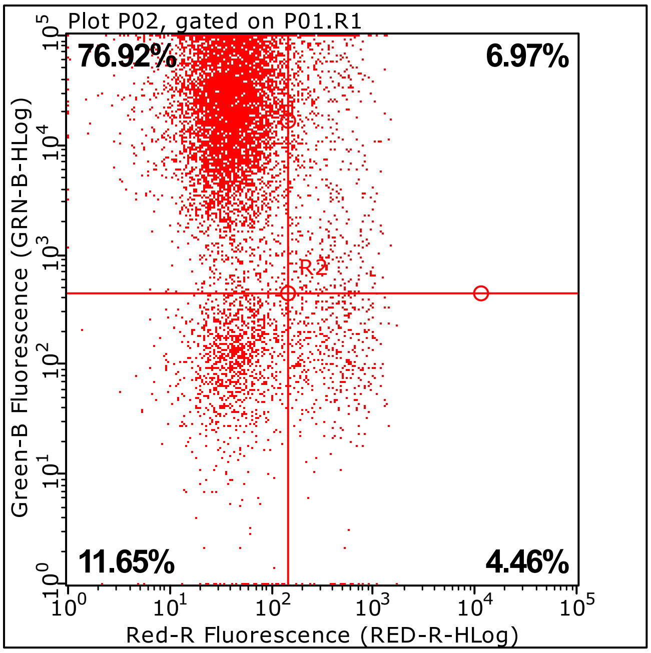

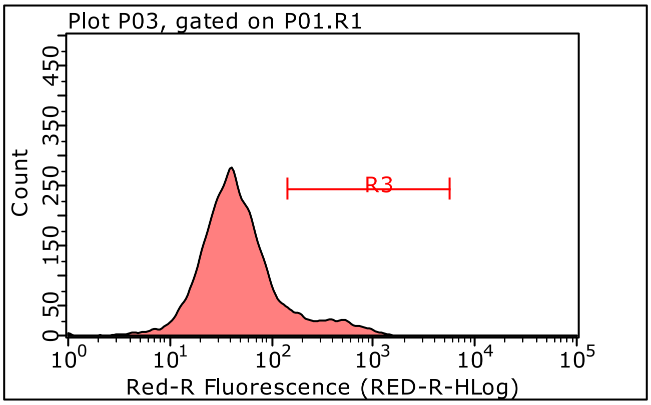

Figure 2C
DU 145 shCDKL3-3

Well	Sample ID	Date	R1.Percent Percent for R1 (%)	R2.Percent.UL Percent for R2 gated by P01.R1 (%)	R2.Percent.UR Percent for R2 gated by P01.R1 (%)	R2.Percent.LL Percent for R2 gated by P01.R1 (%)
H03	H03	11.26.2021	18.23	76.92	6.97	11.65

Well	R2.Percent.LR Percent for R2 gated by P01.R1 (%)	R3.Percent Percent for R3 gated by P01.R1 (%)
H03	4.46	11.47

Well Number: E02 Sample ID: E02

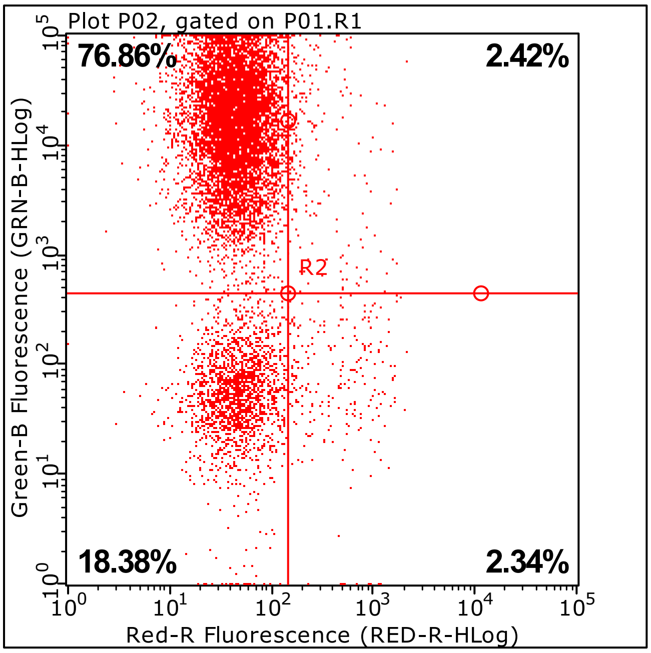

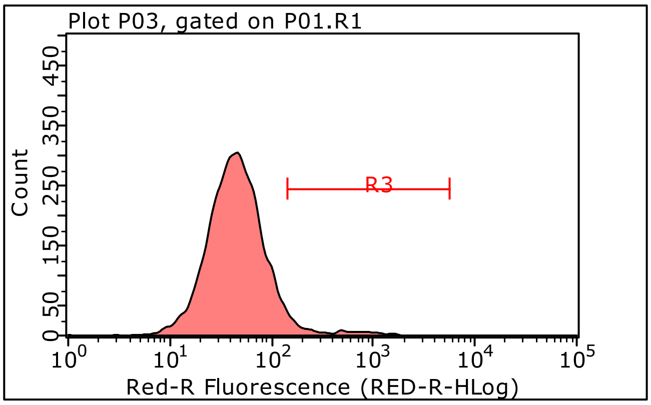

Figure 2C
DU 145 shCtrl-1

Well	Sample ID	Date	R1.Percent Percent for R1 (%)	R2.Percent.UL Percent for R2 gated by P01.R1 (%)	R2.Percent.UR Percent for R2 gated by P01.R1 (%)	R2.Percent.LL Percent for R2 gated by P01.R1 (%)
E02	E02	11.26.2021	17.68	76.86	2.42	18.38

Well	R2.Percent.LR Percent for R2 gated by P01.R1 (%)	R3.Percent Percent for R3 gated by P01.R1 (%)
E02	2.34	4.78

Well Number: E03 Sample ID: E03

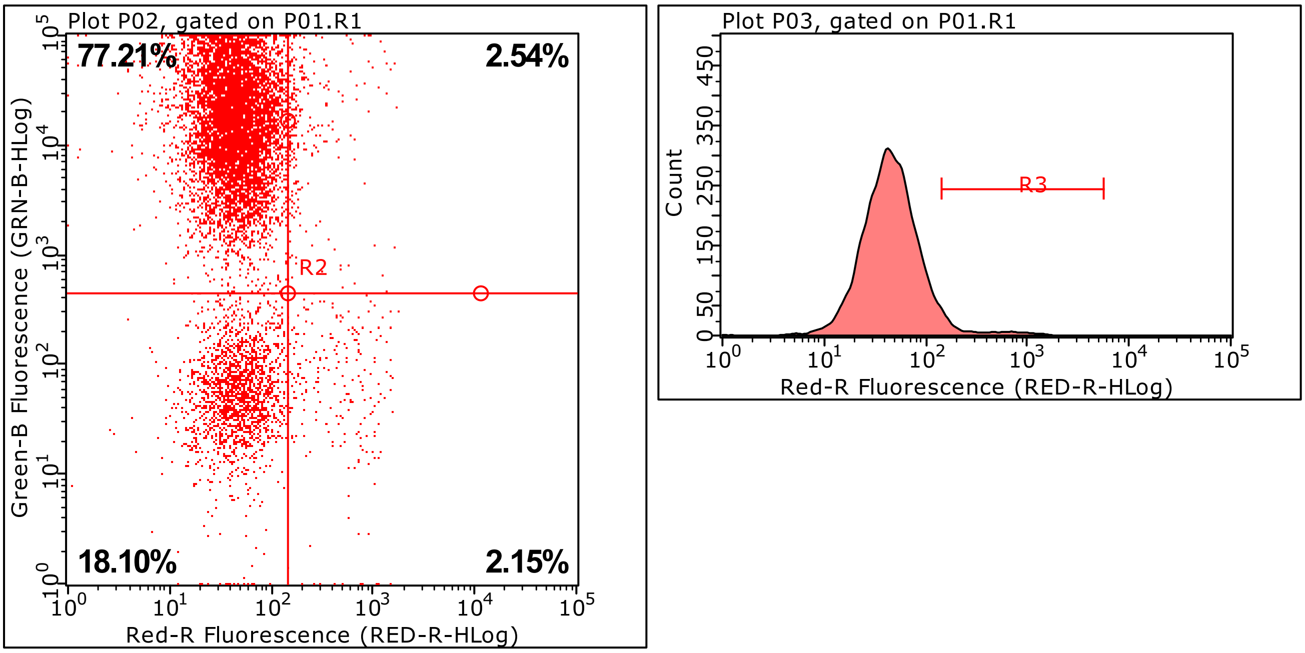

Figure 2C
DU 145 shCtrl-2

Well	Sample ID	Date	R1.Percent Percent for R1 (%)	R2.Percent.UL Percent for R2 gated by P01.R1 (%)	R2.Percent.UR Percent for R2 gated by P01.R1 (%)	R2.Percent.LL Percent for R2 gated by P01.R1 (%)
E03	E03	11.26.2021	16.19	77.21	2.54	18.10

Well	R2.Percent.LR Percent for R2 gated by P01.R1 (%)	R3.Percent Percent for R3 gated by P01.R1 (%)
E03	2.15	4.69

Well Number: E04 Sample ID: E04

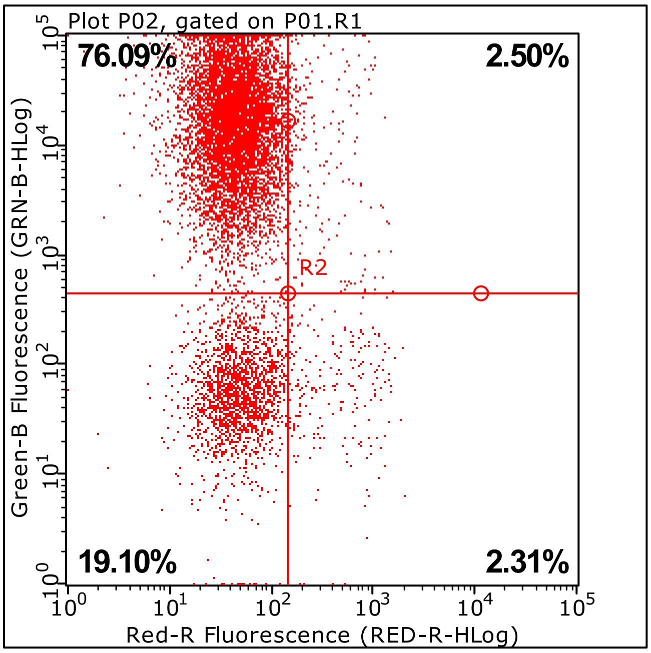

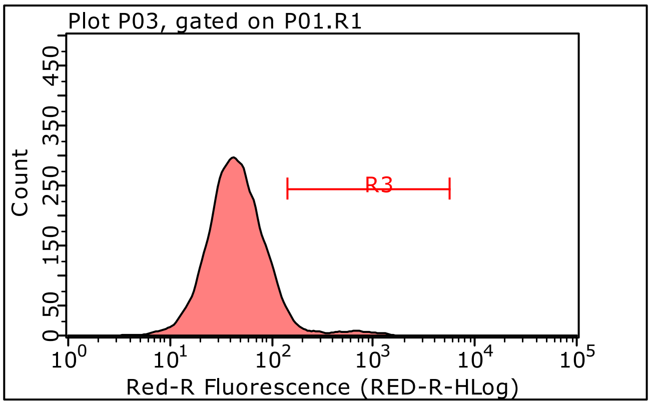

Figure 2C
DU 145 shCtrl-3

Well	Sample ID	Date	R1.Percent Percent for R1 (%)	R2.Percent.UL Percent for R2 gated by P01.R1 (%)	R2.Percent.UR Percent for R2 gated by P01.R1 (%)	R2.Percent.LL Percent for R2 gated by P01.R1 (%)
E04	E04	11.26.2021	14.31	76.09	2.50	19.10

Well	R2.Percent.LR Percent for R2 gated by P01.R1 (%)	R3.Percent Percent for R3 gated by P01.R1 (%)
E04	2.31	4.82

Current Date: 26-11?-2021
Instrument Serial Number: 6735146202
Software Name: InCyte
Version Number: 3.1

Well	Sample ID	Date	R1.Percent Percent for R1 (%)	R2.Percent.UL Percent for R2 gated by P01.R1	R2.Percent.UR Percent for R2 gated by P01.R1 (%)	R2.Percent.LL Percent for R2 gated by P01.R1	R2.Percent.LR Percent for R2 gated by P01.R1 (%)	R3.Percent Percent for R3 gated by P01.R1 (%)
C01	shCtrl-1	11.26.2021	47.381342	72.3565534	2.542698139	22.7691422	2.331606218	4.87430436
C02	shCtrl-2	11.26.2021	36.110918	72.6135489	2.723248653	22.3056197	2.357582756	5.08083141
C03	shCtrl-3	11.26.2021	41.882986	72.6950014	2.782308357	22.3544085	2.168281685	4.95059004
D01	shCDKL3-1	11.26.2021	49.903865	87.0834136	7.358890387	4.80639569	0.751300327	8.11019072
D03	shCDKL3-2	11.26.2021	42.162425	85.928489	8.756247597	4.21953095	1.095732411	9.85198001
D04	shCDKL3-3	11.26.2021	40.956964	85.90966	8.860637581	4.19917172	1.030530675	9.89116826

Well Number: D01 Sample ID: D01

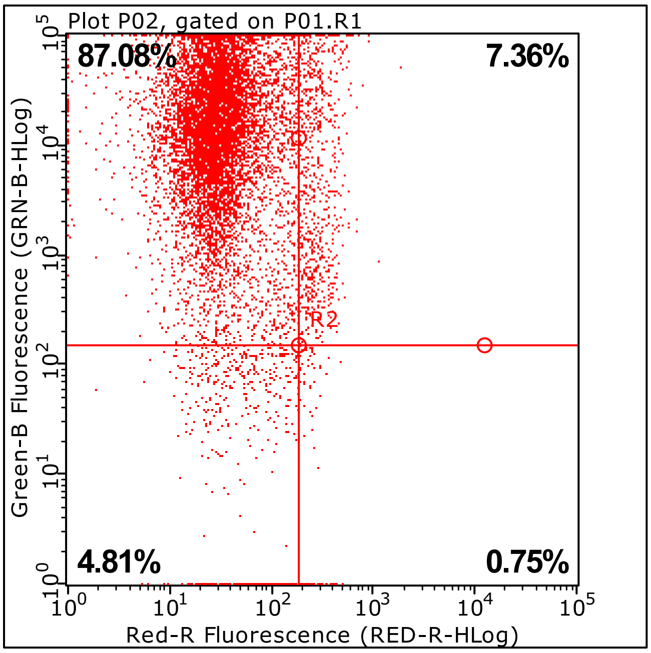

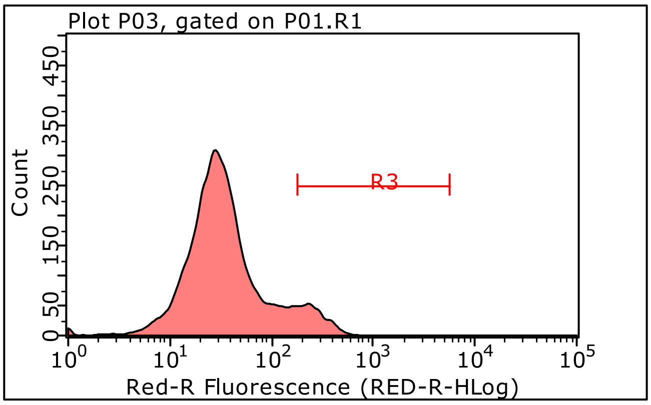

Figure 2C
PC-3 shCDKL3-1

Well	Sample ID	Date	R1.Percent Percent for R1 (%)	R2.Percent.UL Percent for R2 gated by P01.R1 (%)	R2.Percent.UR Percent for R2 gated by P01.R1 (%)	R2.Percent.LL Percent for R2 gated by P01.R1 (%)
D01	D01	11.26.2021	49.90	87.08	7.36	4.81

Well	R2.Percent.LR Percent for R2 gated by P01.R1 (%)	R3.Percent Percent for R3 gated by P01.R1 (%)
D01	0.75	8.11

Well Number: D03 Sample ID: D03

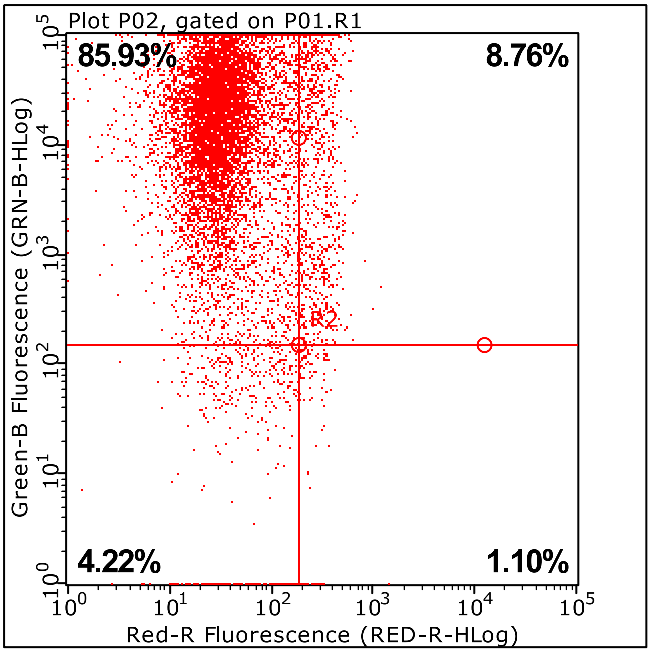

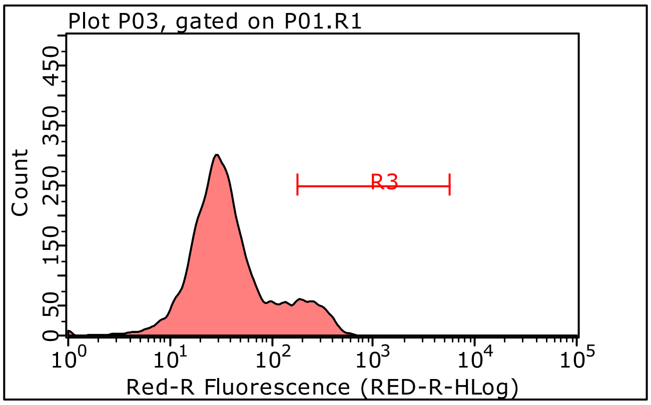

Figure 2C
PC-3 shCDKL3-2

Well	Sample ID	Date	R1.Percent Percent for R1 (%)	R2.Percent.UL Percent for R2 gated by P01.R1 (%)	R2.Percent.UR Percent for R2 gated by P01.R1 (%)	R2.Percent.LL Percent for R2 gated by P01.R1 (%)
D03	D03	11.26.2021	42.16	85.93	8.76	4.22

Well	R2.Percent.LR Percent for R2 gated by P01.R1 (%)	R3.Percent Percent for R3 gated by P01.R1 (%)
D03	1.10	9.85

Well Number: D04 Sample ID: D04

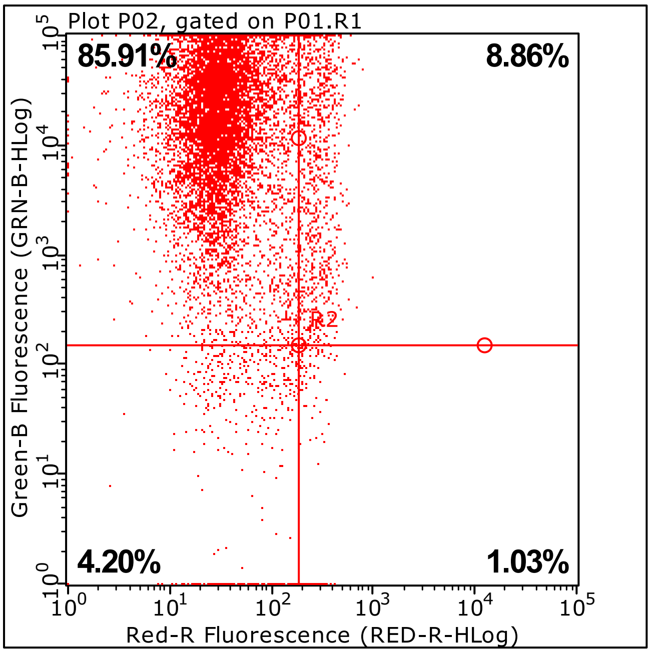

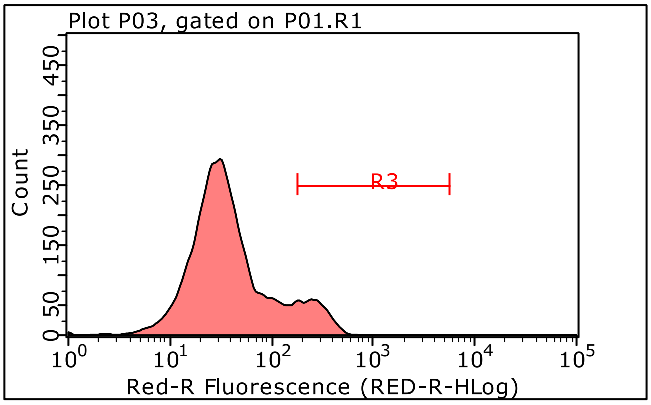

Figure 2C
PC-3 shCDKL3-3

Well	Sample ID	Date	R1.Percent Percent for R1 (%)	R2.Percent.UL Percent for R2 gated by P01.R1 (%)	R2.Percent.UR Percent for R2 gated by P01.R1 (%)	R2.Percent.LL Percent for R2 gated by P01.R1 (%)
D04	D04	11.26.2021	40.96	85.91	8.86	4.20

Well	R2.Percent.LR Percent for R2 gated by P01.R1 (%)	R3.Percent Percent for R3 gated by P01.R1 (%)
D04	1.03	9.89

Well Number: C01 Sample ID: C01

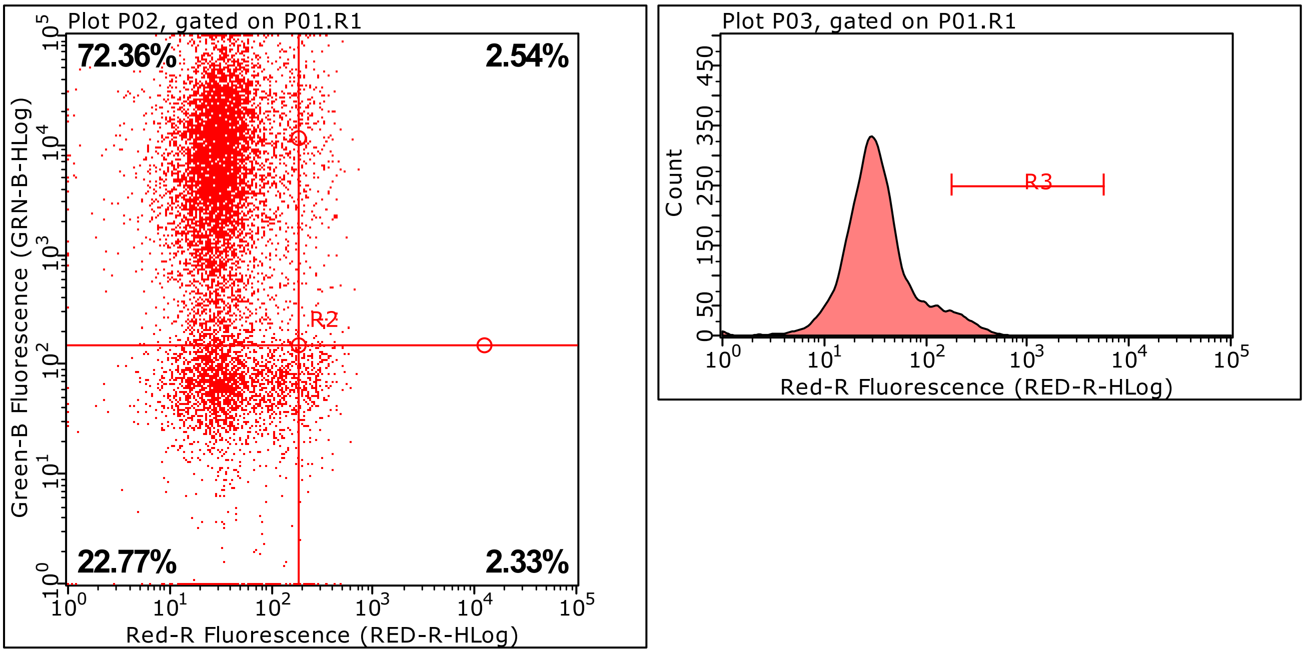

Figure 2C
PC-3 shCtrl-1

Well	Sample ID	Date	R1.Percent Percent for R1 (%)	R2.Percent.UL Percent for R2 gated by P01.R1 (%)	R2.Percent.UR Percent for R2 gated by P01.R1 (%)	R2.Percent.LL Percent for R2 gated by P01.R1 (%)
C01	C01	11.26.2021	47.38	72.36	2.54	22.77

Well	R2.Percent.LR Percent for R2 gated by P01.R1 (%)	R3.Percent Percent for R3 gated by P01.R1 (%)
C01	2.33	4.87

Well Number: C02 Sample ID: C02

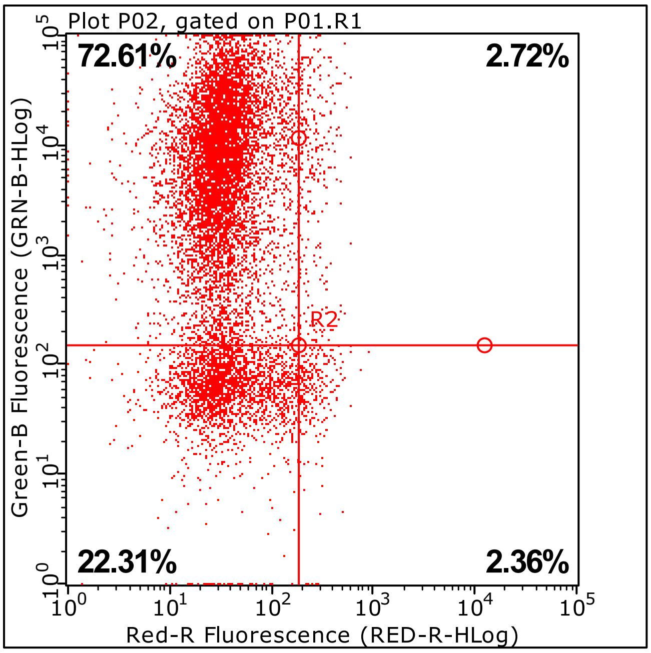

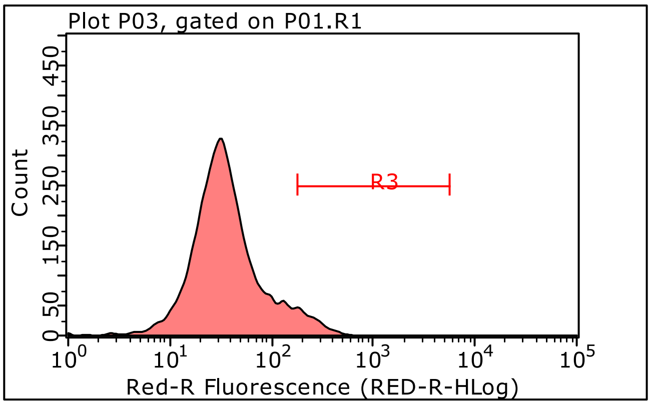

Figure 2C
PC-3 shCtrl-2

Well	Sample ID	Date	R1.Percent Percent for R1 (%)	R2.Percent.UL Percent for R2 gated by P01.R1 (%)	R2.Percent.UR Percent for R2 gated by P01.R1 (%)	R2.Percent.LL Percent for R2 gated by P01.R1 (%)
C02	C02	11.26.2021	36.11	72.61	2.72	22.31

Well	R2.Percent.LR Percent for R2 gated by P01.R1 (%)	R3.Percent Percent for R3 gated by P01.R1 (%)
C02	2.36	5.08

Well Number: C03 Sample ID: C03

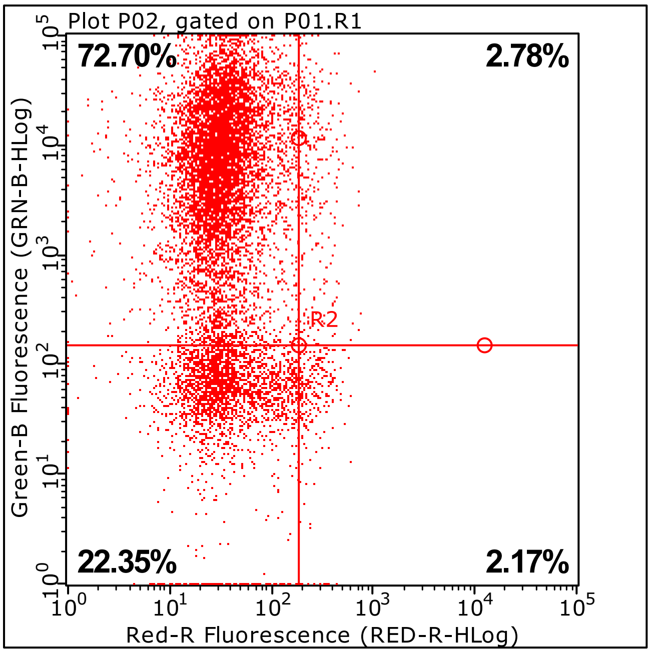

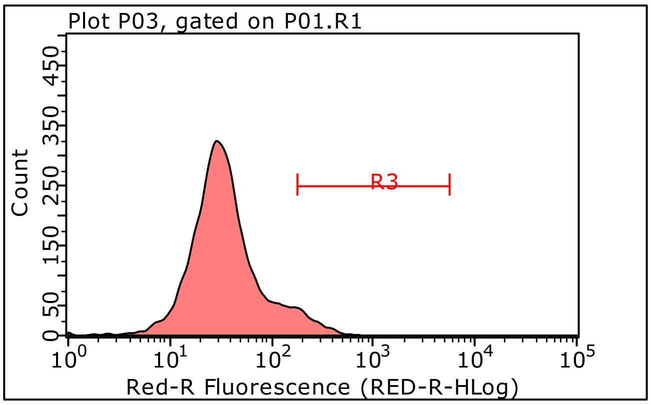

Figure 2C
PC-3 shCtrl-3

Well	Sample ID	Date	R1.Percent Percent for R1 (%)	R2.Percent.UL Percent for R2 gated by P01.R1 (%)	R2.Percent.UR Percent for R2 gated by P01.R1 (%)	R2.Percent.LL Percent for R2 gated by P01.R1 (%)
C03	C03	11.26.2021	41.88	72.70	2.78	22.35

Well	R2.Percent.LR Percent for R2 gated by P01.R1 (%)	R3.Percent Percent for R3 gated by P01.R1 (%)
C03	2.17	4.95

Figure 4F and S7C

DU 145

Current Date: 05-2?-2022
Instrument Serial Number: 6735146202
Software Name: InCyte
Version Number: 3.1

Well	Sample ID	Date	R2.Percent.UL Percent for R2 gated by P01.R1 (%)	R2.Percent.UR Percent for R2 gated by P01.R1 (%)	R2.Percent.LL Percent for R2 gated by P01.R1 (%)	R2.Percent.LR Percent for R2 gated by P01.R1 (%)	R3.Percent Percent for R3 gated by P01.R1 (%)
A02	shCtrl-1	02.05.2022	75.94833723	3.71199024	19.5464253	0.793247229	4.53574697
A03	shCtrl-2	02.05.2022	76.43947101	3.13326551	19.8168871	0.610376399	3.75381485
A04	shCtrl-3	02.05.2022	77.25837662	3.14695998	18.9326815	0.661981872	3.80894185
E01	shCDKL3-1	02.05.2022	74.84787018	6.72413793	17.0182556	1.409736308	8.13387424
E02	shCDKL3-2	02.05.2022	75.85221474	5.96040008	17.1361502	1.051234946	7.05245969
E03	shCDKL3-3	02.05.2022	75.36099248	6.32499492	17.2056132	1.108399431	7.45373195
C02	shCDKL3+ERK激活剂-	02.05.2022	78.13773431	4.88997555	16.2693562	0.702933985	5.61328443
C03	shCDKL3+ERK激活剂-	02.05.2022	77.9248549	4.68384075	16.5767234	0.814581	5.51878627
C04	shCDKL3+ERK激活剂-	02.05.2022	77.13733862	4.75754803	17.200366	0.904747382	5.70295822

Well Number: C02 Sample ID: C02

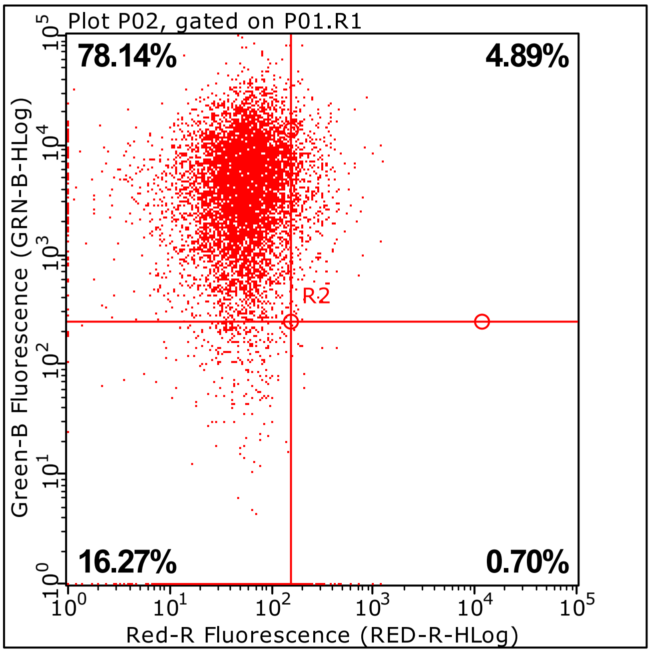

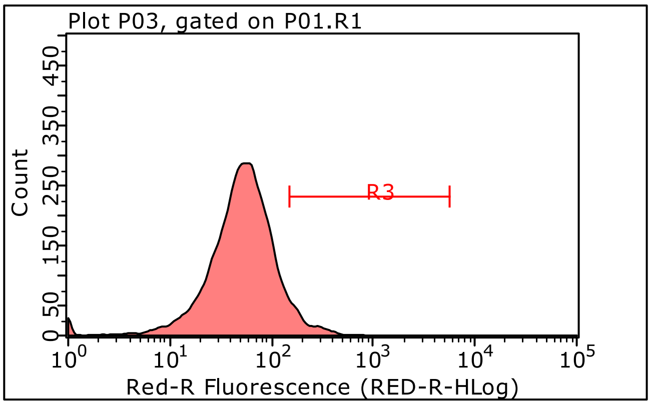

Figure 4F and S7C

DU 145
shCDKL3+ERK activator-1

Well	Sample ID	Date	R2.Percent.UL Percent for R2 gated by P01.R1 (%)	R2.Percent.UR Percent for R2 gated by P01.R1 (%)	R2.Percent.LL Percent for R2 gated by P01.R1 (%)
C02	C02	02.05.2022	78.14	4.89	16.27

Well	R2.Percent.LR Percent for R2 gated by P01.R1 (%)	R3.Percent Percent for R3 gated by P01.R1 (%)
C02	0.70	5.61

Well Number: C03 Sample ID: C03

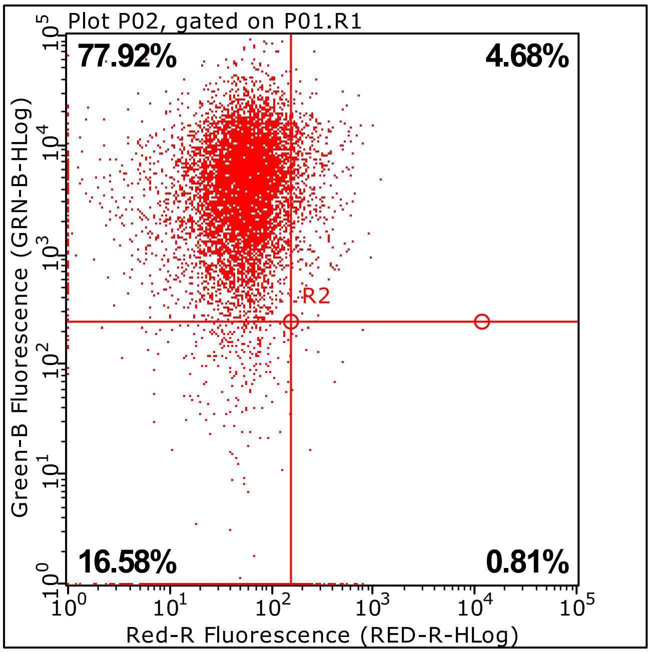

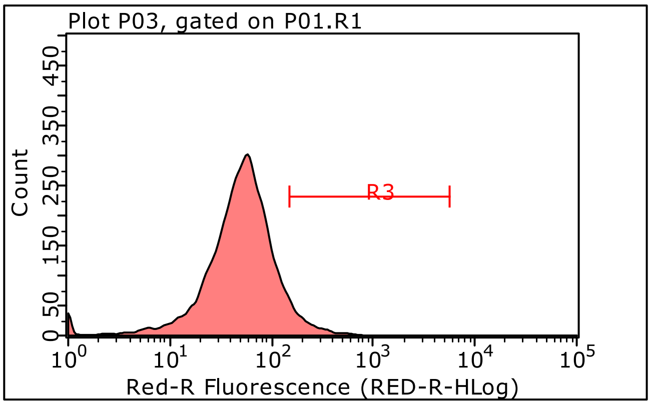

Figure 4F and S7C

DU 145
shCDKL3+ERK activator-2

Well	Sample ID	Date	R2.Percent.UL Percent for R2 gated by P01.R1 (%)	R2.Percent.UR Percent for R2 gated by P01.R1 (%)	R2.Percent.LL Percent for R2 gated by P01.R1 (%)
C03	C03	02.05.2022	77.92	4.68	16.58

Well	R2.Percent.LR Percent for R2 gated by P01.R1 (%)	R3.Percent Percent for R3 gated by P01.R1 (%)
C03	0.81	5.52

Well Number: C04 Sample ID: C04

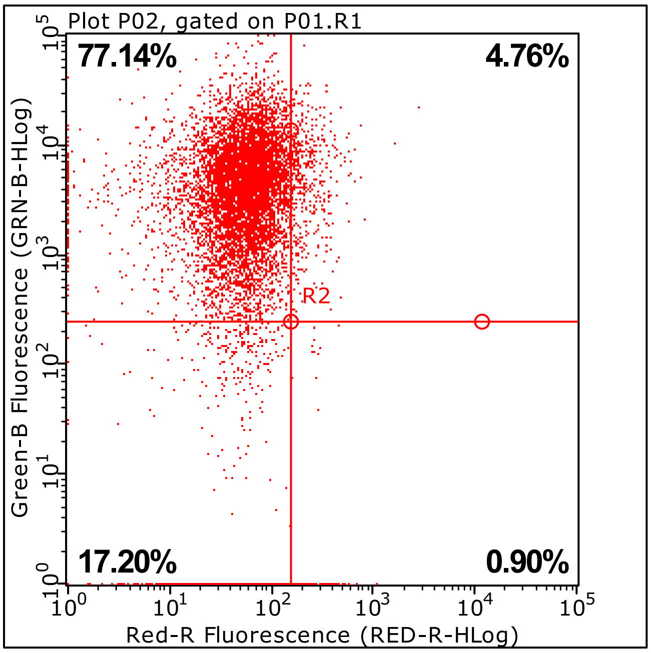

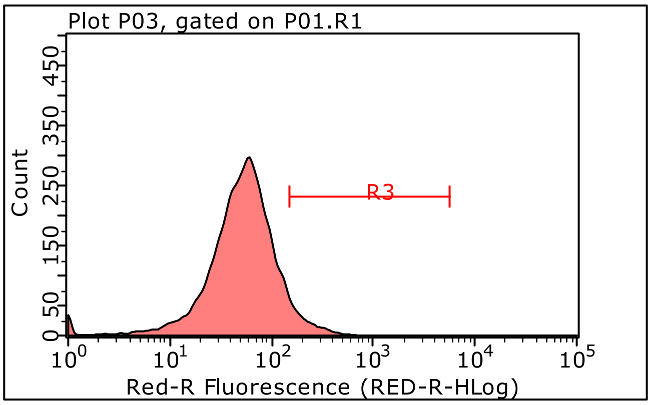

Figure 4F and S7C

DU 145
shCDKL3+ERK activator-3

Well	Sample ID	Date	R2.Percent.UL Percent for R2 gated by P01.R1 (%)	R2.Percent.UR Percent for R2 gated by P01.R1 (%)	R2.Percent.LL Percent for R2 gated by P01.R1 (%)
C04	C04	02.05.2022	77.14	4.76	17.20

Well	R2.Percent.LR Percent for R2 gated by P01.R1 (%)	R3.Percent Percent for R3 gated by P01.R1 (%)
C04	0.90	5.70

Well Number: E01 Sample ID: E01

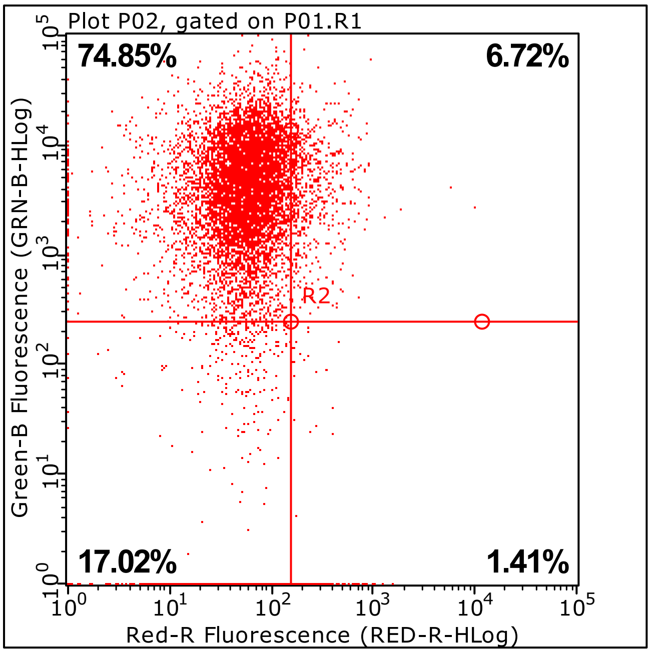

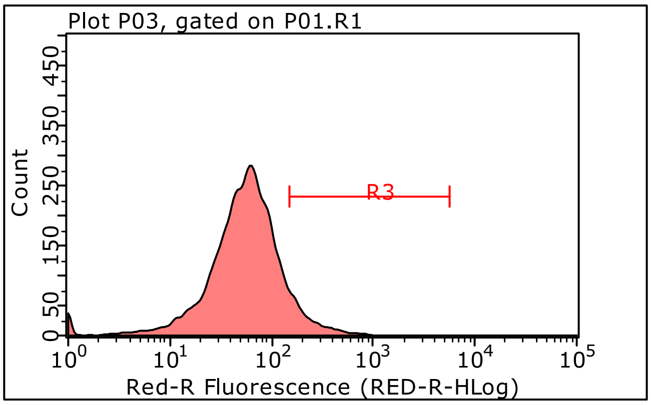

Figure 4F and S7C

DU 145
shCDKL3-1

Well	Sample ID	Date	R2.Percent.UL Percent for R2 gated by P01.R1 (%)	R2.Percent.UR Percent for R2 gated by P01.R1 (%)	R2.Percent.LL Percent for R2 gated by P01.R1 (%)
E01	E01	02.05.2022	74.85	6.72	17.02

Well	R2.Percent.LR Percent for R2 gated by P01.R1 (%)	R3.Percent Percent for R3 gated by P01.R1 (%)
E01	1.41	8.13

Well Number: E02

Sample ID: E02

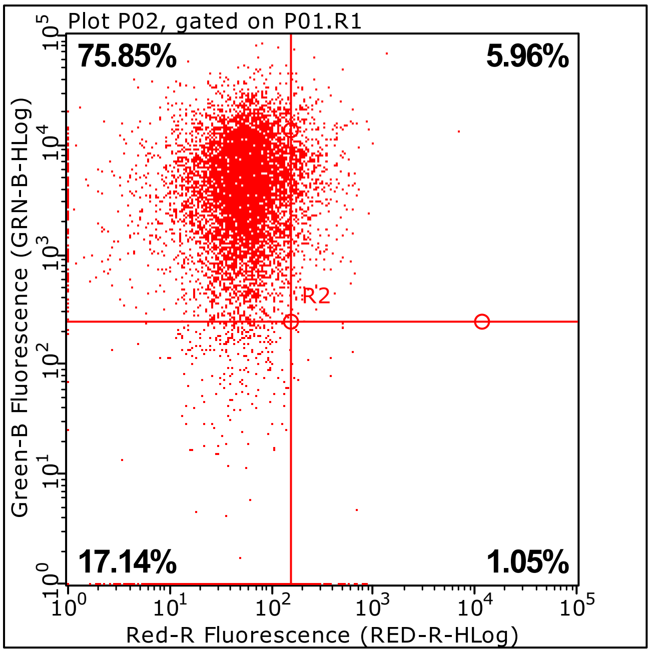

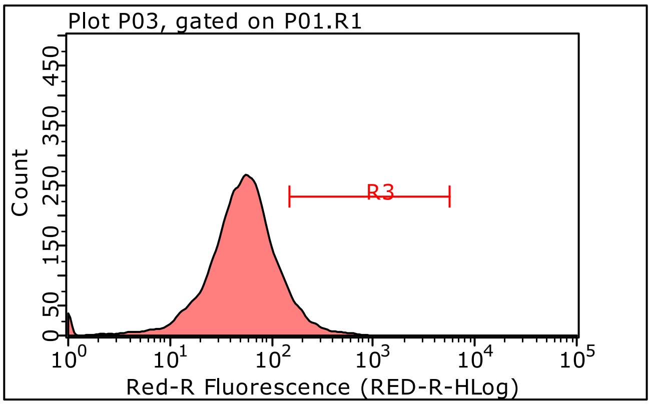

Figure 4F and S7C

DU 145
shCDKL3-2

Well	Sample ID	Date	R2.Percent.UL Percent for R2 gated by P01.R1 (%)	R2.Percent.UR Percent for R2 gated by P01.R1 (%)	R2.Percent.LL Percent for R2 gated by P01.R1 (%)
E02	E02	02.05.2022	75.85	5.96	17.14

Well	R2.Percent.LR Percent for R2 gated by P01.R1 (%)	R3.Percent Percent for R3 gated by P01.R1 (%)
E02	1.05	7.05

Well Number: E03 Sample ID: E03

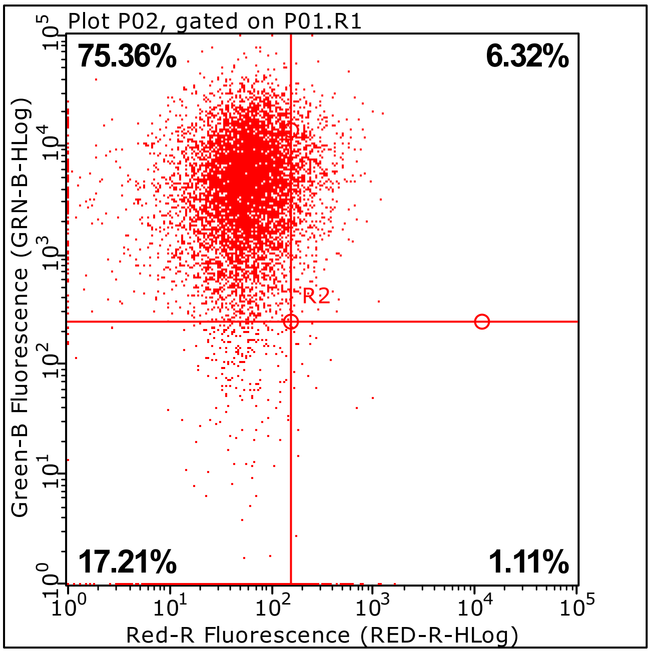

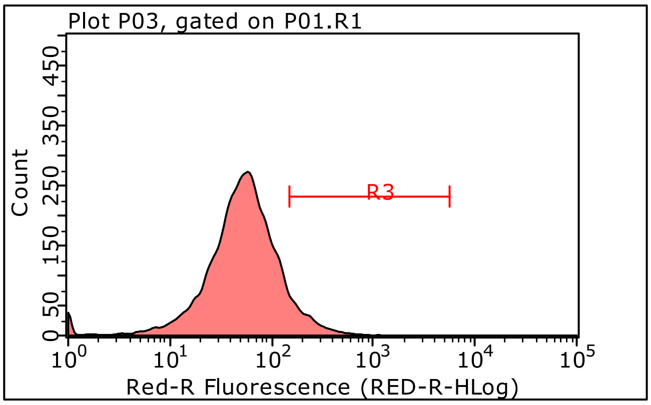

Figure 4F and S7C

DU 145
shCDKL3-3

Well	Sample ID	Date	R2.Percent.UL Percent for R2 gated by P01.R1 (%)	R2.Percent.UR Percent for R2 gated by P01.R1 (%)	R2.Percent.LL Percent for R2 gated by P01.R1 (%)
E03	E03	02.05.2022	75.36	6.32	17.21

Well	R2.Percent.LR Percent for R2 gated by P01.R1 (%)	R3.Percent Percent for R3 gated by P01.R1 (%)
E03	1.11	7.45

Well Number: A02 Sample ID: A02

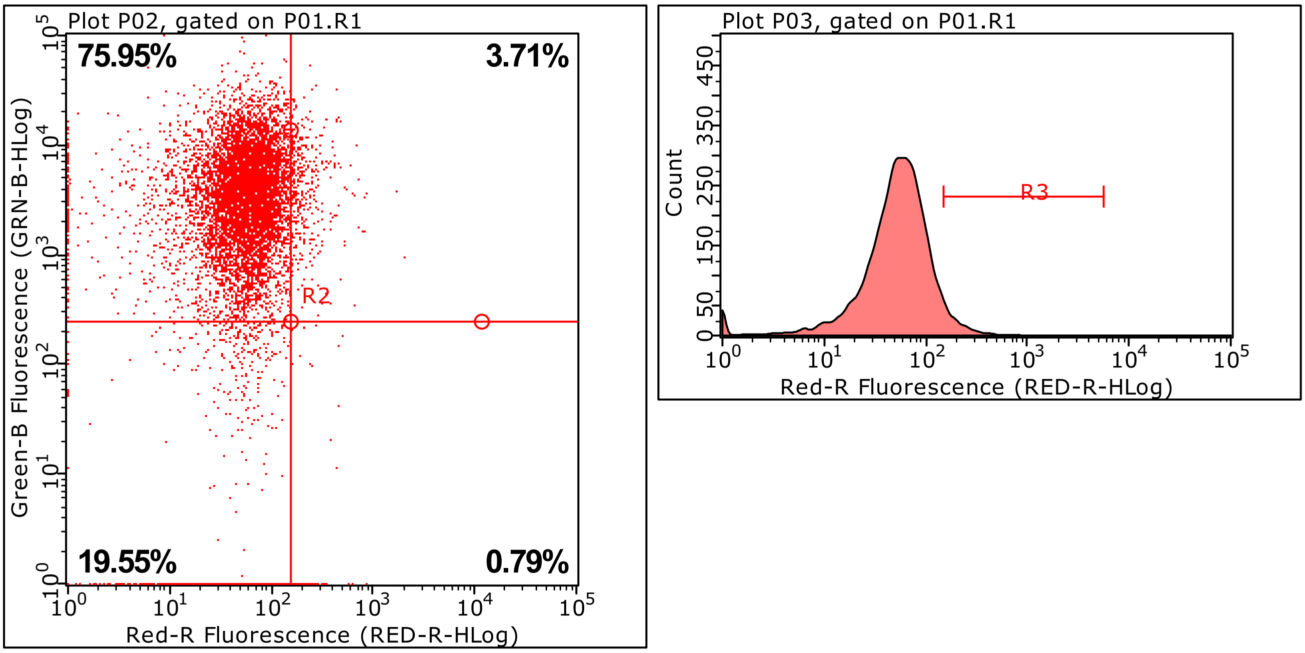

Figure 4F and S7C

DU 145
shCtrl-1

Well	Sample ID	Date	R2.Percent.UL Percent for R2 gated by P01.R1 (%)	R2.Percent.UR Percent for R2 gated by P01.R1 (%)	R2.Percent.LL Percent for R2 gated by P01.R1 (%)
A02	A02	02.05.2022	75.95	3.71	19.55

Well	R2.Percent.LR Percent for R2 gated by P01.R1 (%)	R3.Percent Percent for R3 gated by P01.R1 (%)
A02	0.79	4.54

Well Number: A03 Sample ID: A03

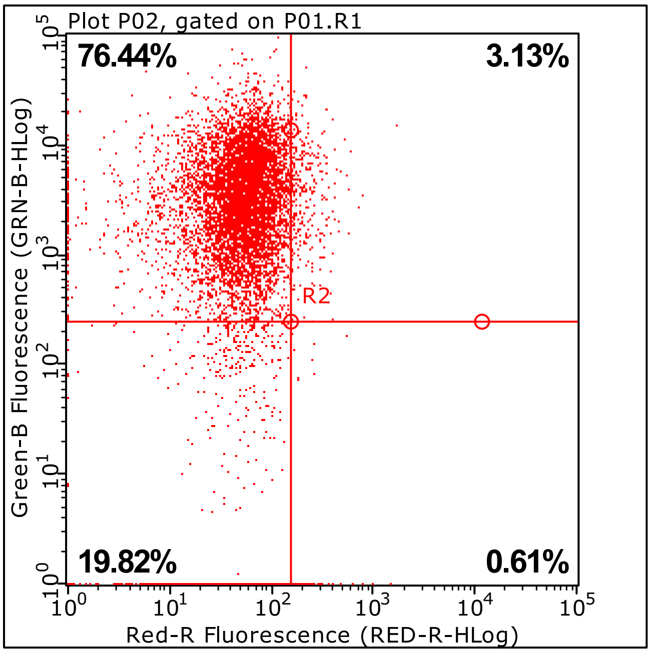

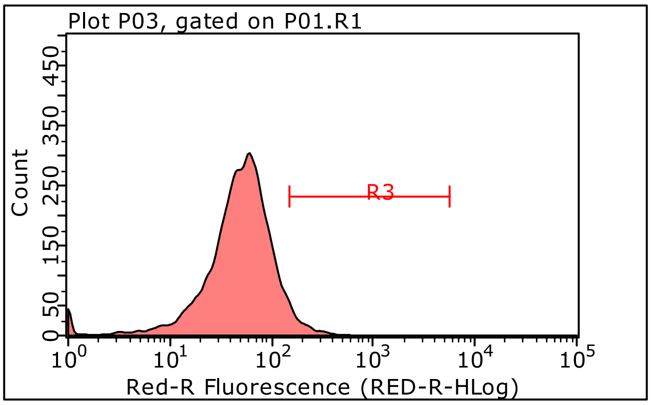

Figure 4F and S7C

DU 145
shCtrl-2

Well	Sample ID	Date	R2.Percent.UL Percent for R2 gated by P01.R1 (%)	R2.Percent.UR Percent for R2 gated by P01.R1 (%)	R2.Percent.LL Percent for R2 gated by P01.R1 (%)
A03	A03	02.05.2022	76.44	3.13	19.82

Well	R2.Percent.LR Percent for R2 gated by P01.R1 (%)	R3.Percent Percent for R3 gated by P01.R1 (%)
A03	0.61	3.75

Well Number: A04 Sample ID: A04

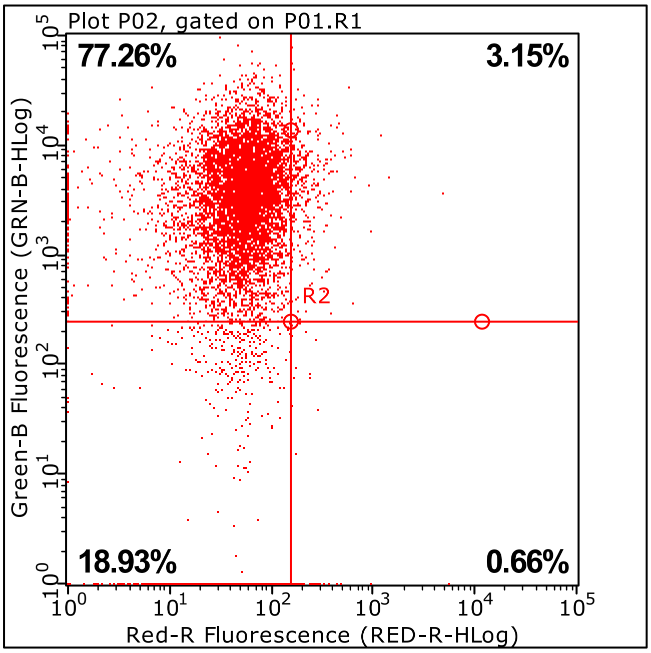

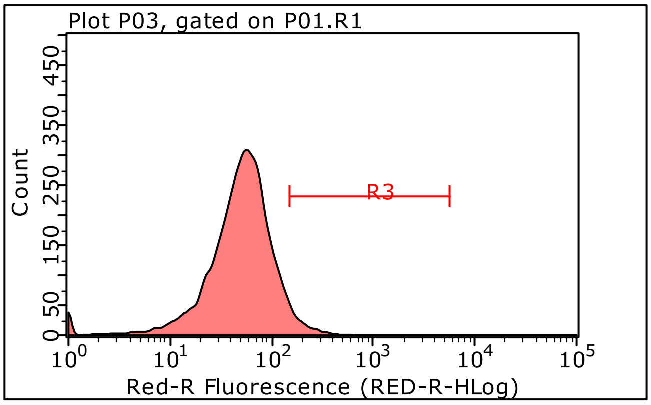

Figure 4F and S7C

DU 145
shCtrl-3

Well	Sample ID	Date	R2.Percent.UL Percent for R2 gated by P01.R1 (%)	R2.Percent.UR Percent for R2 gated by P01.R1 (%)	R2.Percent.LL Percent for R2 gated by P01.R1 (%)
A04	A04	02.05.2022	77.26	3.15	18.93

Well	R2.Percent.LR Percent for R2 gated by P01.R1 (%)	R3.Percent Percent for R3 gated by P01.R1 (%)
A04	0.66	3.81

Figure 4F and S7C
PC-3

Current Date: 07-2?-2022
Instrument Serial Number: 6735146202
Software Name: InCyte
Version Number: 3.1

Well	Sample ID	Date	R2.Percent.UL Percent for R2 gated by P01.R1 (%)	R2.Percent.UR Percent for R2 gated by P01.R1 (%)	R2.Percent.LL Percent for R2 gated by P01.R1 (%)	R2.Percent.LR Percent for R2 gated by P01.R1 (%)	R3.Percent Percent for R3 gated by P01.R1 (%)
A02	shCtrl-1	02.07.2022	83.2052903	2.528445006	13.58552951	0.680735194	3.218904989
A03	shCtrl-2	02.07.2022	82.7025975	2.471057496	14.12588773	0.700457243	3.200700457
A04	shCtrl-3	02.07.2022	83.369036	2.346640701	13.71957157	0.564751704	2.9406037
B01	shCDKL3-1	02.07.2022	75.3105006	4.939781709	17.01166729	2.738050433	7.724877682
B02	shCDKL3-2	02.07.2022	76.6399245	4.398301085	16.81925437	2.142520057	6.569136385
B03	shCDKL3-3	02.07.2022	76.6300227	4.852160728	16.641395	1.876421531	6.747536012
C01	shCDKL3+ERK激活剂-1	02.07.2022	72.6096998	3.020785219	21.35796767	3.011547344	6.050808314
C02	shCDKL3+ERK激活剂-2	02.07.2022	72.4713917	2.943890735	21.62236988	2.96234773	5.933923957
C03	shCDKL3+ERK激活剂-3	02.07.2022	71.7299189	2.864775239	22.54053058	2.864775239	5.757184967

Well Number: C01 Sample ID: C01

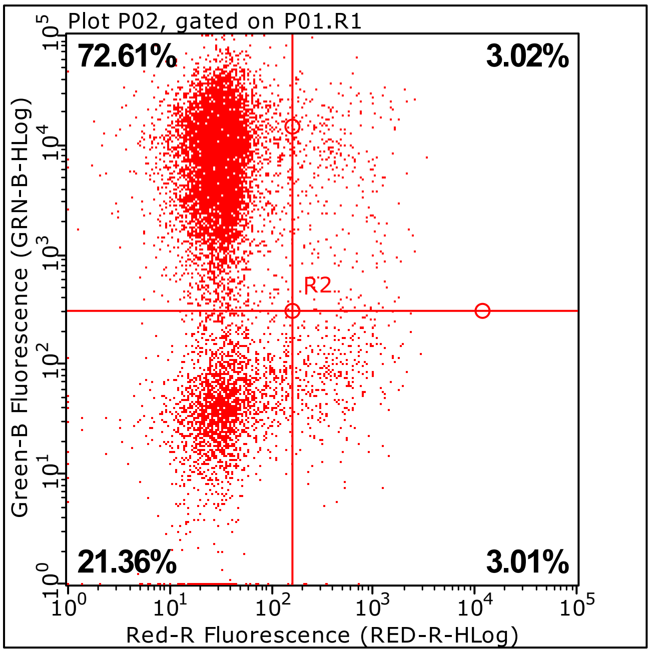

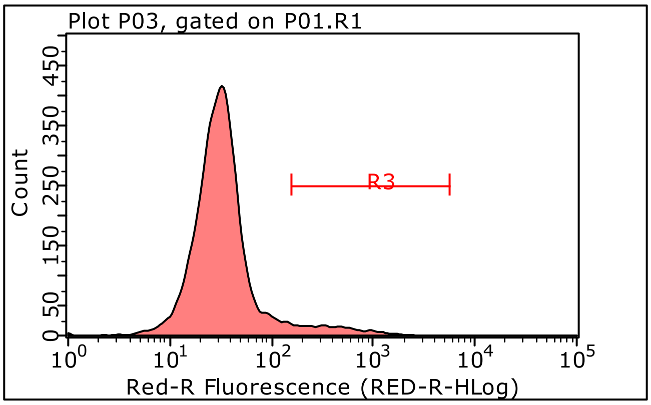

Figure 4F and S7C
PC-3
shCDKL3+ERK activator-1

Well	Sample ID	Date	R2.Percent.UL Percent for R2 gated by P01.R1 (%)	R2.Percent.UR Percent for R2 gated by P01.R1 (%)	R2.Percent.LL Percent for R2 gated by P01.R1 (%)
C01	C01	02.07.2022	72.61	3.02	21.36

Well	R2.Percent.LR Percent for R2 gated by P01.R1 (%)	R3.Percent Percent for R3 gated by P01.R1 (%)
C01	3.01	6.05

Well Number: C02 Sample ID: C02

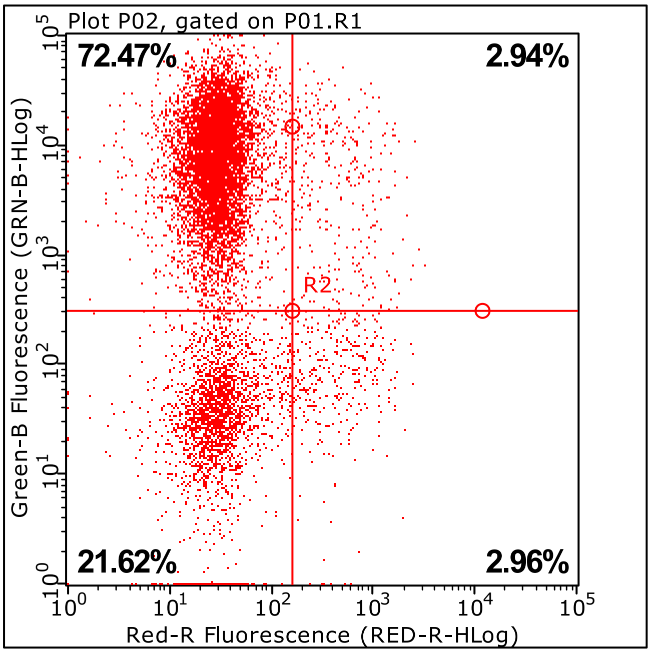

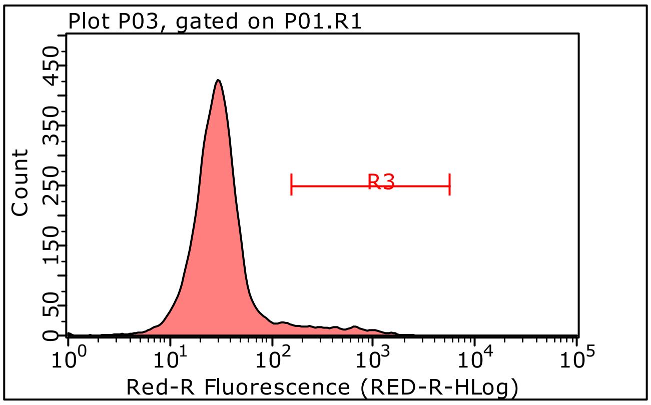

Figure 4F and S7C
PC-3
shCDKL3+ERK activator-2

Well	Sample ID	Date	R2.Percent.UL Percent for R2 gated by P01.R1 (%)	R2.Percent.UR Percent for R2 gated by P01.R1 (%)	R2.Percent.LL Percent for R2 gated by P01.R1 (%)
C02	C02	02.07.2022	72.47	2.94	21.62

Well	R2.Percent.LR Percent for R2 gated by P01.R1 (%)	R3.Percent Percent for R3 gated by P01.R1 (%)
C02	2.96	5.93

Well Number: C03 Sample ID: C03

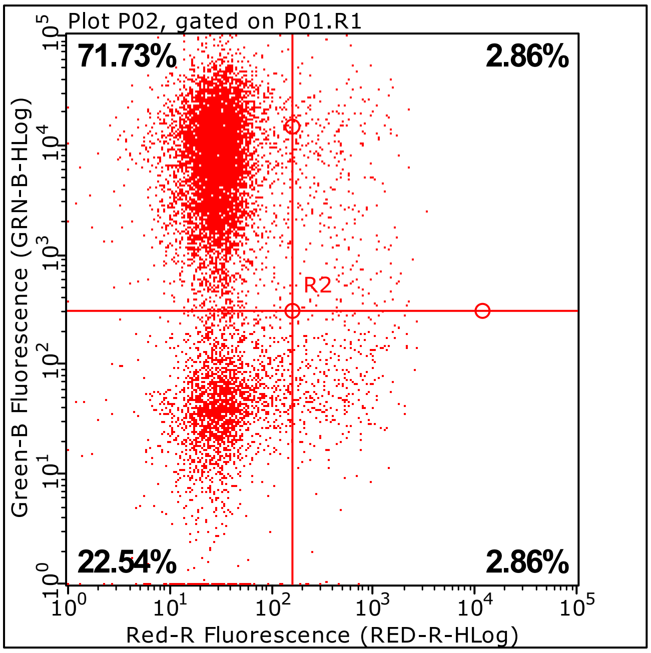

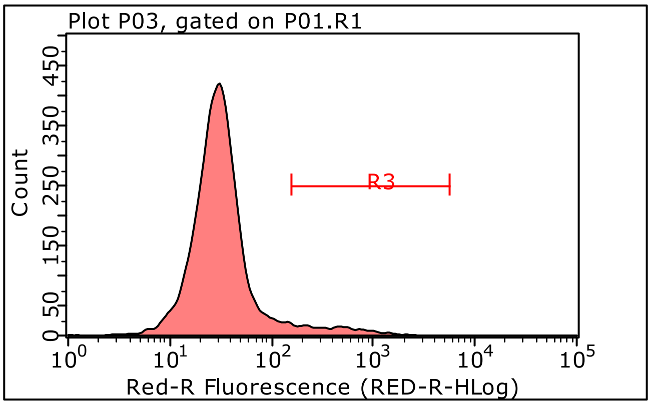

Figure 4F and S7C
PC-3
shCDKL3+ERK activator-3

Well	Sample ID	Date	R2.Percent.UL Percent for R2 gated by P01.R1 (%)	R2.Percent.UR Percent for R2 gated by P01.R1 (%)	R2.Percent.LL Percent for R2 gated by P01.R1 (%)
C03	C03	02.07.2022	71.73	2.86	22.54

Well	R2.Percent.LR Percent for R2 gated by P01.R1 (%)	R3.Percent Percent for R3 gated by P01.R1 (%)
C03	2.86	5.76

Well Number: B01 Sample ID: B01

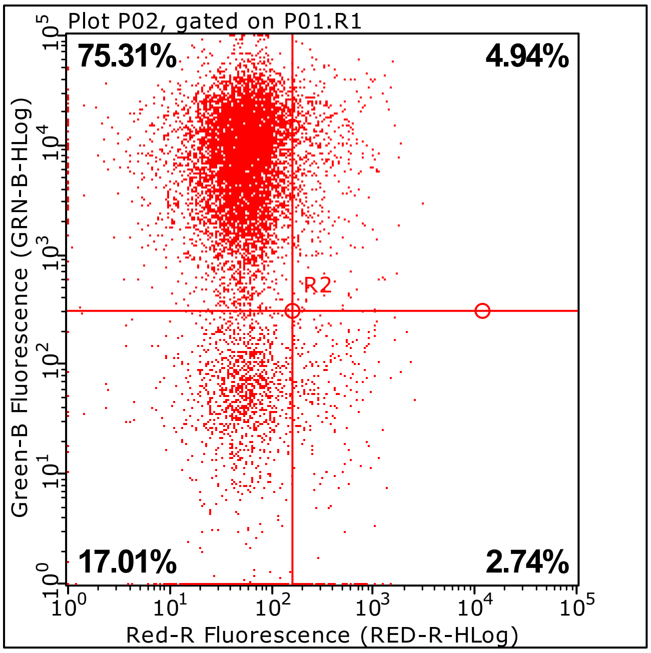

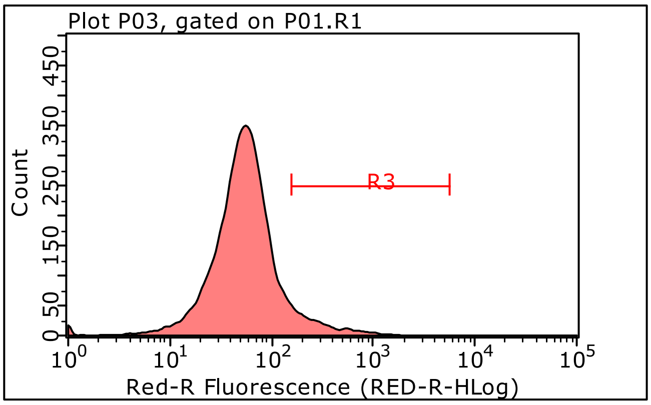

Figure 4F and S7C
PC-3
shCDKL3-1

Well	Sample ID	Date	R2.Percent.UL Percent for R2 gated by P01.R1 (%)	R2.Percent.UR Percent for R2 gated by P01.R1 (%)	R2.Percent.LL Percent for R2 gated by P01.R1 (%)
B01	B01	02.07.2022	75.31	4.94	17.01

Well	R2.Percent.LR Percent for R2 gated by P01.R1 (%)	R3.Percent Percent for R3 gated by P01.R1 (%)
B01	2.74	7.72

Well Number: B02 Sample ID: B02

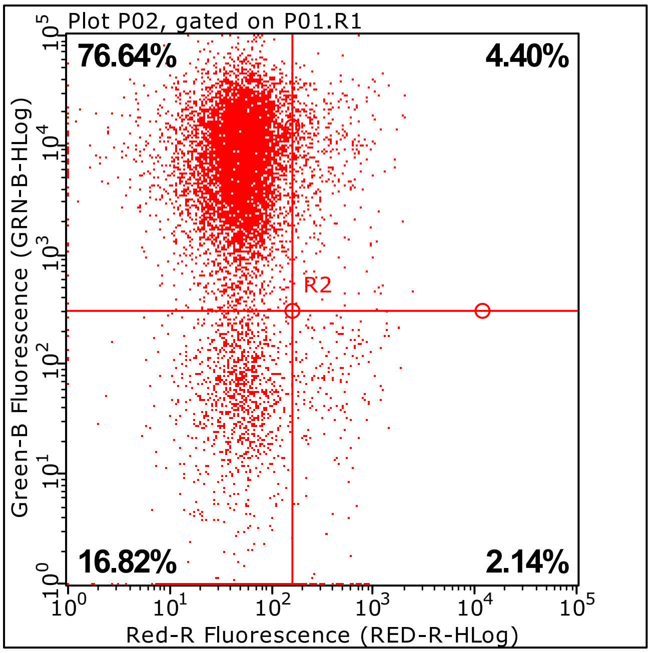

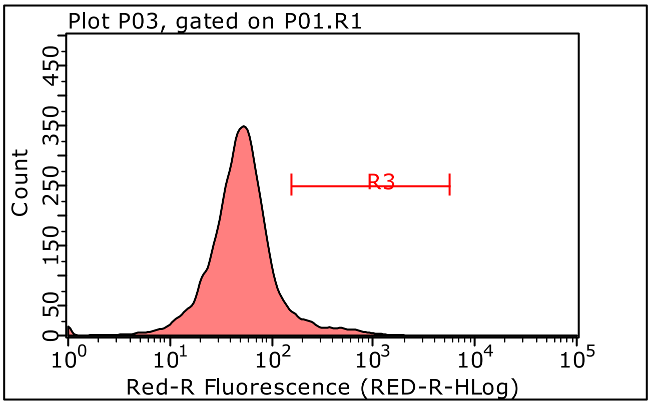

Figure 4F and S7C
PC-3
shCDKL3-2

Well	Sample ID	Date	R2.Percent.UL Percent for R2 gated by P01.R1 (%)	R2.Percent.UR Percent for R2 gated by P01.R1 (%)	R2.Percent.LL Percent for R2 gated by P01.R1 (%)
B02	B02	02.07.2022	76.64	4.40	16.82

Well	R2.Percent.LR Percent for R2 gated by P01.R1 (%)	R3.Percent Percent for R3 gated by P01.R1 (%)
B02	2.14	6.57

Well Number: B03 Sample ID: B03

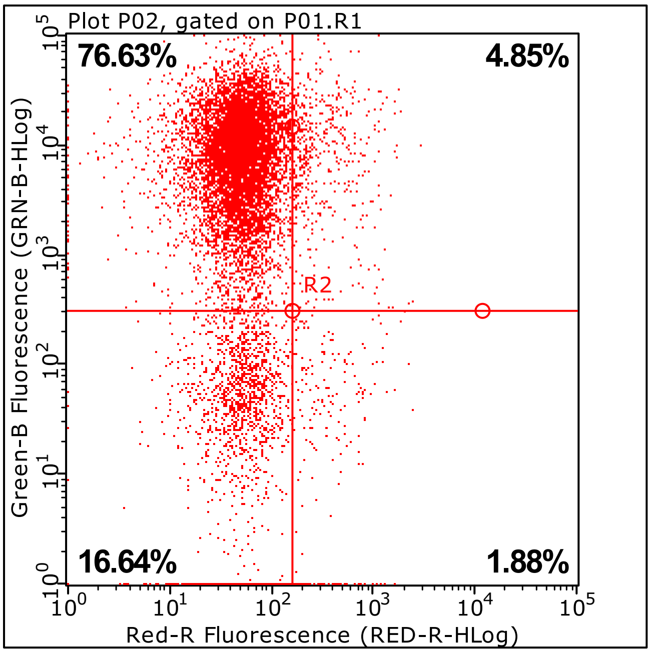

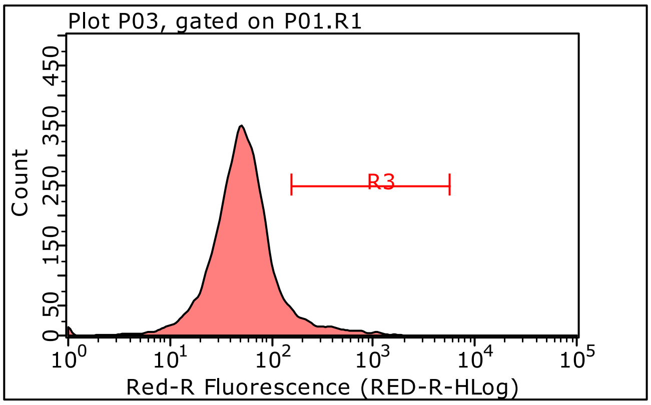

Figure 4F and S7C
PC-3
shCDKL3-3

Well	Sample ID	Date	R2.Percent.UL Percent for R2 gated by P01.R1 (%)	R2.Percent.UR Percent for R2 gated by P01.R1 (%)	R2.Percent.LL Percent for R2 gated by P01.R1 (%)
B03	B03	02.07.2022	76.63	4.85	16.64

Well	R2.Percent.LR Percent for R2 gated by P01.R1 (%)	R3.Percent Percent for R3 gated by P01.R1 (%)
B03	1.88	6.75

Well Number: A02 Sample ID: A02

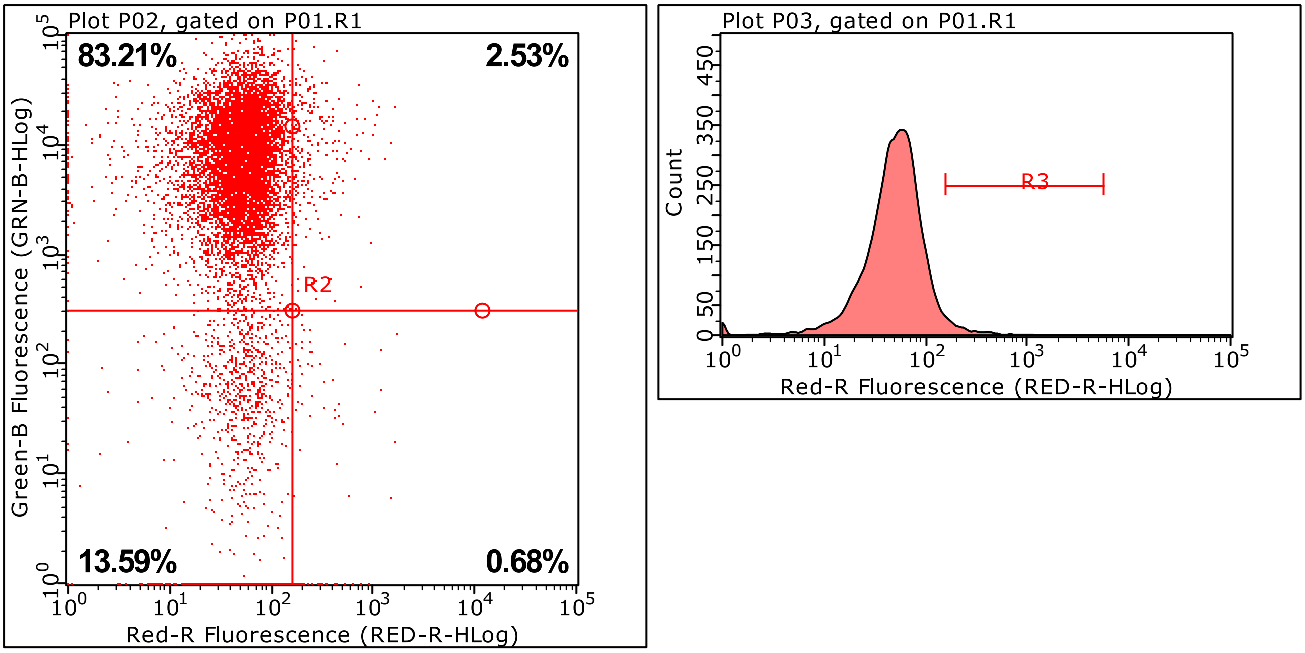

Figure 4F and S7C
PC-3
shCtrl-1

Well	Sample ID	Date	R2.Percent.UL Percent for R2 gated by P01.R1 (%)	R2.Percent.UR Percent for R2 gated by P01.R1 (%)	R2.Percent.LL Percent for R2 gated by P01.R1 (%)
A02	A02	02.07.2022	83.21	2.53	13.59

Well	R2.Percent.LR Percent for R2 gated by P01.R1 (%)	R3.Percent Percent for R3 gated by P01.R1 (%)
A02	0.68	3.22

Well Number: A03 Sample ID: A03

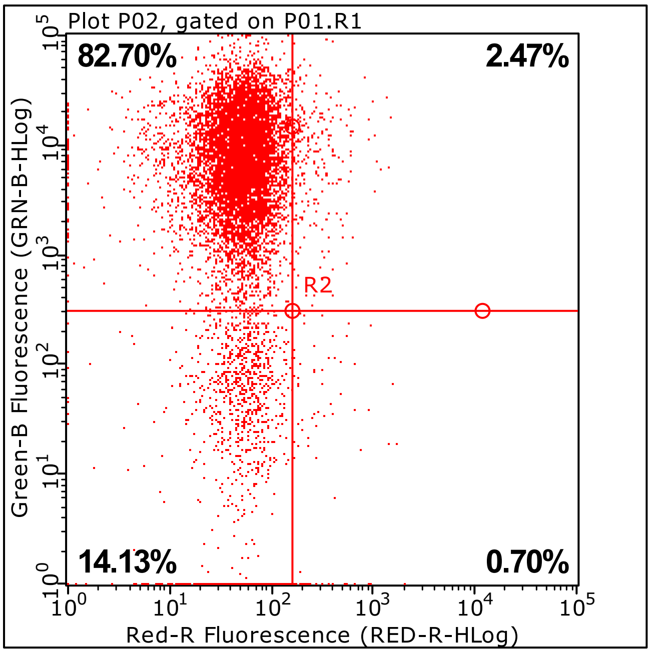

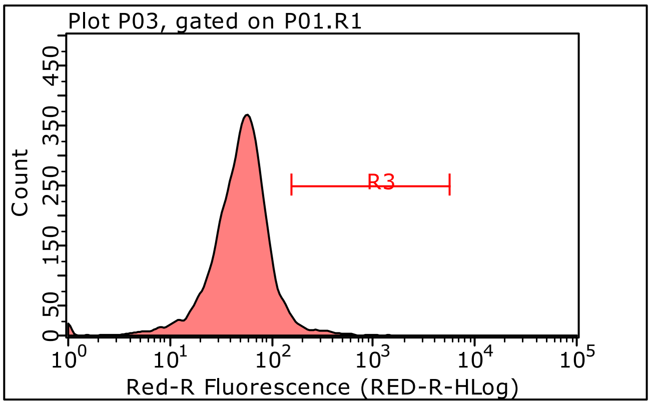

Figure 4F and S7C
PC-3
shCtrl-2

Well	Sample ID	Date	R2.Percent.UL Percent for R2 gated by P01.R1 (%)	R2.Percent.UR Percent for R2 gated by P01.R1 (%)	R2.Percent.LL Percent for R2 gated by P01.R1 (%)
A03	A03	02.07.2022	82.70	2.47	14.13

Well	R2.Percent.LR Percent for R2 gated by P01.R1 (%)	R3.Percent Percent for R3 gated by P01.R1 (%)
A03	0.70	3.20

Well Number: A04 Sample ID: A04

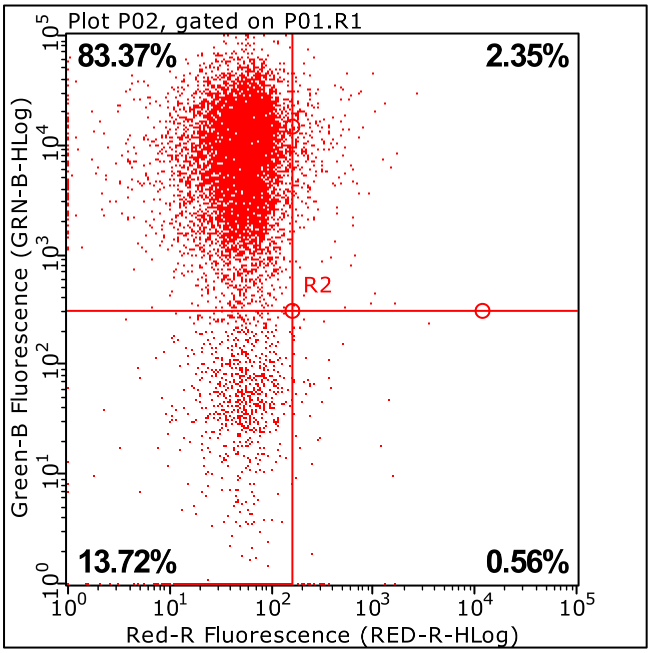

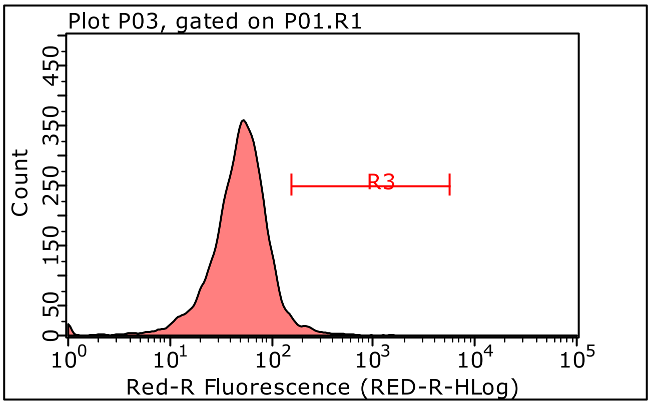

Figure 4F and S7C
PC-3
shCtrl-3

Well	Sample ID	Date	R2.Percent.UL Percent for R2 gated by P01.R1 (%)	R2.Percent.UR Percent for R2 gated by P01.R1 (%)	R2.Percent.LL Percent for R2 gated by P01.R1 (%)
A04	A04	02.07.2022	83.37	2.35	13.72

Well	R2.Percent.LR Percent for R2 gated by P01.R1 (%)	R3.Percent Percent for R3 gated by P01.R1 (%)
A04	0.56	2.94

Well Number: B01 Sample ID: B01
File Name: E:/a-ZY/20200927 DU145 HF DW/2020-09-27_at_04-38-26pm.fcs

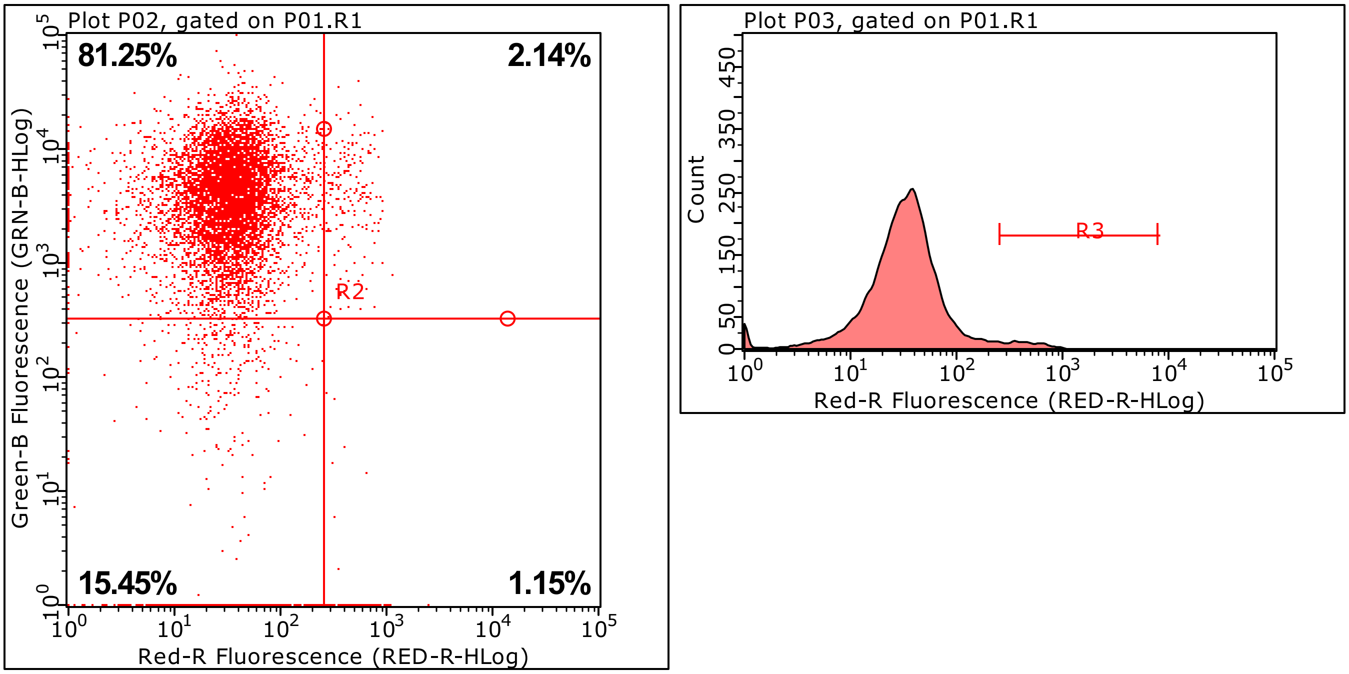

Fugure 6C
NC(shSTAT1+CDKL3)-1

Well	Sample ID	Date	R1.Percent Percent for R1 (%)	R2.Percent.UL Percent for R2 gated by P01.R1 (%)	R2.Percent.UR Percent for R2 gated by P01.R1 (%)	R2.Percent.LL Percent for R2 gated by P01.R1 (%)
B01	B01	09.28.2020	55.83	81.25	2.14	15.45

Well	R2.Percent.LR Percent for R2 gated by P01.R1 (%)	R3.Percent Percent for R3 gated by P01.R1 (%)
B01	1.15	3.30

Well Number: B03 Sample ID: B03
File Name: E:/a-ZY/20200927 DU145 HF DW/2020-09-27_at_04-38-26pm.fcs

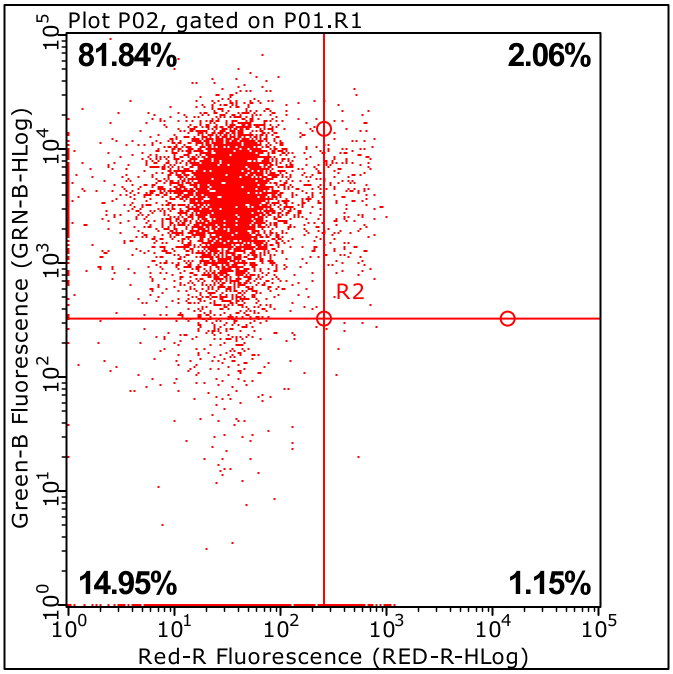

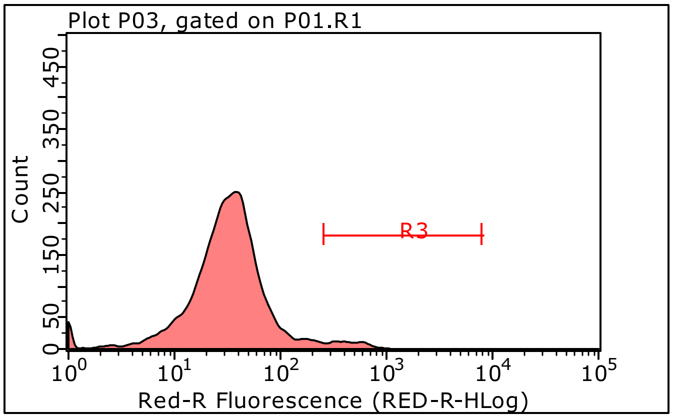

Fugure 6C
NC(shSTAT1+CDKL3)-2

Well	Sample ID	Date	R1.Percent Percent for R1 (%)	R2.Percent.UL Percent for R2 gated by P01.R1 (%)	R2.Percent.UR Percent for R2 gated by P01.R1 (%)	R2.Percent.LL Percent for R2 gated by P01.R1 (%)
B03	B03	09.28.2020	55.78	81.84	2.06	14.95

Well	R2.Percent.LR Percent for R2 gated by P01.R1 (%)	R3.Percent Percent for R3 gated by P01.R1 (%)
B03	1.15	3.22

Well Number: B04 Sample ID: B04
File Name: E:/a-ZY/20200927 DU145 HF DW/2020-09-27_at_04-38-26pm.fcs

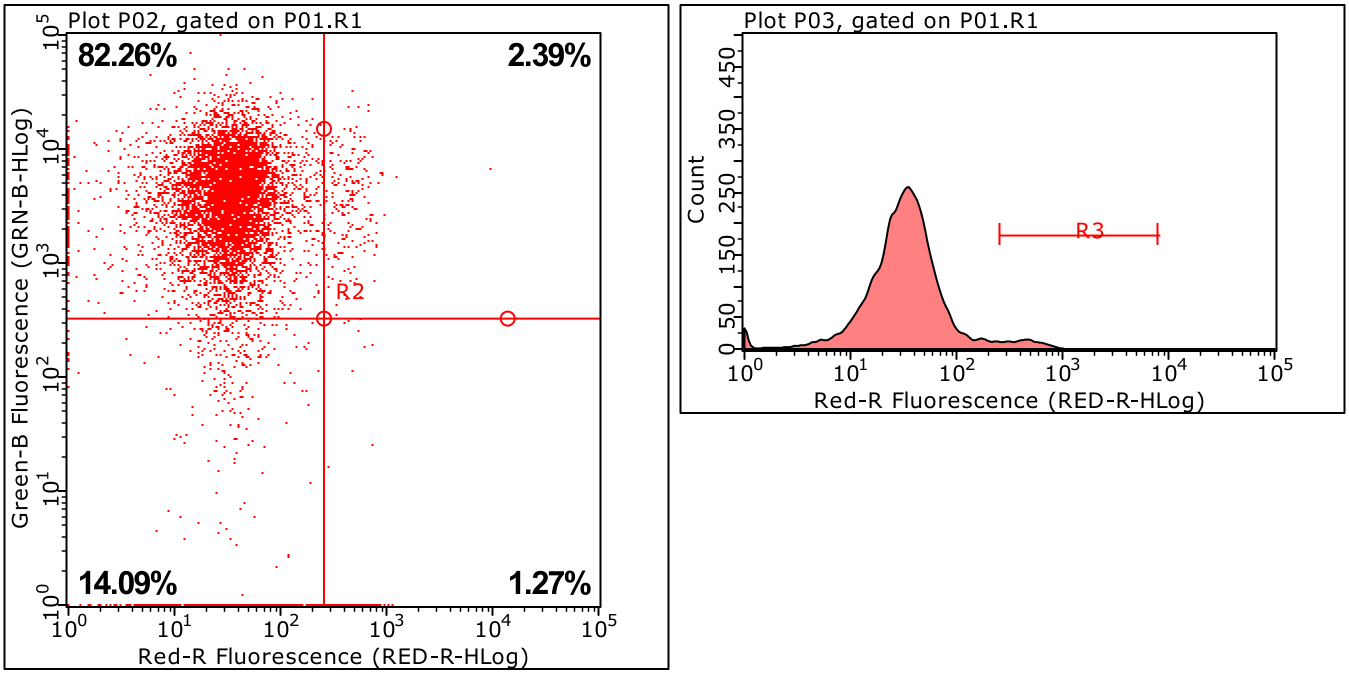

Fugure 6C
NC(shSTAT1+CDKL3)-3

Well	Sample ID	Date	R1.Percent Percent for R1 (%)	R2.Percent.UL Percent for R2 gated by P01.R1 (%)	R2.Percent.UR Percent for R2 gated by P01.R1 (%)	R2.Percent.LL Percent for R2 gated by P01.R1 (%)
B04	B04	09.28.2020	59.07	82.26	2.39	14.09

Well	R2.Percent.LR Percent for R2 gated by P01.R1 (%)	R3.Percent Percent for R3 gated by P01.R1 (%)
B04	1.27	3.65

Well Number: C02 Sample ID: C02
File Name: E:/a-ZY/20200927 DU145 HF DW/2020-09-27_at_04-38-26pm.fcs

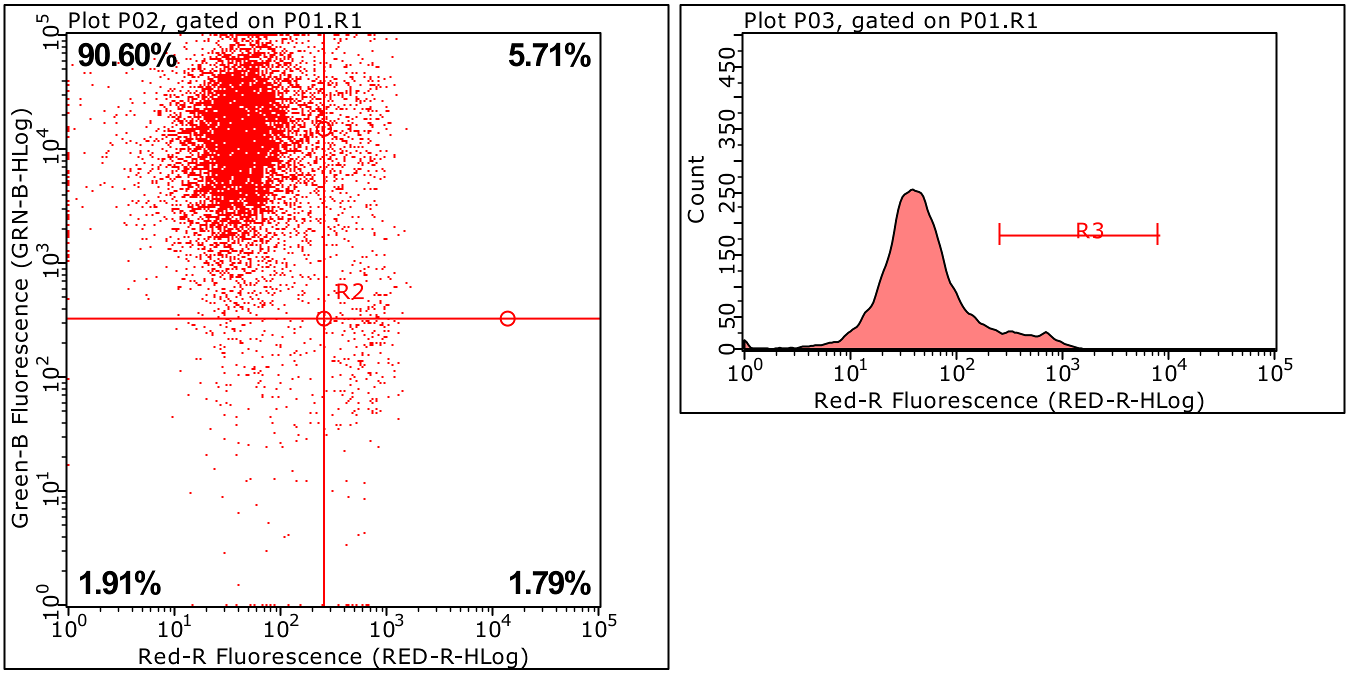

Figure 6C
shSTAT1+CDKL3-1

Well	Sample ID	Date	R1.Percent Percent for R1 (%)	R2.Percent.UL Percent for R2 gated by P01.R1 (%)	R2.Percent.UR Percent for R2 gated by P01.R1 (%)	R2.Percent.LL Percent for R2 gated by P01.R1 (%)
C02	C02	09.28.2020	59.21	90.60	5.71	1.91

Well	R2.Percent.LR Percent for R2 gated by P01.R1 (%)	R3.Percent Percent for R3 gated by P01.R1 (%)
C02	1.79	7.52

Well Number: C03 Sample ID: C03
File Name: E:/a-ZY/20200927 DU145 HF DW/2020-09-27_at_04-38-26pm.fcs

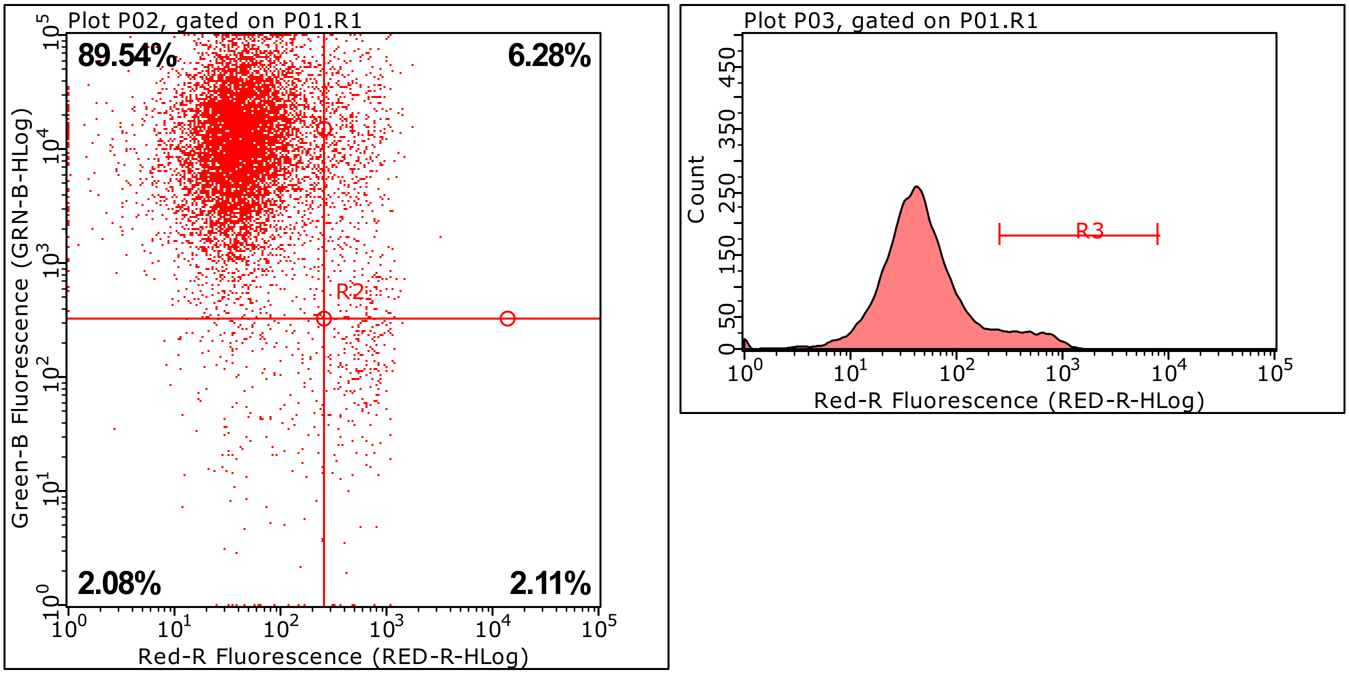

Figure 6C
shSTAT1+CDKL3-2

Well	Sample ID	Date	R1.Percent Percent for R1 (%)	R2.Percent.UL Percent for R2 gated by P01.R1 (%)	R2.Percent.UR Percent for R2 gated by P01.R1 (%)	R2.Percent.LL Percent for R2 gated by P01.R1 (%)
C03	C03	09.28.2020	58.82	89.54	6.28	2.08

Well	R2.Percent.LR Percent for R2 gated by P01.R1 (%)	R3.Percent Percent for R3 gated by P01.R1 (%)
C03	2.11	8.38

Well Number: C04 Sample ID: C04
File Name: E:/a-ZY/20200927 DU145 HF DW/2020-09-27_at_04-38-26pm.fcs

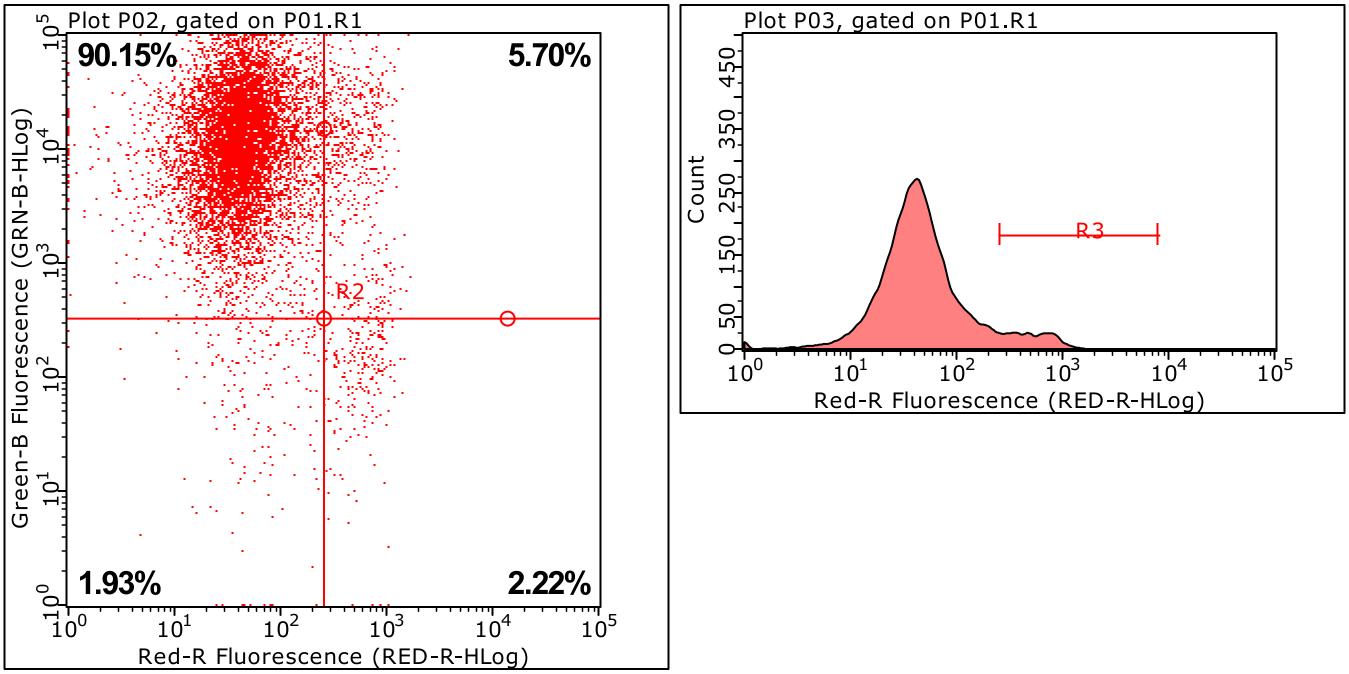

Figure 6C
shSTAT1+CDKL3-3

Well	Sample ID	Date	R1.Percent Percent for R1 (%)	R2.Percent.UL Percent for R2 gated by P01.R1 (%)	R2.Percent.UR Percent for R2 gated by P01.R1 (%)	R2.Percent.LL Percent for R2 gated by P01.R1 (%)
C04	C04	09.28.2020	57.03	90.15	5.70	1.93

Well	R2.Percent.LR Percent for R2 gated by P01.R1 (%)	R3.Percent Percent for R3 gated by P01.R1 (%)
C04	2.22	7.95

Well Number: D02 Sample ID: D02
File Name: E:/a-ZY/20200927 DU145 HF DW/2020-09-27_at_04-38-26pm.fcs

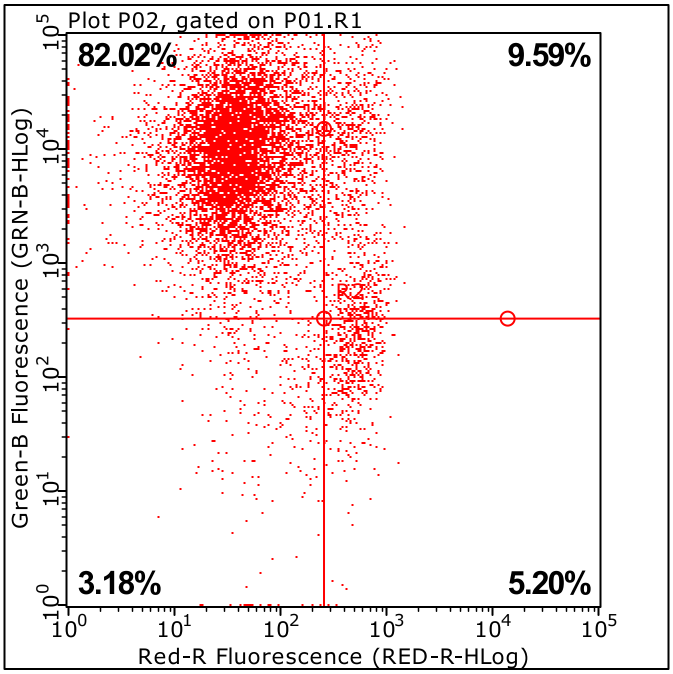

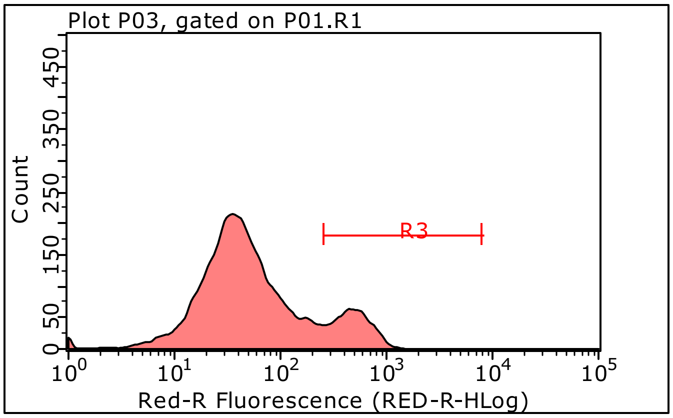

Figure 6C
shSTAT1+NC-CDKL3-1

Well	Sample ID	Date	R1.Percent Percent for R1 (%)	R2.Percent.UL Percent for R2 gated by P01.R1 (%)	R2.Percent.UR Percent for R2 gated by P01.R1 (%)	R2.Percent.LL Percent for R2 gated by P01.R1 (%)
D02	D02	09.28.2020	59.67	82.02	9.59	3.18

Well	R2.Percent.LR Percent for R2 gated by P01.R1 (%)	R3.Percent Percent for R3 gated by P01.R1 (%)
D02	5.20	14.79

Well Number: D03 Sample ID: D03
File Name: E:/a-ZY/20200927 DU145 HF DW/2020-09-27_at_04-38-26pm.fcs

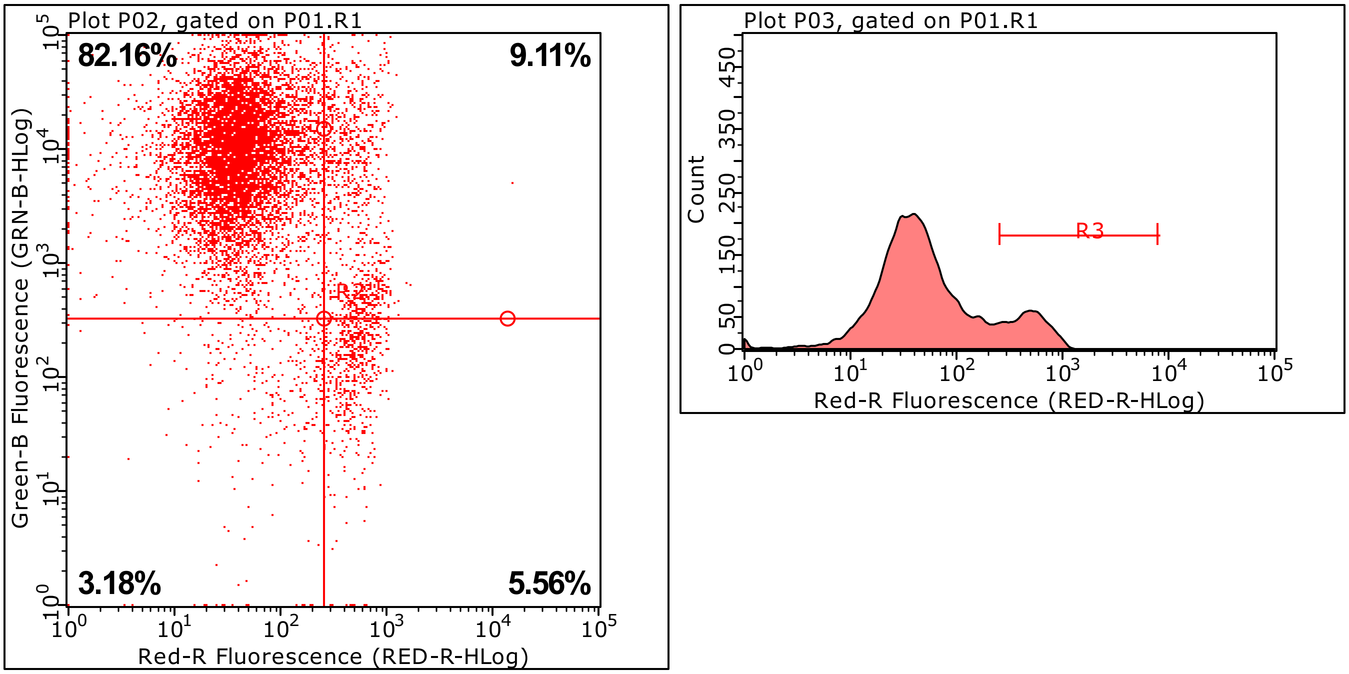

Figure 6C
shSTAT1+NC-CDKL3-2

Well	Sample ID	Date	R1.Percent Percent for R1 (%)	R2.Percent.UL Percent for R2 gated by P01.R1 (%)	R2.Percent.UR Percent for R2 gated by P01.R1 (%)	R2.Percent.LL Percent for R2 gated by P01.R1 (%)
D03	D03	09.28.2020	60.10	82.16	9.11	3.18

Well	R2.Percent.LR Percent for R2 gated by P01.R1 (%)	R3.Percent Percent for R3 gated by P01.R1 (%)
D03	5.56	14.69

Well Number: D04 Sample ID: D04
File Name: E:/a-ZY/20200927 DU145 HF DW/2020-09-27_at_04-38-26pm.fcs

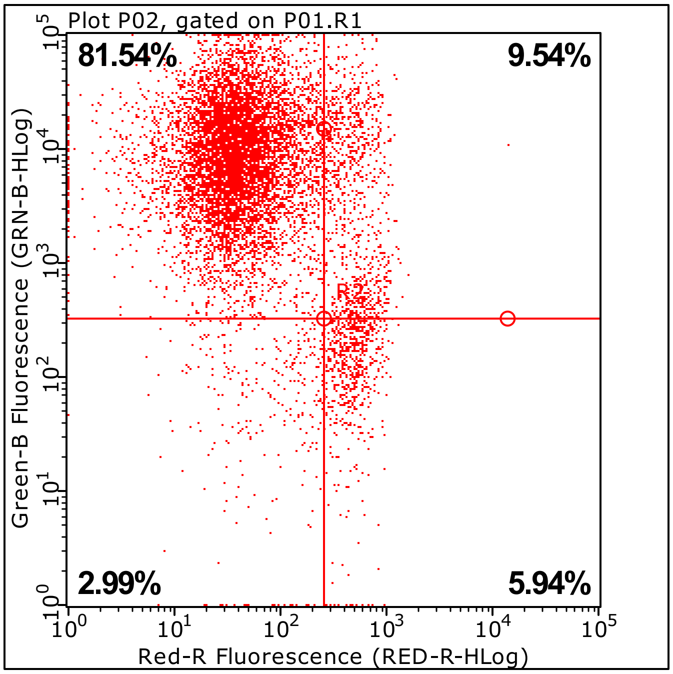

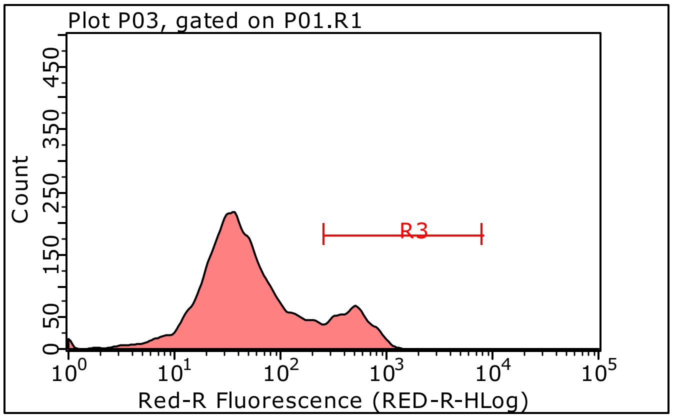

Figure 6C
shSTAT1+NC-CDKL3-3

Well	Sample ID	Date	R1.Percent Percent for R1 (%)	R2.Percent.UL Percent for R2 gated by P01.R1 (%)	R2.Percent.UR Percent for R2 gated by P01.R1 (%)	R2.Percent.LL Percent for R2 gated by P01.R1 (%)
D04	D04	09.28.2020	58.97	81.54	9.54	2.99

Well	R2.Percent.LR Percent for R2 gated by P01.R1 (%)	R3.Percent Percent for R3 gated by P01.R1 (%)
D04	5.94	15.47

Well Number: A02 Sample ID: A02
File Name: E:/a-ZY/20200927 DU145 HF DW/2020-09-27_at_04-38-26pm.fcs

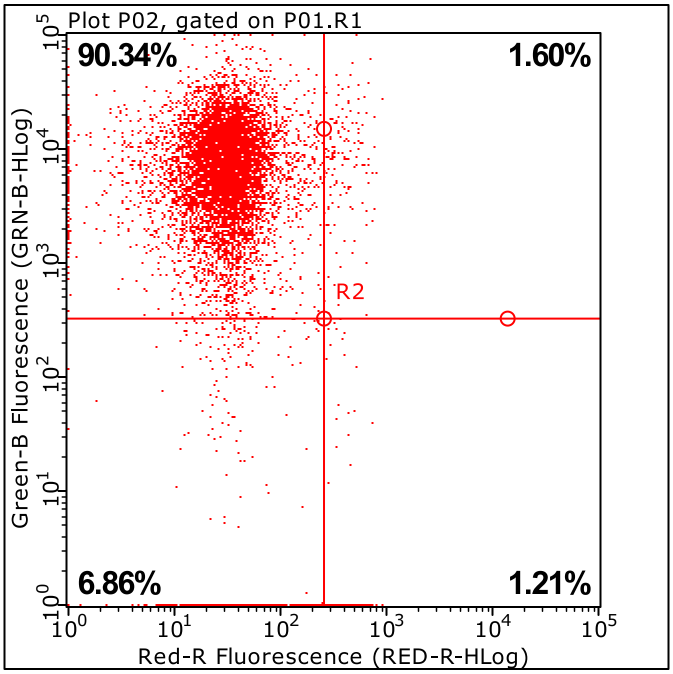

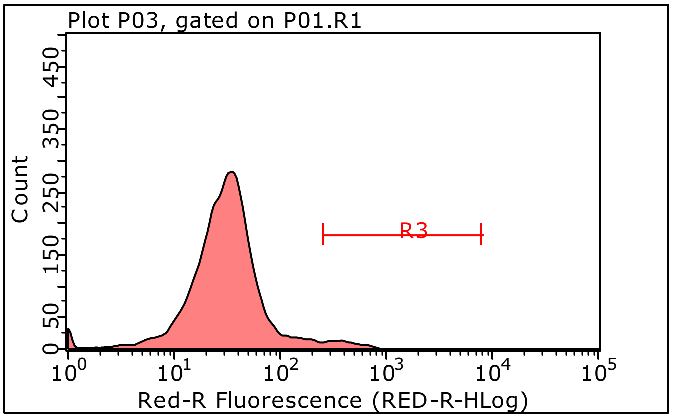

Figure 6C
CDKL3+NC-shSTAT1-1

Well	Sample ID	Date	R1.Percent Percent for R1 (%)	R2.Percent.UL Percent for R2 gated by P01.R1 (%)	R2.Percent.UR Percent for R2 gated by P01.R1 (%)	R2.Percent.LL Percent for R2 gated by P01.R1 (%)
A02	A02	09.28.2020	64.43	90.34	1.60	6.86

Well	R2.Percent.LR Percent for R2 gated by P01.R1 (%)	R3.Percent Percent for R3 gated by P01.R1 (%)
A02	1.21	2.83

Well Number: A03 Sample ID: A03
File Name: E:/a-ZY/20200927 DU145 HF DW/2020-09-27_at_04-38-26pm.fcs

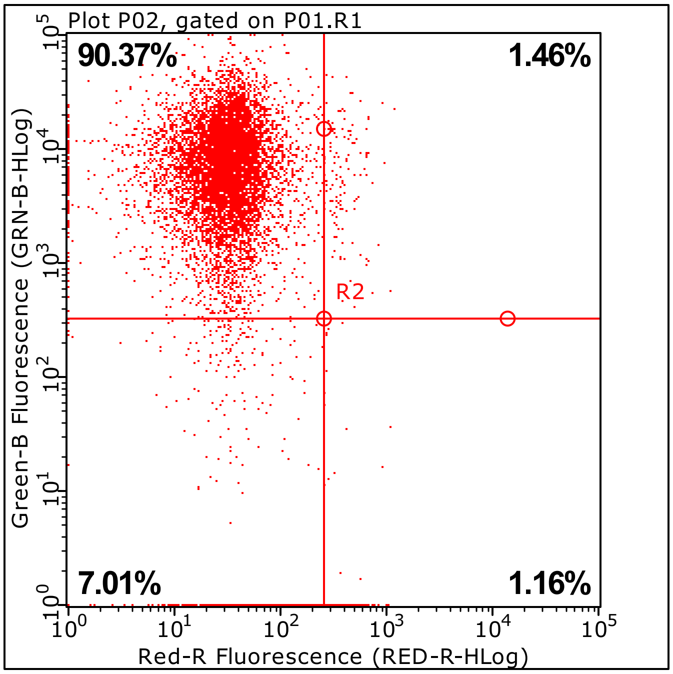

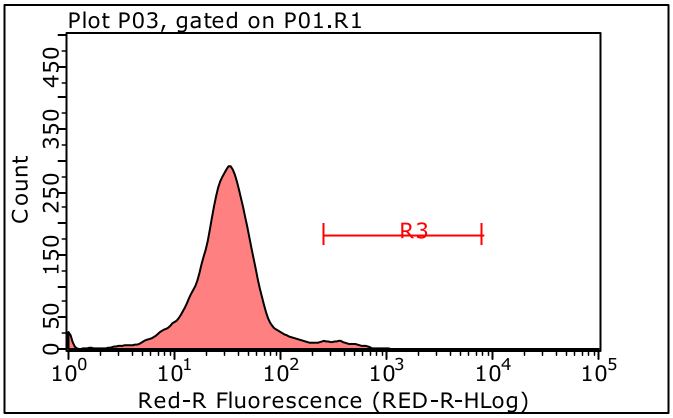

Figure 6C
CDKL3+NC-shSTAT1-2

Well	Sample ID	Date	R1.Percent Percent for R1 (%)	R2.Percent.UL Percent for R2 gated by P01.R1 (%)	R2.Percent.UR Percent for R2 gated by P01.R1 (%)	R2.Percent.LL Percent for R2 gated by P01.R1 (%)
A03	A03	09.28.2020	63.69	90.37	1.46	7.01

Well	R2.Percent.LR Percent for R2 gated by P01.R1 (%)	R3.Percent Percent for R3 gated by P01.R1 (%)
A03	1.16	2.62

Well Number: A04 Sample ID: A04
File Name: E:/a-ZY/20200927 DU145 HF DW/2020-09-27_at_04-38-26pm.fcs

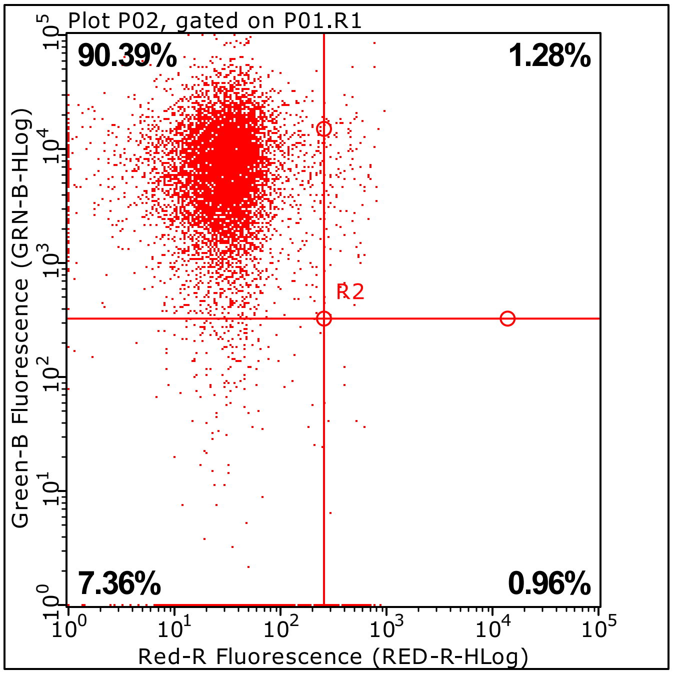

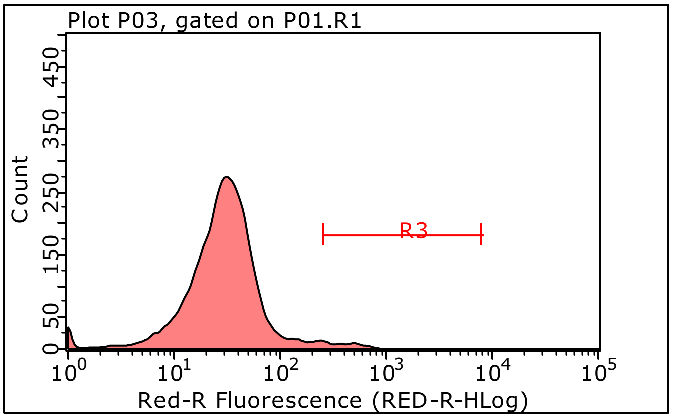

Figure 6C
CDKL3+NC-shSTAT1-3

Well	Sample ID	Date	R1.Percent Percent for R1 (%)	R2.Percent.UL Percent for R2 gated by P01.R1 (%)	R2.Percent.UR Percent for R2 gated by P01.R1 (%)	R2.Percent.LL Percent for R2 gated by P01.R1 (%)
A04	A04	09.28.2020	62.58	90.39	1.28	7.36

Well	R2.Percent.LR Percent for R2 gated by P01.R1 (%)	R3.Percent Percent for R3 gated by P01.R1 (%)
A04	0.96	2.24
