# Supplementary material for: Inhibition of CDKL3 downregulates STAT1 thus suppressing prostate cancer development
Source: Cell Death Dis. 2023 Mar 10;14(3):189. doi: 10.1038/s41419-023-05694-3 (PMC10006411; doi:10.1038/s41419-023-05694-3)
Supplement: Supplementary file 22 — Original Data File [file 41419_2023_5694_MOESM22_ESM.pptx]

## Slide 1
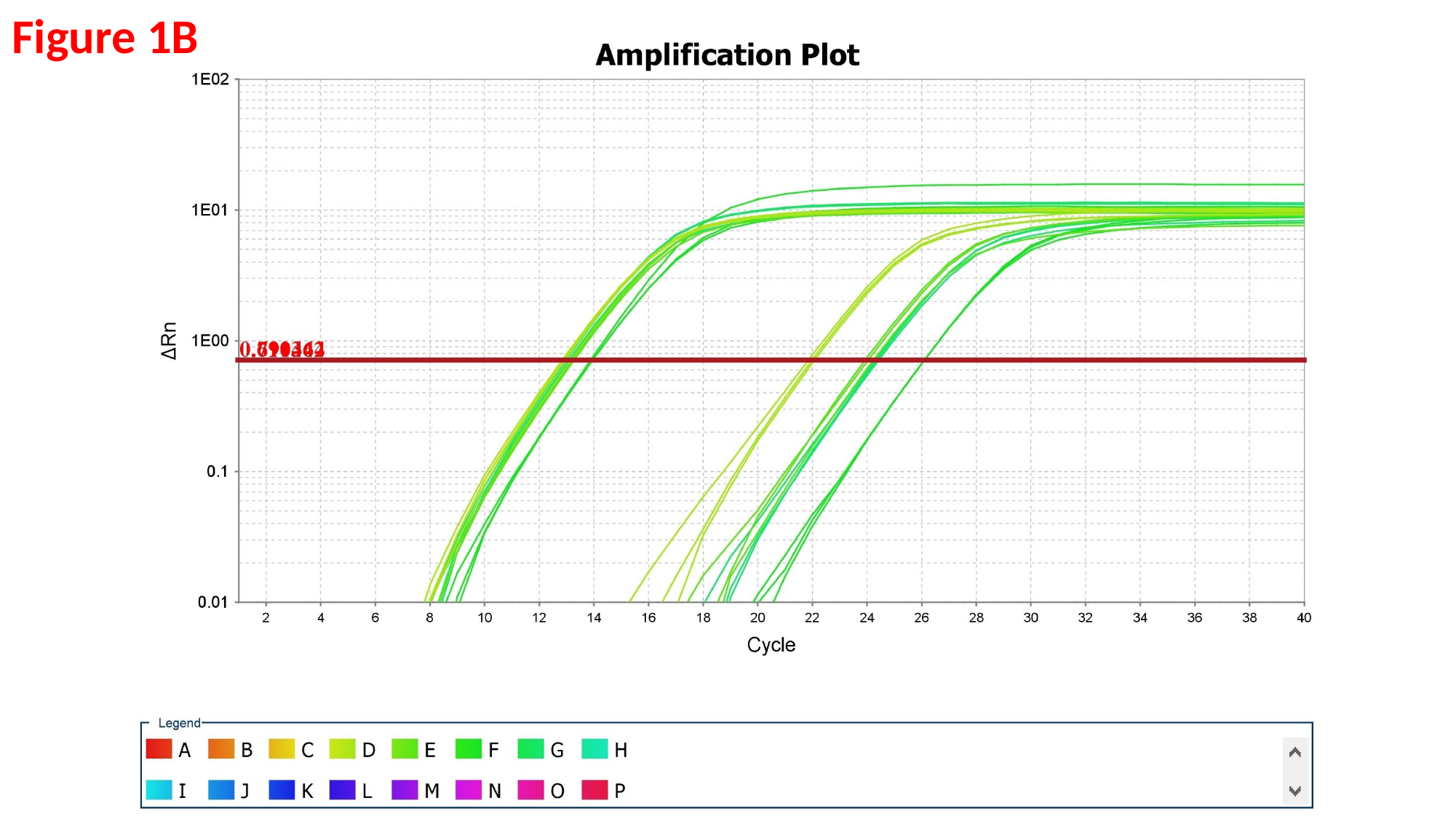

Figure 1B

## Slide 2
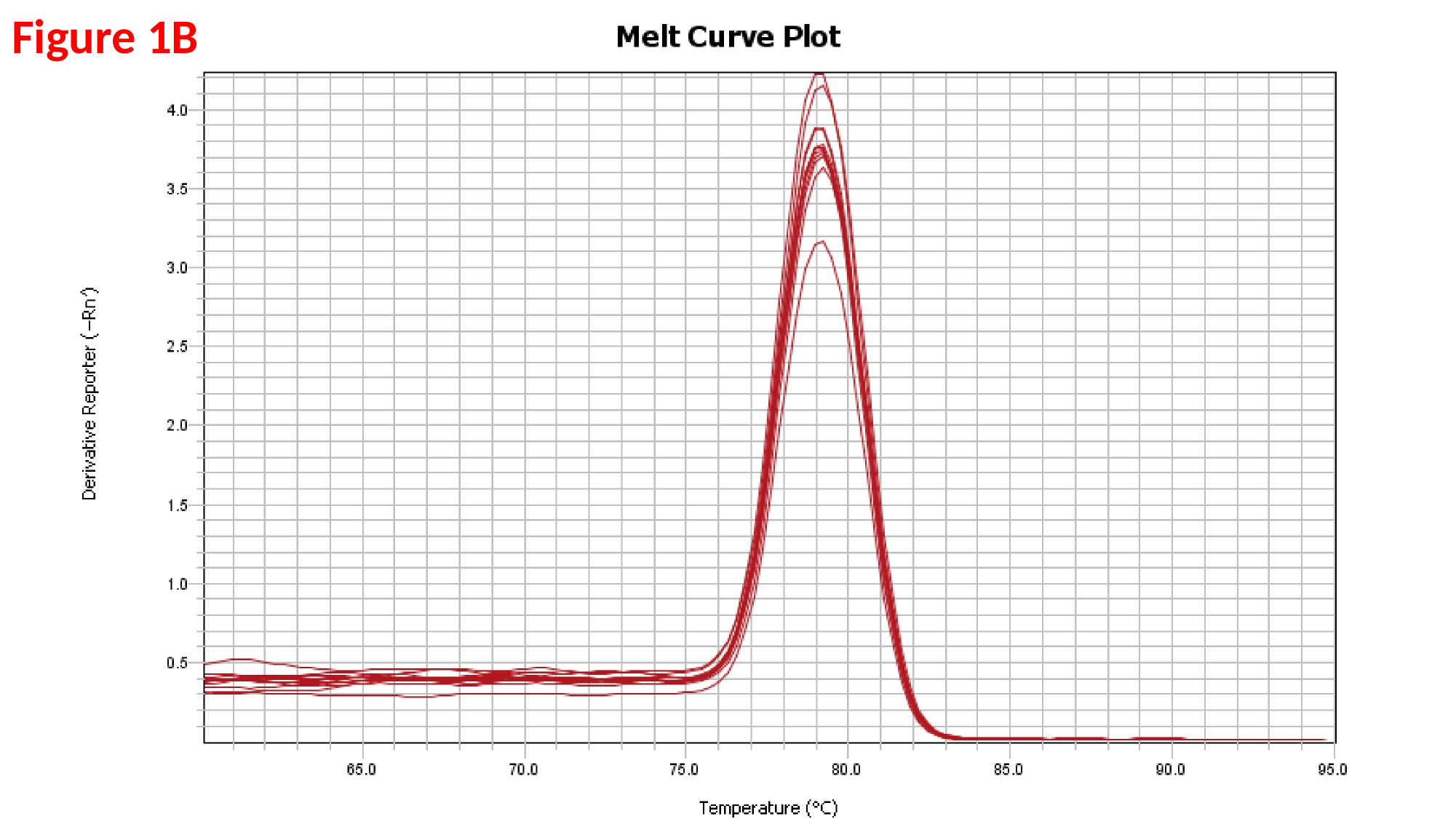

Figure 1B

## Slide 3
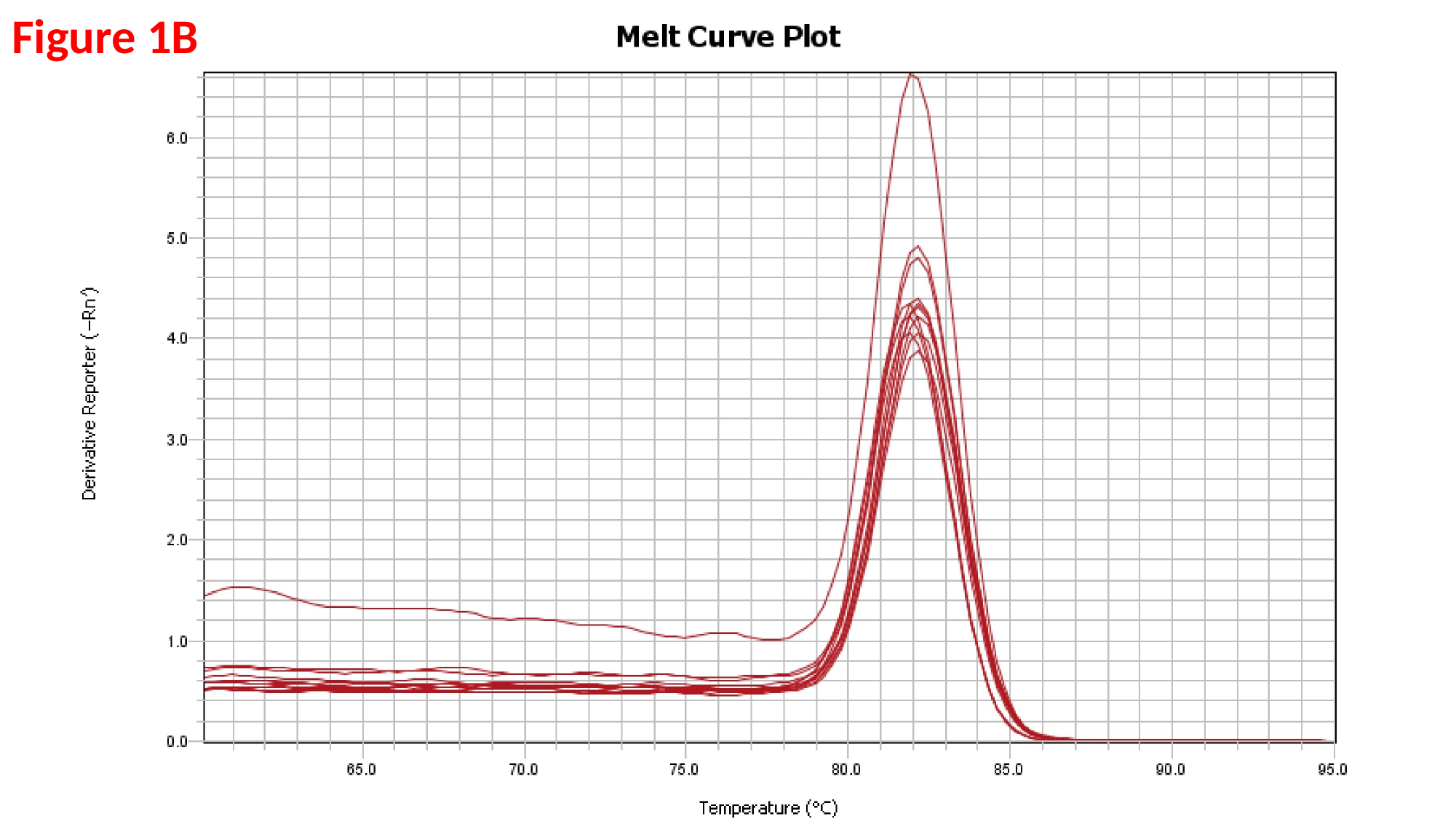

Figure 1B

## Slide 4
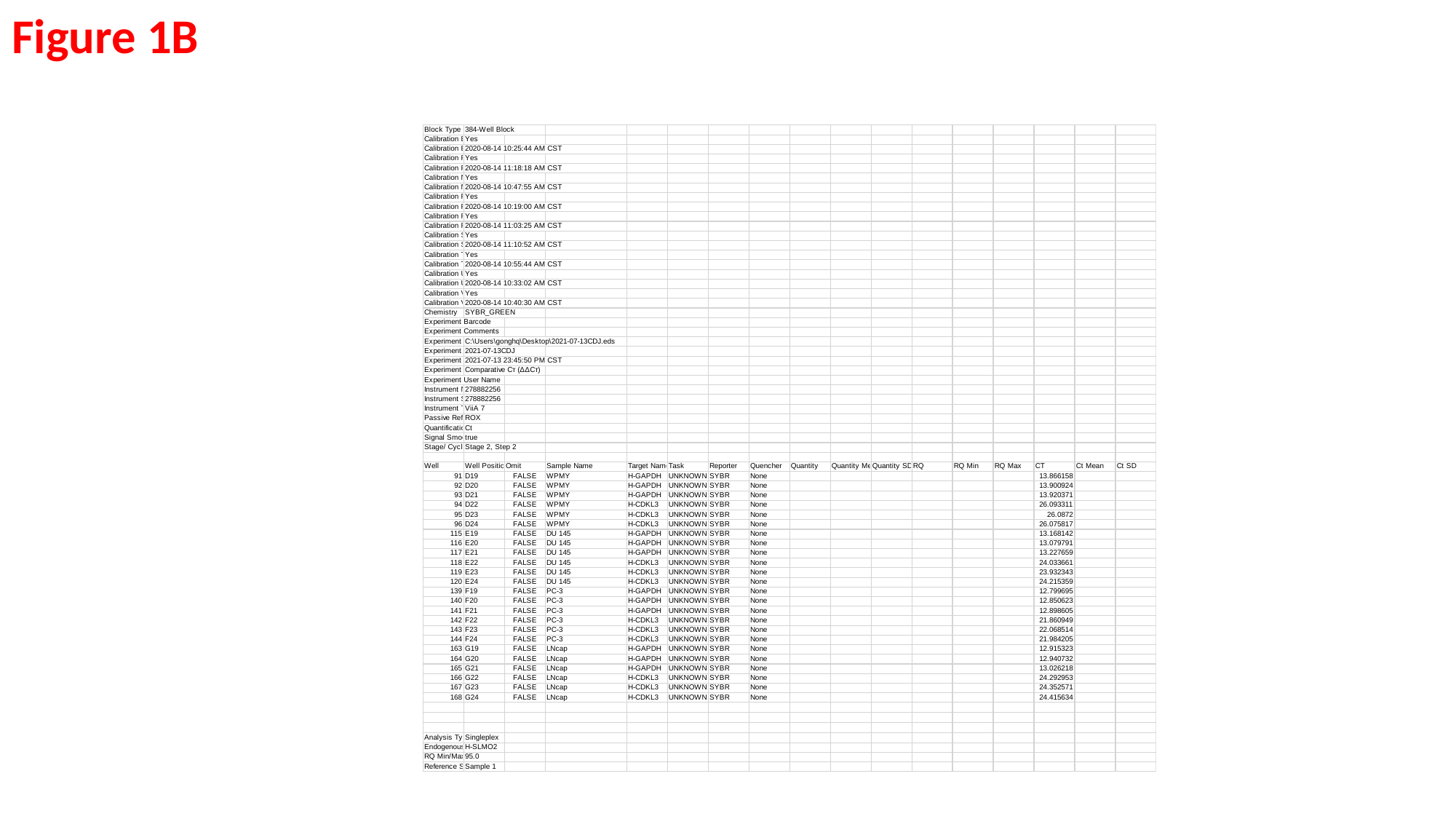

Figure 1B

## Slide 5
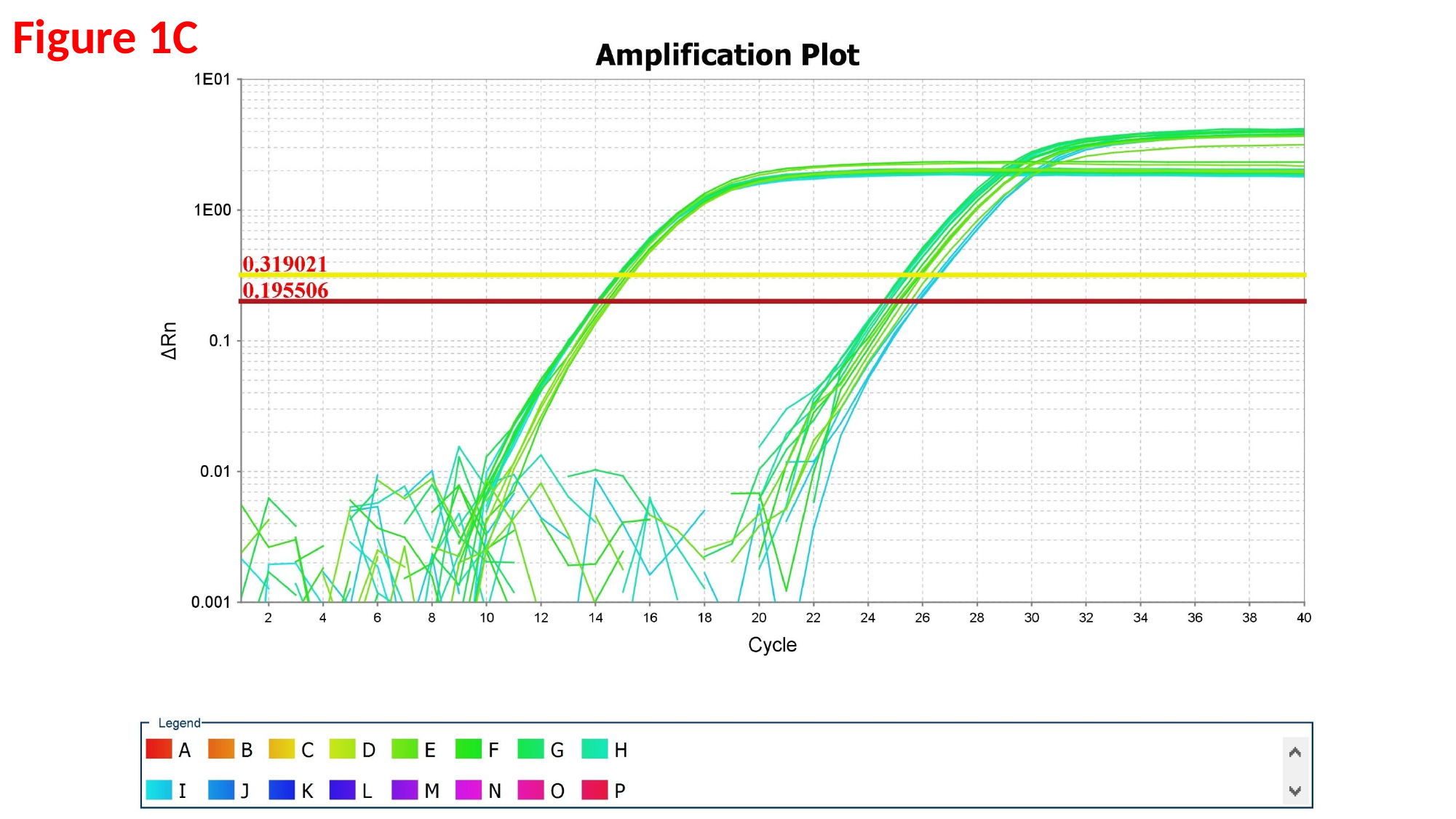

Figure 1C

## Slide 6
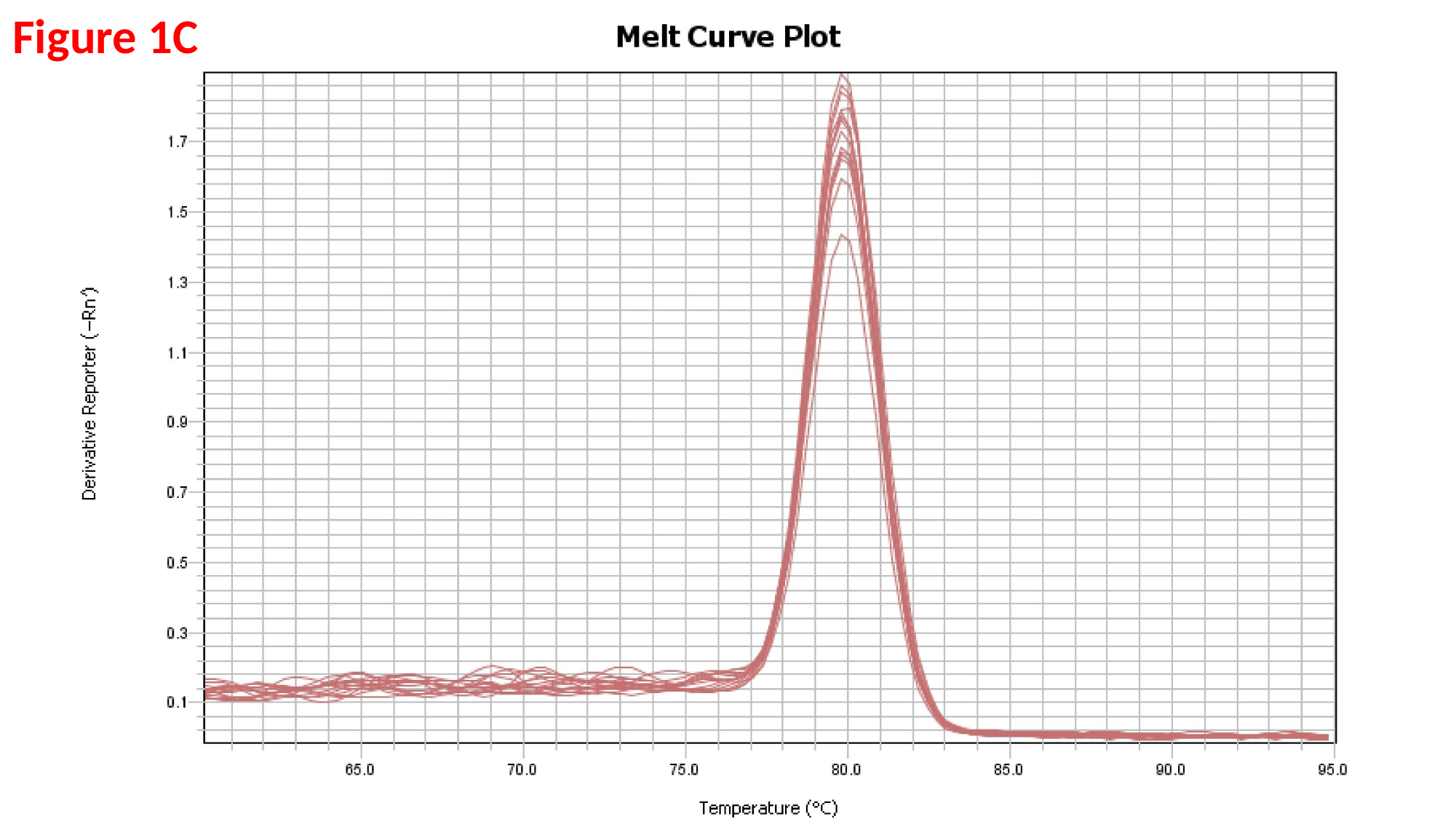

Figure 1C

## Slide 7
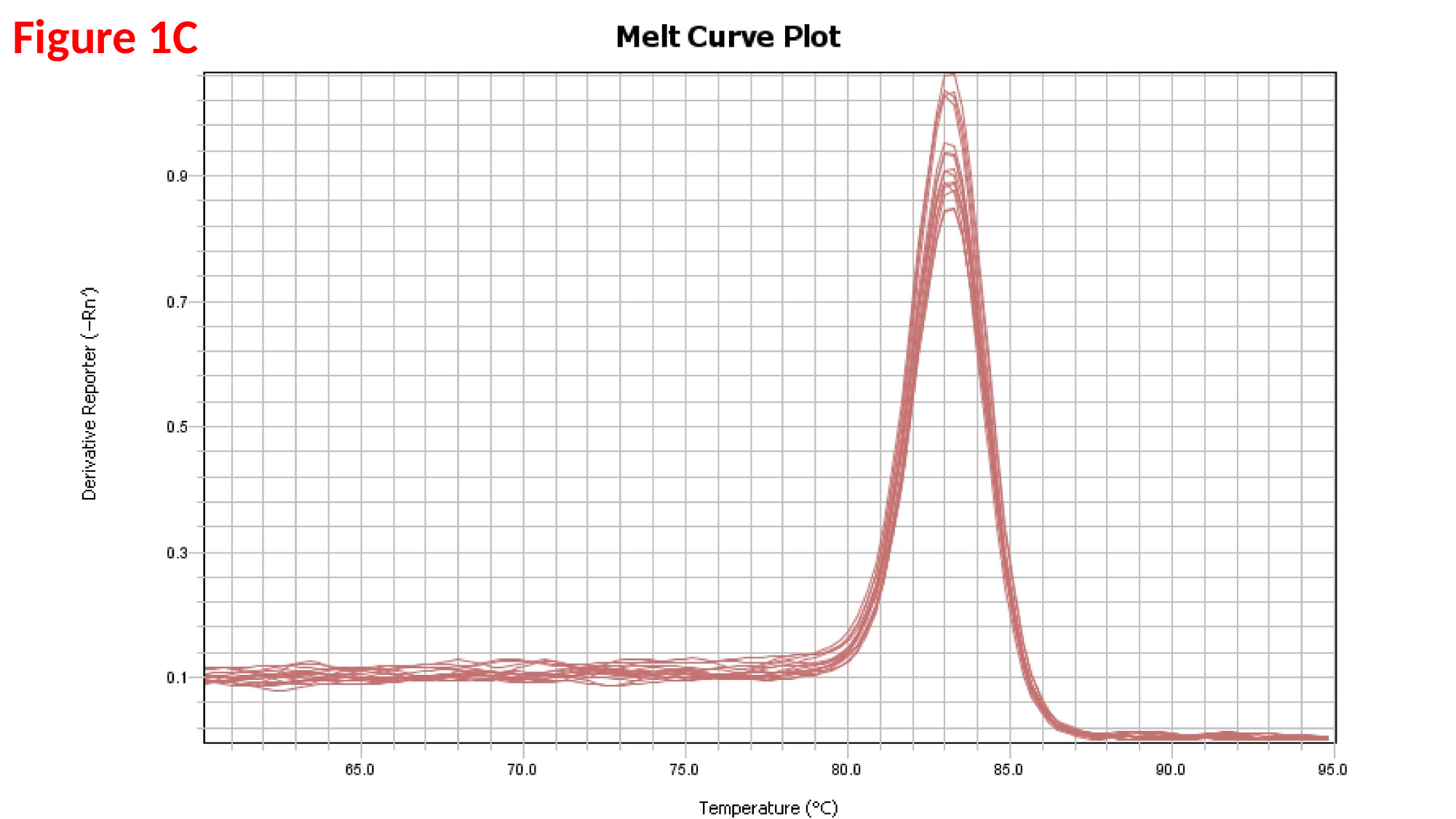

Figure 1C

## Slide 8
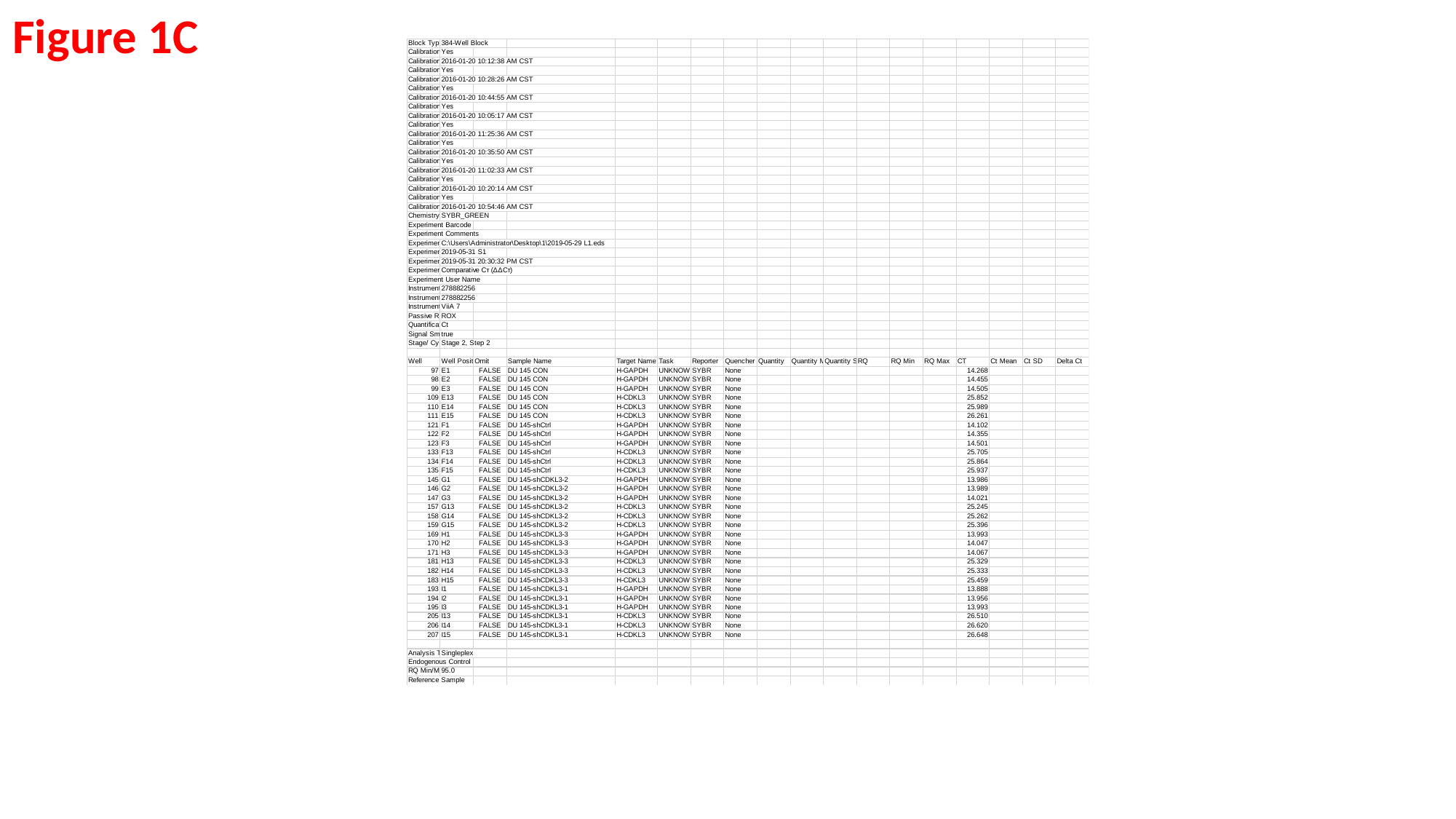

Figure 1C

## Slide 9
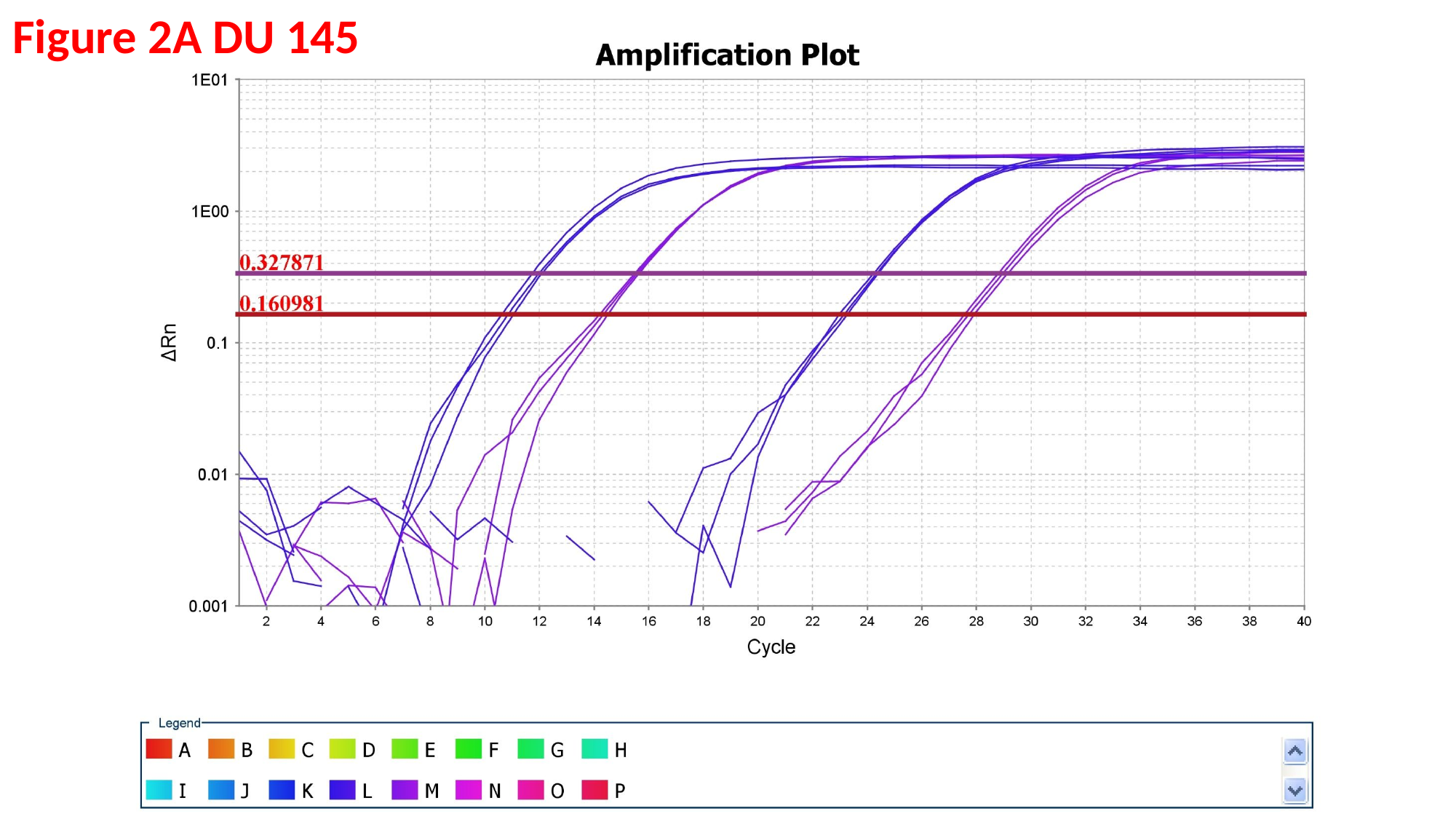

Figure 2A DU 145

## Slide 10
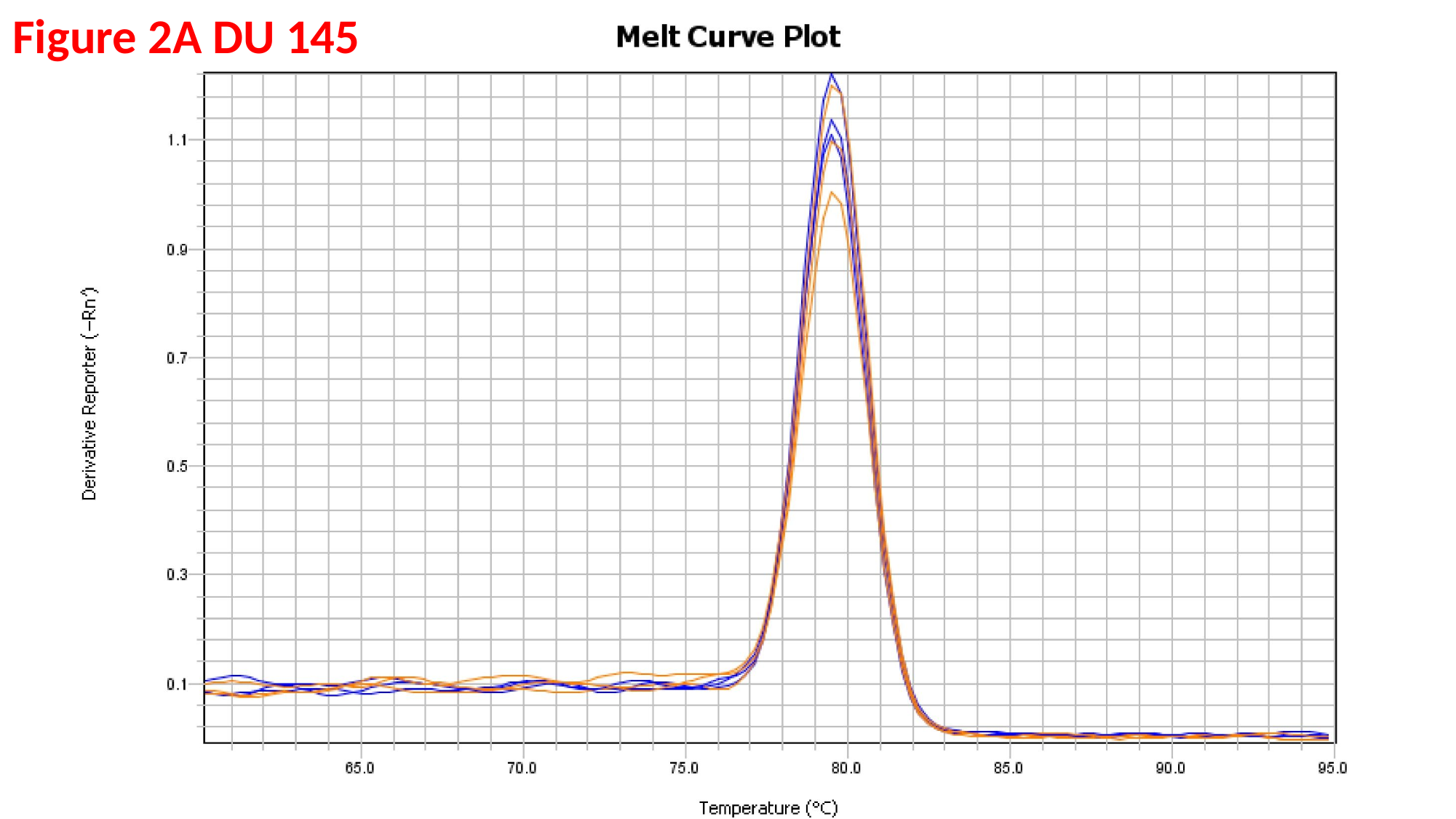

Figure 2A DU 145

## Slide 11
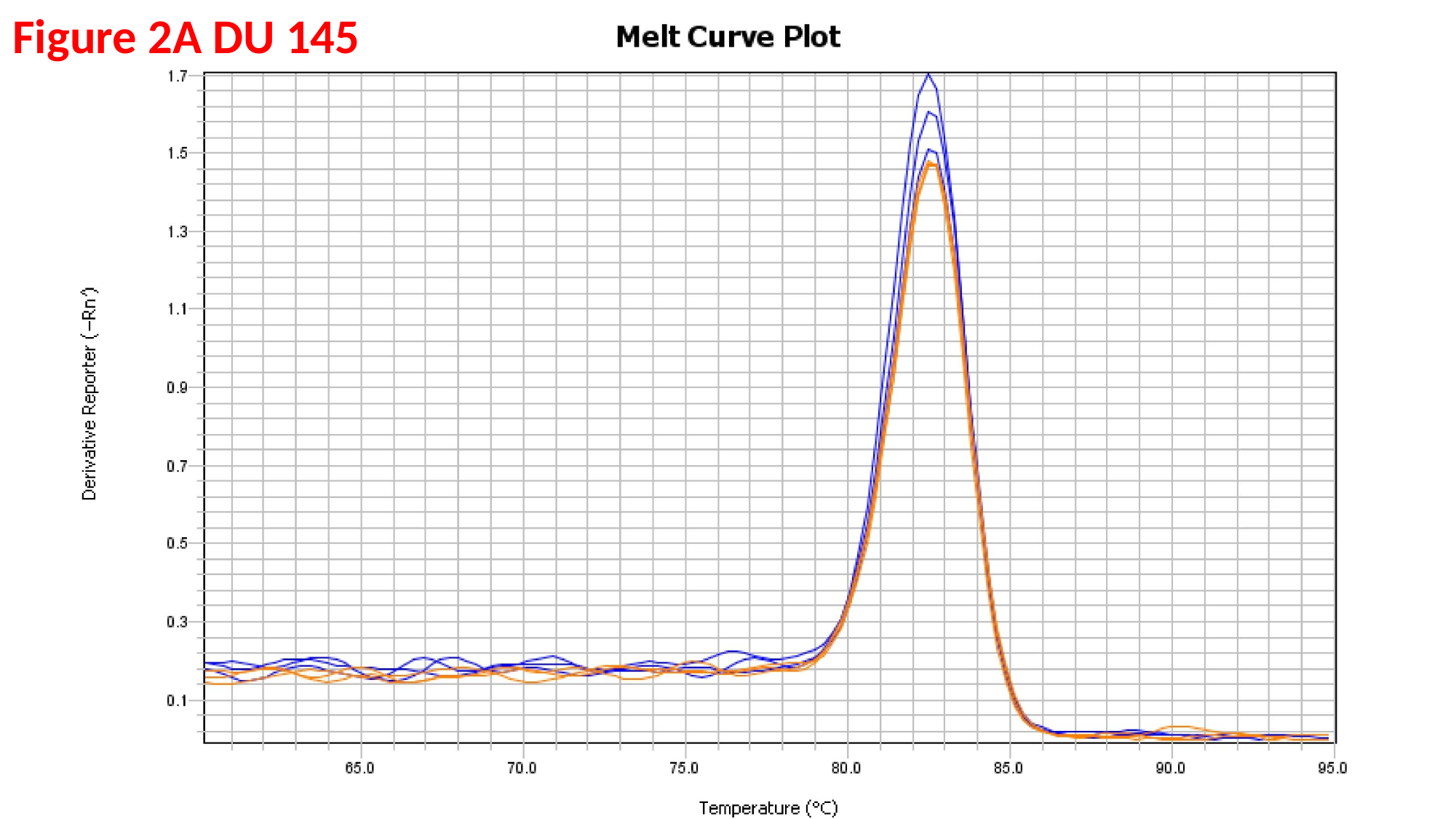

Figure 2A DU 145

## Slide 12
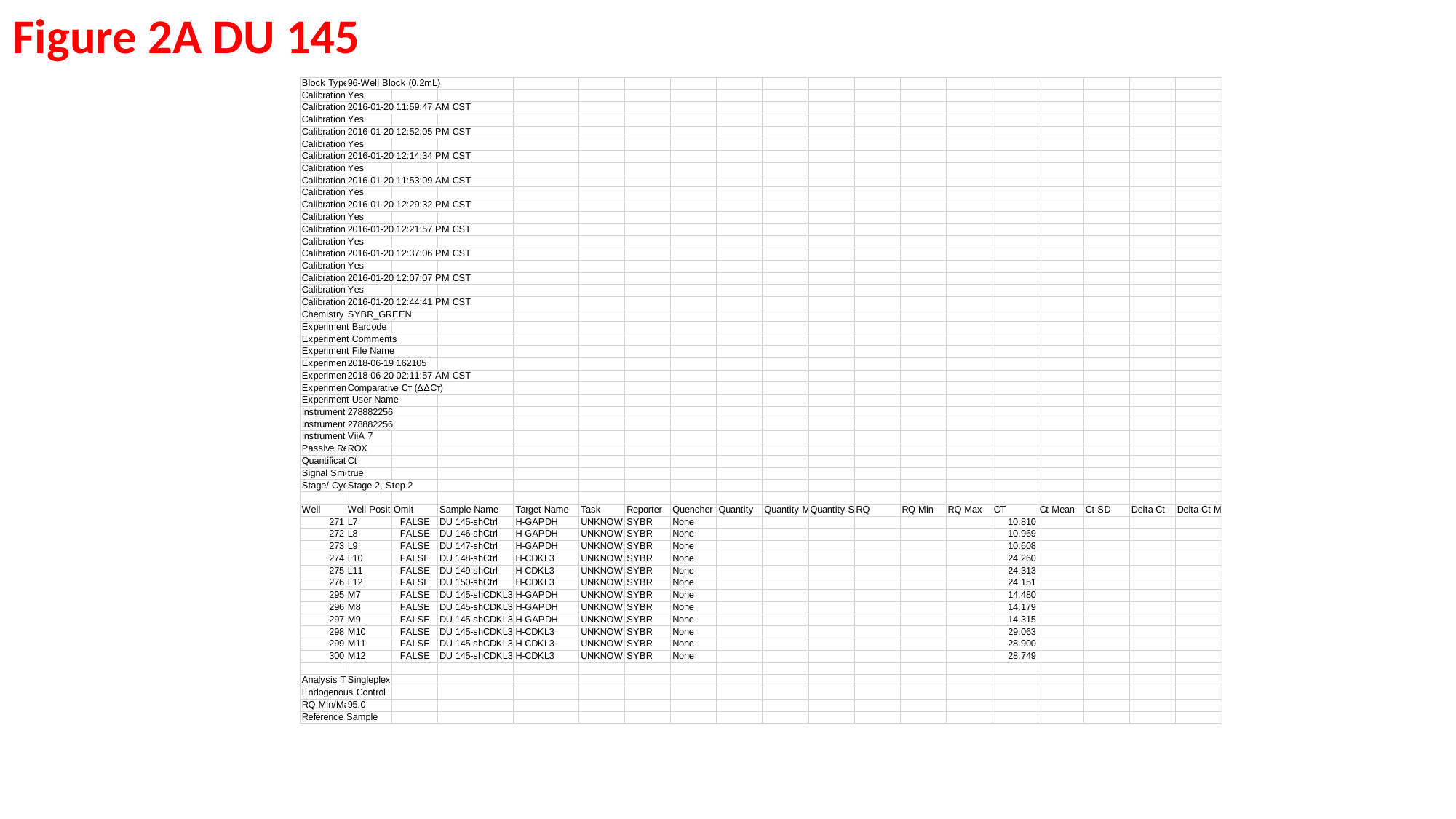

Figure 2A DU 145

## Slide 13
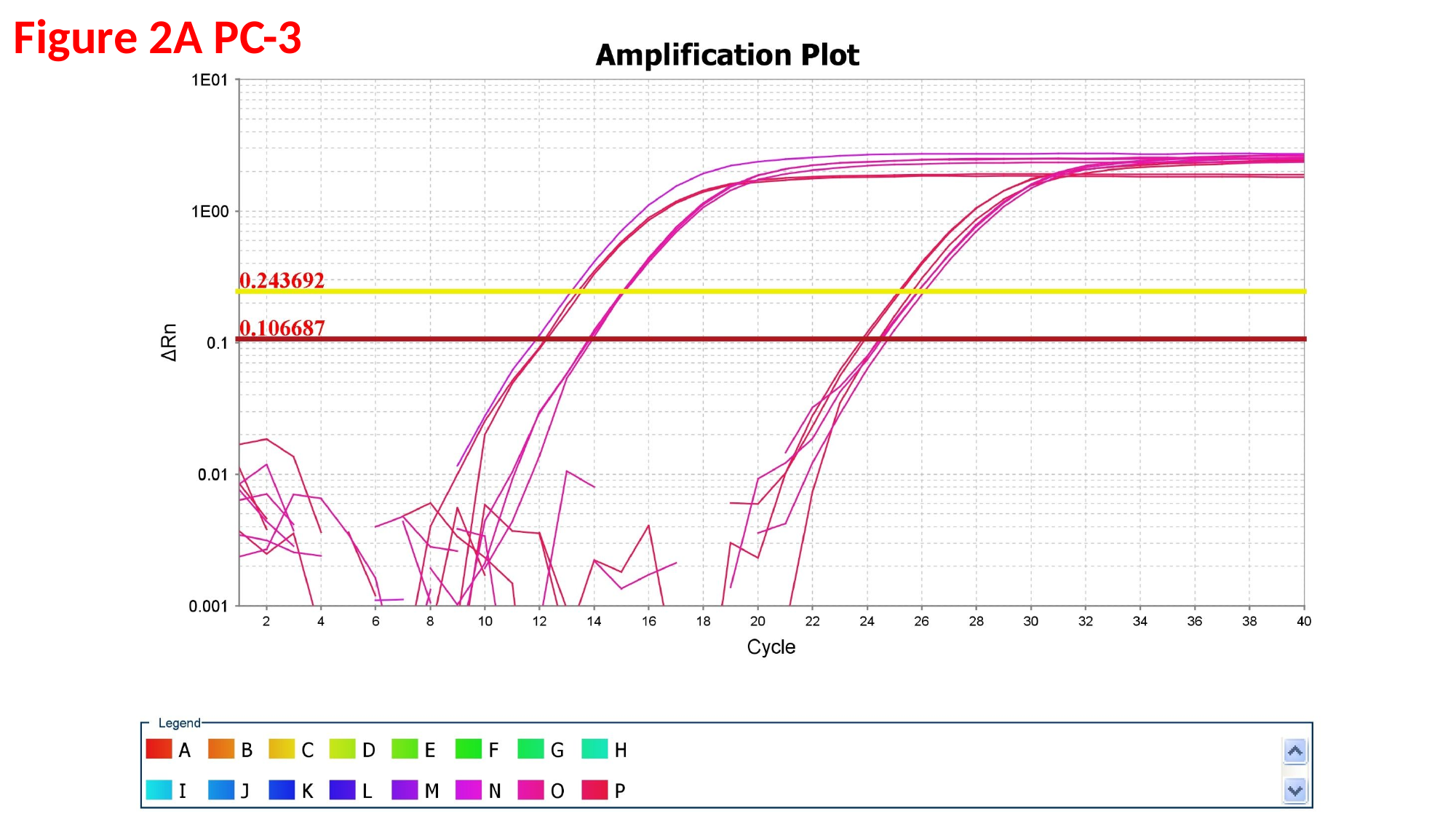

Figure 2A PC-3

## Slide 14
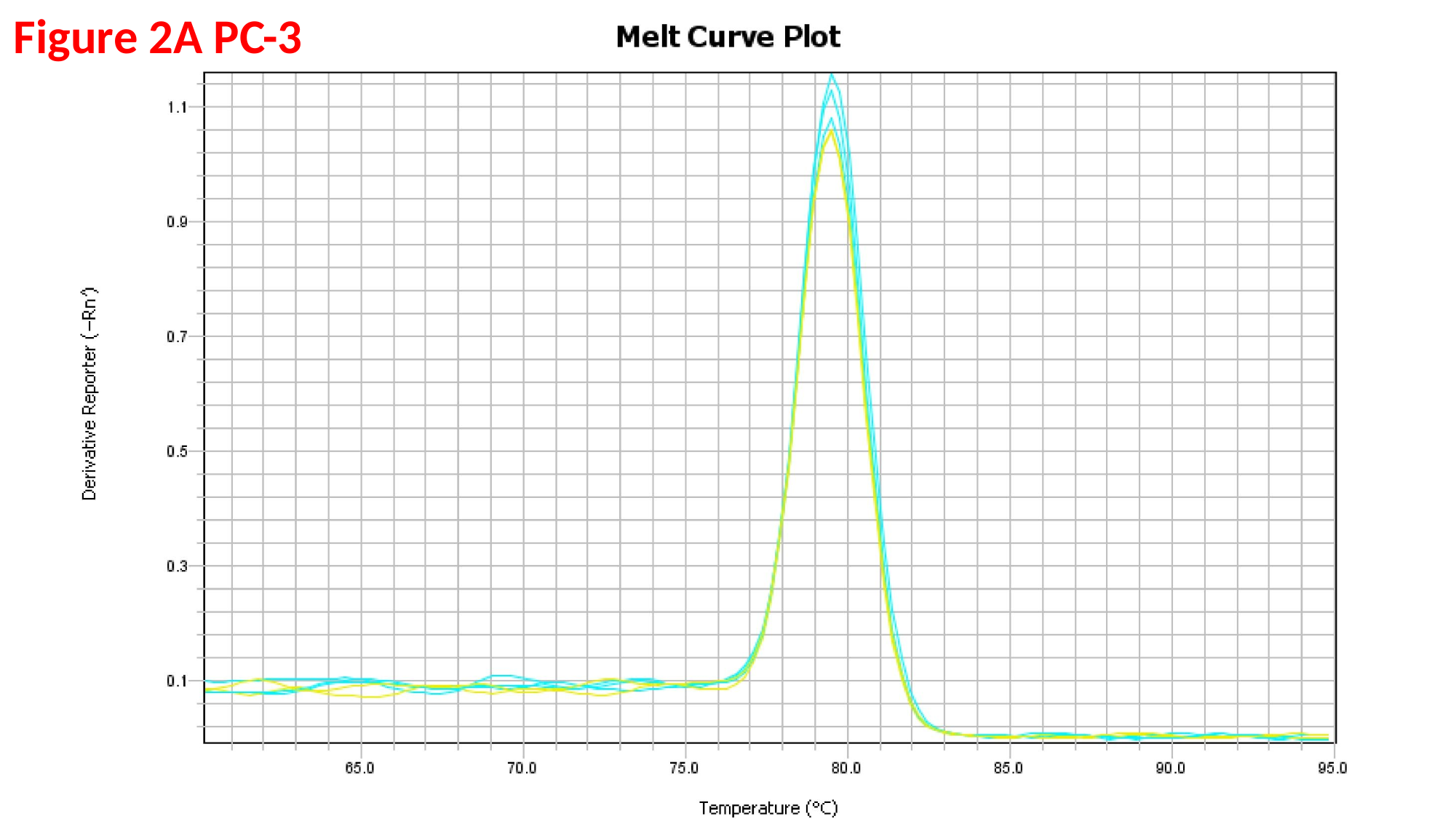

Figure 2A PC-3

## Slide 15
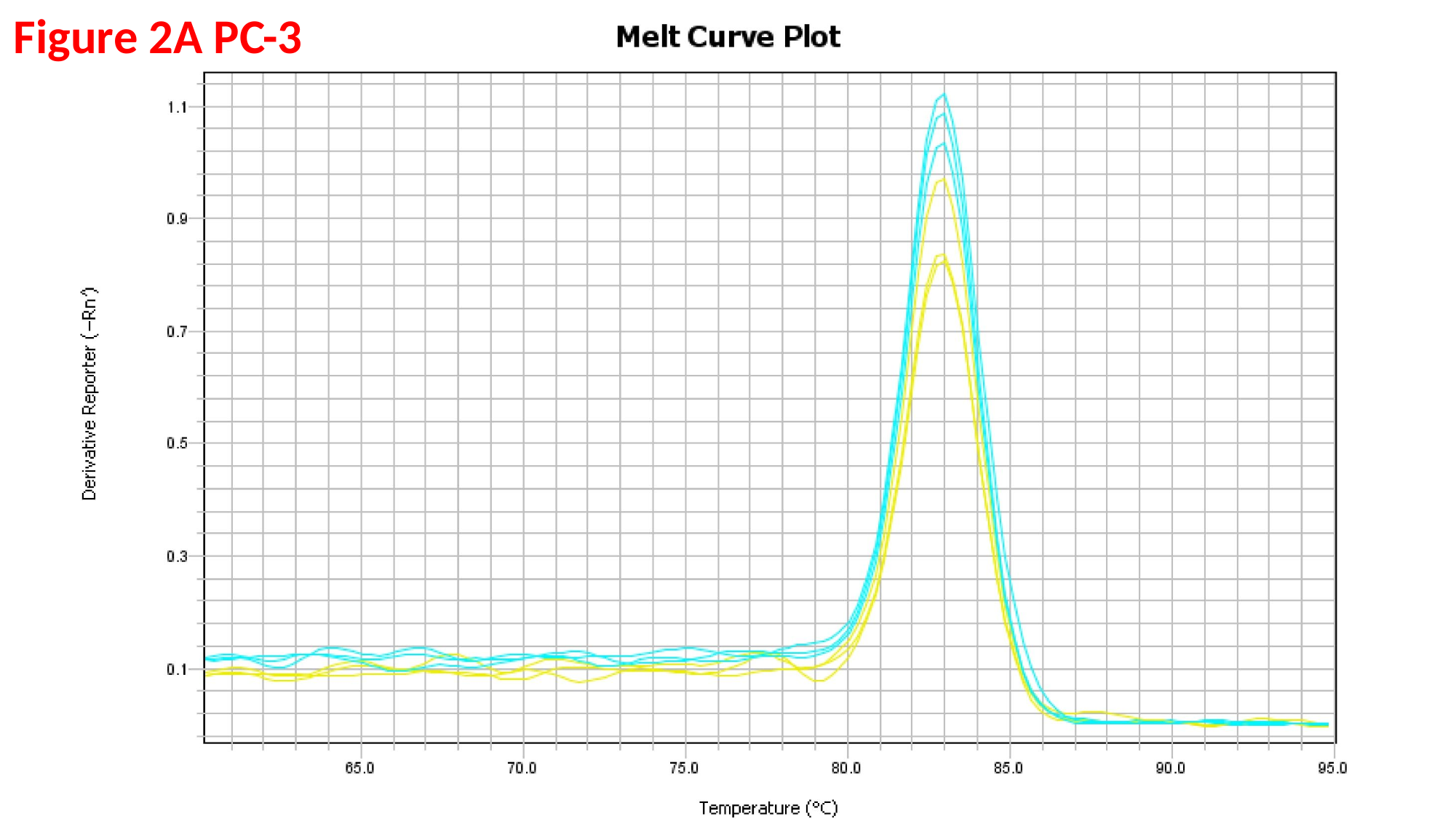

Figure 2A PC-3

## Slide 16
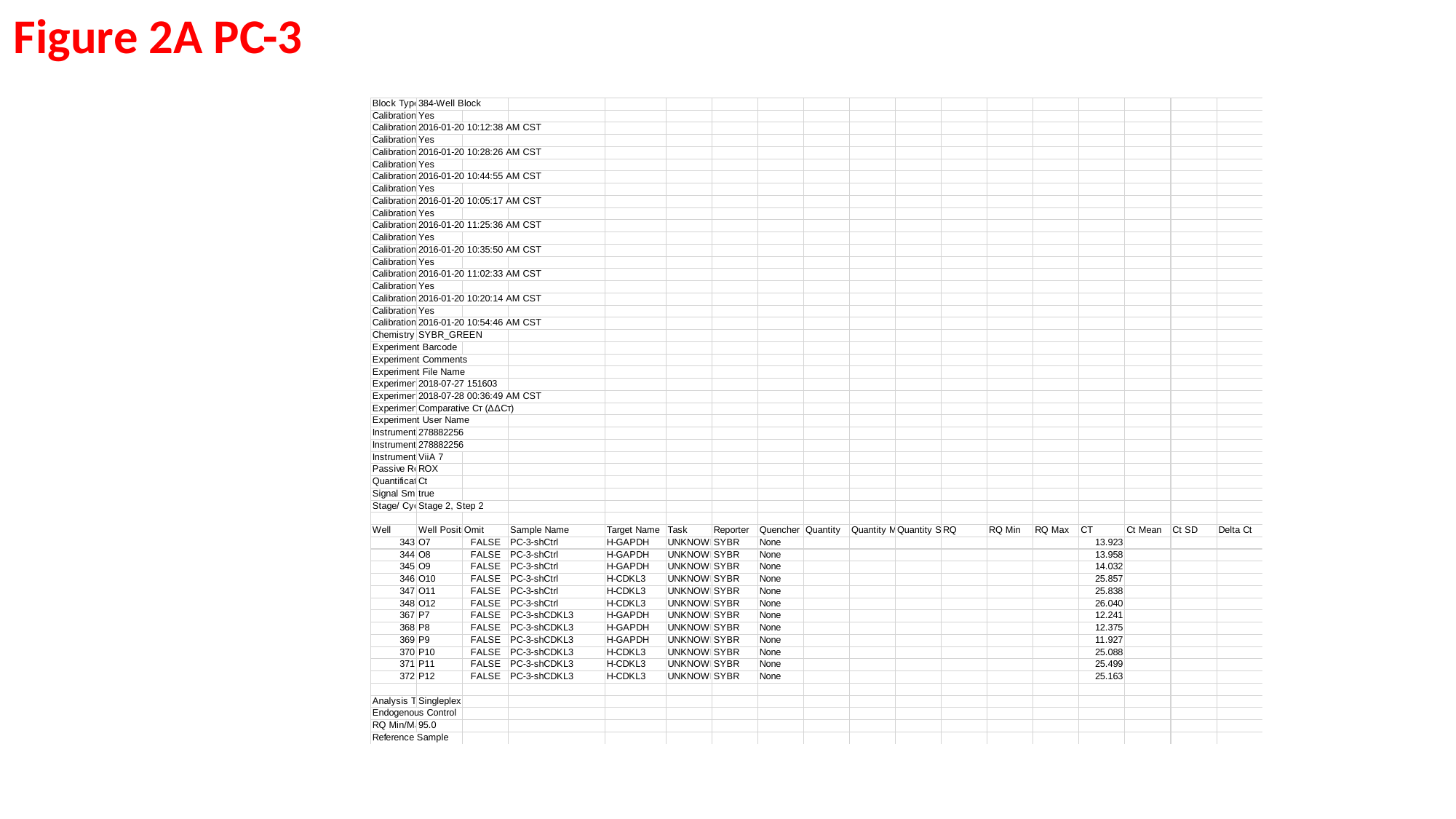

Figure 2A PC-3

## Slide 17
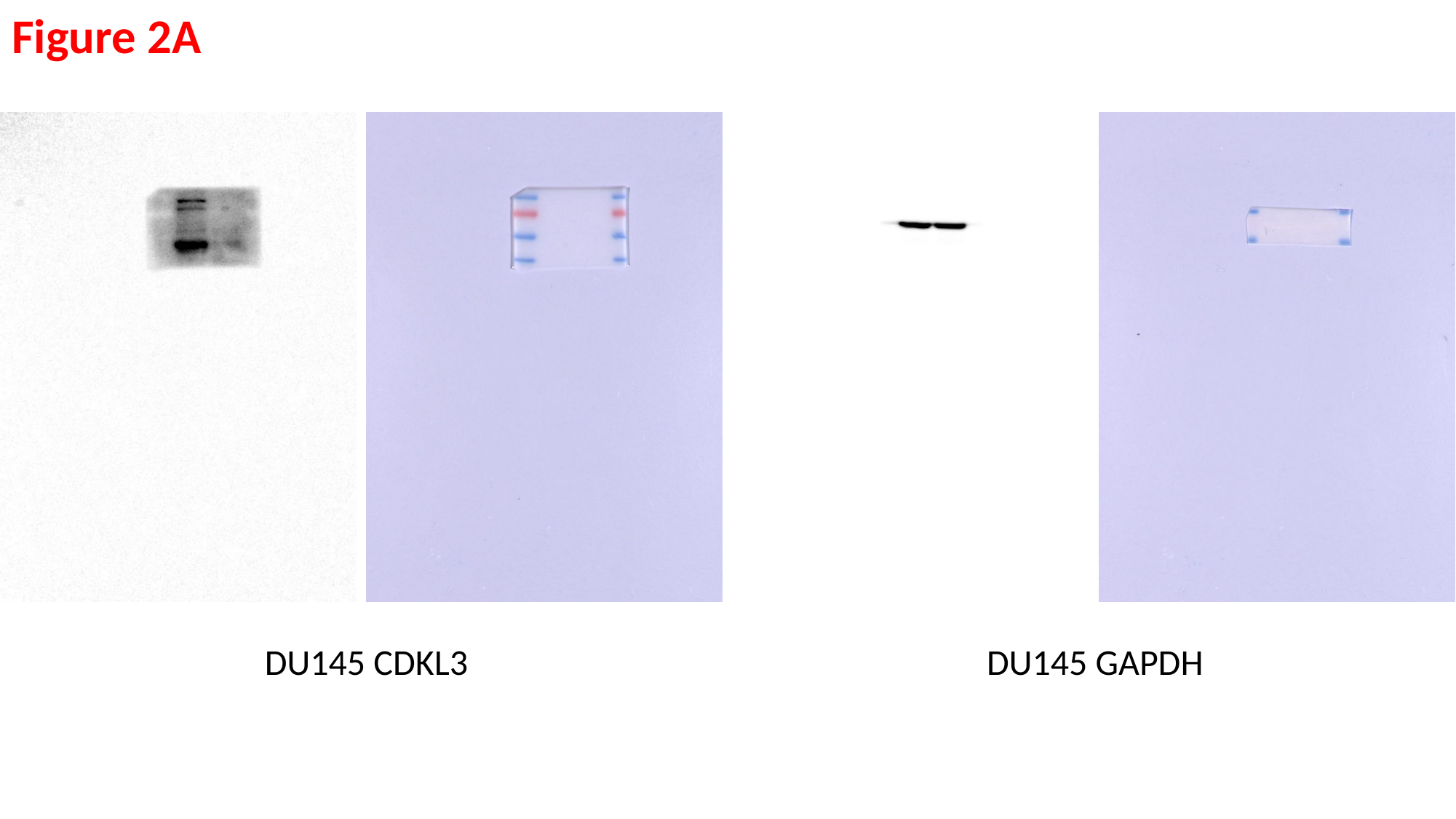

Figure 2A
DU145 CDKL3
DU145 GAPDH

## Slide 18
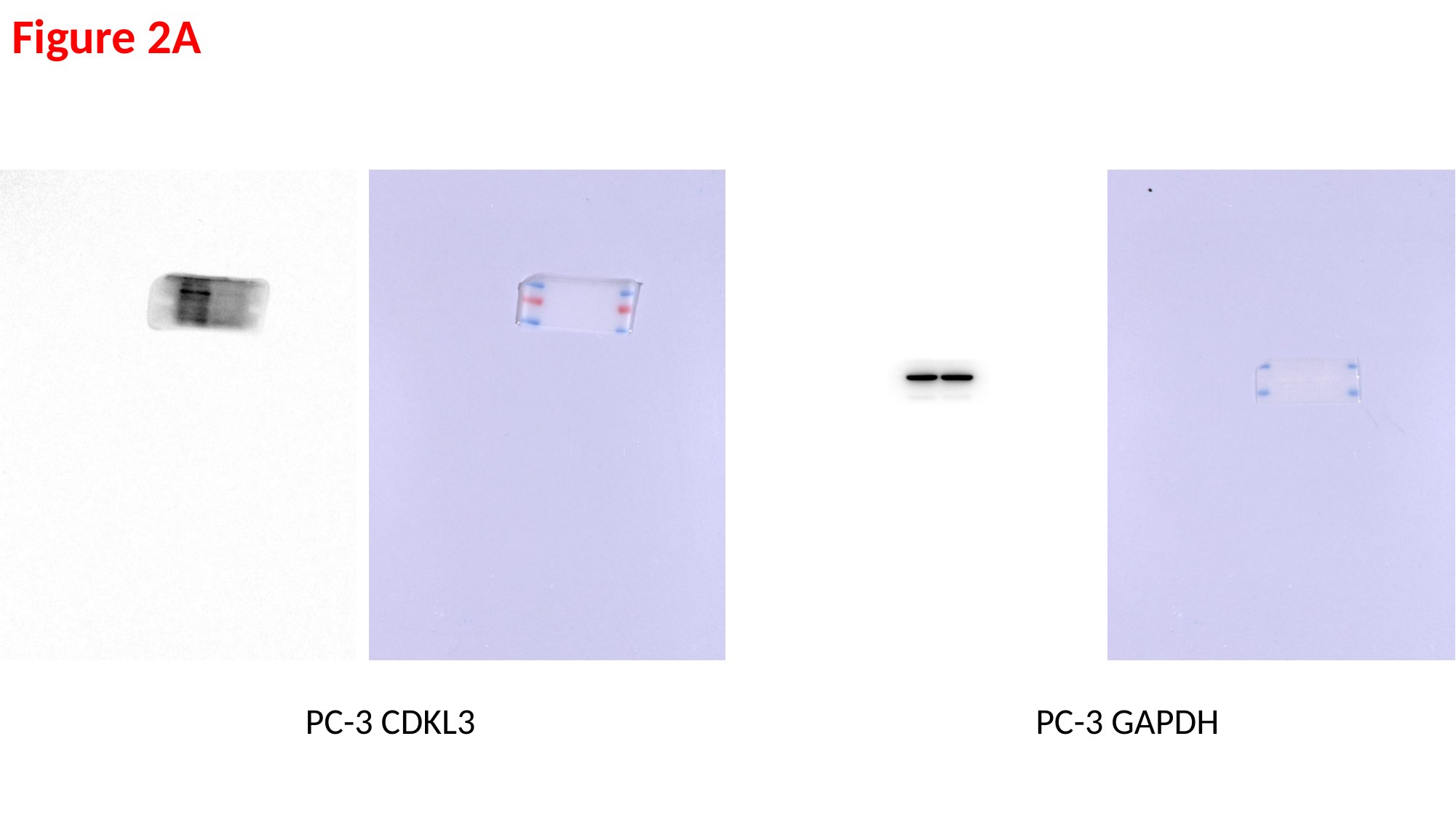

Figure 2A
PC-3 CDKL3
PC-3 GAPDH

## Slide 19
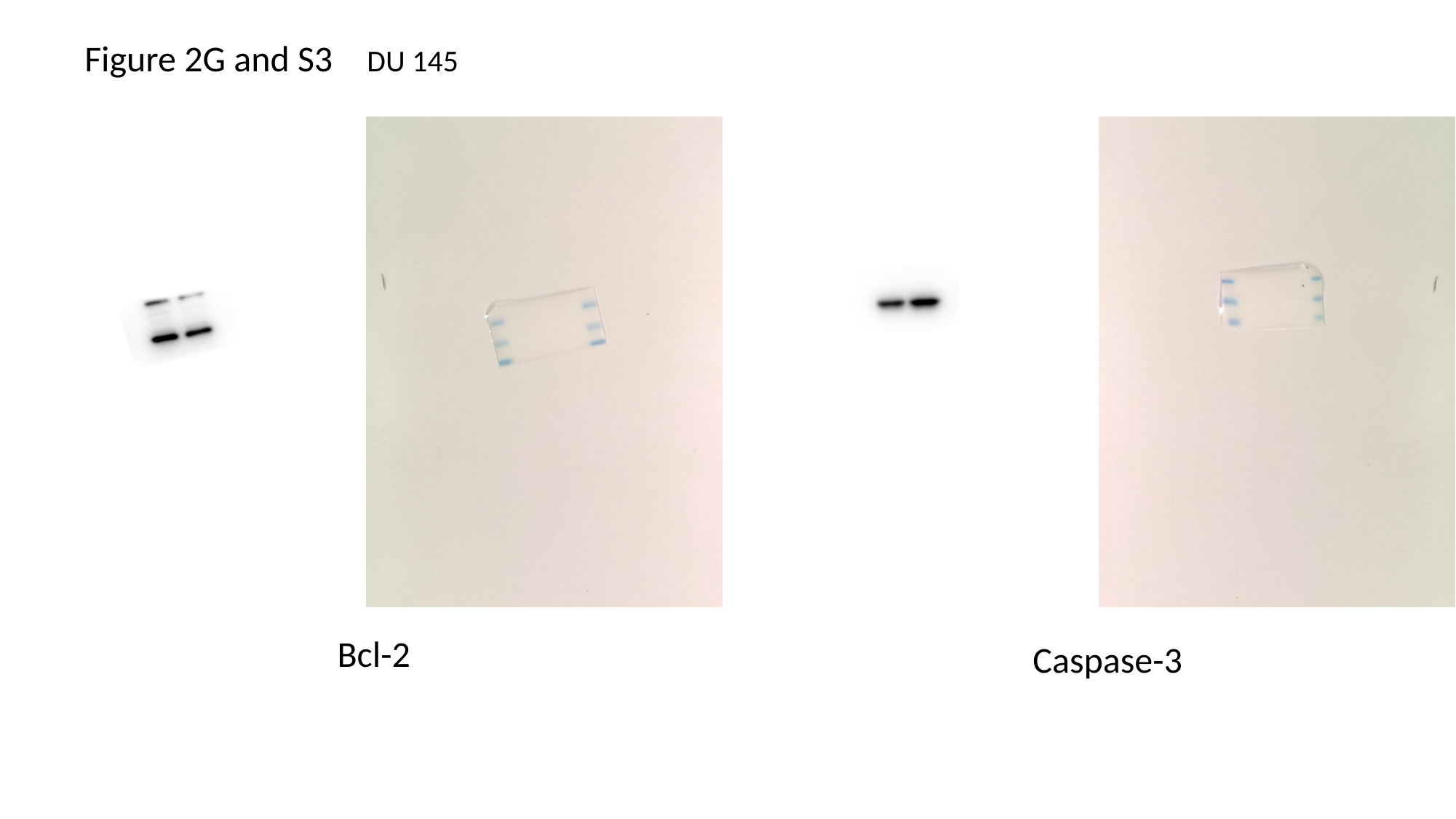

Figure 2G and S3
DU 145
Bcl-2
Caspase-3

## Slide 20
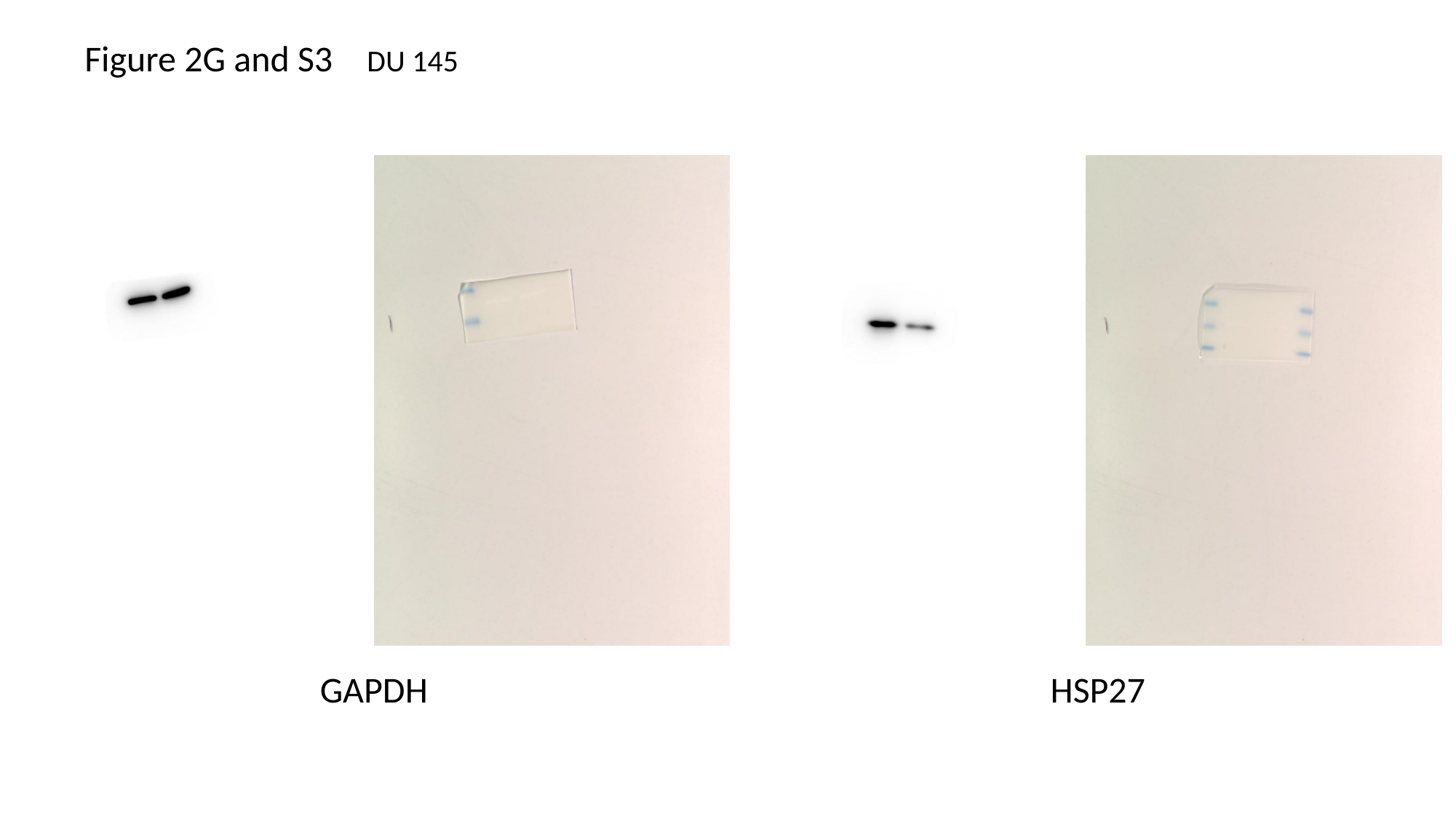

Figure 2G and S3
DU 145
GAPDH
HSP27

## Slide 21
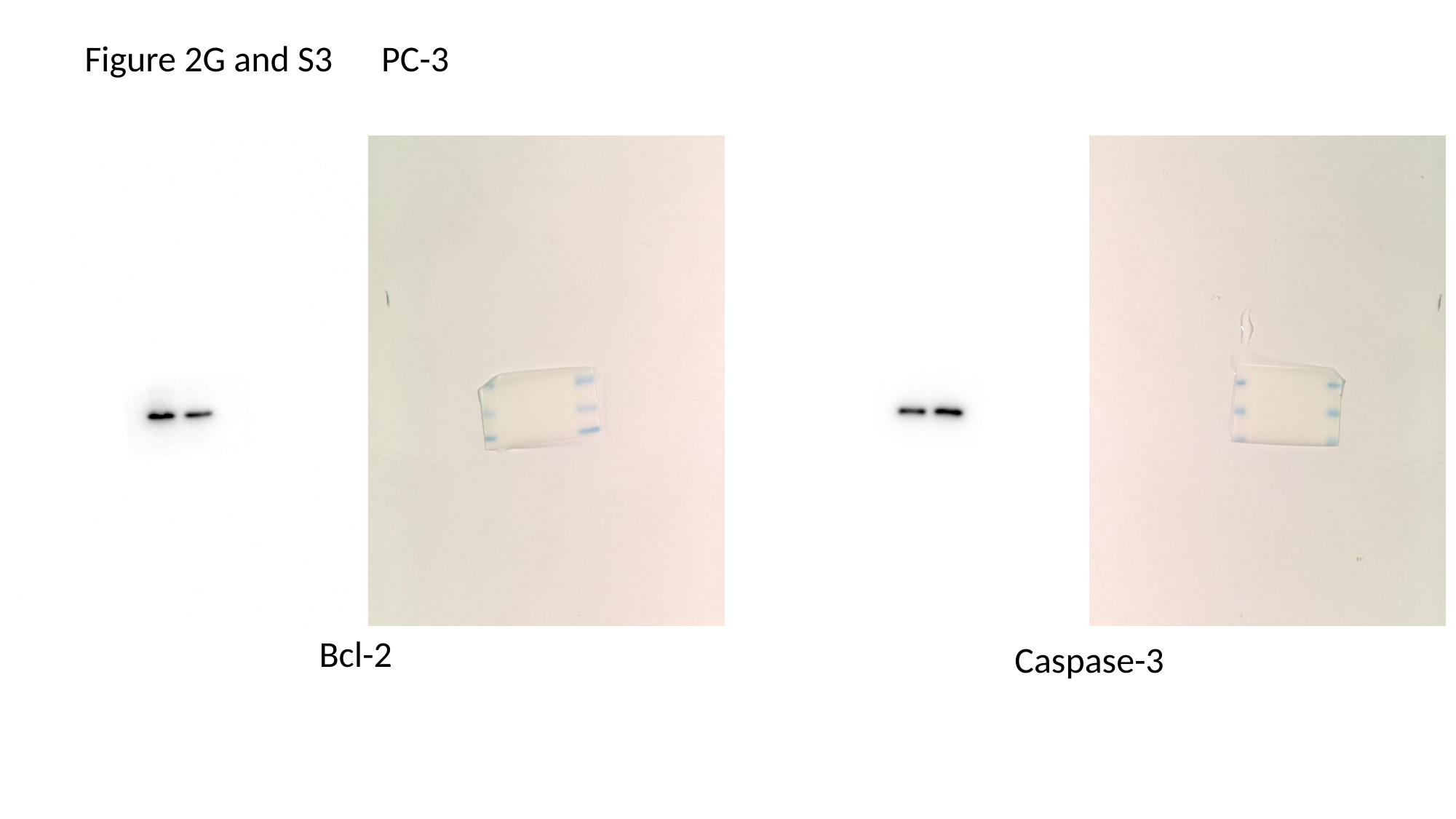

Figure 2G and S3
PC-3
Bcl-2
Caspase-3

## Slide 22
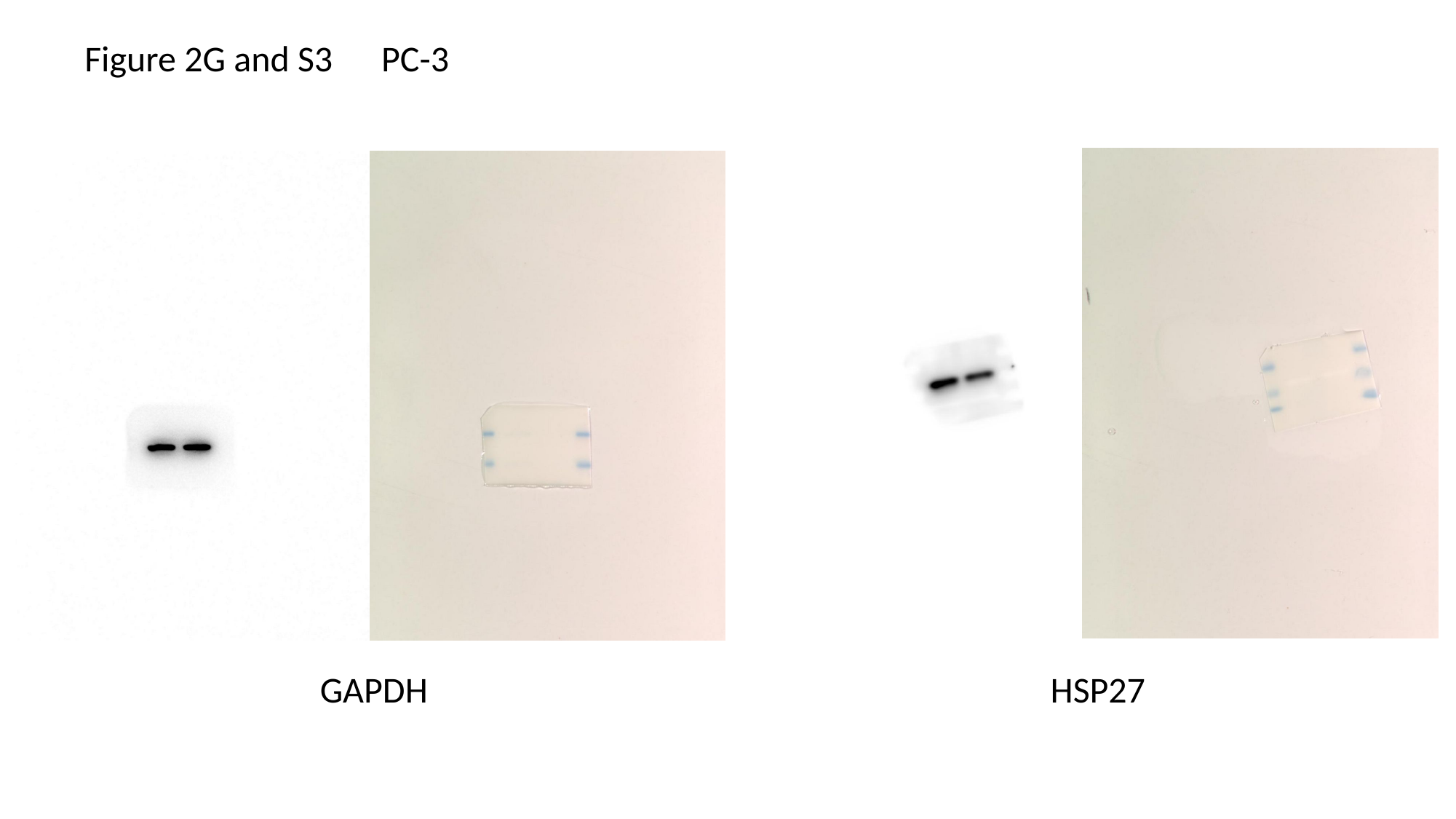

Figure 2G and S3
PC-3
GAPDH
HSP27

## Slide 23
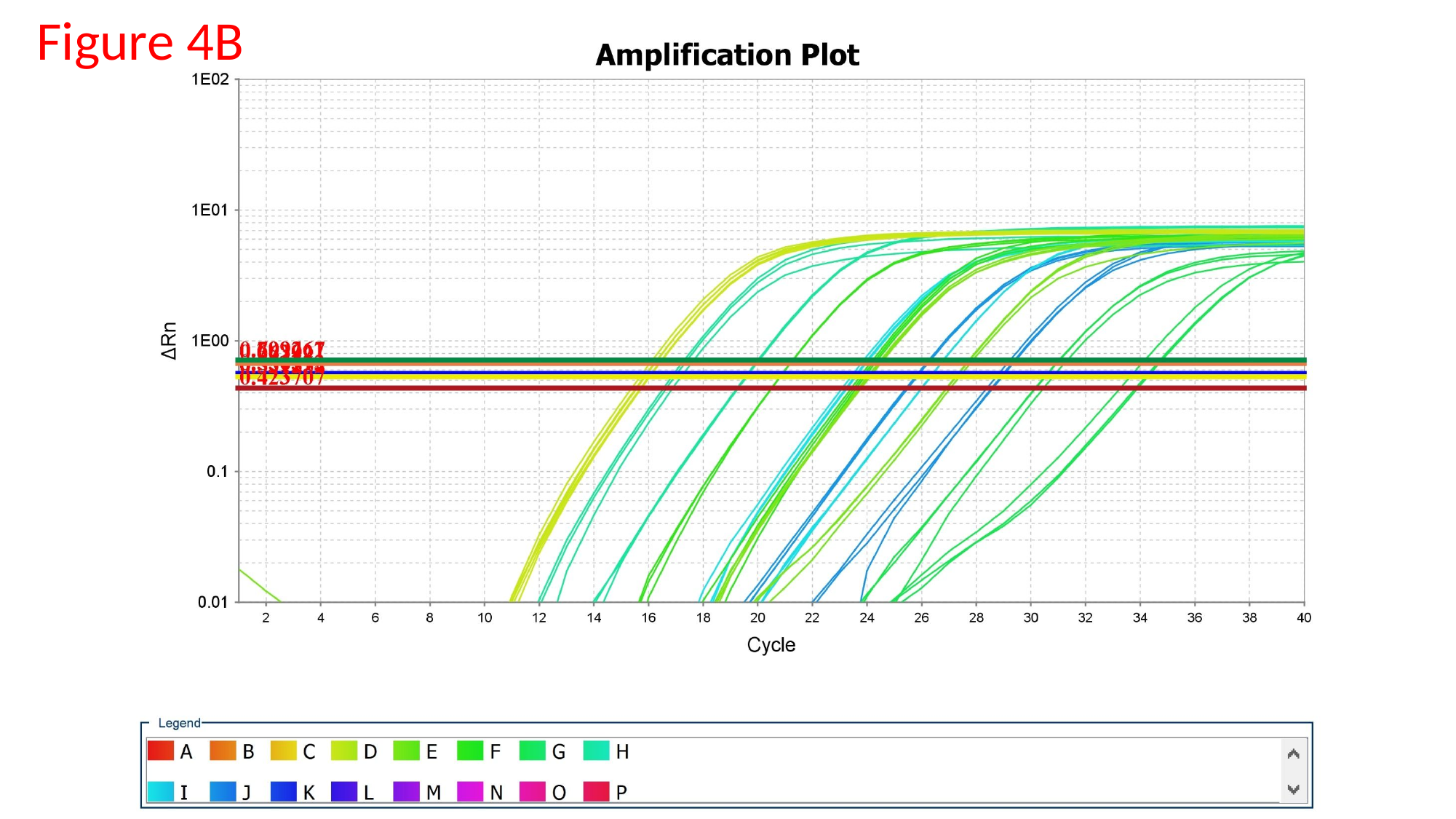

Figure 4B

## Slide 24
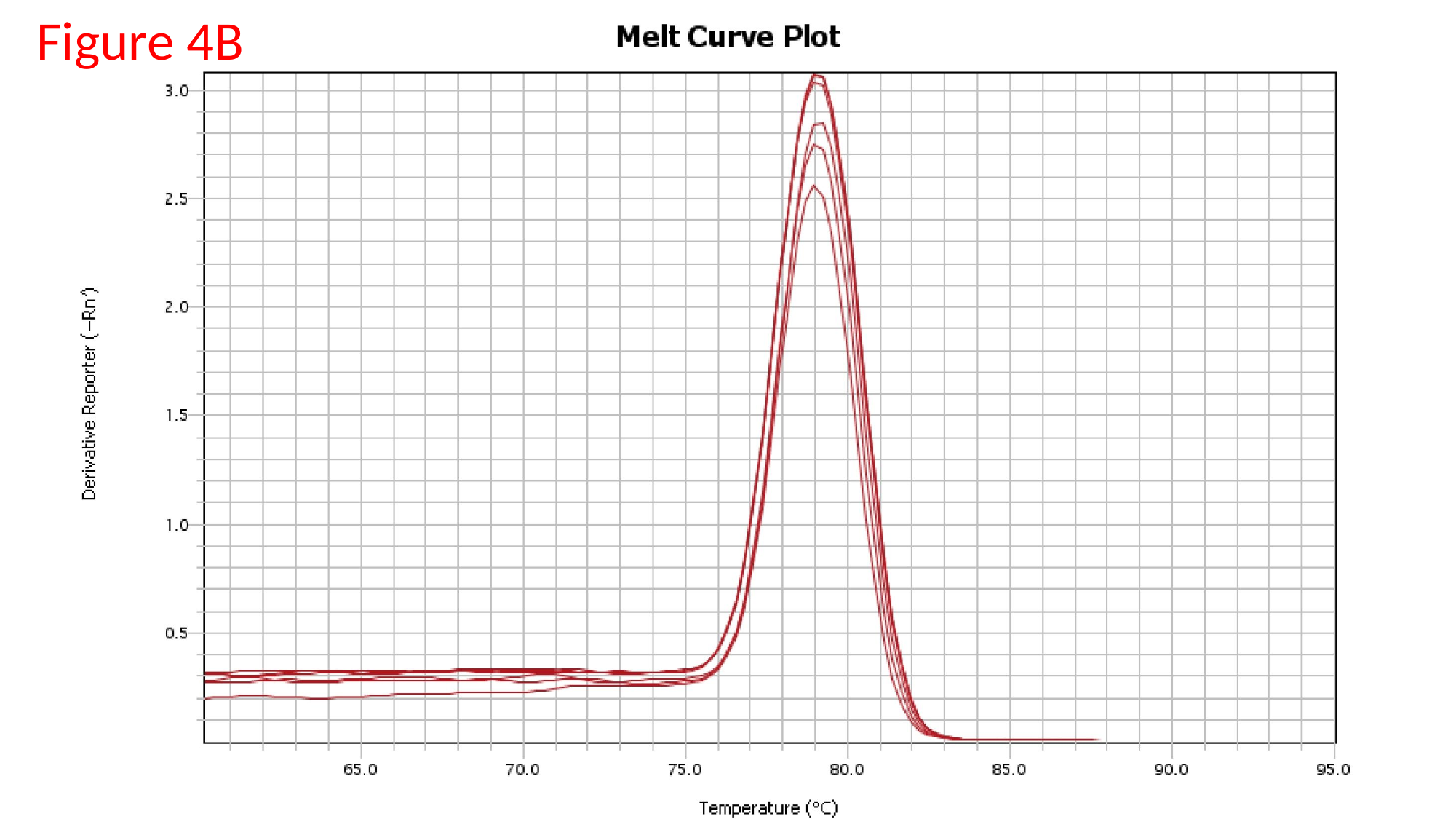

Figure 4B

## Slide 25
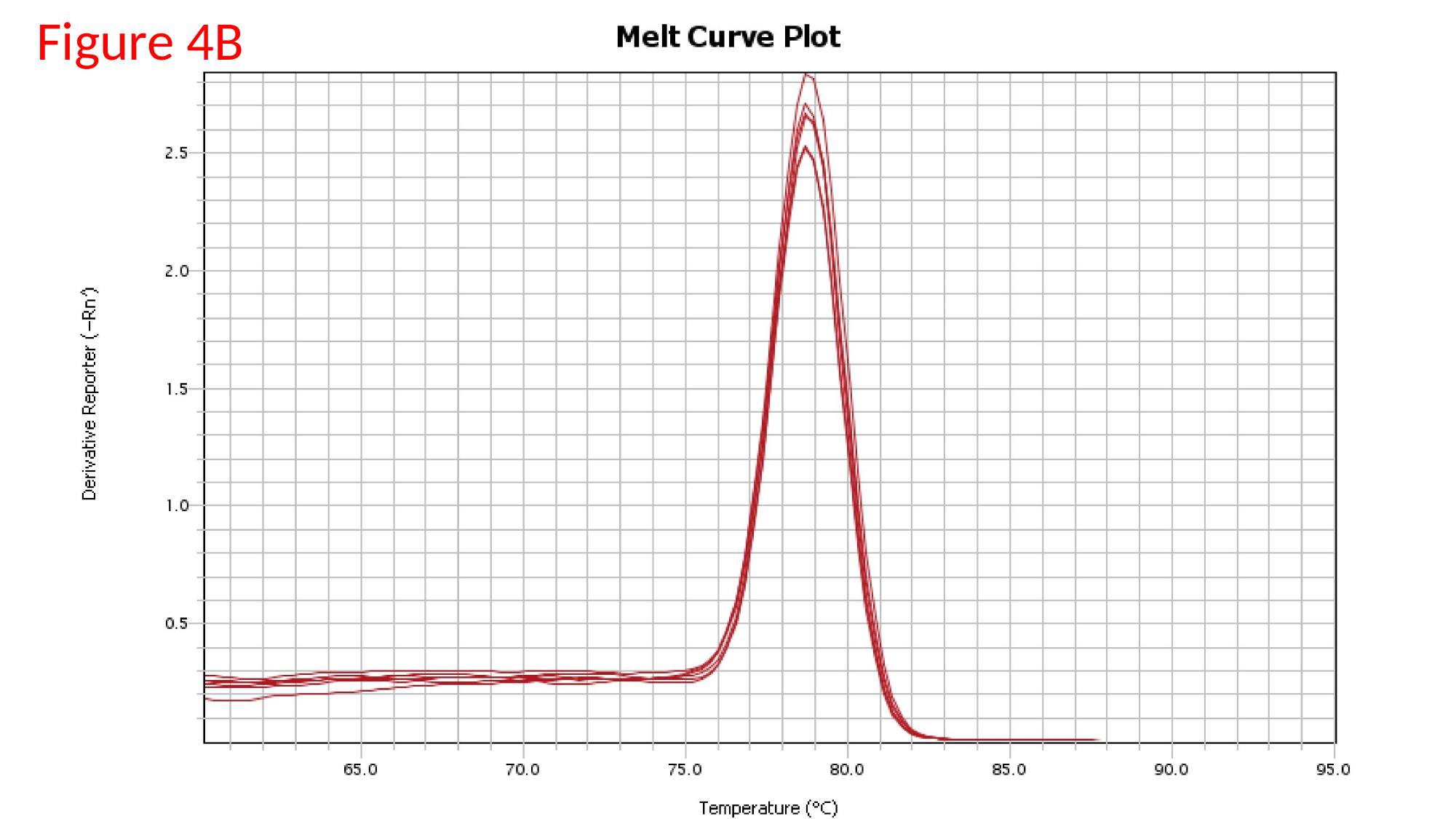

Figure 4B

## Slide 26
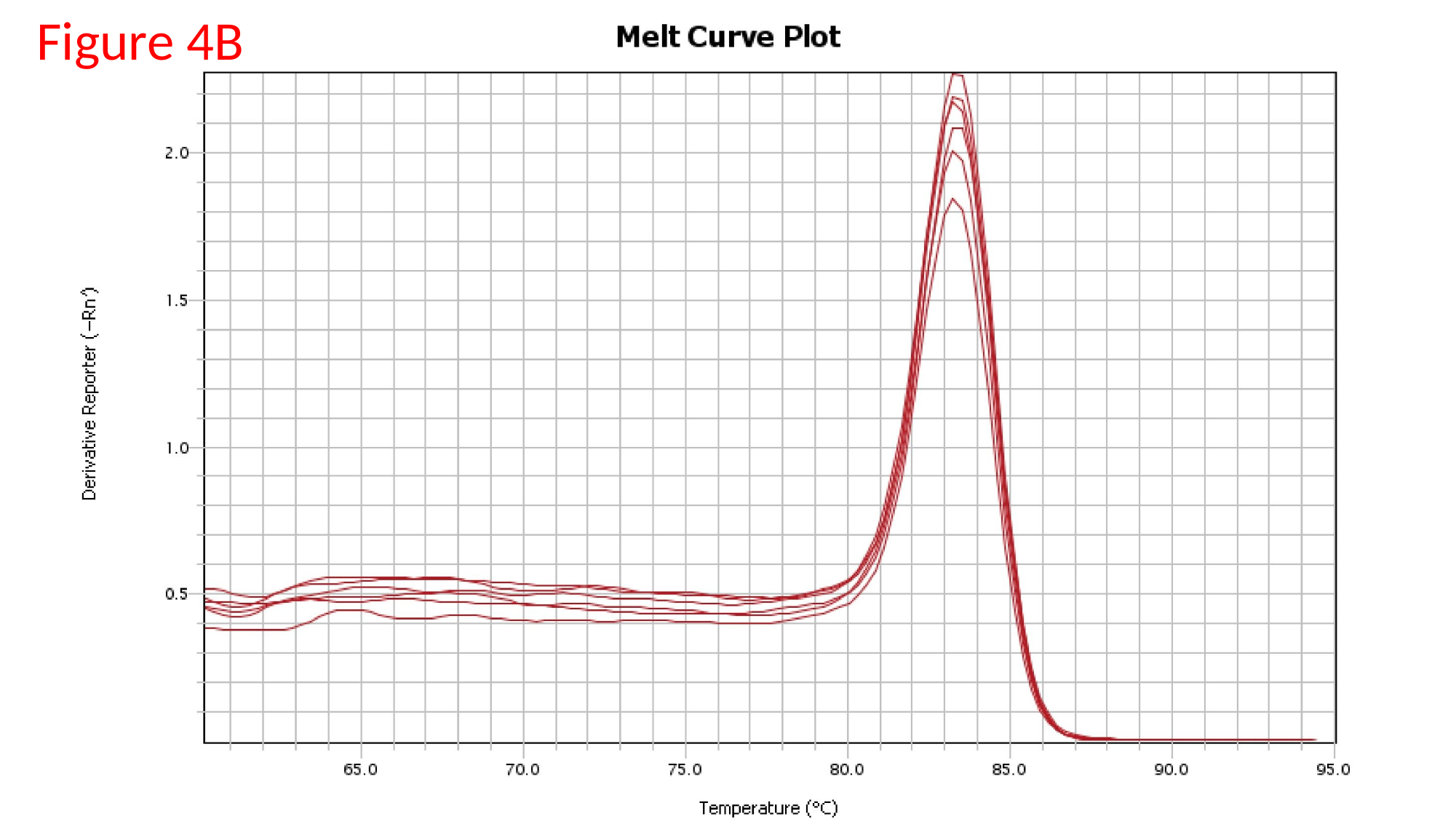

Figure 4B

## Slide 27
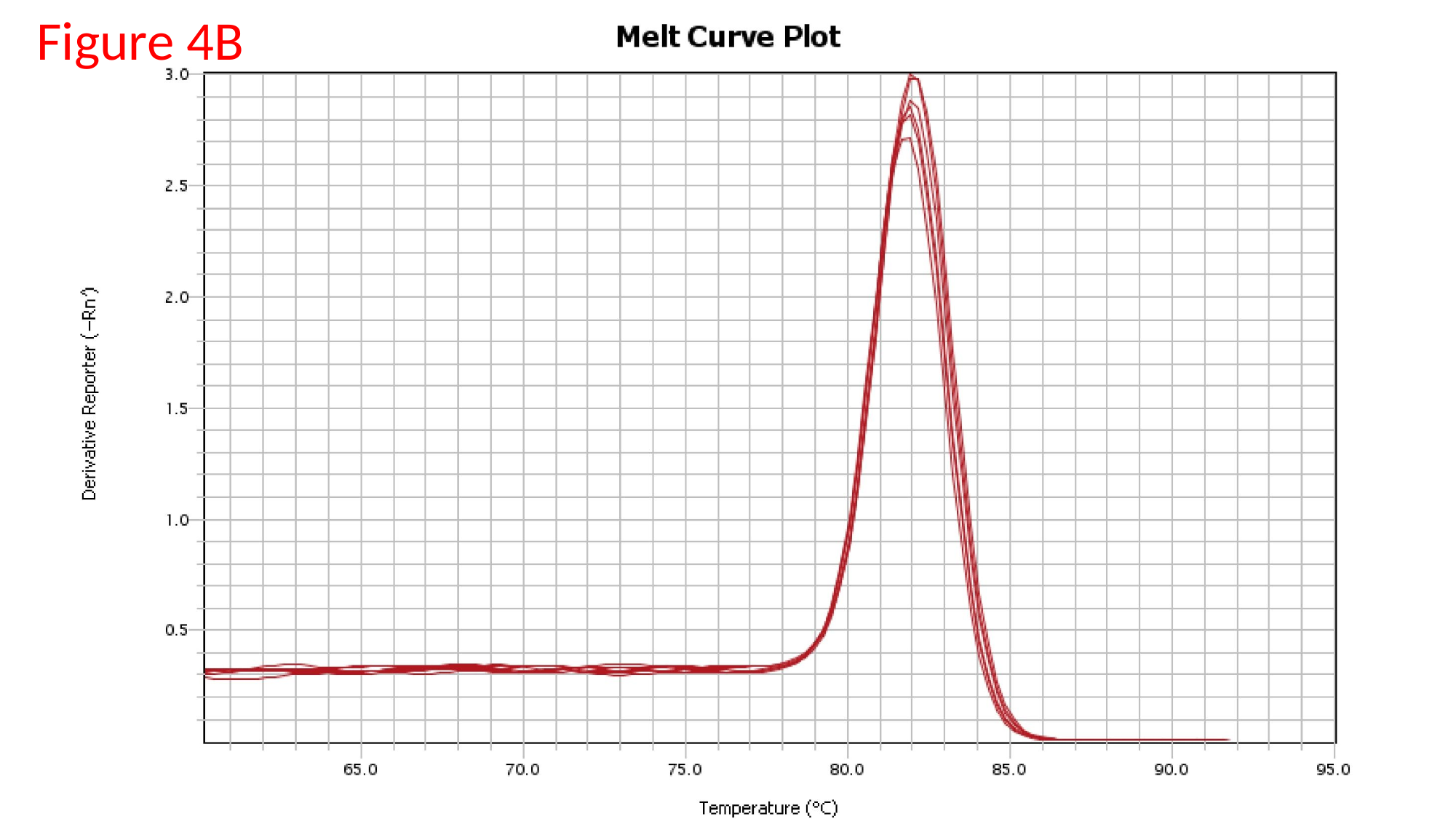

Figure 4B

## Slide 28
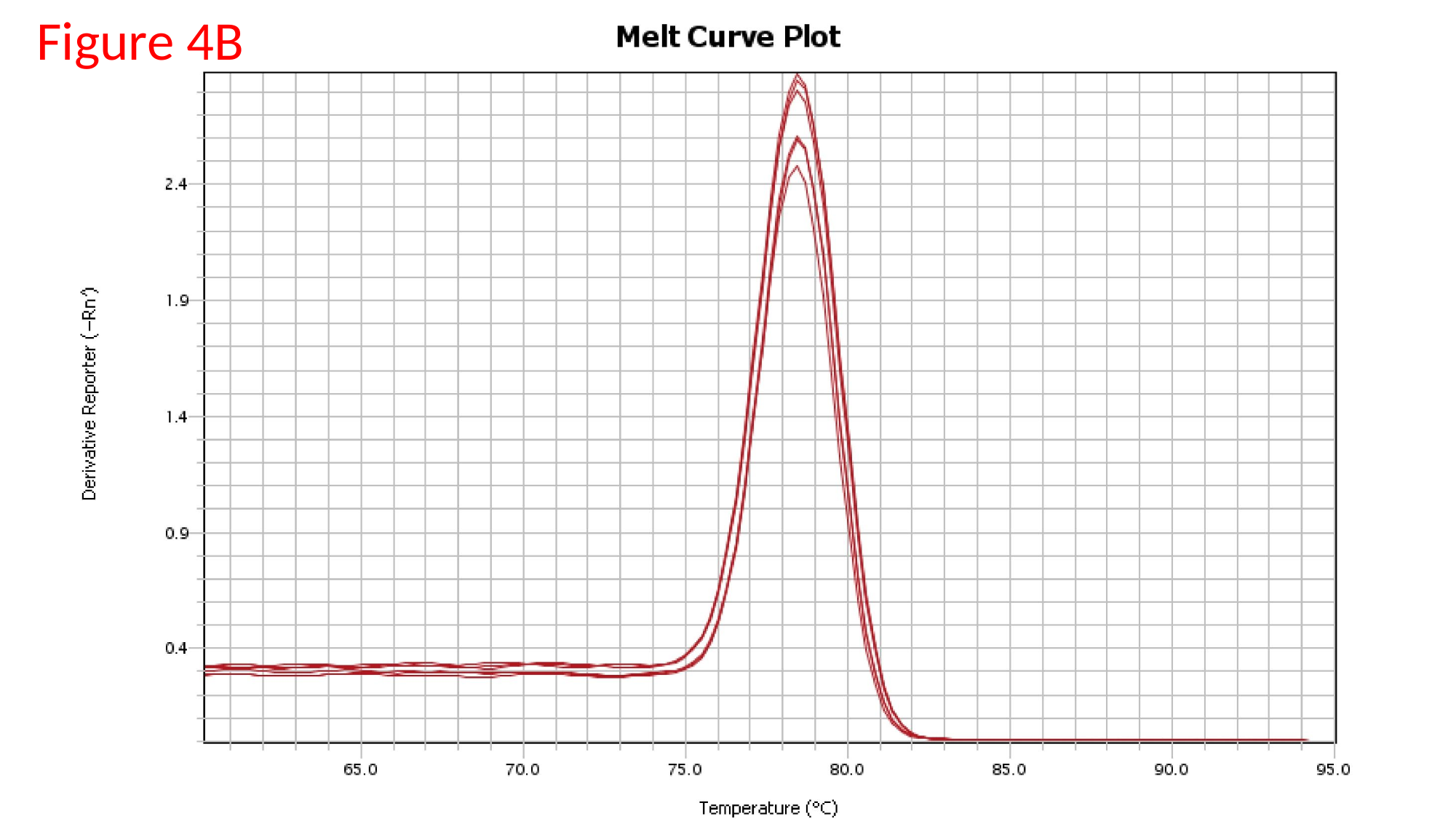

Figure 4B

## Slide 29
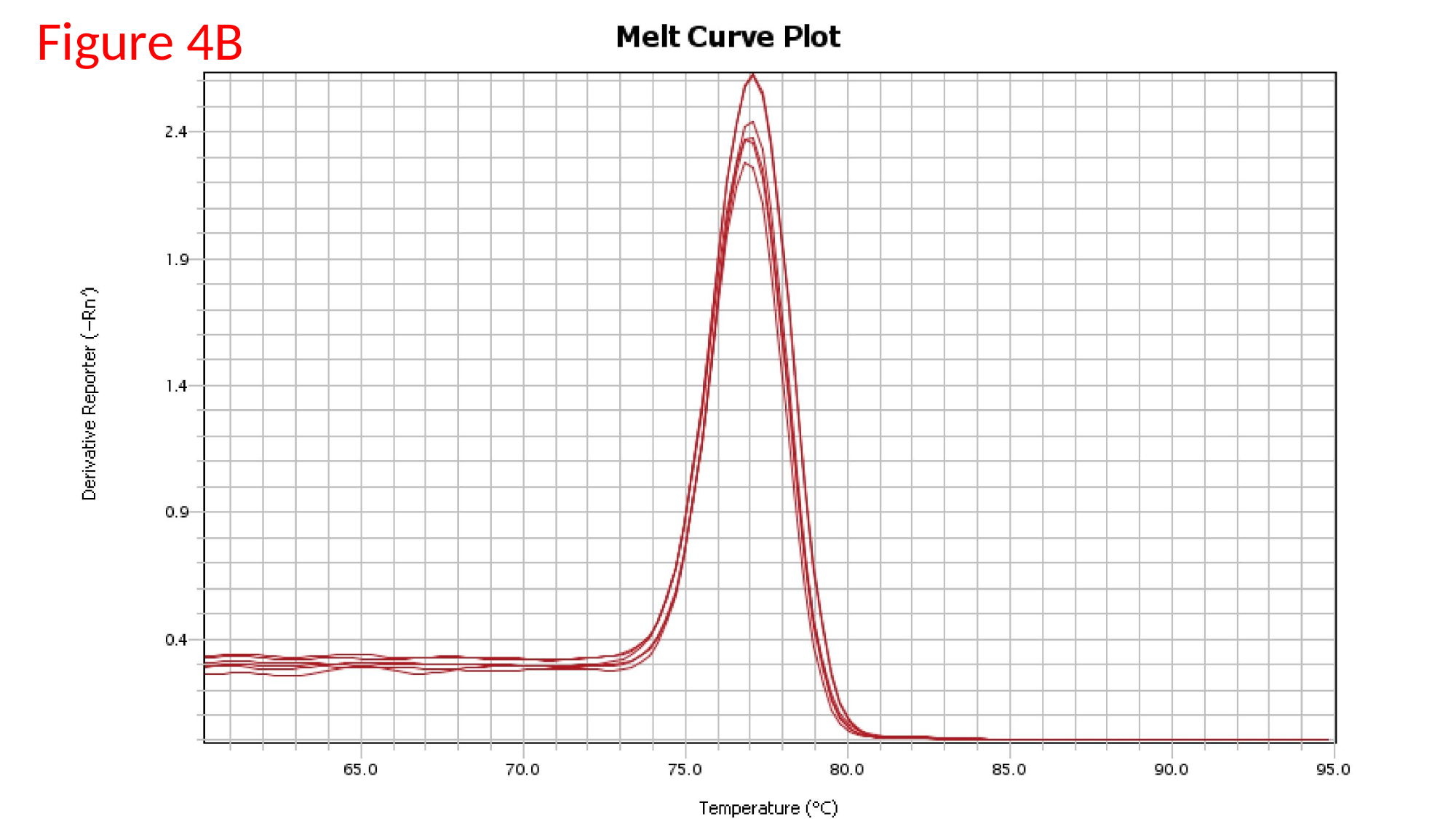

Figure 4B

## Slide 30
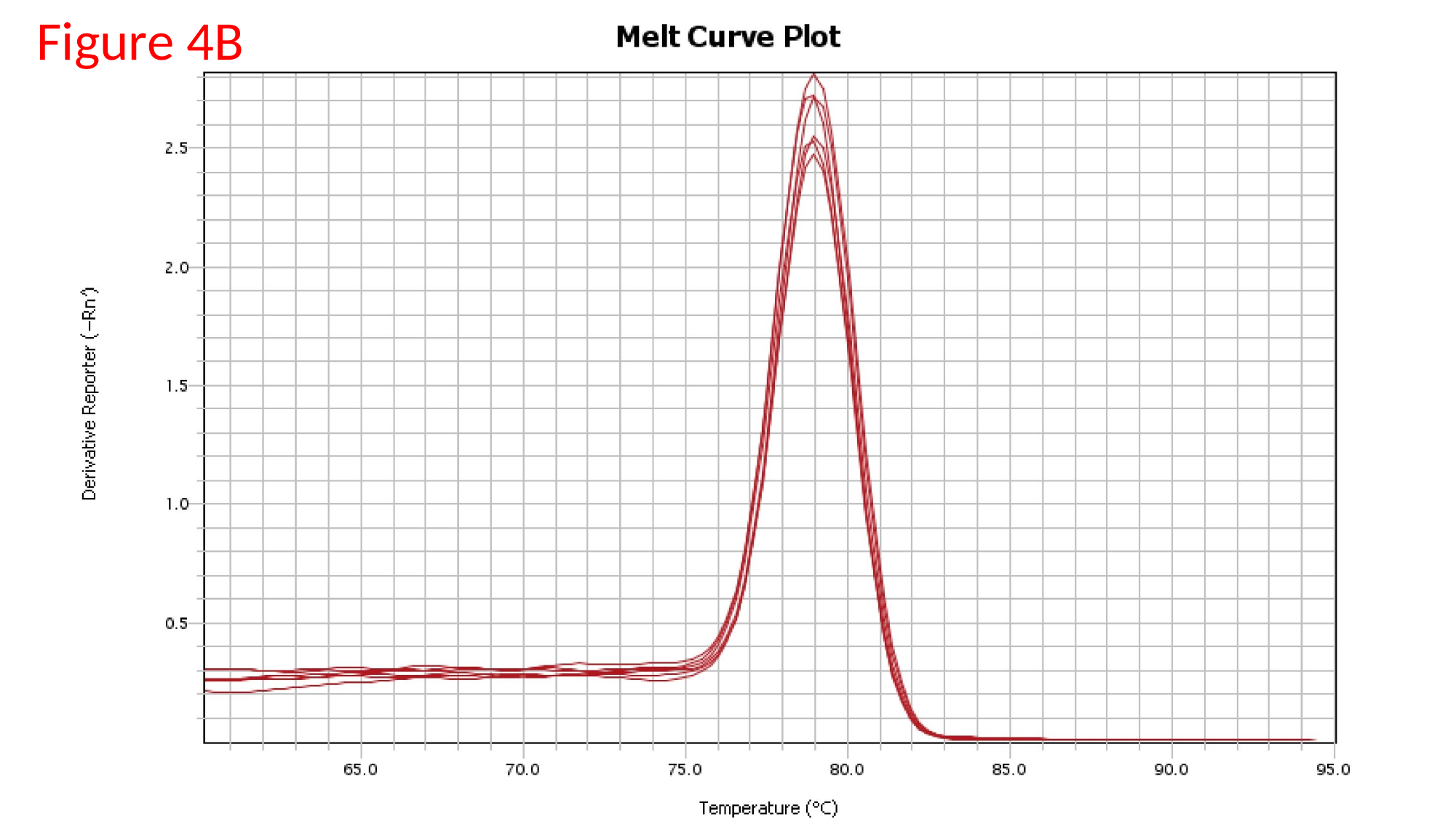

Figure 4B

## Slide 31
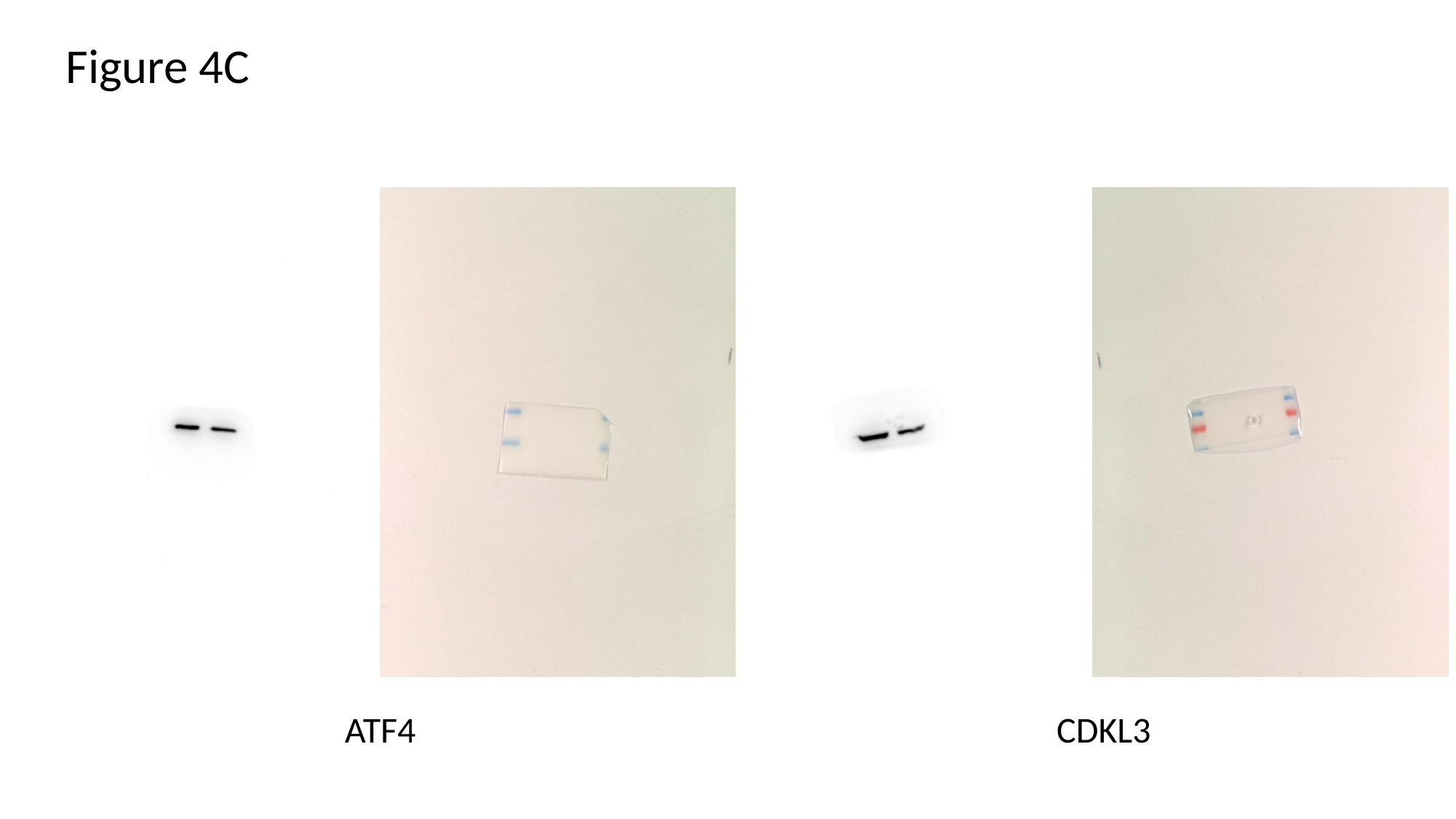

Figure 4C
ATF4
CDKL3

## Slide 32
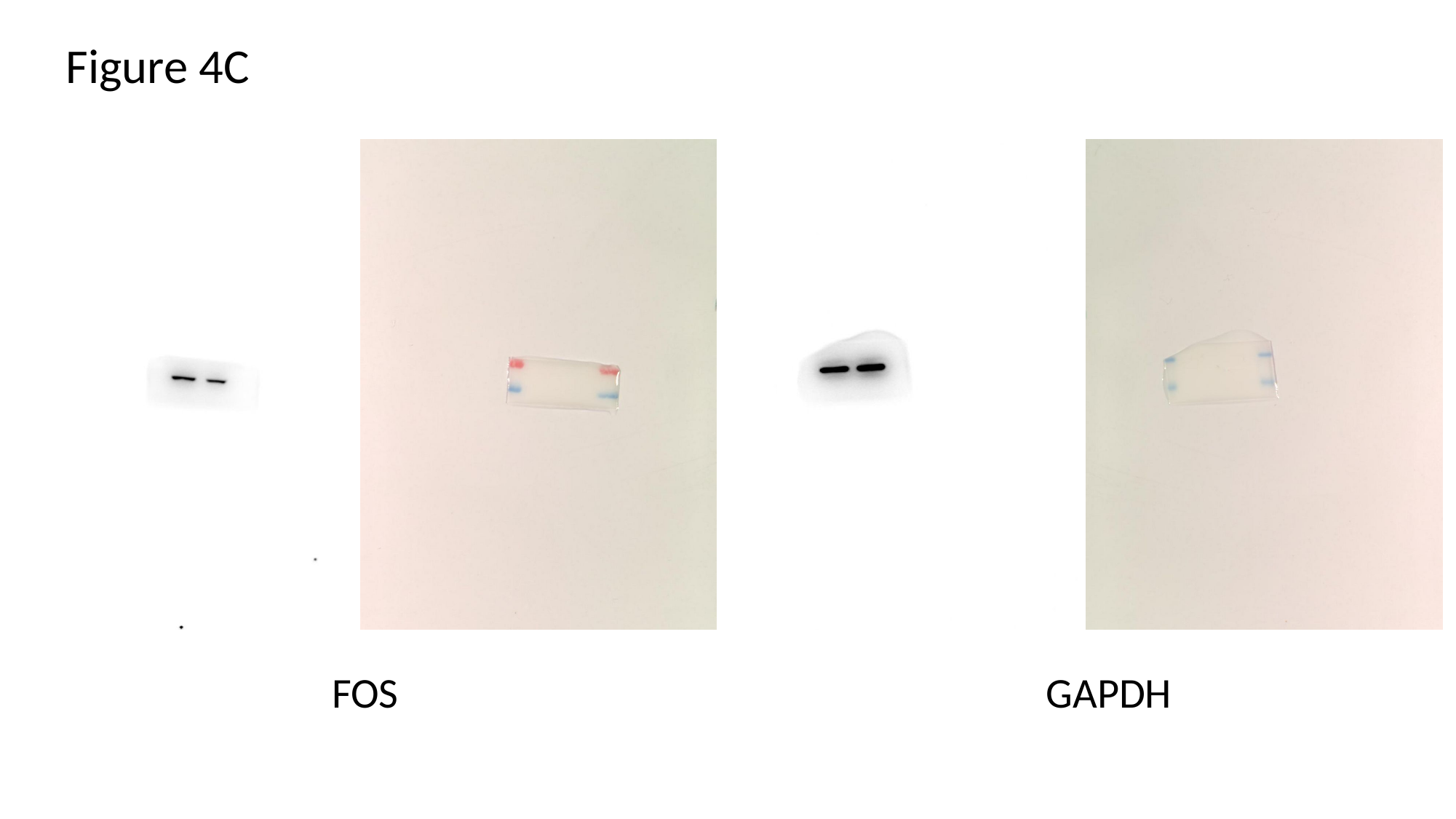

Figure 4C
FOS
GAPDH

## Slide 33
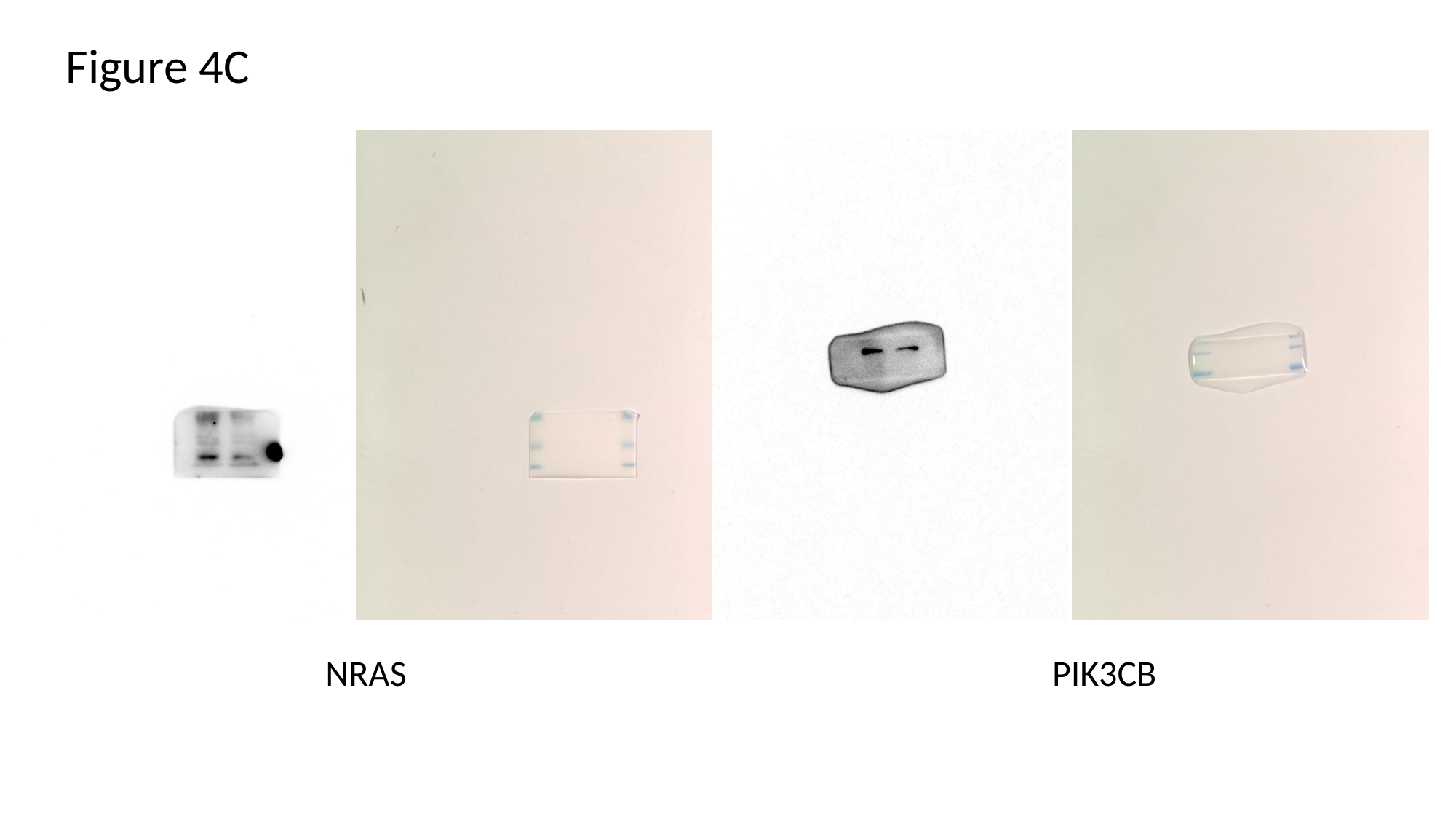

Figure 4C
NRAS
PIK3CB

## Slide 34
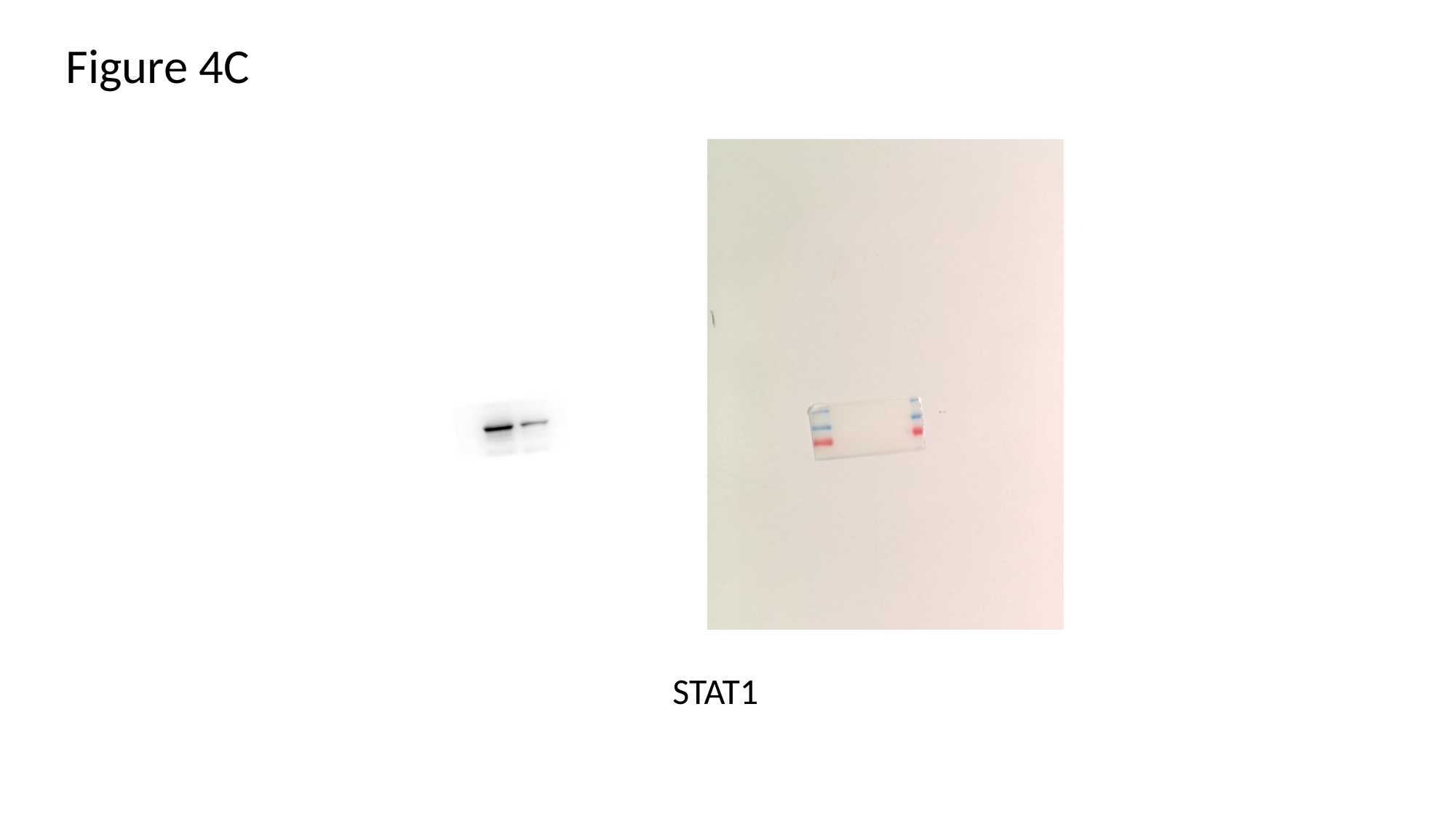

Figure 4C
STAT1

## Slide 35
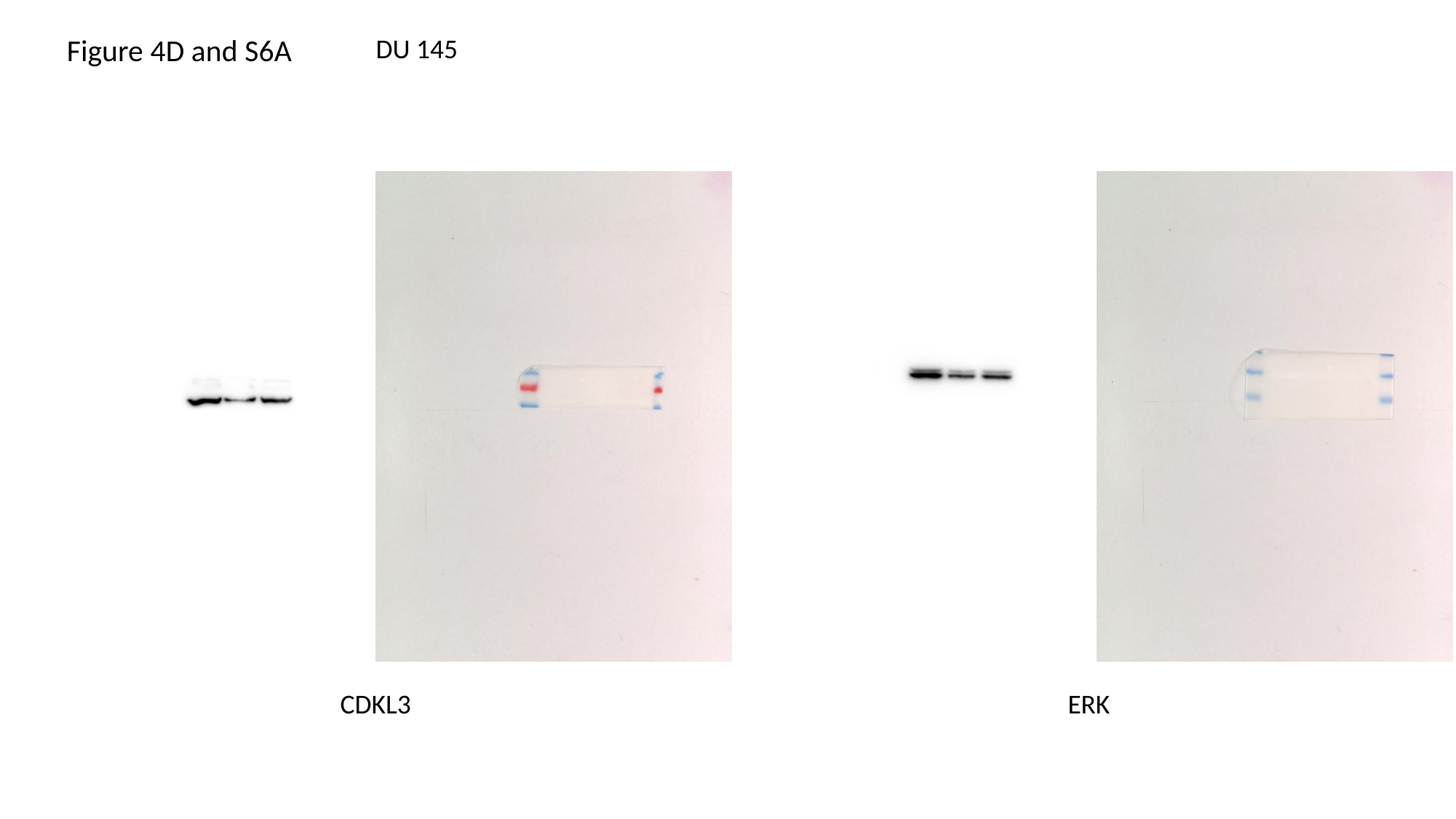

Figure 4D and S6A
DU 145
CDKL3
ERK

## Slide 36
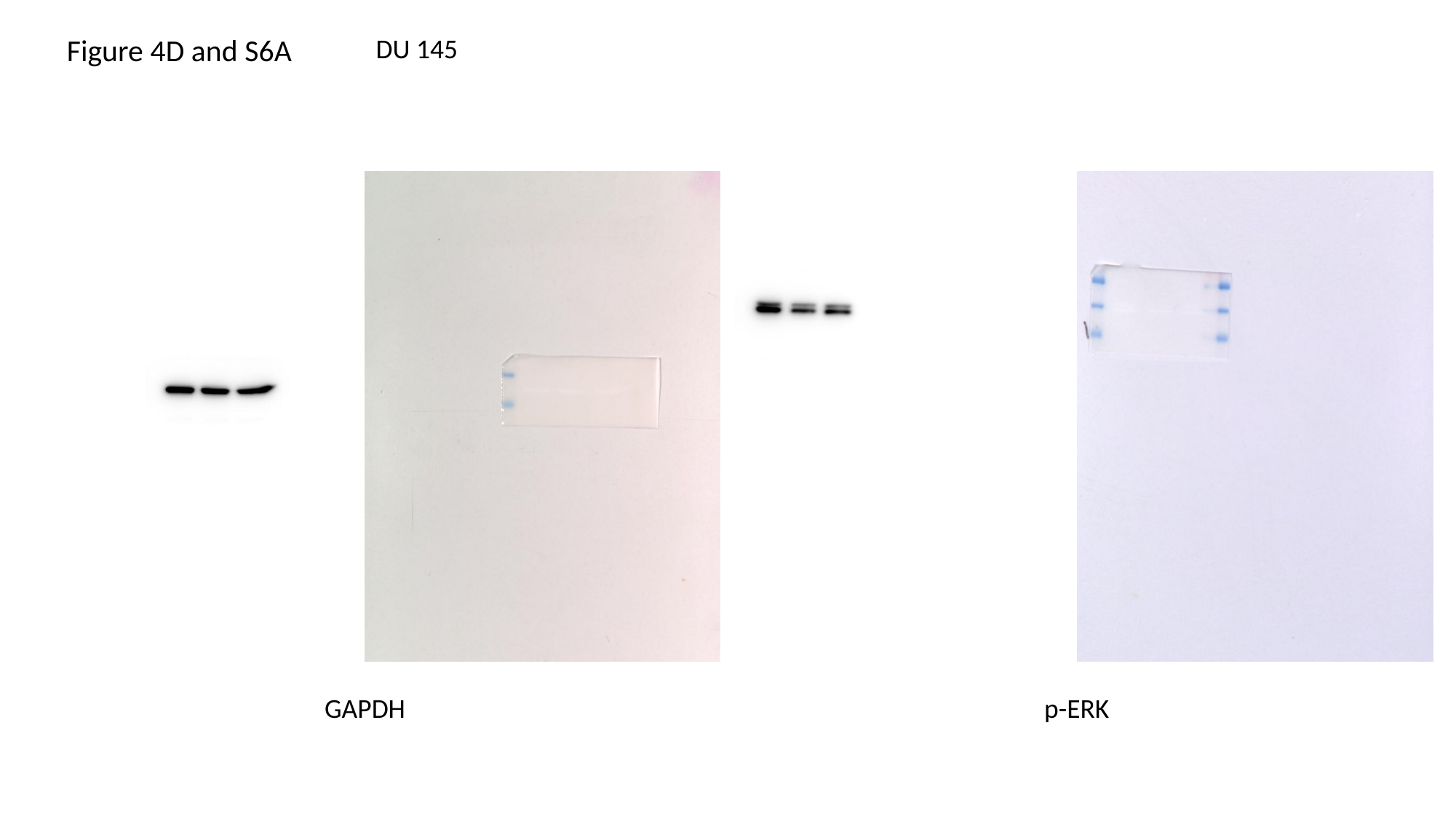

Figure 4D and S6A
DU 145
GAPDH
p-ERK

## Slide 37
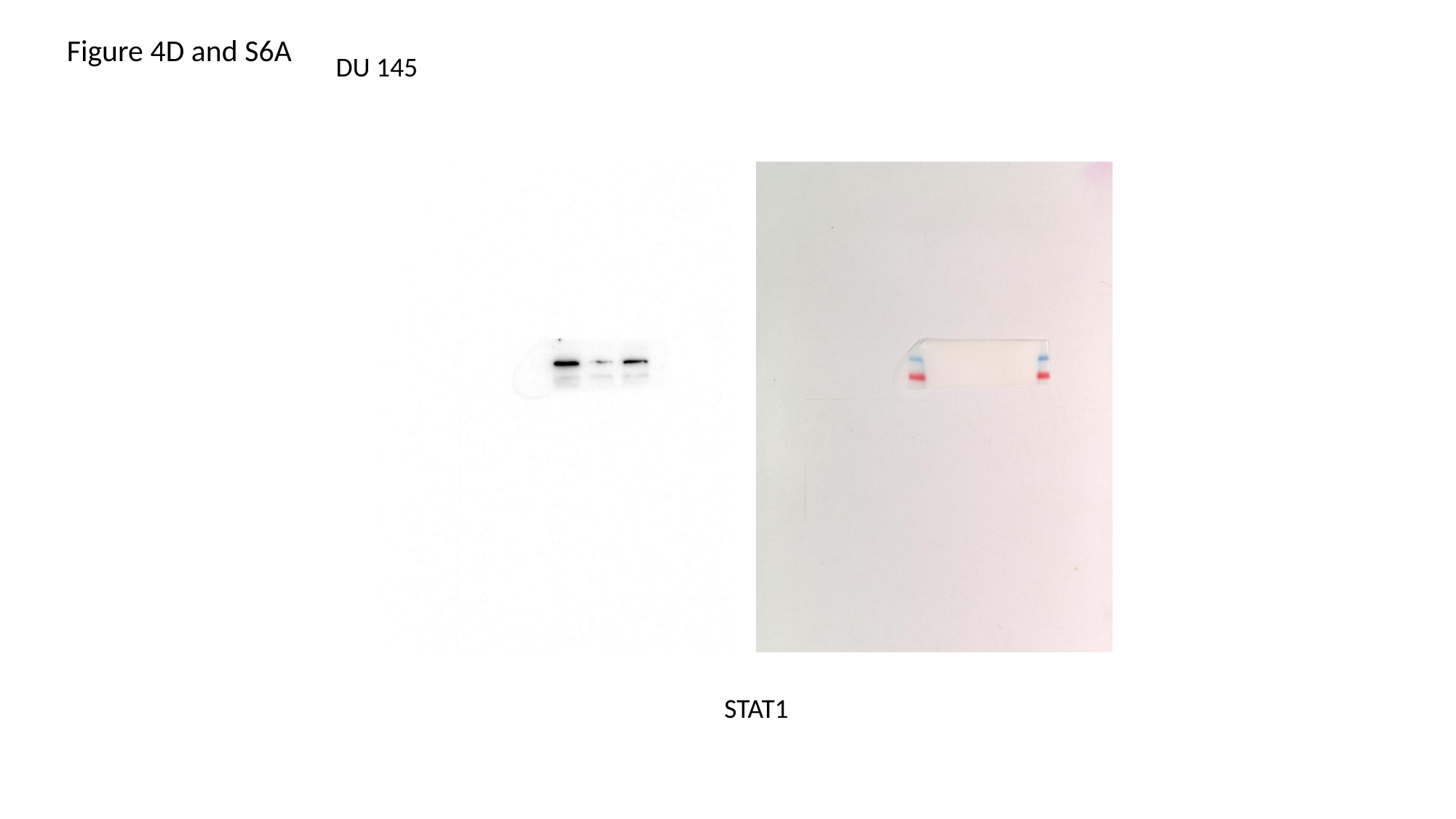

Figure 4D and S6A
DU 145
STAT1

## Slide 38
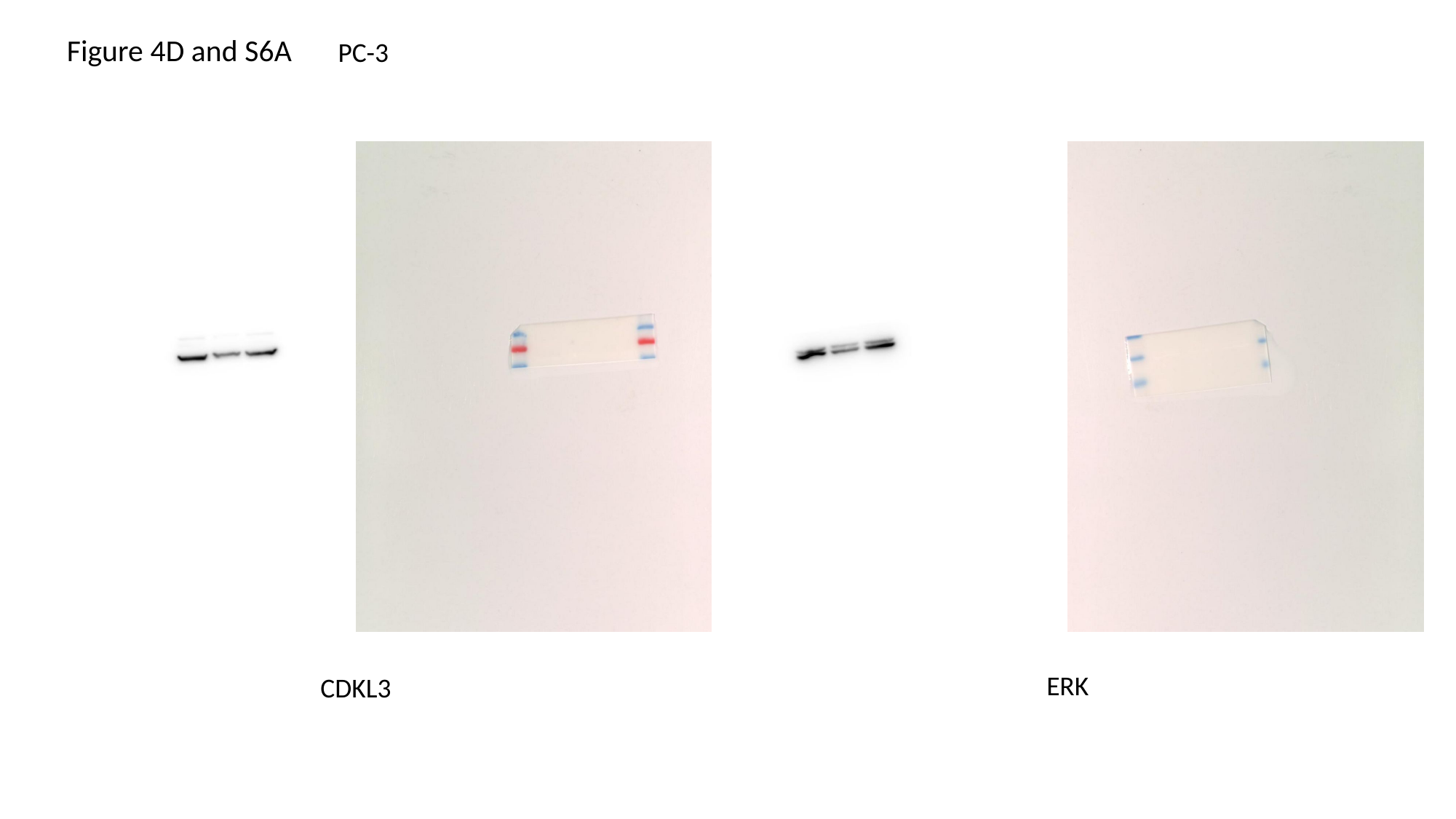

Figure 4D and S6A
PC-3
ERK
CDKL3

## Slide 39
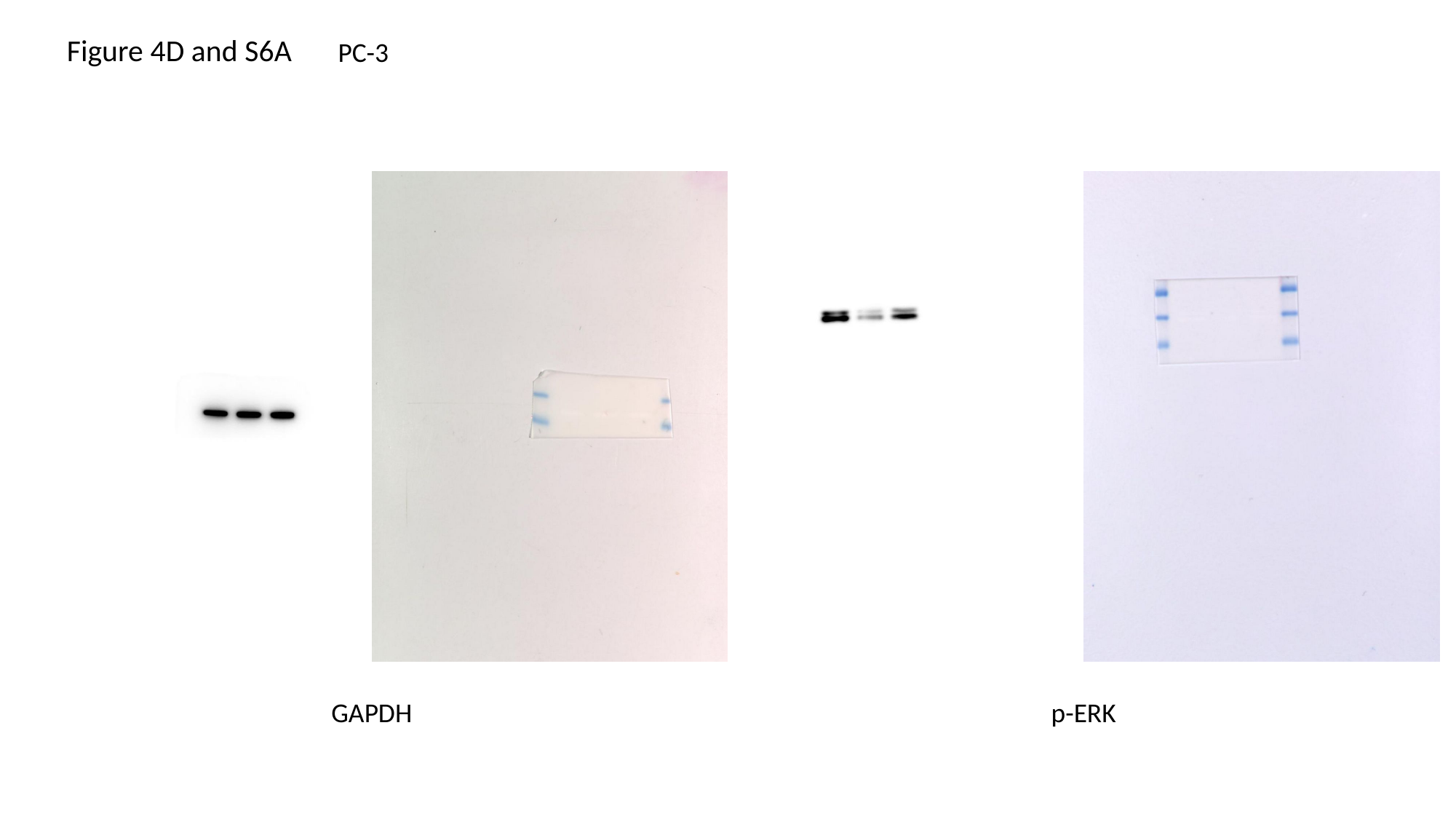

Figure 4D and S6A
PC-3
GAPDH
p-ERK

## Slide 40
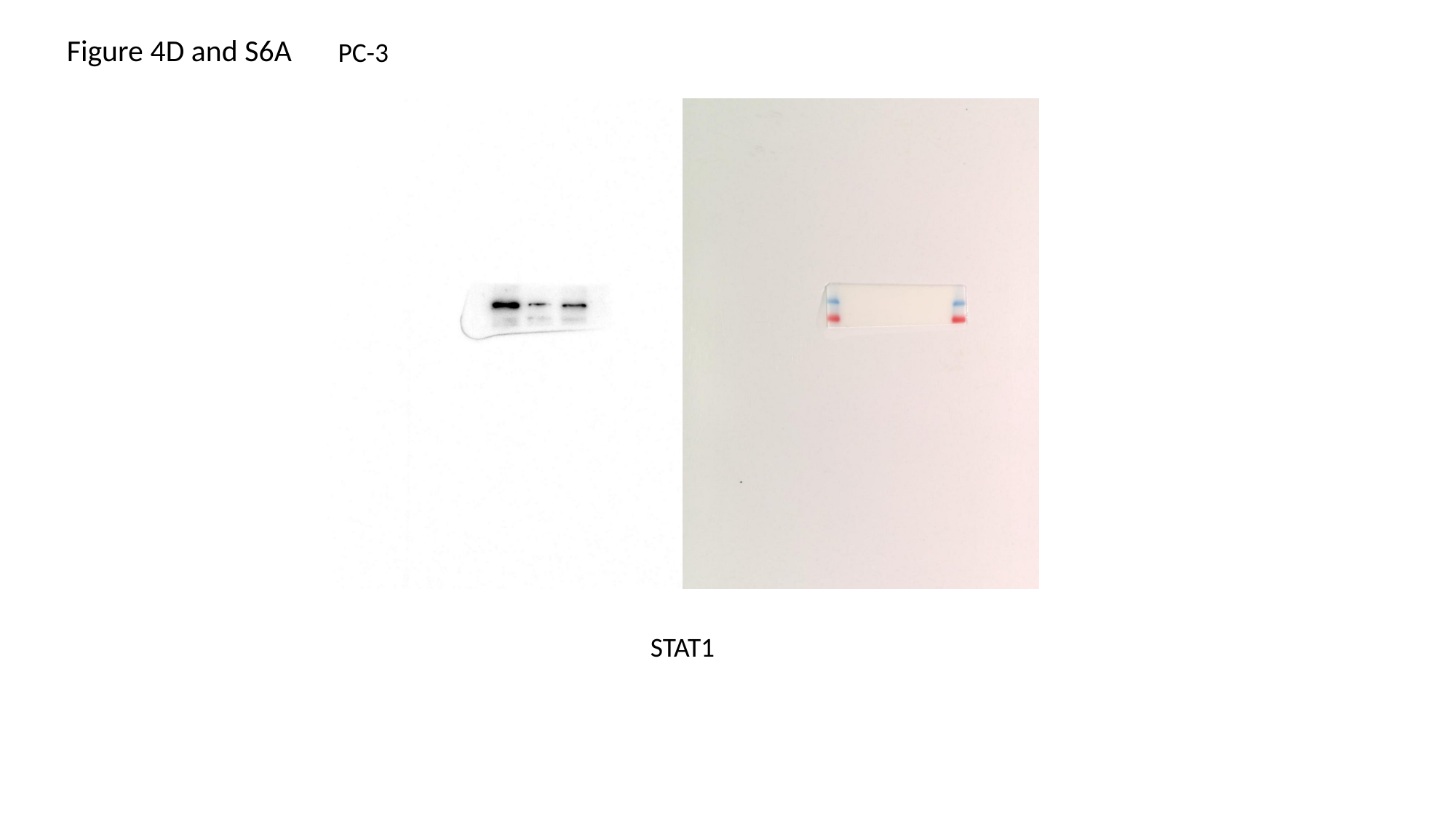

Figure 4D and S6A
PC-3
STAT1

## Slide 41
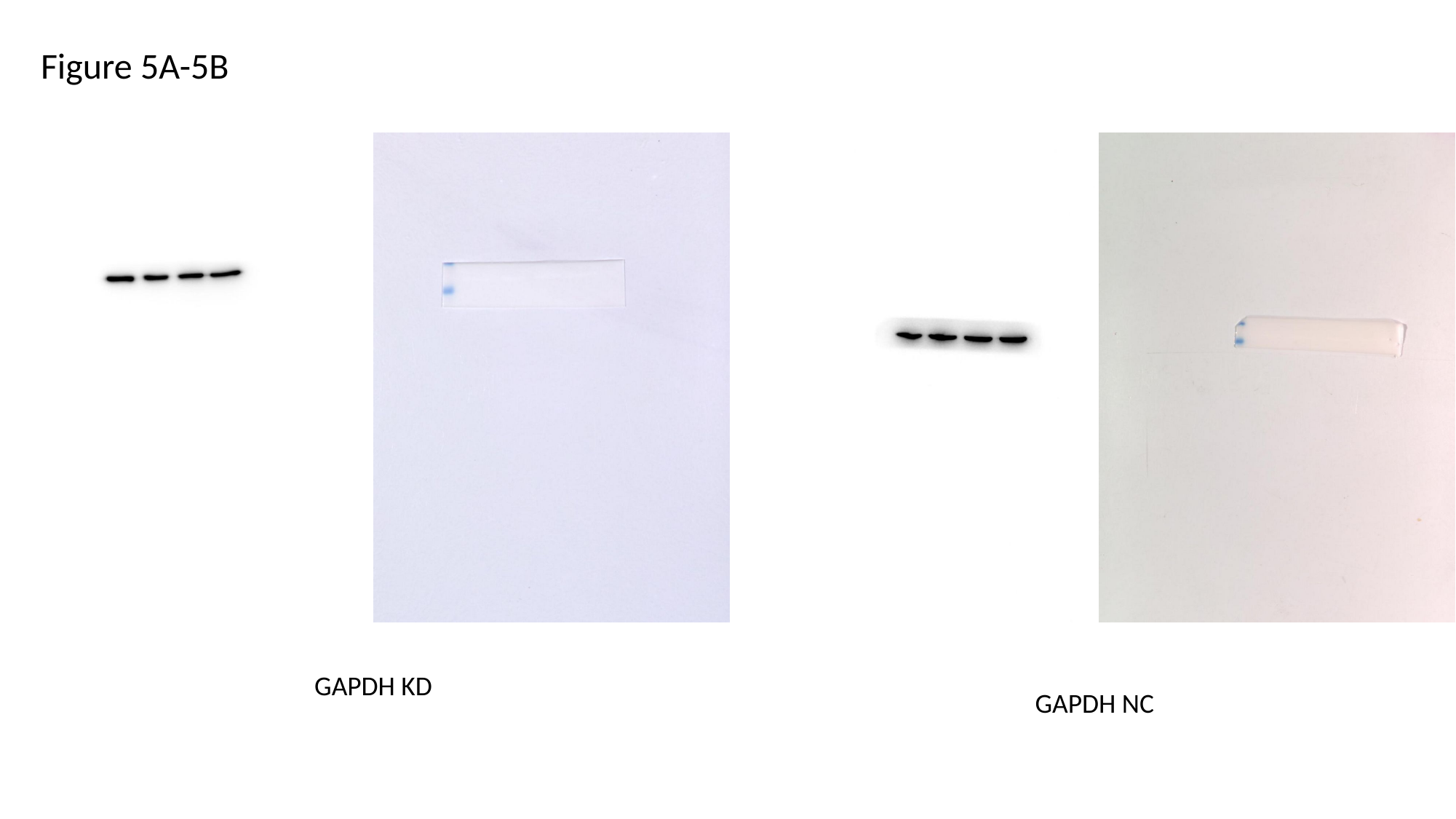

Figure 5A-5B
GAPDH KD
GAPDH NC

## Slide 42
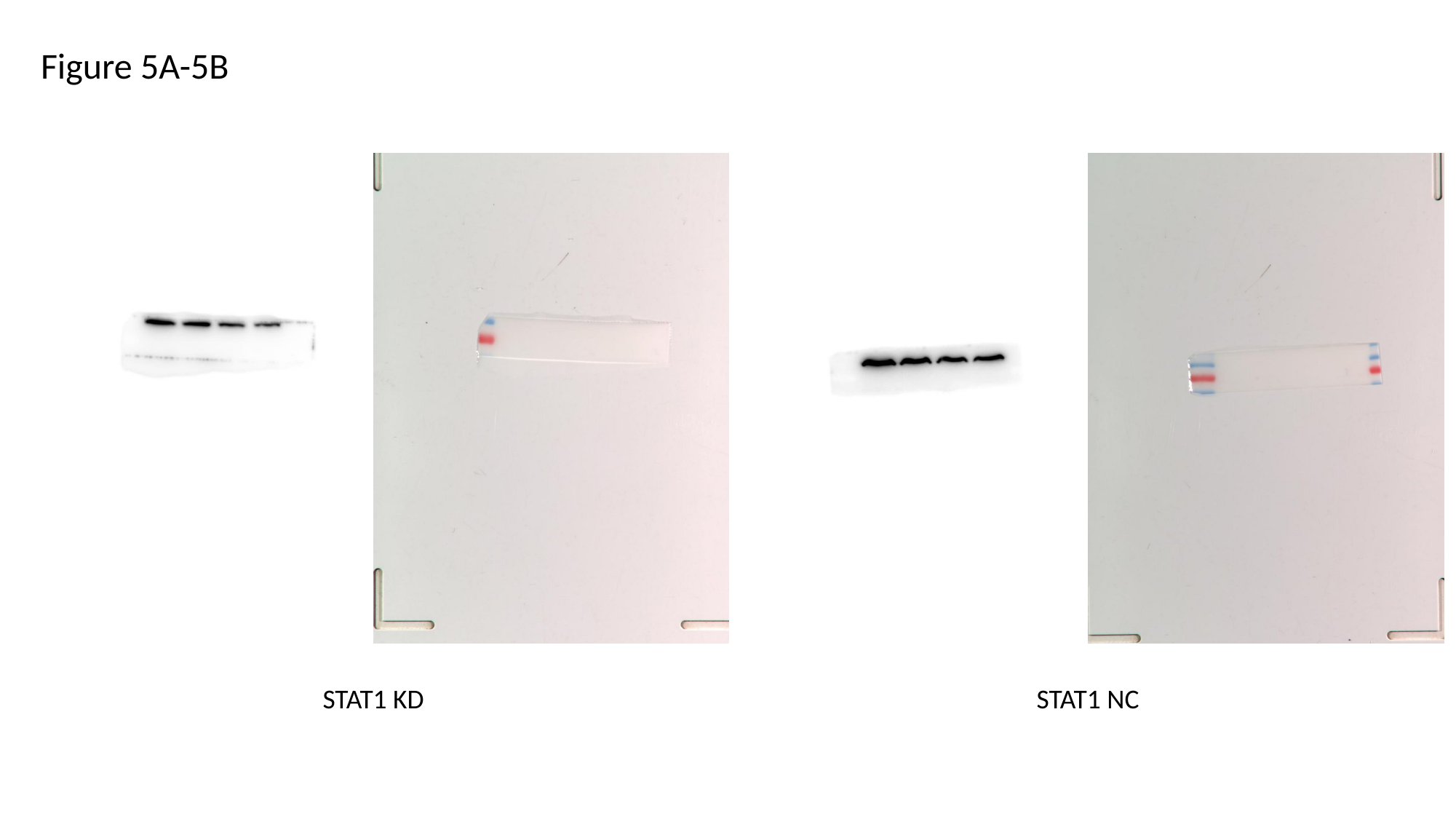

Figure 5A-5B
STAT1 KD
STAT1 NC

## Slide 43
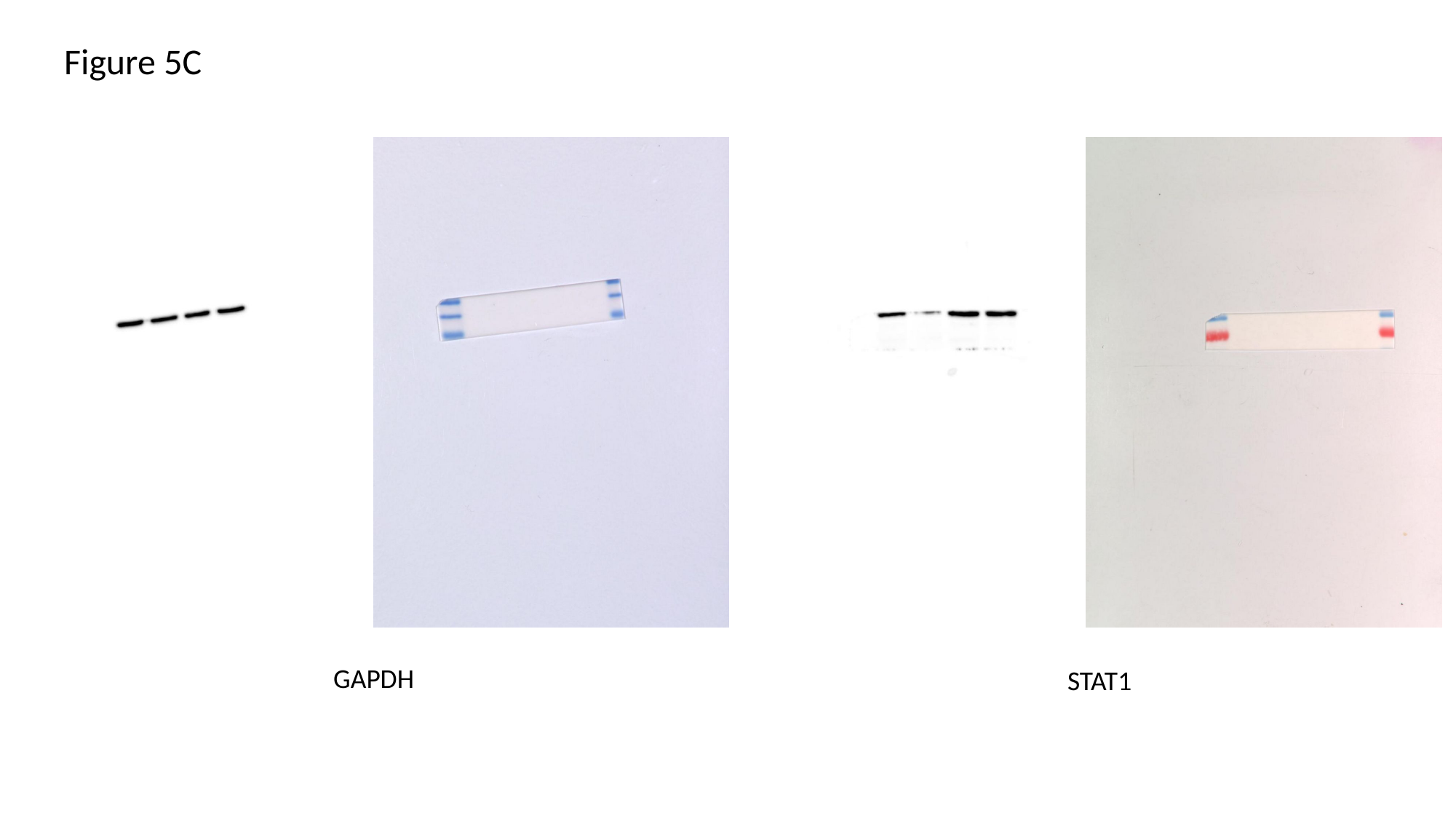

Figure 5C
GAPDH
STAT1

## Slide 44
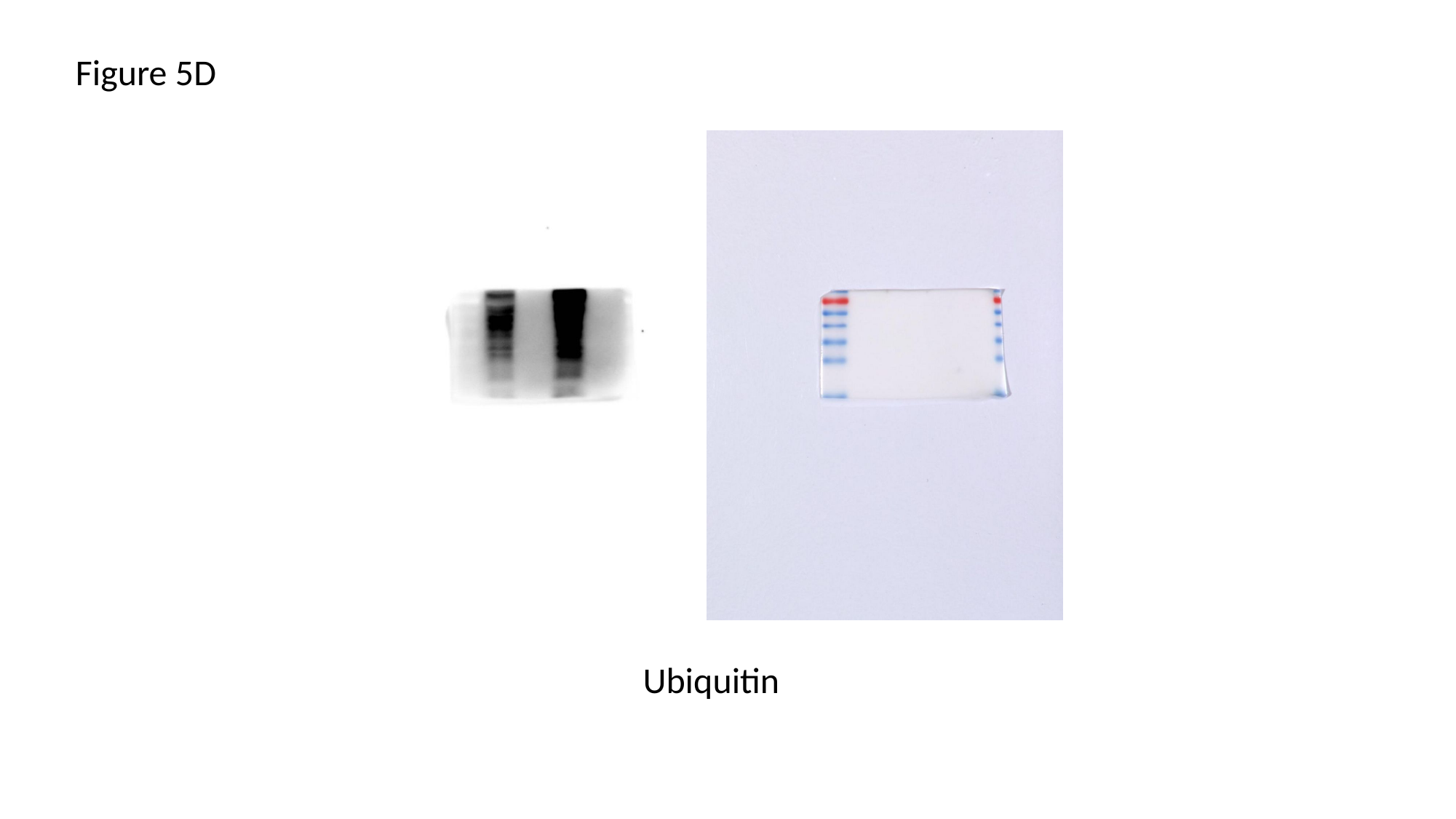

Figure 5D
Ubiquitin

## Slide 45
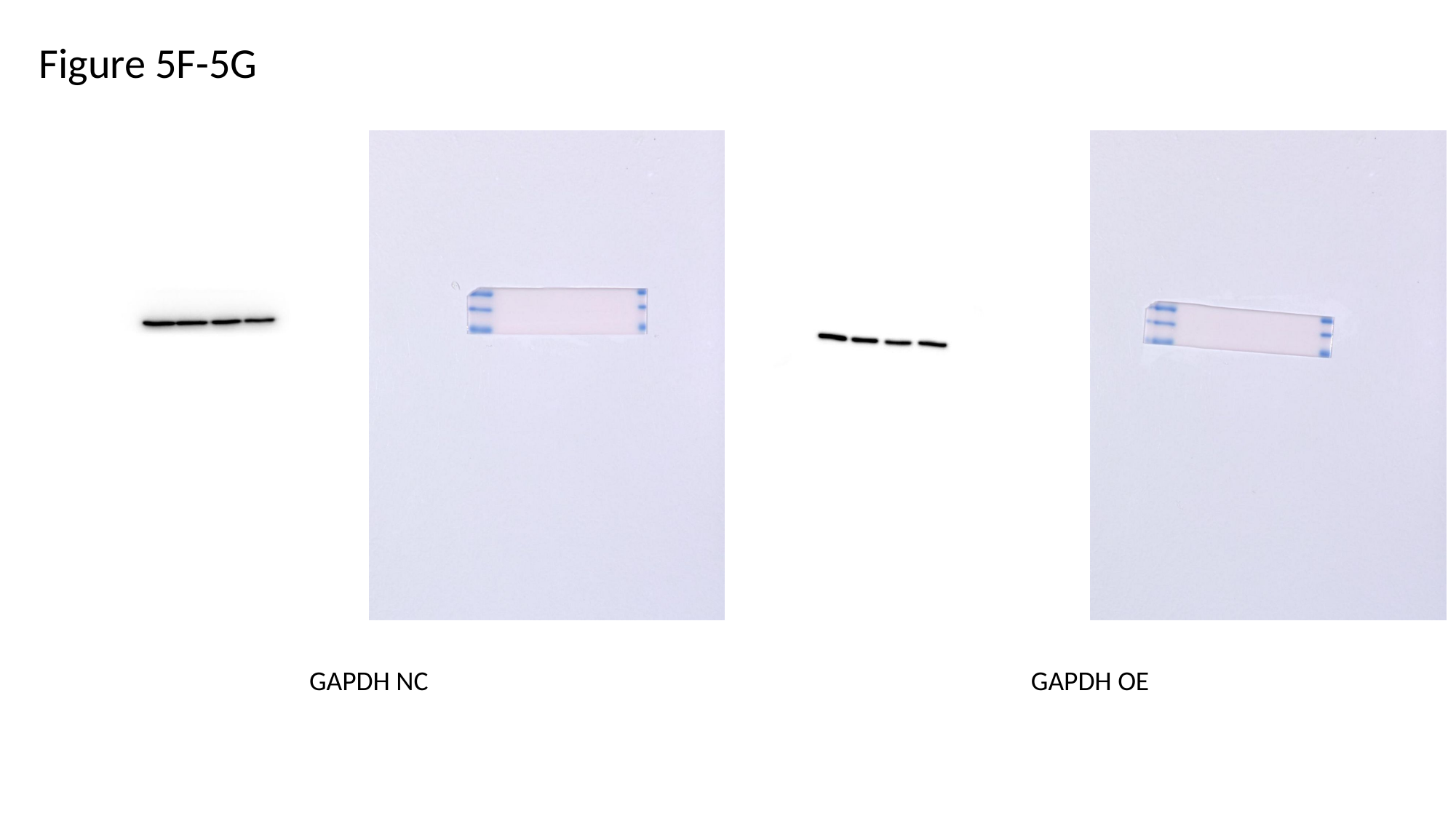

Figure 5F-5G
GAPDH NC
GAPDH OE

## Slide 46
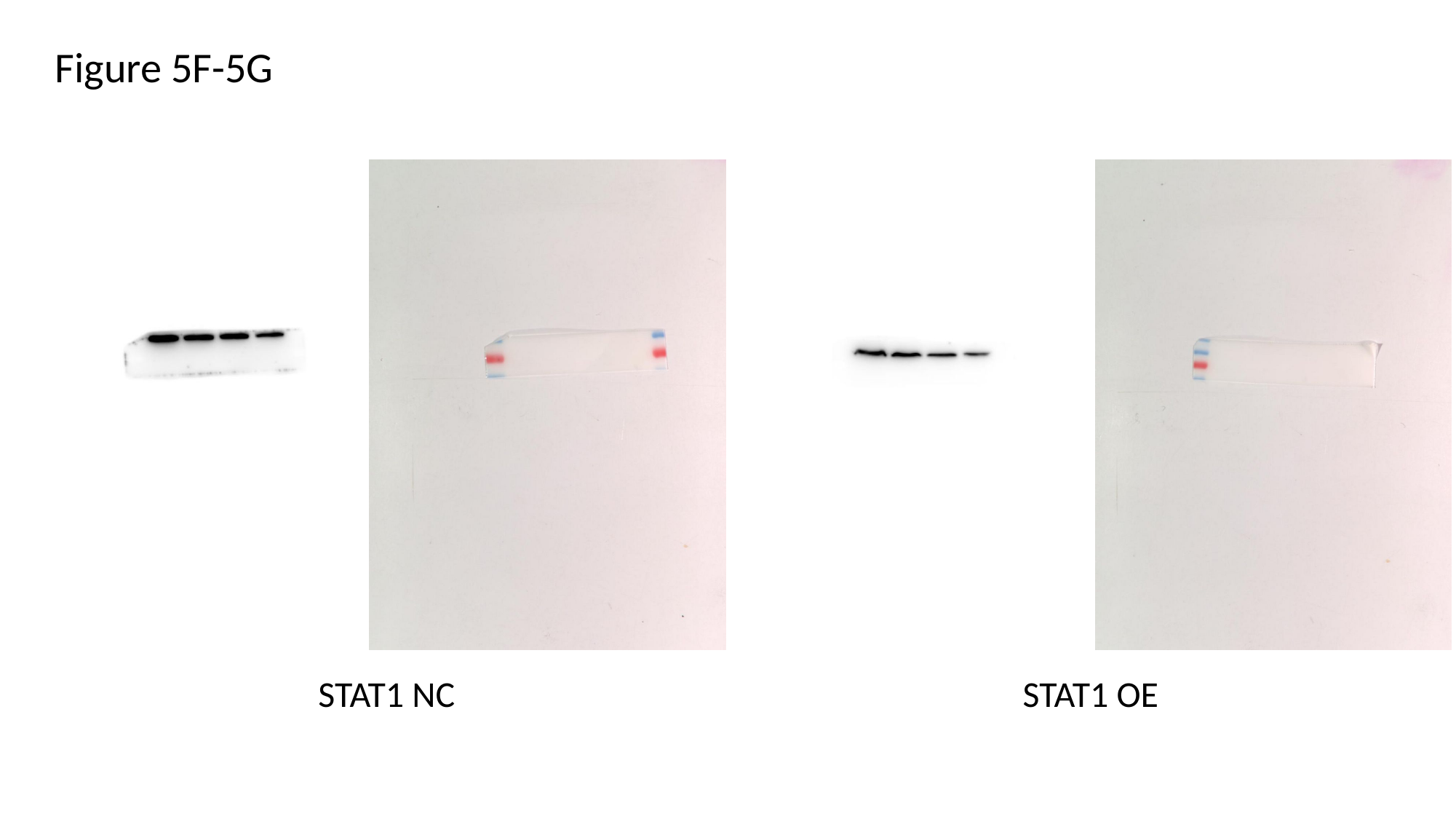

Figure 5F-5G
STAT1 OE
STAT1 NC

## Slide 47
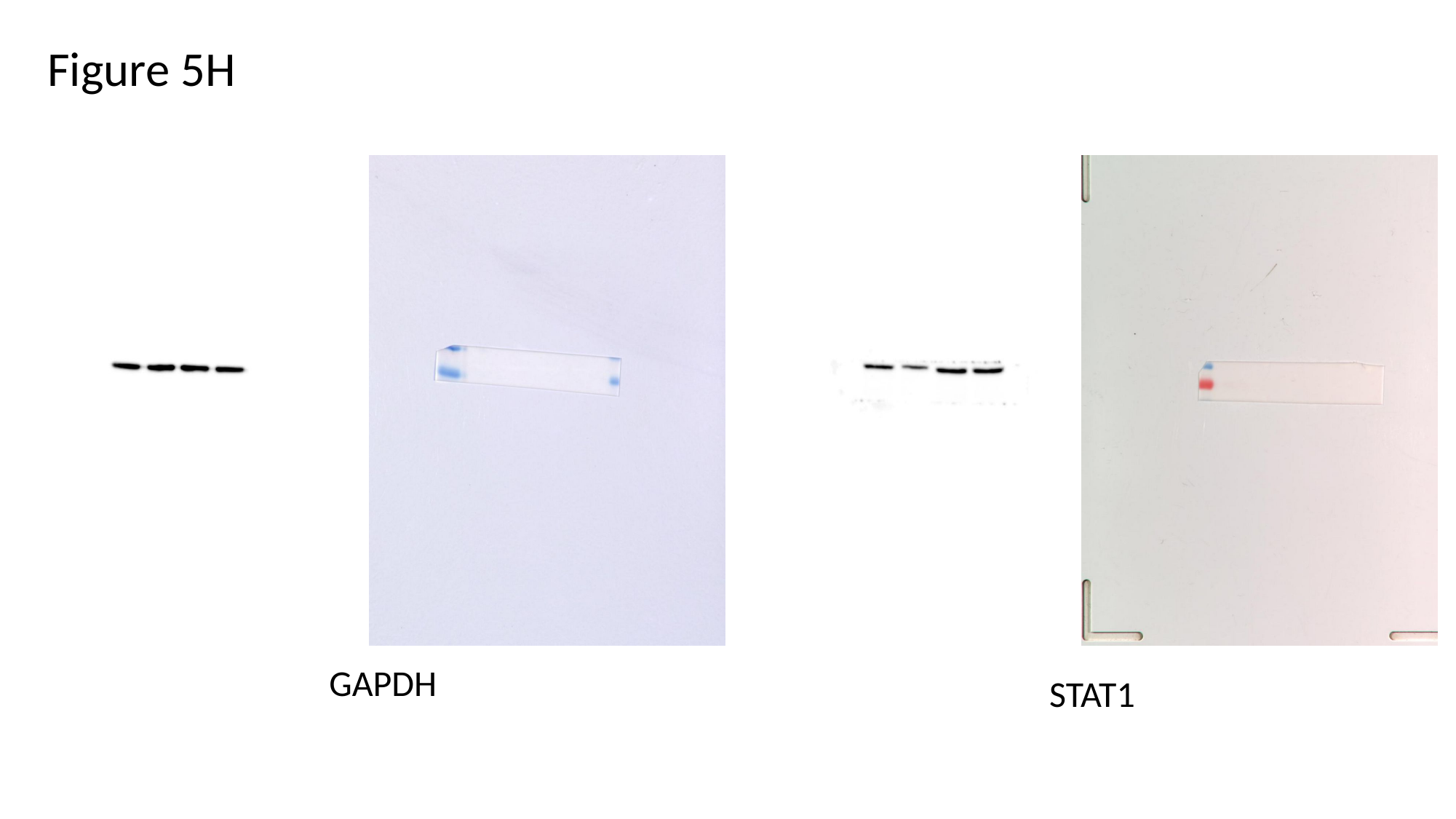

Figure 5H
GAPDH
STAT1

## Slide 48
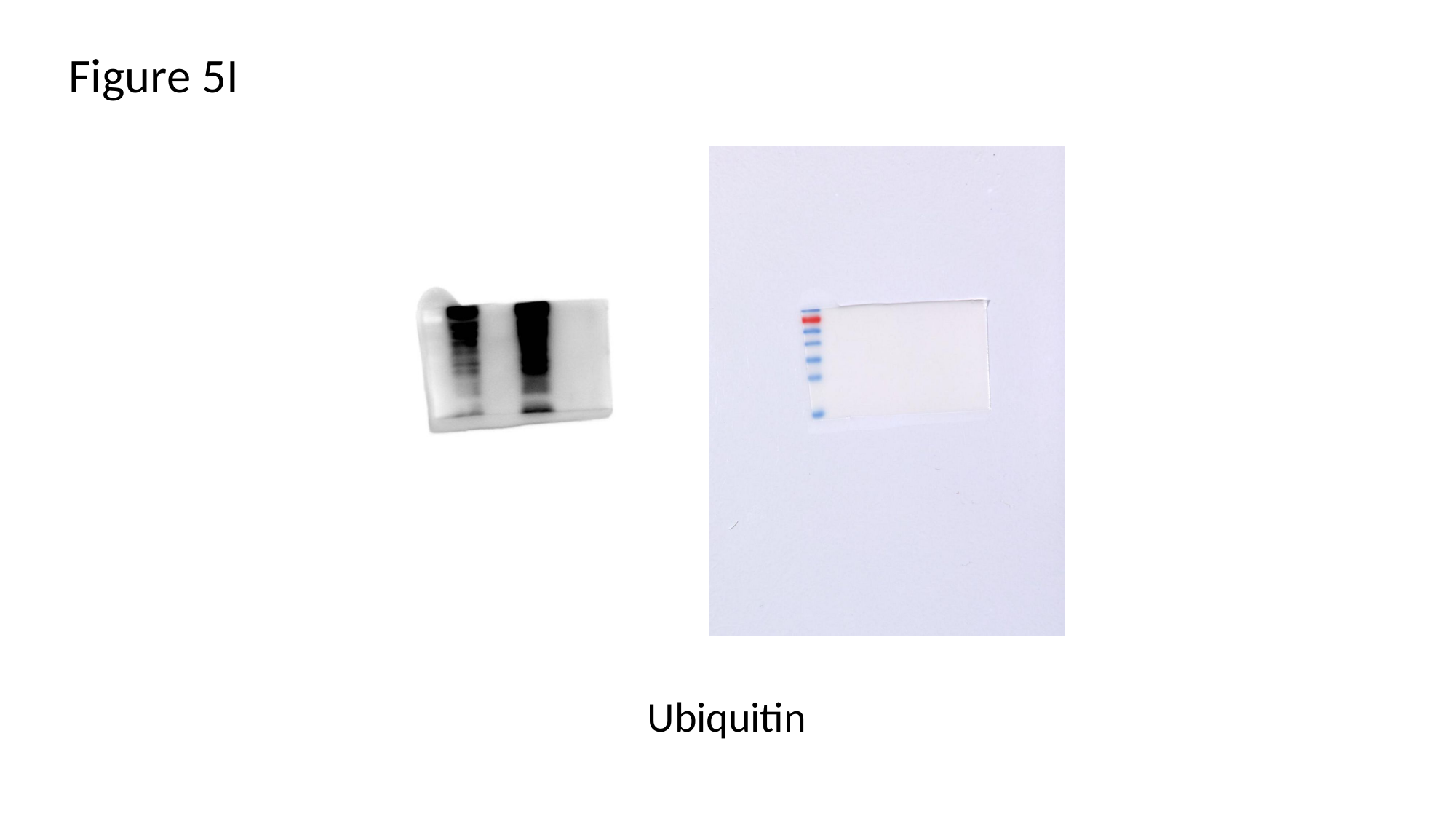

Figure 5I
Ubiquitin

## Slide 49
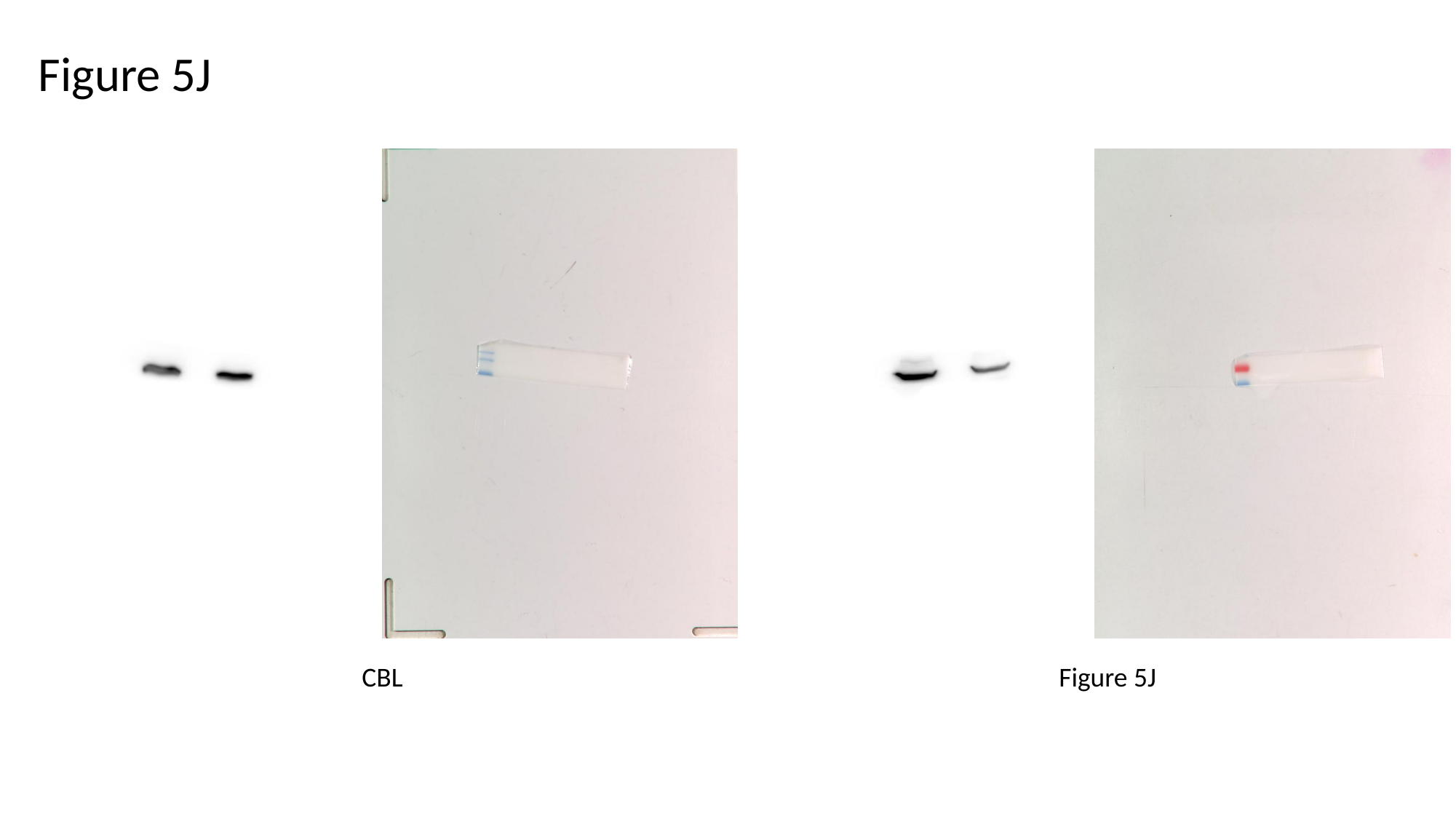

Figure 5J
CBL
Figure 5J

## Slide 50
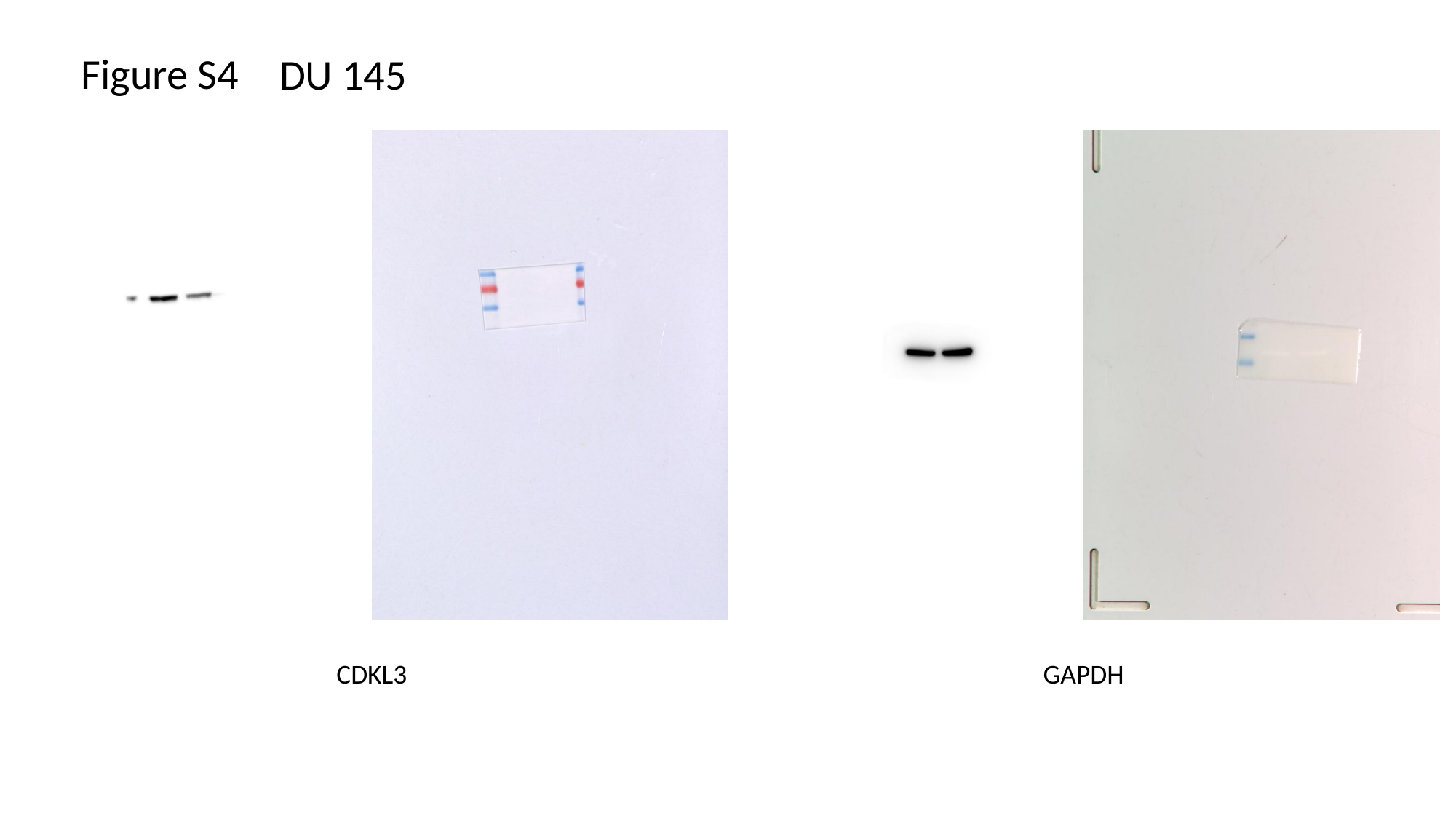

Figure S4
DU 145
CDKL3
GAPDH

## Slide 51
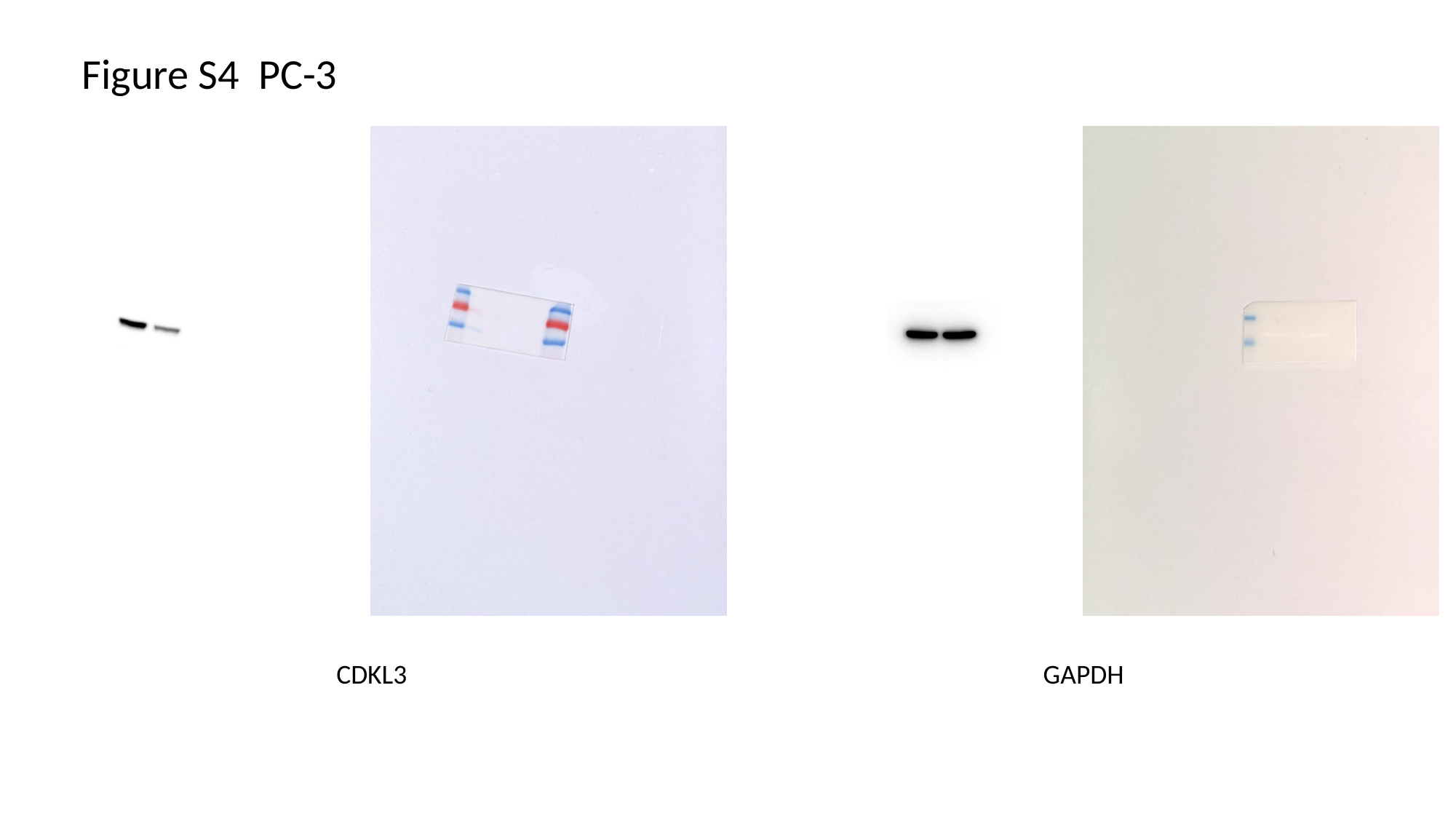

Figure S4 PC-3
CDKL3
GAPDH

## Slide 52
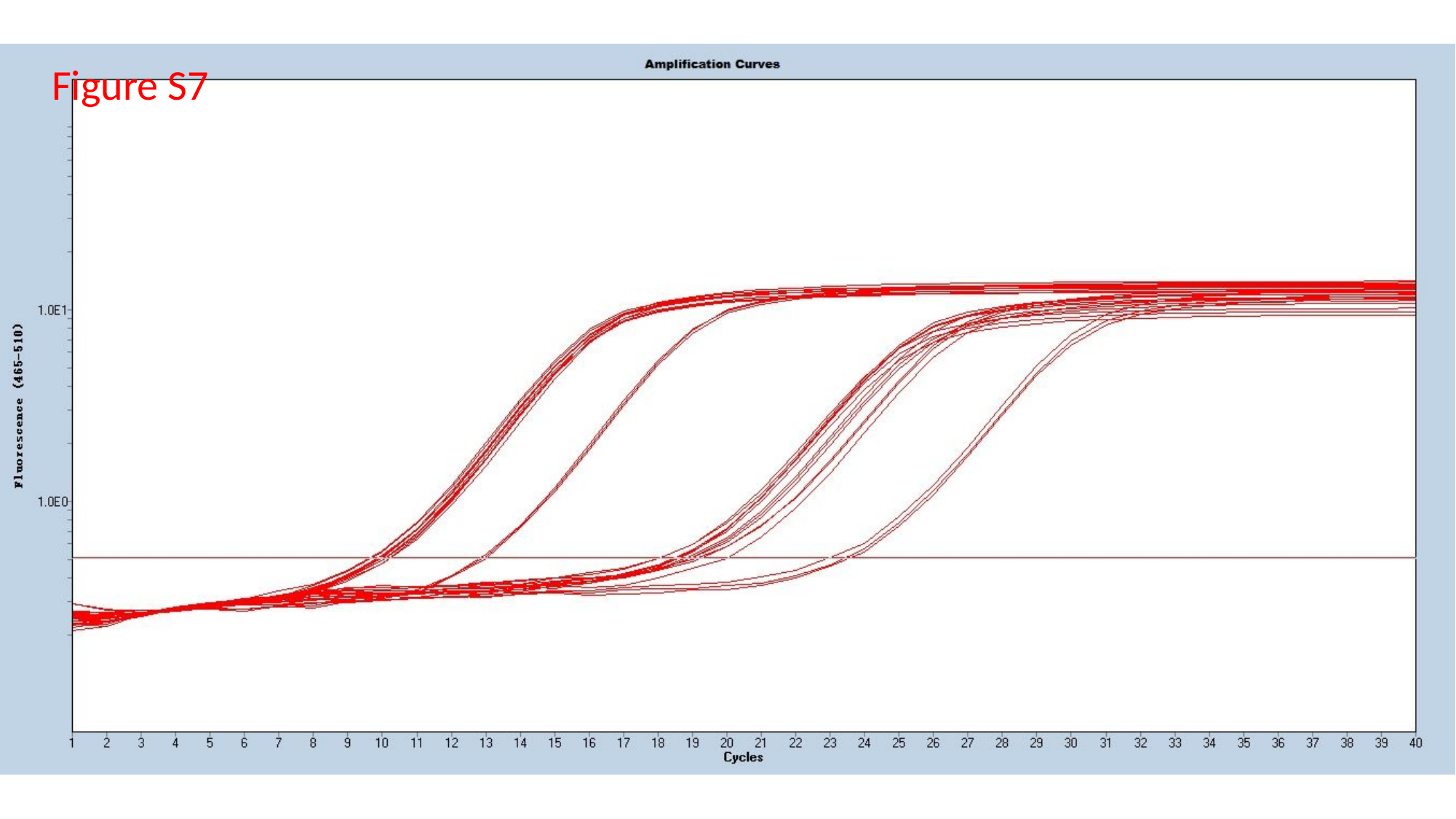

Figure S7

## Slide 53
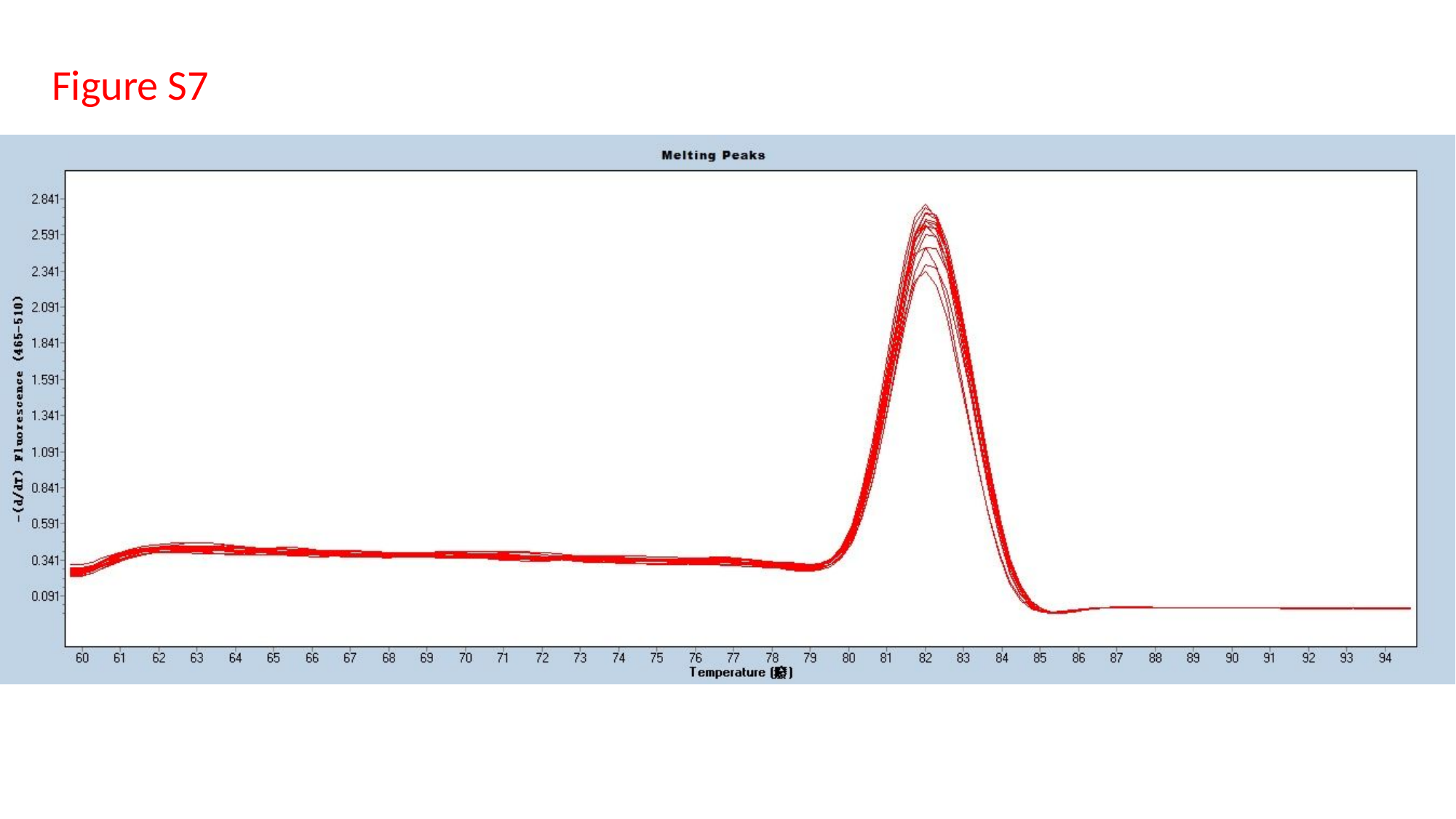

Figure S7

## Slide 54
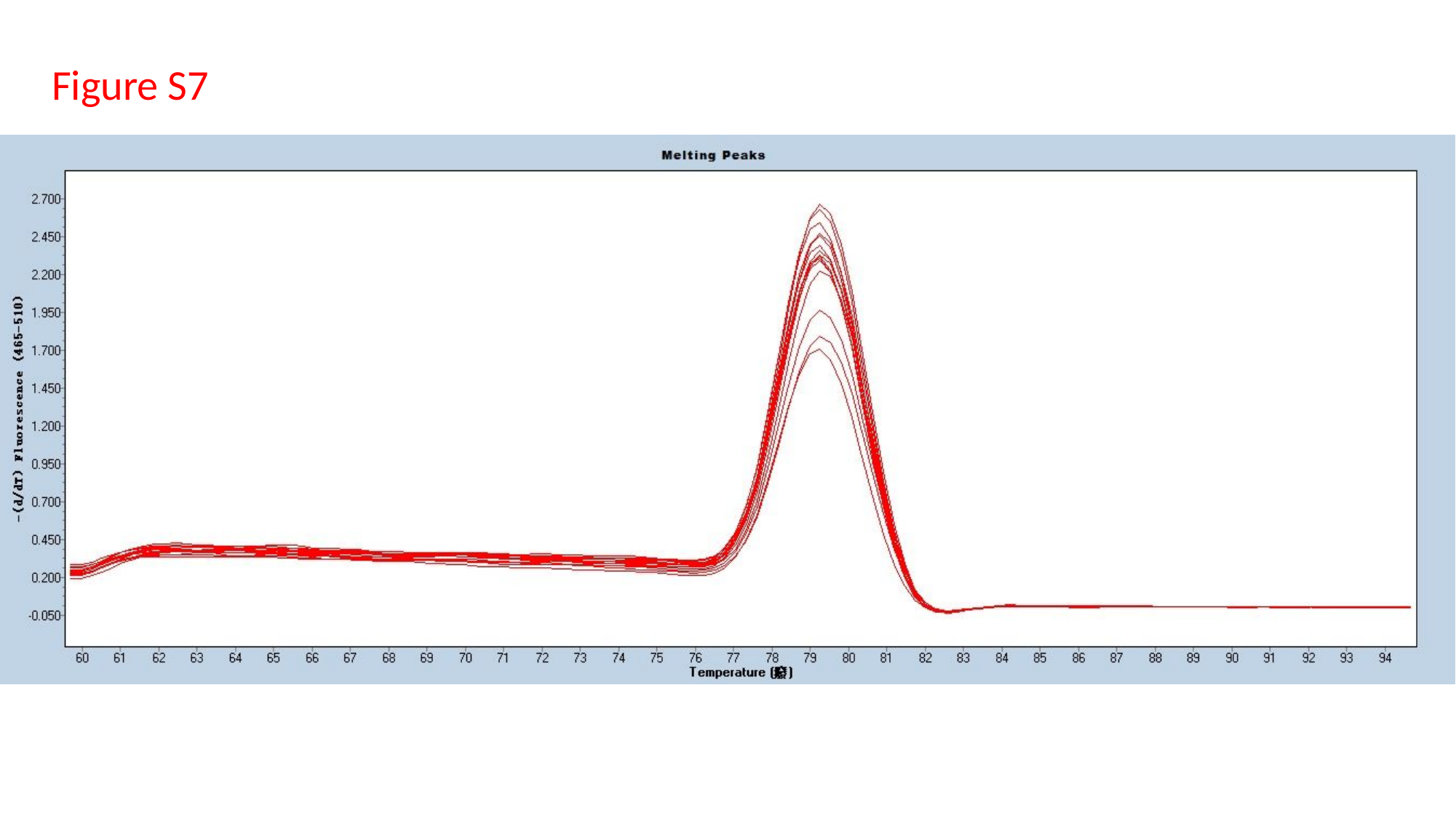

Figure S7

## Slide 55
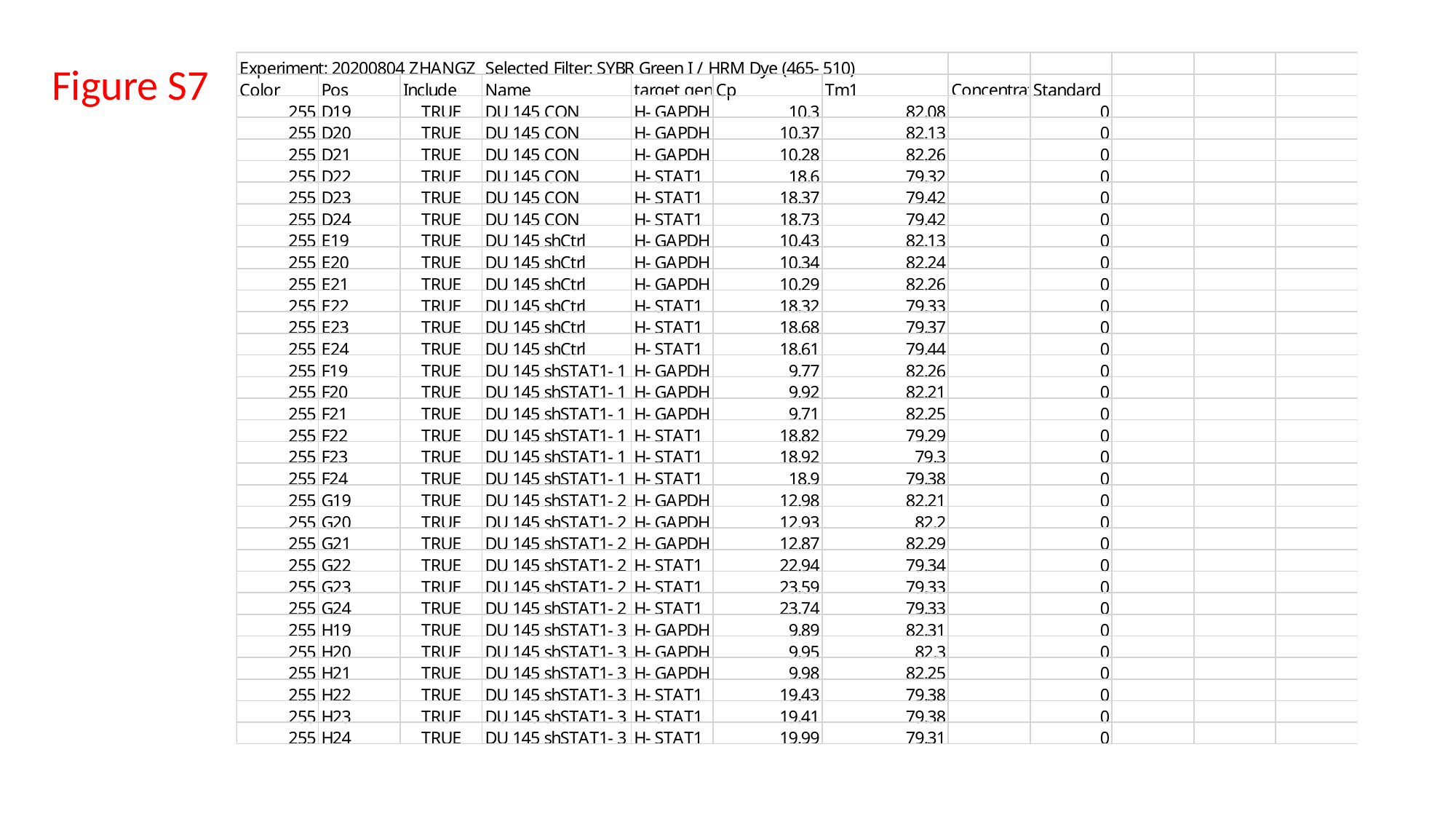

Figure S7

## Slide 56
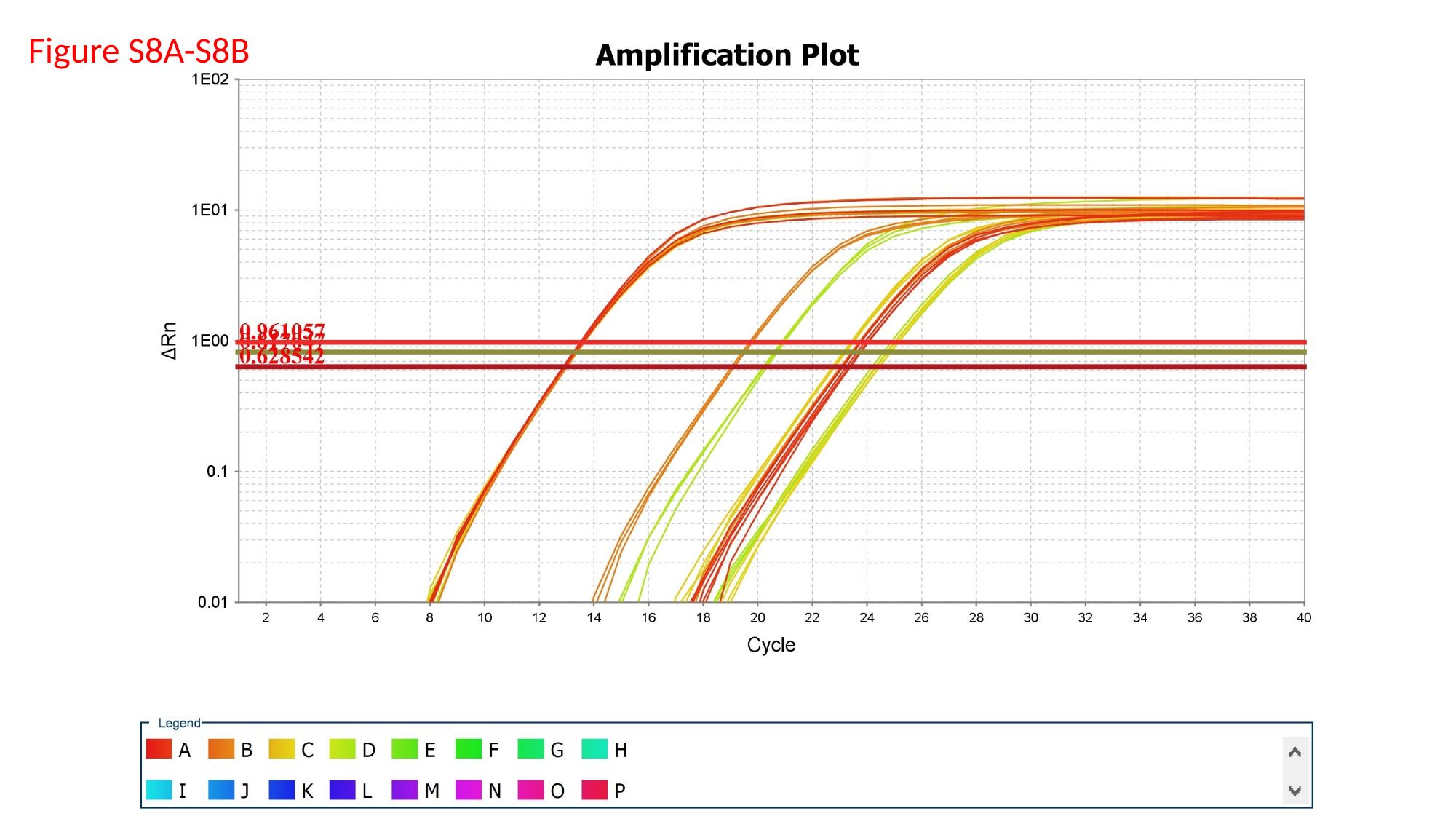

Figure S8A-S8B

## Slide 57
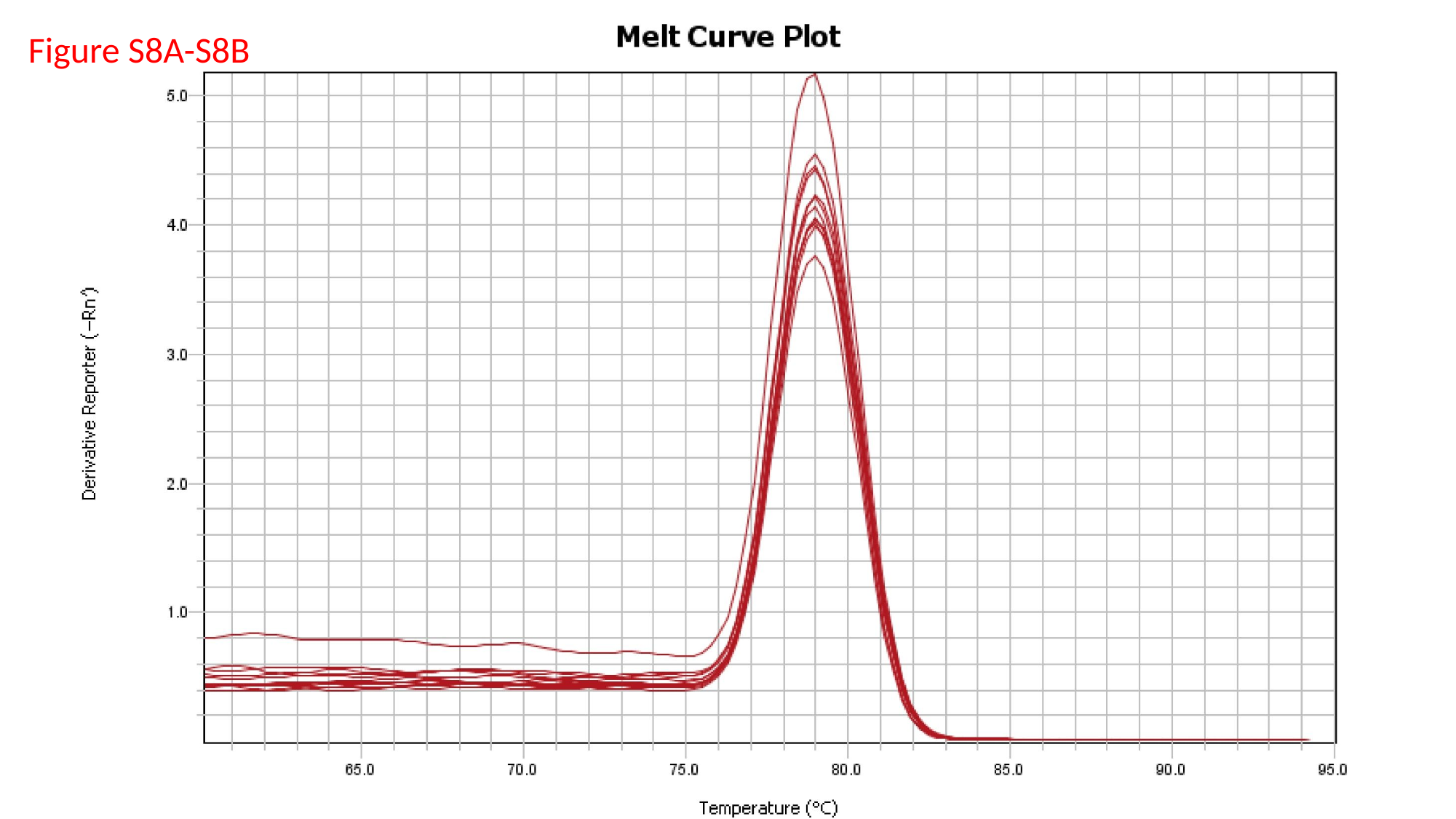

Figure S8A-S8B

## Slide 58
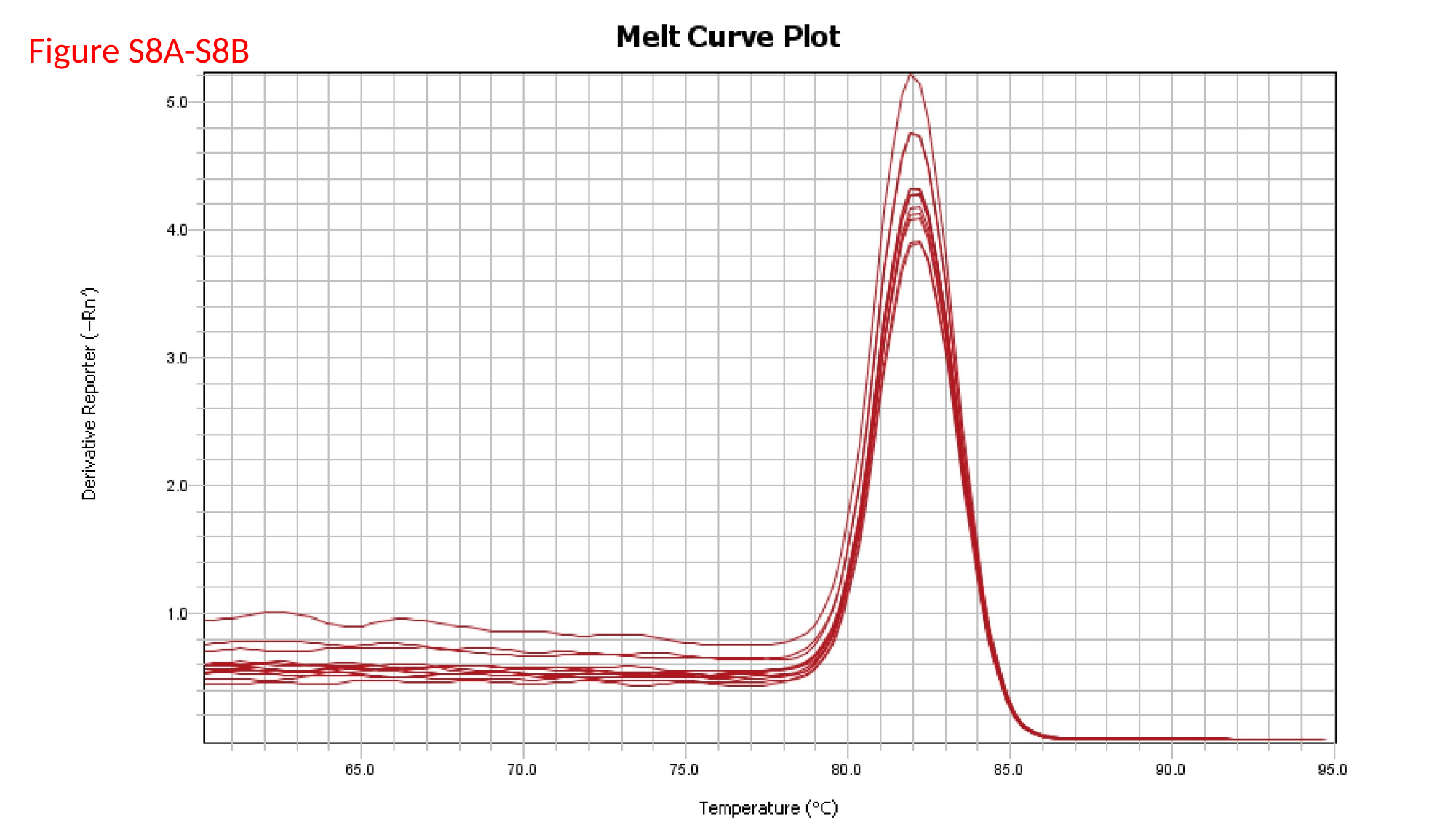

Figure S8A-S8B

## Slide 59
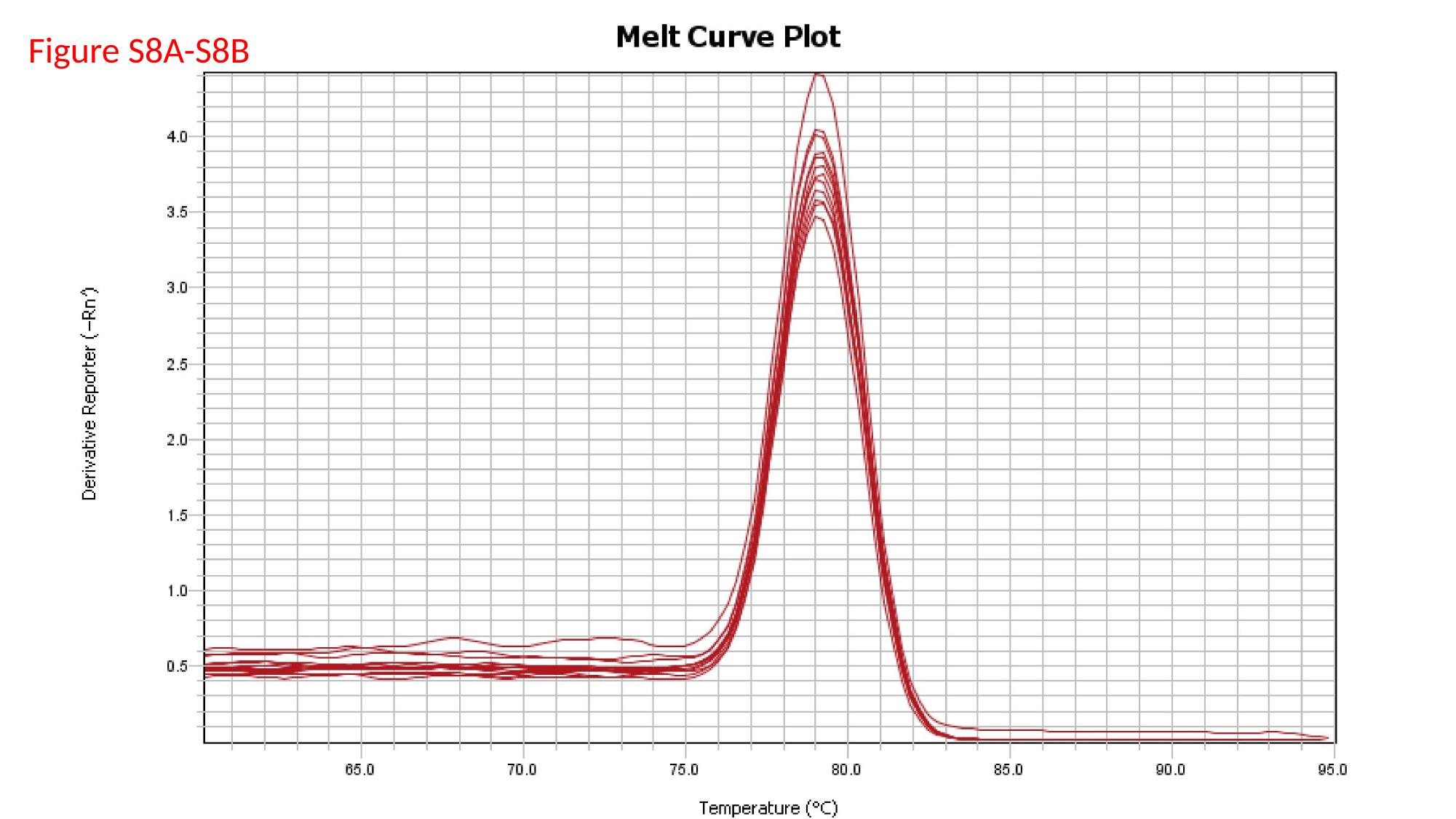

Figure S8A-S8B

## Slide 60
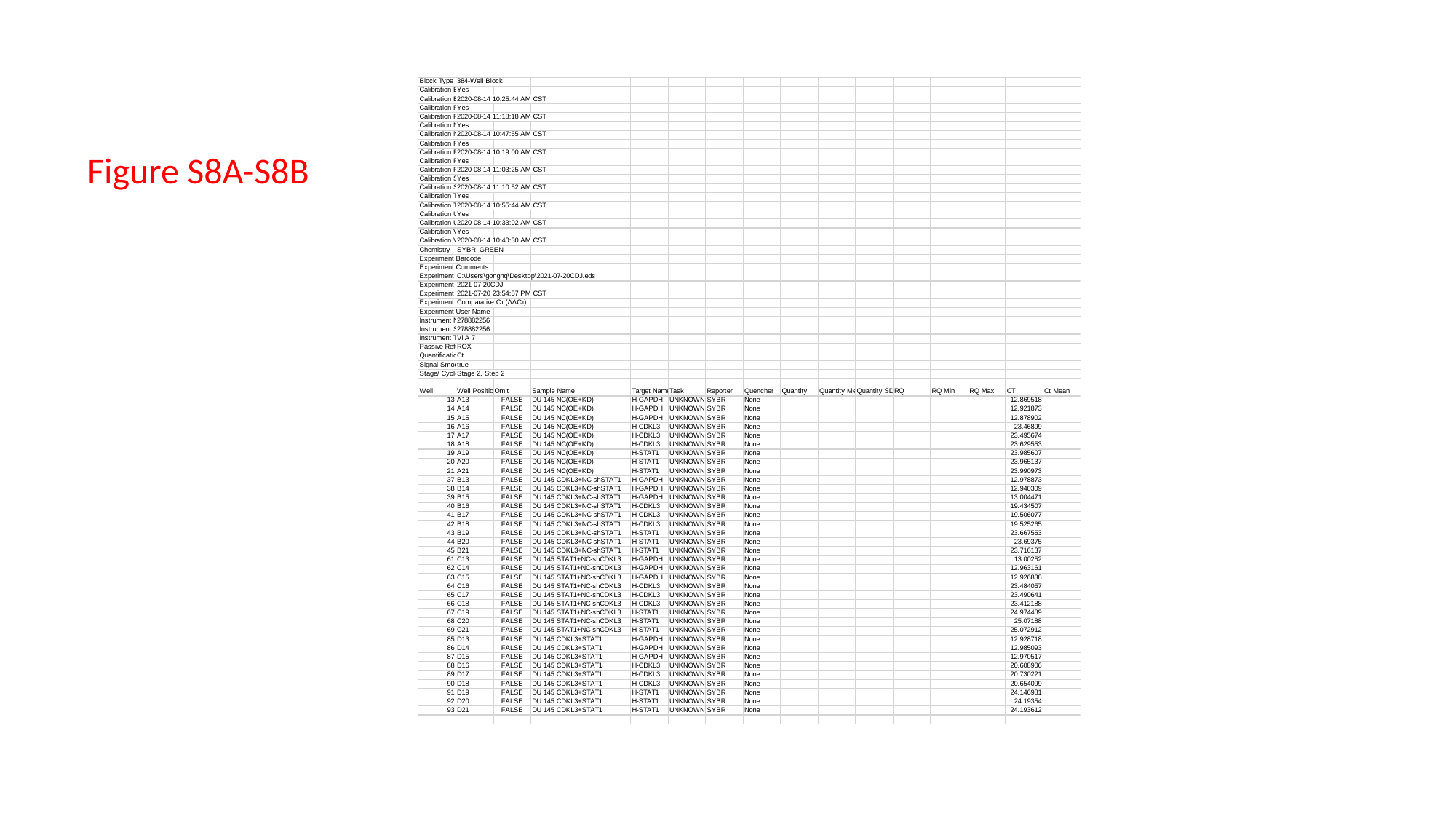

Figure S8A-S8B

## Slide 61
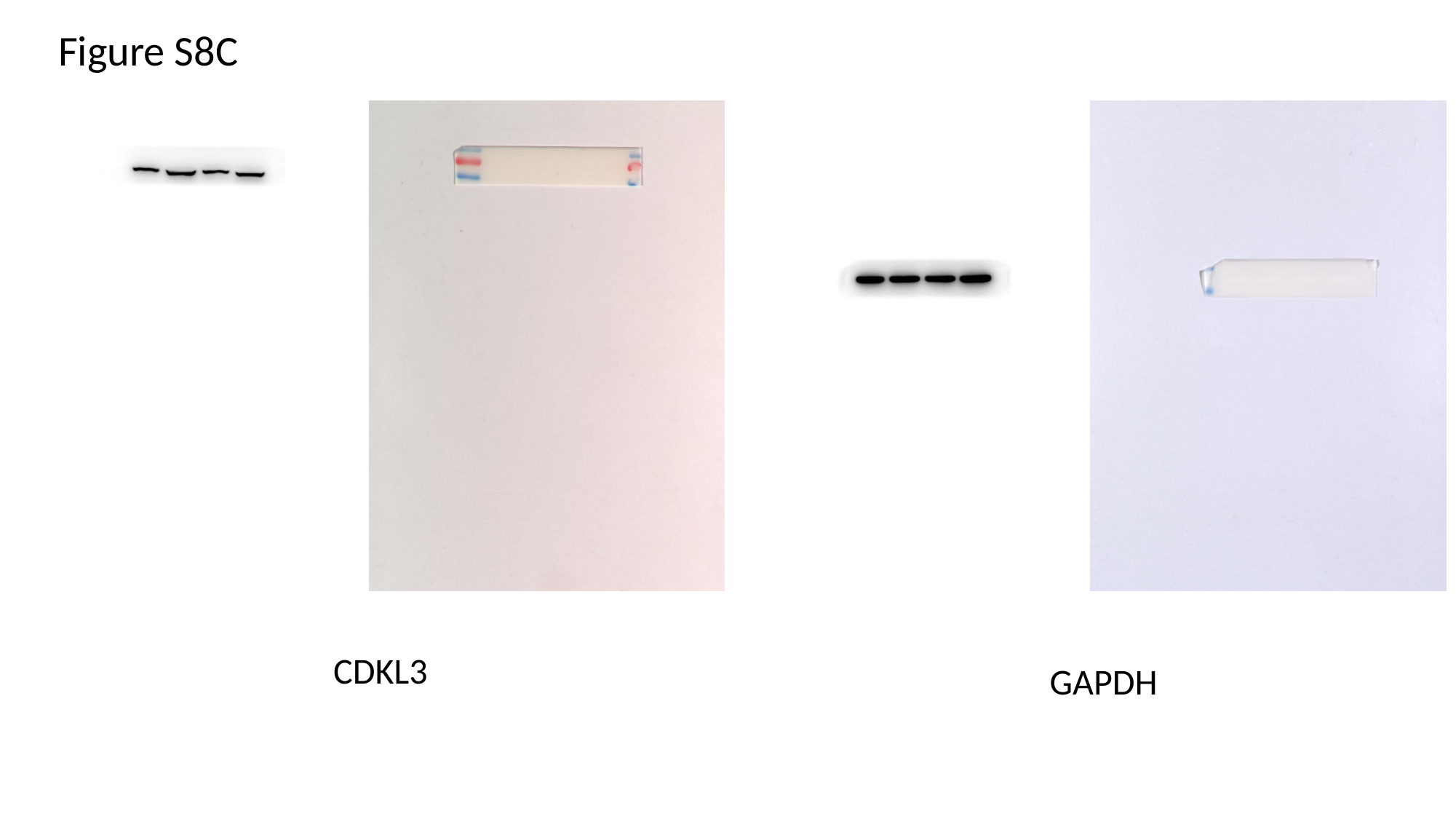

Figure S8C
CDKL3
GAPDH

## Slide 62
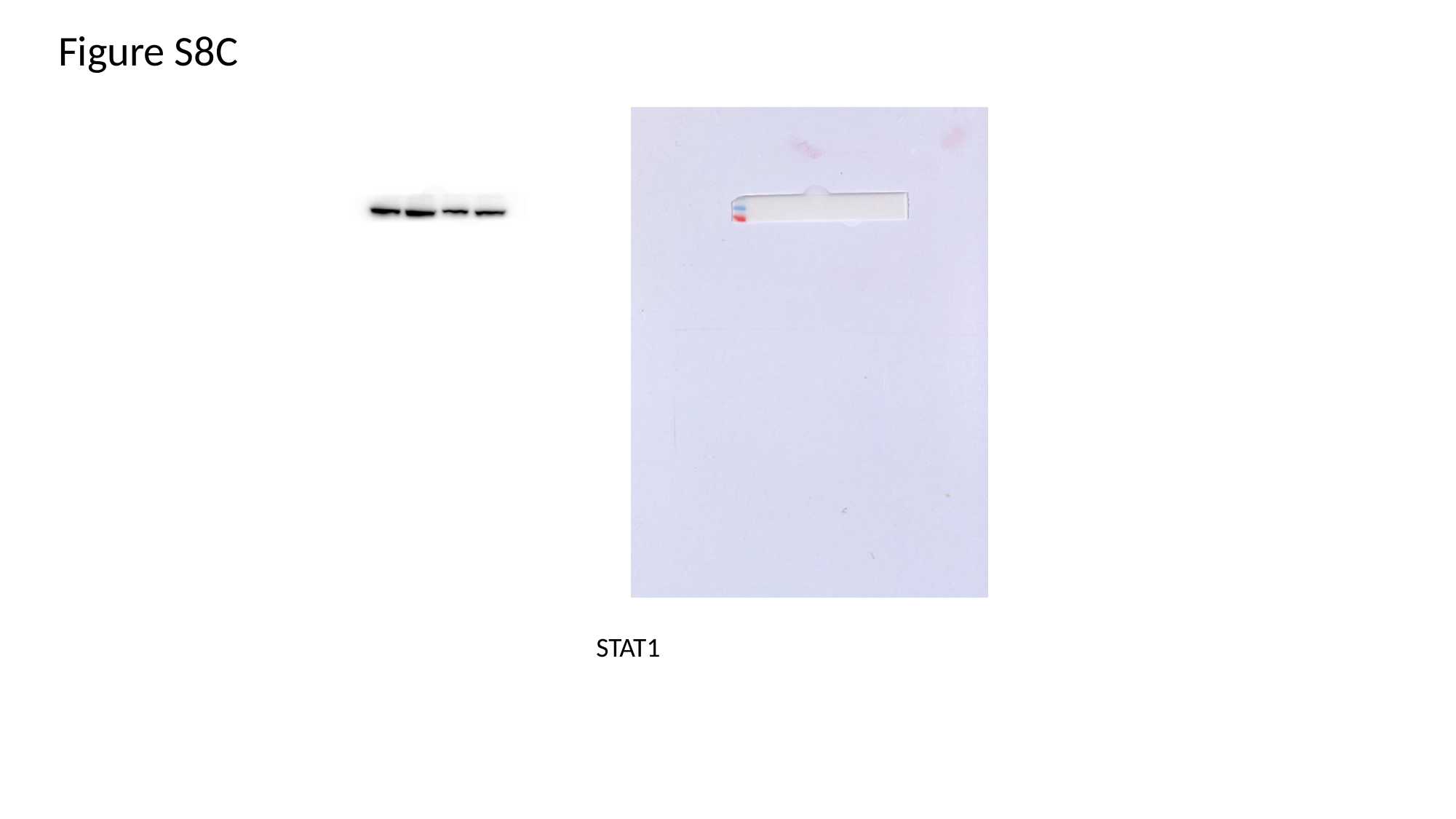

Figure S8C
STAT1
