# Supplementary material for: Inhibition of CDKL3 downregulates STAT1 thus suppressing prostate cancer development
Source: Cell Death Dis. 2023 Mar 10;14(3):189. doi: 10.1038/s41419-023-05694-3 (PMC10006411; doi:10.1038/s41419-023-05694-3)
Supplement: Supplementary file 23 — cddis-author-contribution-form [file 41419_2023_5694_MOESM23_ESM.pdf]

DECLARATION OF CONTRIBUTIONS TO ARTICLE

ADMC

Manuscript Number: CDDIS-21-3209

Journal Name: *Cell Death & Disease* (the 'Journal')

Proposed Title of the Contribution:

Inhibition of CDKL3 downregulates STAT1 thus suppressing prostate cancer development (the 'Contribution)

Author(s):

Qi Jiang, Juan Li, Jingyue Wang, Weibing Zhang (the 'Authors')

For all *CDDis* articles, each person named as an author in the published version must be able to show he or she has contributed substantially to the article.

Authorship credit should be based on 1) substantial contributions to conception and design, acquisition of data, or analysis and interpretation of data; 2) drafting the article or revising it critically for important intellectual content; and 3) final approval of the version to be published. Authors should meet conditions 1, 2 and 3.

Any person who cannot be shown to have made a substantial contribution to the article cannot be listed as an author in the final version. The name of any person who is deemed to have made a minor contribution can, however, appear in the Acknowledgments section of the article.

Please complete the table below to indicate the contributions of all named authors to the manuscript.

Author Full Name:	Specification of Contribution to the Manuscript:
Qi Jiang	13
Juan Li	123
Jingyue Wang	123
Weibing Zhang	123

Please complete the table below to indicate the contributions of all named authors to the figures.

Figure 1:

Qi Jiang, Juan Li, Jingyue Wang

Figure 2:

Qi Jiang, Juan Li, Jingyue Wang

Figure 3:

Qi Jiang, Juan Li, Jingyue Wang

Figure 4:

Qi Jiang, Juan Li, Jingyue Wang

Figure 5:

Qi Jiang, Juan Li, Jingyue Wang

Figure 6:

Qi Jiang, Juan Li, Jingyue Wang

Signed for and on behalf of the Author(s):	Print Name:	Date:
Weibing Zhang	Weibing Zhang	2023.2.3
